# Protective Group-Dependent Iridium-Catalyzed CH Borylations of Levodopa

**DOI:** 10.1021/acs.joc.5c00476

**Published:** 2025-05-27

**Authors:** Cliff Yang, Jinda Fan, Robert E. Maleczka

**Affiliations:** † Department of Chemistry, 3078 Michigan State University, 578 South Shaw Lane, East Lansing, Michigan 48824, United States; ‡ Department of Radiology, Michigan State University, 846 Service Road, East Lansing, Michigan 48824, United States; § Institute for Quantitative Health Science & Engineering, 775 Woodlot Drive, East Lansing, Michigan 48824, United States

## Abstract

Despite being an important aromatic amino acid, iridium-catalyzed borylation (CHB) of levodopa has not been reported. Via the application of carefully chosen protecting groups for the catechol moiety, the steric hindrance around the arene can be reduced, enabling selective C5 CHB of levodopa. Methylene and boronic ester protecting groups were explored, the latter of which is deprotected in situ to yield the free catechol.

## Graphical abstract

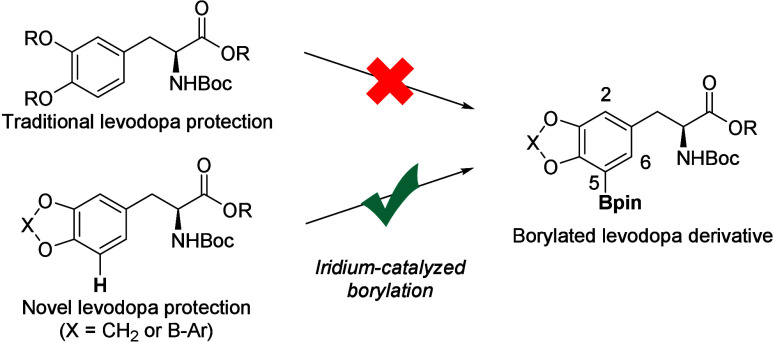

Levodopa is an important aromatic amino acid and a precursor to both dopamine and fluorodopa.^1,2^ Previous routes to fluorodopa have used a three-step process in which levodopa is halogenated at C6, followed by a Miyaura borylation and finally a Bpin/^18^F exchange.^3,4^ Iridium-catalyzed CH borylation (CHB) has also been found to be similarly useful in the radiofluorination of some protected amino acid derivatives. However, CHB of levodopa has not been reported.^5^ This is perhaps not surprising owing to the regiochemical outcomes of CHB being primarily driven by sterics and the steric hindrance about the levodopa arene (C2, C5, and C6 being *ortho* to the alkyl chain and/or the catechol hydroxyls).

To test and unequivocally document the impact of levodopa’s steric environment on CHBs, levodopa derivative **1** (Figure 1), in which the catechol was left unprotected, failed to borylate when subjected to [Ir­(OMe)­cod]_2_, B_2_pin_2_, and common ligands such as dtbpy and tmphen. Several dialkyl ether analogues (**2**–**5**) were then synthesized. They also proved to be resistant to standard CHB conditions. Even when borylations utilizing aniline^6^ or dimethyl amine^7^ directing effects were employed for **4** and **5**, no borylation was observed. These unsuccessful borylations confirmed that protecting the arene ring of levodopa with simple ethers hinders CHB.

**Figure 1:**
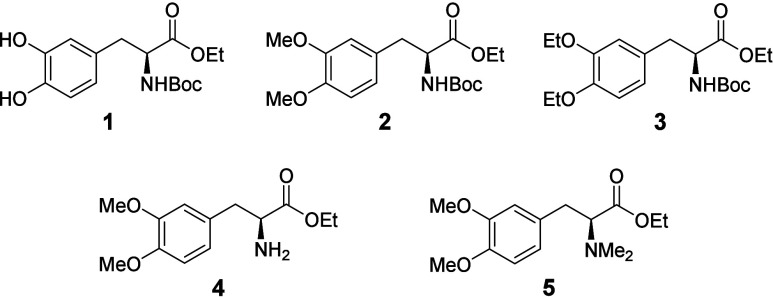
Derivatives of levodopa with traditional protecting groups for the catechol moiety.

While the hydroxyls of catechol can be protected individually, they can also be protected via a bridging methylene. This change in protection can cause drastic changes in the selectivity of borylation. Previous work in our lab demonstrated that while borylation of veratrole yielded the *meta* product, borylation of benzodioxole gave only the *ortho* product.^8^ This effect, if applied to levodopa, could reduce the steric hindrance of the arene and allow CHB to form via iridium catalysis.

Since benzodioxole is known to undergo CHB,^8^ we synthesized derivatives (**6**–**10**) of levodopa, in which the catechol was protected with a methylene bridge, emulating the structure of benzodioxole. In contrast to the unsuccessful borylations of **1**–**5**, these derivatives of levodopa underwent successful CHB (Scheme 1). For derivatives **6**–**10**, the protecting group on the α-amine did not negatively affect the borylation, as *N*-Boc, *N*-methyl, and *N*,*N*-dimethyl groups were all tolerated during CHB. Borylation occurred selectively *ortho* to the benzodioxole moiety, since the methylene bridge tied back the catechol, reducing the steric hindrance at C5.^8^ No borylation was observed *ortho* to the alkyl chain of the amino acid (C2 or C6) for any derivative tested.

**Scheme 1:**
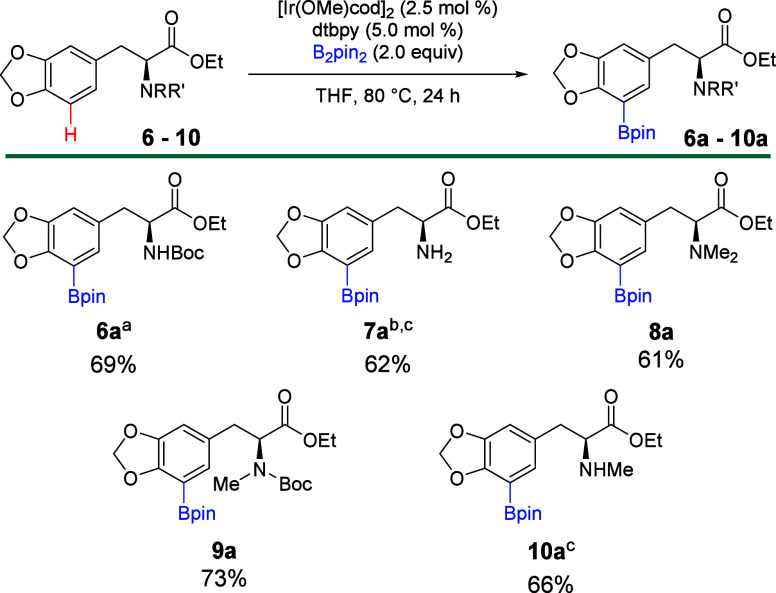
Iridium-Catalyzed Borylation of Levodopa Derivatives Including the Benzodioxole Moiety^a^

a (a) See the Supporting Information for detailed conditions, (b) isolated as the trifluoroacetic acid salt, (c) 3.0 equiv B_2_pin_2_ required for complete conversion (see SI).

The failure to borylate at C2 and C6 was not entirely surprising, as CHB of amino acids *ortho* to their alkyl chain has not been reported.^9–11^ However, we question if C6 could be borylated by utilizing directing effects previously employed in the *ortho* selective CHB of anilines^6^ and dimethylamines.^7^ In those cases, coordination between the active iridium catalyst and the substrate nitrogen atoms directs borylation *ortho* to the nitrogen-containing functional group. We hypothesized that the same Ir–N coordination combined with the flexibility of the alkyl chain might direct borylation at C6 of levodopa. In practice, neither derivative **7** nor **8** was amenable to this approach as only the starting derivatives were observed after exposure to *ortho* directing CHB conditions, even when using less hindered diboron glycolates such as those derived from ethylene glycol^6^ or 1,2-butanediol.

Our focus returned to C5-borylated **6a** and its post-CHB modification. Once CHB occurs at C5, the boronic ester can be converted into different functional groups via mild reactions. Borylated levodopa derivative **6a** underwent deuterodeborylation,^12^ oxidation,^13^ and Suzuki coupling^14^ (Scheme 2). These reactions demonstrate the utility of iridium-catalyzed CHB in the late-stage functionalization of sterically hindered catechols.

**Scheme 2:**
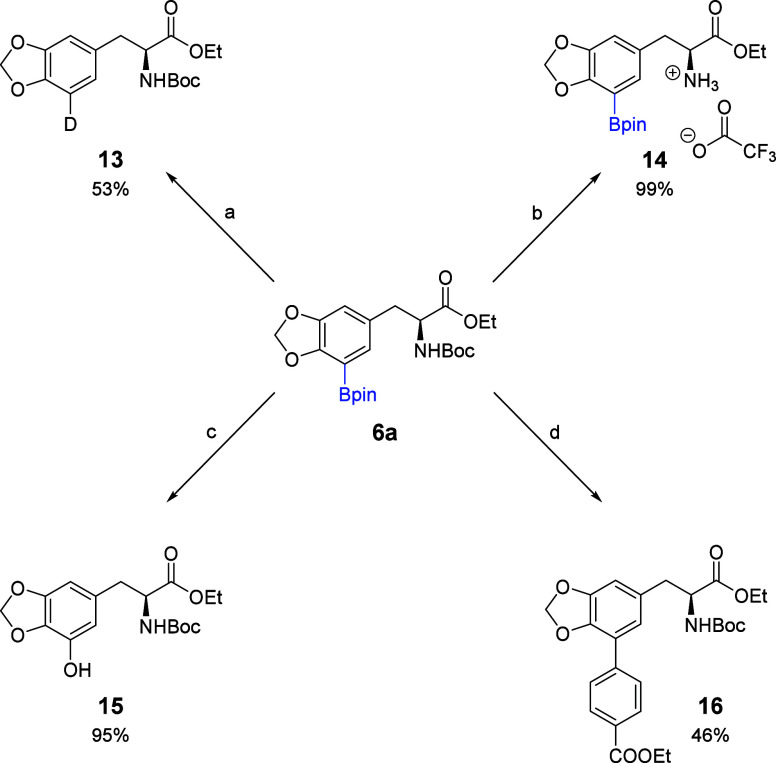
Post Borylation Functionalization of Levodopa^a^

a See the Supporting Information for detailed conditions. (a) [Ir­(OMe)­cod]_2_ and D_2_O. (b) TFA in DCM. (c) Aqueous oxone. (d) Pd­(dppf)­Cl_2_, K_3_PO_4_, and aryl bromide.

*N*-Boc deprotection was nearly quantitative and occurred without a loss of the Bpin (Scheme 2). In contrast, attempts to deprotect the catechol were unsuccessful due to the stability of the methylene bridge. A structurally similar protecting group for diols is a boronic ester derived from condensation of the diol with a boronic acid. Previous reports have shown that these boronic ester protecting groups can be installed on silsesquioxanes and carbohydrates and easily removed after they are no longer required.^15,16^

Thus, we reacted **1** with 4-methoxyphenyl boronic acid to form capped boronic ester derivative **11** (Scheme 3). This derivative underwent CHB at C5 like the other derivatives. During CHB, however, the boronic ester cap was lost in situ, allowing direct access to **12**. Here, CHB likely occurs before the loss of the boronic ester, as unprotected derivative **1** was resistant to borylation under similar conditions. Though the unoptimized yield of **12** was a modest 43%, the use of a boronic ester as the catechol protecting group has the advantage of enabling selective C5 CHB and obviating the need for a separate deprotection step.

**Scheme 3:**
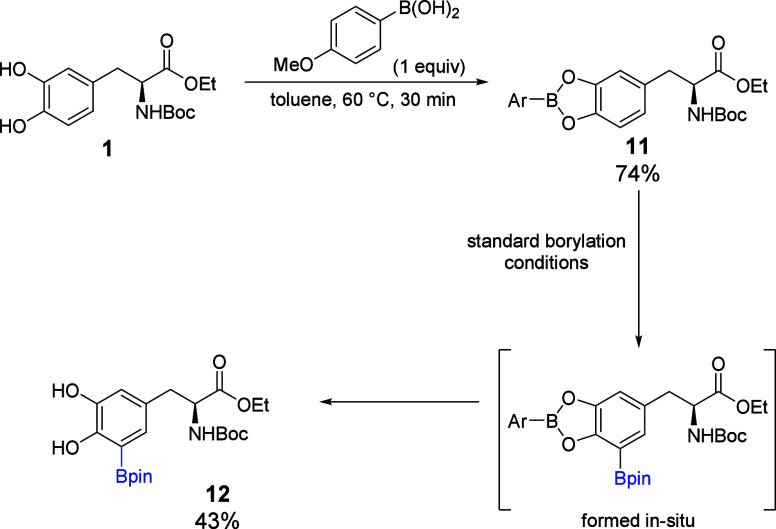
Borylation of a Levodopa Derivative Protected by Boronic Acid to Yield the Deprotected Catechol

In summary, the choice of protecting group for the catechol moiety of levodopa plays a significant role in CHB success or failure. CHB is not feasible when levodopa is protected via simple ethers. However, bridging the catechol hydroxyls with a methylene group reduces the steric hindrance about the arene and allows selective CHB at C5. Moreover, employing a boronic ester bridge offers the additional advantage of in situ deprotection to afford the free catechol. These observations provide insights into the positive and negative effects that different protecting groups have on the CHB of sterically hindered catechols, which is valuable for both late-stage functionalization strategies and the application of machine learning to organic synthesis.

## Supplementary Material



## Data Availability

The data underlying this study are available in the published article and its Supporting Information.

## Notes

Experimental procedures, including preparation of starting materials, compound characterization data, and product spectra (PDF)
