# Supplementary material for: Protective Group-Dependent Iridium-Catalyzed CH Borylations of Levodopa
Source: J Org Chem. 2025 May 27;90(22):7507–9. doi: 10.1021/acs.joc.5c00476 (PMC12150333; doi:10.1021/acs.joc.5c00476)

Supporting Information

Protective Group Dependent Iridium-Catalyzed CH Borylations of Levodopa

Cliff Yang,[a] Jinda Fan,[a,b,c] and Robert E. Maleczka, Jr.[a,*]

[a]Department of Chemistry, Michigan State University, 578 South Shaw Lane, East Lansing, Michigan 48824, USA

[b]Department of Radiology, Michigan State University, 846 Service Road, East Lansing, Michigan 48824, USA

[c]Institute for Quantitative Health Science & Engineering, 775 Woodlot Drive, East Lansing MI 48824, USA

[*]Corresponding Author: maleczka@chemistry.msu.edu

Table of Contents

Table of Contents **S1**

General Information **S3**

Experimental Methods **S4**

Synthesis of ethyl (*S*)-2-((*tert*-butoxycarbonyl)amino)-3-(3,4-dihydroxyphenyl)propanoate (**1**) S4

Synthesis of ethyl (*S*)-2-((*tert*-butoxycarbonyl)amino)-3-(3,4-dimethoxyphenyl)propanoate (**2**) S5

Synthesis of ethyl (*S*)-2-((*tert*-butoxycarbonyl)amino)-3-(3,4-diethoxyphenyl)propanoate (**3**) S6

Synthesis of ethyl (*S*)-2-amino-3-(3,4-dimethoxyphenyl)propanoate (**4**) S7

Synthesis of ethyl (*S*)-3-(3,4-dimethoxyphenyl)-2-(dimethylamino)propanoate (**5**) S7

Synthesis of ethyl (*S*)-3-(benzo[*d*][1,3]dioxol-5-yl)-2-((*tert*-butoxycarbonyl)amino) propanoate (**6**) S8

Synthesis of ethyl (*S*)-2-amino-3-(benzo[*d*][1,3]dioxol-5-yl)propanoate (**7**) S9

Synthesis of ethyl (*S*)-3-(benzo[*d*][1,3]dioxol-5-yl)-2-(dimethylamino)propanoate (**8**) S10

Synthesis of ethyl (*S*)-3-(benzo[*d*][1,3]dioxol-5-yl)-2-((*tert*-butoxycarbonyl)(methyl)amino) propanoate (**9**) S11

Synthesis of ethyl (*S*)-3-(benzo[*d*][1,3]dioxol-5-yl)-2-(methylamino)propanoate (**10**) S12

Synthesis of ethyl (*S*)-2-((*tert*-butoxycarbonyl)amino)-3-(2-(4-methoxyphenyl) benzo[*d*][1,3,2]dioxaborol-5-yl)propanoate (**11**) S12

Synthesis of ethyl (*S*)-2-((*tert*-butoxycarbonyl)amino)-3-(7-(4,4,5,5-tetramethyl-1,3,2-dioxaborolan-2-yl)benzo[*d*][1,3]dioxol-5-yl)propanoate (**6a**) S13

Synthesis of (*S*)-1-ethoxy-1-oxo-3-(7-(4,4,5,5-tetramethyl-1,3,2-dioxaborolan-2-yl) benzo[*d*][1,3]dioxol-5-yl)propan-2-aminium trichloroacetate (**7a**) S14

Synthesis of ethyl (*S*)-2-(dimethylamino)-3-(7-(4,4,5,5-tetramethyl-1,3,2-dioxaborolan-2-yl) benzo[*d*][1,3]dioxol-5-yl)propanoatetrichloroacetate (**8a**) S15

Synthesis of ethyl (*S*)-2-((*tert*-butoxycarbonyl)(methyl)amino)-3-(7-(4,4,5,5-tetramethyl-1,3,2-dioxaborolan-2-yl)benzo[*d*][1,3]dioxol-5-yl)propanoate (**9a**) S16

Synthesis of ethyl (*S*)-2-(methylamino)-3-(7-(4,4,5,5-tetramethyl-1,3,2-dioxaborolan-2-yl) benzo[*d*][1,3]dioxol-5-yl)propanoate (**10a**) S17

Synthesis of ethyl (*S*)-2-((*tert*-butoxycarbonyl)amino)-3-(3,4-dihydroxy-5-(4,4,5,5-tetramethyl-1,3,2-dioxaborolan-2-yl)phenyl)propanoate (**12**) S18

Synthesis of ethyl (*S*)-3-(benzo[*d*][1,3]dioxol-5-yl-7-d)-2-((tert-butoxycarbonyl)amino) propanoate (**13**) S18

Synthesis of (*S*)-1-ethoxy-1-oxo-3-(7-(4,4,5,5-tetramethyl-1,3,2-dioxaborolan-2-yl) benzo[*d*][1,3]dioxol-5-yl)propan-2-aminium trifluoroacetate (**14**) S19

Synthesis of ethyl (*S*)-2-((*tert*-butoxycarbonyl)amino)-3-(7-hydroxybenzo[*d*][1,3]dioxol-5-yl) propanoate (**15**) S20

Synthesis of ethyl (*S*)-4-(6-(2-((*tert*-butoxycarbonyl)amino)-3-ethoxy-3-oxopropyl) benzo[*d*][1,3]dioxol-4-yl)benzoate (**16**) S21

References S23

NMR Spectra S24

General Information

All commercially available chemicals were used as received unless otherwise indicated.

Tetrahydrofuran (THF) for borylation reactions was refluxed over sodium/benzophenone ketyl, distilled and degassed twice before borylation. All borylations were set up in a N_2 filled glovebox.

^{1}H and ^{13}C{^{1}H} NMR spectra were recorded on a Varian 500 MHz DD2 Spectrometer equipped with a 1H-19F/15N-31P 5 mm Pulsed Field Gradient (PFG) Probe. Spectra taken in $CDCl_3$ were referenced to 7.26 ppm in ^{1}H NMR and 77.2 ppm in ^{13}C{^{1}H} NMR. Reference values are based on previously reported values.[1]

All coupling constants are apparent J values measured at the indicated field strengths in Hertz (s =singlet, d = doublet, t = triplet, q = quartet, dd = doublet of doublets, ddd = doublet of doublet of doublets, bs = broad singlet). NMR spectra were processed for display using the MNova software program with only phasing and baseline corrections applied.

Structural assignments were made with additional information from gCOSY, gHSQC, and gHMBC experiments.

High-resolution mass spectra (HRMS) were obtained at the Mass Spectrometry Service Center at Michigan State University using electrospray ionization (ESI^+ or ESI^-) on quadrupole time-of-flight (Q-TOF) instruments.

Optical activities ($[\alpha]_D$) were measured using a JASCO P-2000 polarimeter. Samples were analyzed at a 1 g / 1 mL concentration in dichloromethane using a 100 mm cell at 22 °C.

Experimental Methods

Synthesis of ethyl (*S*)-2-((*tert*-butoxycarbonyl)amino)-3-(3,4-dihydroxyphenyl)propanoate (1)

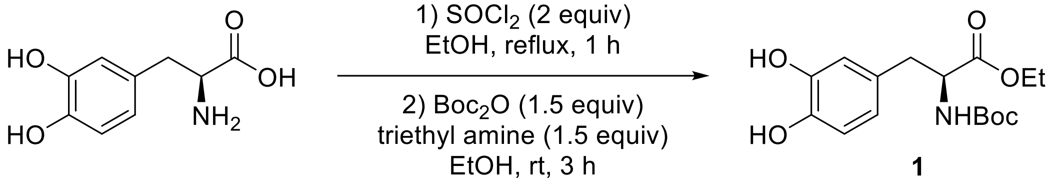

1
61%

A suspension of L-DOPA (50.00 mmol, 9.87 g) in ethanol (100 mL) in a round bottom flask was cooled in an ice bath. While stirring, $SOCl_2$ (100.00 mmol, 11.90 g, 7.25 mL) was added dropwise. The mixture was stirred at 0 °C for 5 min to form a clear solution before being removed from the ice bath and refluxed via heating in an aluminum bead bath for 1 h. After reflux, volatile solvents were evaporated under reduced pressure to yield a yellow oil. The residue was redissolved in ethanol (100 mL). Triethyl amine (75.00 mmol, 7.58 g, 10.52 mL) and Boc_2O (75.00 mmol, 16.36 g, 17.24 mL) were added and the solution was stirred at room temperature for 3 h. Volatile solvents were evaporated and the residue dissolved in 150 mL of 1 M HCl. The resulting solution was extracted three times with 100 mL of ethyl acetate. The organic layers were dried with Na_2SO_4, filtered to remove Na_2SO_4, then volatile solvents were evaporated. The resulting residue was recrystallized from hexane to yield the product **1** as a white solid (10.039 g, 61%).

Melting point: 94-95 °C

^{1}H-NMR (500 MHz, $CDCl_3$): δ 6.76 – 6.61 (m, 2H), 6.52 (dd, *J* = 8.2, 2.0 Hz, 1H), 6.32 (s, 2H), 5.10 (d, *J* = 8.5 Hz, 1H), 4.48 (dt, *J* = 8.4, 6.2 Hz, 1H), 4.23 – 4.12 (m, 2H), 3.01 – 2.86 (m, 2H), 1.41 (s, 9H), 1.23 (t, *J* = 7.1 Hz, 3H).

^{13}C{^{1}H}-NMR (126 MHz, $CDCl_3$): δ 172.5, 155.8, 144.1, 143.2, 128.4, 121.7, 116.4, 115.4, 80.7, 61.8, 54.9, 38.0, 28.5, 14.3.

HRMS (ESI) m/z: $[M + Na]^+$ calculated for $C_{16}H_{23}NaNO_6^+$ 348.1418; found 348.1417.

$[\alpha]_D$ = +44.57°.

Synthesis of ethyl (*S*)-2-((*tert*-butoxycarbonyl)amino)-3-(3,4-dimethoxyphenyl)propanoate (2)

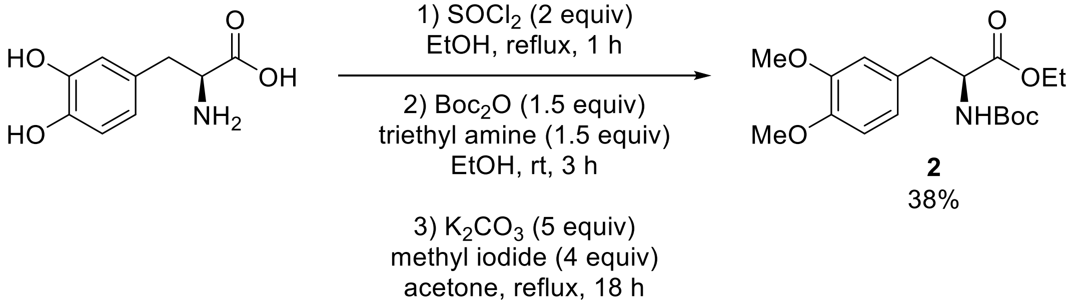

2
38%

A suspension of L-DOPA (15.00 mmol, 2.96 g) in ethanol (30 mL) in a round bottom flask was cooled in an ice bath. While stirring, $SOCl_2$ (30.00 mmol, 3.57 g, 2.18 mL) was added dropwise. The mixture was stirred at 0 °C for 5 min to form a clear solution before being removed from the ice bath and refluxed via heating in an aluminum bead bath for 1 h. After reflux, volatile solvents were evaporated under reduced pressure to yield a yellow oil. The residue was redissolved in ethanol (30 mL). Triethyl amine (22.50 mmol, 2.27 g, 3.16 mL) and Boc_2O (22.50 mmol, 4.91 g, 5.17 mL) were added and the solution was stirred at room temperature for 3 h. Volatile solvents were evaporated and the residue dissolved in 50 mL of 1 M HCl. The resulting solution was extracted three times with 50 mL of ethyl acetate. The organic layers were dried with Na_2SO_4, filtered to remove Na_2SO_4, then volatile solvents were evaporated. The residue was dissolved in acetone (100 mL) and potassium carbonate (75.00 mmol, 10.35 g) and methyl iodide (60.00 mmol, 8.51 g, 3.74 mL) were added. The reaction was refluxed via heating in an aluminum bead bath for 18 h, then filtered to remove the solids. Following the removal of solvents, the resulting solid was recrystallized from hexane to yield the purified product **2** as a white solid (2.002 g, 38%). The NMR spectra were consistent with previously reported spectra.[2]

Melting point: 77-78 °C.

^{1}H-NMR (500 MHz, $CDCl_3$): δ 6.78 (d, *J* = 8.1 Hz, 1H), 6.67 (dd, *J* = 8.0, 2.0 Hz, 1H), 6.65 (d, *J* = 2.0 Hz, 1H), 4.98 (d, *J* = 8.3 Hz, 1H), 4.76 – 4.40 (m, 1H), 4.16 (q, *J* = 7.1 Hz, 2H), 3.85 (s, 3H), 3.85 (s, 3H), 3.03 (dd, *J* = 8.8, 6.0 Hz, 2H), 1.42 (s, 9H), 1.24 (t, *J* = 7.1 Hz, 3H).

$^{13}C\{^1H\}$-NMR (126 MHz, $CDCl_3$): δ 172.1, 155.2, 148.9, 148.2, 128.6, 121.6, 112.5, 111.3, 80.0, 61.5, 56.0, 55.9, 54.7, 38.0, 28.5, 14.3.

GC-MS: Calculated 353.18, found 353.20.

HRMS (ESI) m/z: $[M + Na]^+$ calculated for $C_{18}H_{27}NNaO_6^+$ 376.173; found 376.1739.

$[\alpha]_D$ = +31.33°.

Synthesis of ethyl (*S*)-2-((*tert*-butoxycarbonyl)amino)-3-(3,4-diethoxyphenyl)propanoate (3)

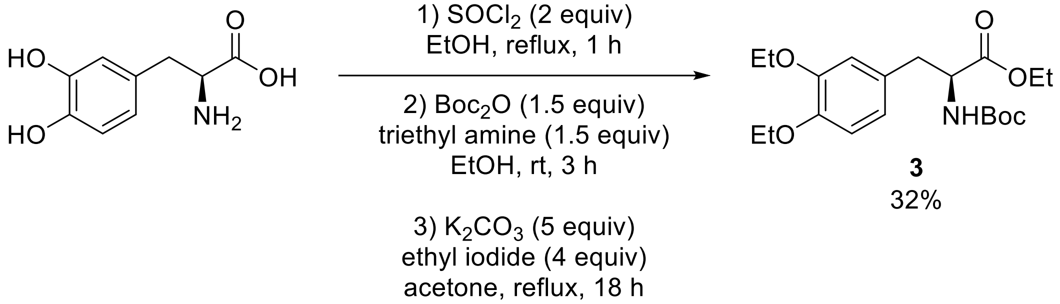

A suspension of L-DOPA (15.00 mmol, 2.96 g) in ethanol (30 mL) in a round bottom flask was cooled in an ice bath. While stirring, $SOCl_2$ (30.00 mmol, 3.57 g, 2.18 mL) was added dropwise. The mixture was stirred at 0 °C for 5 min to form a clear solution before being removed from the ice bath and refluxed via heating in an aluminum bead bath for 1 h. After reflux, volatile solvents were evaporated under reduced pressure to yield a yellow oil. The residue was redissolved in ethanol (30 mL). Triethyl amine (22.50 mmol, 2.27 g, 3.16 mL) and Boc_2O (22.50 mmol, 4.91 g, 5.17 mL) were added and the solution was stirred at room temperature for 3 h. Volatile solvents were evaporated and the residue dissolved in 50 mL of 1 M HCl. The resulting solution was extracted three times with 50 mL of ethyl acetate. The organic layers were dried with Na_2SO_4, filtered to remove Na_2SO_4, then volatile solvents were evaporated. The residue was dissolved in acetone (100 mL) and potassium carbonate (75.00 mmol, 10.35 g) and ethyl iodide (60.00 mmol, 9.38 g, 4.82 mL) were added. The reaction was refluxed via heating in an aluminum bead bath for 18 h, then filtered to remove the solids. Following the removal of solvents, the resulting solid was recrystallized from hexane to yield the purified product **3** as a yellow solid (1.836 g, 32%).

Melting point: 89-90 °C.

^{1}H-NMR (500 MHz, $CDCl_3$): δ 6.78 (d, *J* = 8.1 Hz, 1H), 6.67 (dd, *J* = 8.0, 2.0 Hz, 1H), 6.64 (d, *J* = 2.0 Hz, 1H), 4.97 (d, *J* = 8.3 Hz, 1H), 4.76 – 4.40 (m, 1H), 4.16 (q, *J* = 7.1 Hz, 2H), 3.85 (q *J* = 0.9 Hz, 4H), 3.03 (dd, *J* = 8.8, 6.0 Hz, 2H), 1.42 (m, 15H), 1.24 (t, *J* = 7.1 Hz, 3H).

^{13}C{^{1}H}-NMR (126 MHz, $CDCl_3$): δ 172.1, 155.2, 148.7, 147.9, 128.7, 121.7, 114.8, 113.6, 79.9, 64.7, 64.6, 61.4, 54.6, 38.0, 28.5, 15.0, 15.0, 14.3.

HRMS (ESI) m/z: $[M + Na]^+$ calculated for $C_{20}H_{31}NNaO_6^+$ 404.2043; found 404.2044.

$[\alpha]_D$ = +48.23°.

Synthesis of ethyl (*S*)-2-amino-3-(3,4-dimethoxyphenyl)propanoate (4)

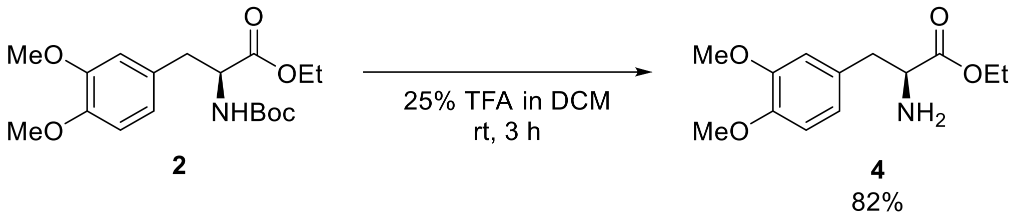

Compound **2** (5.23 mmol, 1.85 g) was dissolved in 15 mL of dichloromethane. 5 mL of trifluoroacetic acid was added. The solution was stirred at room temperature for 3 h. Volatile solvents were removed and the remaining residue was dissolved in a 7.24 M K_2CO_3 solution in water. The aqueous layer was extracted 3 times with 10 mL of $CHCl_3$. The organic layers were dried with Na_2SO_4, filtered to remove Na_2SO_4, then volatile solvents were evaporated to yield **4** as a yellow oil (1.092g, 82%). The NMR spectra were consistent with previously reported spectra.[3]

^{1}H-NMR (500 MHz, $CDCl_3$): δ 6.78 (d, *J* = 8.0 Hz, 1H), 6.75 – 6.68 (m, 2H), 4.15 (q, *J* = 7.1 Hz, 2H), 3.84 (s, 3H), 3.84 (s, 3H), 3.67 (dd, *J* = 7.8, 5.2 Hz, 1H), 3.01 (dd, *J* = 13.6, 5.2 Hz, 1H), 2.80 (dd, *J* = 13.6, 7.8 Hz, 1H), 1.49 (s, 2H), 1.24 (t, *J* = 7.2 Hz, 3H).

^{13}C{^{1}H}-NMR (126 MHz, $CDCl_3$): δ 175.2, 149.0, 148.0, 129.8, 121.5, 112.4, 111.3, 61.0, 56.0, 56.0, 55.9, 40.8, 14.4.

GC-MS: Calculated 253.13, found 253.15.

Synthesis of ethyl (*S*)-3-(3,4-dimethoxyphenyl)-2-(dimethylamino)propanoate (5)

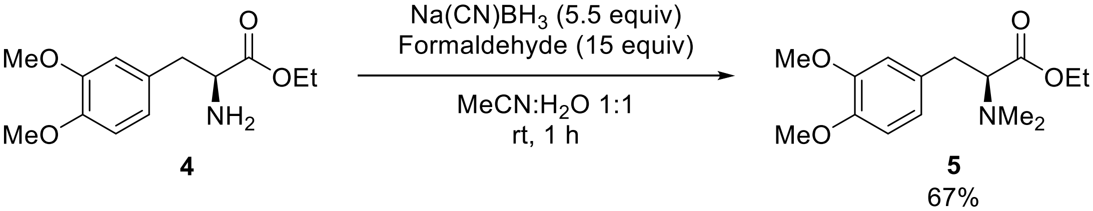

4 (1.97 mmol, 0.50 g) was dissolved in 50 mL of MeCN. Formaldehyde was added as a 37% solution in H_2O (29.55 mmol, 0.89 g, 2.4 mL). Additional H_2O (47.6 mL) was added and the solution was stirred briefly at room temperature. $Na(CN)BH_3$ (10.84 mmol, 0.68 g) was added in three equal additions, separated by five minutes. The pH was adjusted to 7 with glacial acetic acid and the reaction was stirred for an hour at room temperature. Glacial acetic acid was added as needed to maintain a pH of 7. After the hour was over, volatile solvents were removed. The reaction

was quenched with HCl and then basified with aqueous sodium bicarbonate. The aqueous layer was extracted 3 times with ethyl acetate. The organic layers were dried with Na_2SO_4, filtered to remove Na_2SO_4, then volatile solvents were evaporated. Further purification with basic alumina chromatography with 80:19:1 EtOAc:hexane:TEA yielded the pure product **5** as a clear oil (0.372 g, 67%). This procedure was based on a previously reported reaction.[4]

^{1}H-NMR (500 MHz, $CDCl_3$): δ 6.77 (d, *J* = 8.2 Hz, 1H), 6.75 – 6.70 (m, 2H), 4.08 (qq, *J* = 10.8, 7.1 Hz, 2H), 3.85 (s, 3H), 3.84 (s, 3H), 3.38 (dd, *J* = 9.7, 5.4 Hz, 1H), 3.00 (dd, *J* = 13.4, 9.8 Hz, 1H), 2.93 – 2.84 (m, 1H), 2.40 (s, 6H), 1.16 (t, *J* = 7.1 Hz, 3H).

^{13}C{^{1}H}-NMR (126 MHz, $CDCl_3$): δ 171.5, 148.8, 147.7, 130.7, 121.2, 112.4, 111.2, 69.8, 60.2, 55.9, 55.9, 42.0, 35.8, 14.5.

GC-MS: Calculated 281.16, found 281.15.

HRMS (ESI) m/z: $[M + H]^+$ calculated for $C_{15}H_{24}NO_4^+$ 282.1700; found 282.1708.

$[\alpha]_D = +11.34°$.

Synthesis of ethyl (*S*)-3-(benzo[*d*][1,3]dioxol-5-yl)-2-((*tert*-butoxycarbonyl)amino) propanoate (6)

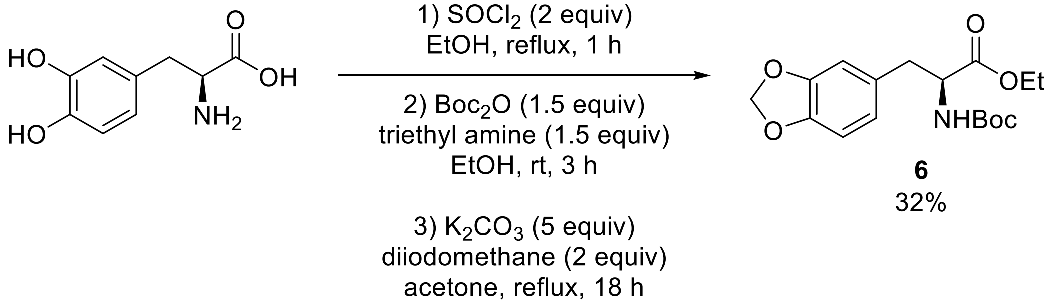

A suspension of L-DOPA (50.00 mmol, 9.85 g) in ethanol (100 mL) in a round bottom flask was cooled in an ice bath. While stirring, $SOCl_2$ (100.00 mmol, 3.57 g, 11.89 g, 7.35 mL) was added dropwise. The mixture was stirred at 0 °C for 5 min to form a clear solution before being removed from the ice bath and refluxed via heating in an aluminum bead bath for 1 h. After reflux, volatile solvents were evaporated under reduced pressure to yield a yellow oil. The residue was redissolved in ethanol (100 mL). Triethyl amine (75.00 mmol, 7.59 g, 10.54 mL) and Boc_2O (75.00 mmol, 16.36 g, 17.24 mL) were added and the solution was stirred at room temperature for 3 h. Volatile solvents were evaporated and the residue dissolved in 100 mL of 1 M HCl. The resulting solution was extracted three times with 75 mL of ethyl acetate. The organic layers were dried with Na_2SO_4,

filtered to remove Na_2SO_4, then volatile solvents were evaporated. The residue was dissolved in acetone (200 mL) and potassium carbonate (250.00 mmol, 34.52 g) and diiodomethane (100.00 mmol, 26.68 g, 8.06 mL) were added. The reaction was refluxed via heating in an aluminum bead bath for 18 h, then filtered to remove the solids. Following the removal of solvents, the product was purified using silica-gel column chromatography with 60:20:20 hexane:chloroform:ethyl acetate. The purified product **6** was isolated as a pale-yellow oil (5.384 g, 32%).

^{1}H-NMR (500 MHz, $CDCl_3$): δ 6.69 (d, J = 7.8 Hz, 1H), 6.59 (d, J = 1.7 Hz, 1H), 6.55 (dd, J = 7.8, 1.8 Hz, 1H), 5.89 (q, J = 1.5 Hz, 2H), 5.03 (d, J = 8.3 Hz, 1H), 4.47 (dt, J = 8.2, 5.8 Hz, 1H), 4.14 (q, J = 7.2 Hz, 2H), 2.97 (td, J = 13.9, 5.9 Hz, 2H), 1.40 (s, 9H), 1.23 (t, J = 7.1 Hz, 3H).

^{13}C{^{1}H}-NMR (126 MHz, $CDCl_3$): δ 171.9, 155.2, 147.7, 146.7, 129.8, 122.5, 109.8, 108.3, 101.0, 79.9, 61.4, 54.7, 38.1, 28.4, 14.3.

HRMS (ESI) m/z: $[M + H]^+$ for $C_{17}H_{24}NO_6^+$ 338.1598; found 338.1604.

$[\alpha]_D$ = +48.32°.

Synthesis of ethyl (*S*)-2-amino-3-(benzo[*d*][1,3]dioxol-5-yl)propanoate (7)

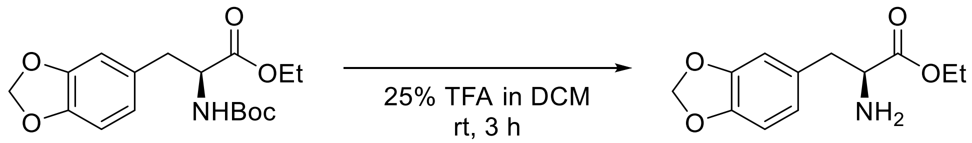

6

7
68%

Compound **6** (8.89 mmol, 3.00 g) was dissolved in 30 mL of dichloromethane and 10 mL of trifluoroacetic acid. The solution was stirred at room temperature for 3 h. Volatile solvents were removed and the residue was dissolved in a 7.24 M K_2CO_3 solution in water. The aqueous layer was extracted 3 times with 10 mL of $CHCl_3$. The organic layers were dried with Na_2SO_4, filtered to remove Na_2SO_4, and evaporated to yield **7** as a yellow-brown oil (1.443g, 68%). The NMR spectra were consistent with previously reported spectra.[5]

^{1}H-NMR (500 MHz, $CDCl_3$): δ 6.74 (d, J = 7.9 Hz, 1H), 6.69 (d, J = 1.7 Hz, 1H), 6.64 (dd, J = 7.9, 1.7 Hz, 1H), 5.93 (d, J = 1.1 Hz, 2H), 4.17 (q, J = 7.1 Hz, 2H), 3.65 (dd, J = 7.7, 5.3 Hz, 1H), 2.99 (dd, J = 13.6, 5.3 Hz, 1H), 2.79 (dd, J = 13.6, 7.8 Hz, 1H), 1.53 (s, 2H), 1.26

(t, $J = 7.1$ Hz, 3H).

$^{13}C\{^1H\}$-NMR (126 MHz, $CDCl_3$): δ 175.0, 147.7, 146.5, 130.9, 122.4, 109.6, 108.3, 101.0, 61.0, 56.0, 40.8, 14.3.

GC-MS: Calculated 237.10, found 237.10.

HRMS (ESI) m/z: $[M + H]^+$ calculated for $C_{12}H_{16}NO_4^+$ 238.1074; found 238.1074.

$[\alpha]_D = +14.64°$.

Synthesis of ethyl (*S*)-3-(benzo[*d*][1,3]dioxol-5-yl)-2-(dimethylamino)propanoate (8)

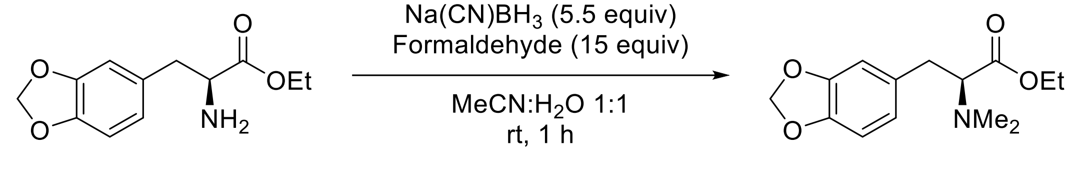

7

8
70%

7 (6.32 mmol, 1.50 g) was dissolved in 50 mL of MeCN. Formaldehyde was added as a 37% solution in H_2O (94.80 mmol, 2.86 g, 7.7 mL). Additional H_2O (42.3 mL) was added and the solution was stirred briefly at room temperature. $Na(CN)BH_3$ (34.17 mmol, 2.19 g) was added in three equal additions, separated by five minutes. The pH was adjusted to 7 with glacial acetic acid and the reaction was stirred for an hour at room temperature. Glacial acetic acid was added as needed to maintain a pH of 7. After the hour was over, volatile solvents were removed. The reaction was quenched with HCl and then basified with aqueous sodium bicarbonate. The aqueous layer was extracted 3 times with ethyl acetate. The organic layers were dried with Na_2SO_4, filtered to remove Na_2SO_4, then volatile solvents were evaporated. Further purification with basic alumina chromatography with 70:29:1 EtOAc:hexane:TEA yielded the pure product **8** as a clear oil (1.167 g, 70%). This procedure was based on a previously reported reaction.[4]

^1H-NMR (500 MHz, $CDCl_3$): δ 6.72 – 6.67 (m, 2H), 6.63 (dd, $J = 7.9$, 1.8 Hz, 1H), 5.90 (d, $J = 0.8$ Hz, 2H), 4.16 – 4.02 (m, 2H), 3.33 (dd, $J = 9.5$, 5.7 Hz, 1H), 2.95 (dd, $J = 13.5$, 9.5 Hz, 1H), 2.83 (dd, $J = 13.5$, 5.7 Hz, 1H), 2.37 (s, 6H), 1.18 (t, $J = 7.2$ Hz, 3H).

$^{13}C\{^1H\}$-NMR (126 MHz, $CDCl_3$): δ 171.5, 147.7, 146.2, 132.0, 122.2, 109.7, 108.3, 101.0, 69.9, 60.3, 42.0, 35.9, 14.6.

GC-MS: Calculated 265.13, found 265.10.

HRMS (ESI) m/z: $[M + H]^+$ for $C_{14}H_{20}NO_4^+$ 266.1389; found 266.1397.

$[\alpha]_D$ = +9.95°.

Synthesis of ethyl (*S*)-3-(benzo[*d*][1,3]dioxol-5-yl)-2-((*tert*-butoxycarbonyl)(methyl)amino) propanoate (9)

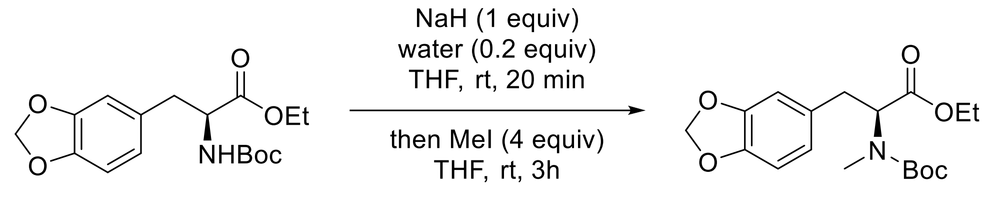

6 **9** 77%

An oven dried found bottom flask and stir bar was charged with NaH (60% dispersion in mineral oil, 12.15 mmol, 0.29 g). The flask was sealed with a septum and purged with N_2 for 1 hour. 20 mL of dry THF was added and the reaction stirred for 5 minutes. Then DI water was added (2.43 mmol, 0.04 mL). Compound **6** (12.15 mmol, 4.10 g) was dissolved in 25 mL of dry THF and the solution was added to the reaction slowly over 10 minutes. The reaction was stirred at room temperature for 20 minutes and then MeI (48.6 mmol, 6.90 g) was added. The reaction was stirred at room temperature for 3 hours. After quenching with 50 mL of water, the reaction was immediately extracted with 3 50 mL portions of ethyl acetate. Organic layers were combined, dried with Na_2SO_4, filtered to remove Na_2SO_4, and then volatile solvents were evaporated to yield the crude product. Further purification with 30/70 EtOAc/hexane yielded **9** as a clear oil (3.300 g, 77%). This procedure was based on a previously reported reaction.[6]

^{1}H-NMR (500 MHz, $CDCl_3$) – mixture of rotomors: δ 6.75 – 6.58 (m, 3H), 5.93 – 5.88 (m, 2H), 4.88 – 4.40 (m, 1H), 4.22 – 4.11 (m, 2H), 3.25 – 3.14 (m, 1H), 2.97 – 2.86 (m, 1H), 2.75 – 2.69 (m, 3H), 1.38 (dd, *J* = 13.9, 2.7 Hz, 9H), 1.26 (dtd, *J* = 13.4, 6.9, 2.6 Hz, 3H).

$^{13}C\{^1H\}$-NMR (126 MHz, $CDCl_3$) – mixture of rotomers: δ 171.5, 171.2, 156.0, 155.2, 147.8, 147.7, 146.4, 131.6, 131.5, 122.2, 122.1, 109.5, 108.5, 108.3, 101.0, 101.0, 80.4, 80.1, 61.8, 61.4, 61.2, 60.2, 35.4, 34.9, 32.6, 32.4, 28.4, 28.4, 14.4.

HRMS (ESI) m/z: $[M + Na]^+$ calculated for $C_{18}H_{25}NNaO_6^+$ 374.1574; found 374.1574.

$[\alpha]_D$ = −12.12°.

Synthesis of ethyl (*S*)-3-(benzo[*d*][1,3]dioxol-5-yl)-2-(methylamino)propanoate (10)

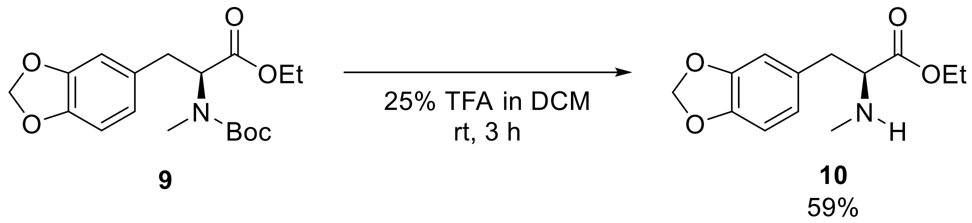

Compound **9** (4.27 mmol, 1.50 g) was dissolved in 30 mL of dichloromethane and 10 mL of trifluoroacetic acid. The solution was stirred at room temperature for 3 h. Volatile solvents were removed and the residue was dissolved in a 7.24 M K_2CO_3 solution in water. The aqueous layer was extracted 3 times with 10 mL of $CHCl_3$. The organic layers were dried with Na_2SO_4, filtered to remove Na_2SO_4, and evaporated to yield **10** as a brown oil (0.635 g, 59%).

^{1}H-NMR (500 MHz, $CDCl_3$) – δ 6.72 (d, J = 7.9 Hz, 1H), 6.68 (d, J = 1.7 Hz, 1H), 6.62 (dd, J = 7.9, 1.7 Hz, 1H), 5.92 (s, 2H), 4.15 (q, J = 7.2 Hz, 2H), 3.37 (t, J = 6.8 Hz, 1H), 2.90 – 2.82 (m, 2H), 2.37 (s, 3H), 1.61 (s, 1H), 1.22 (t, J = 7.2 Hz, 3H).

^{13}C{^{1}H}-NMR (126 MHz, $CDCl_3$) – δ 174.5, 147.8, 146.5, 131.1, 122.4, 109.7, 108.4, 101.1, 65.0, 60.8, 39.4, 34.9, 14.5.

GC-MS: Calculated 251.12, found 251.10.

HRMS (ESI) m/z: $[M + H]^+$ calculated $C_{13}H_{18}NO_4^+$ for 252.1230; found 252.1237.

$[\alpha]_D = -6.14°$.

Synthesis of ethyl (*S*)-2-((*tert*-butoxycarbonyl)amino)-3-(2-(4-methoxyphenyl) benzo[*d*][1,3,2]dioxaborol-5-yl)propanoate (11)

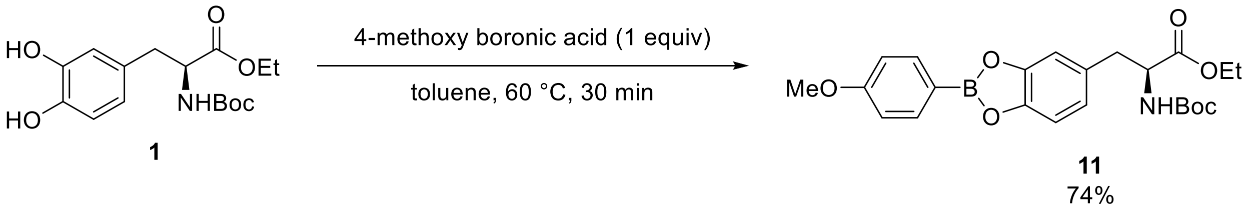

Compound **1** (5 mmol, 1.63 g) and 4-methoxy boronic acid (5 mmol, 0.76 g) were dissolved in 100 mL of toluene in a round bottom flask. The round bottom flask was attached to a rotovap and heated at 60 °C with rotation but no vacuum until all the solids dissolved (about 30 minutes). Then the solvent was removed under an 80 torr vacuum. White solid was recovered and recrystallized from hexane to yield the final product as a flaky white solid (1.677 g). The product showed traces

of 4-methoxy boronic acid in a ratio of 11:1 (product:boronic acid) based on NMR analysis. This corresponds with 1.627 g of **11** and 0.050g of 4-methoxy boronic acid. Thus the product is 97% compound **11** by mass, corresponding with a 74% yield of **11**.

Melting point: 114-117 °C

^{1}H-NMR (500 MHz, $CDCl_3$) – δ 8.00 (d, *J* = 8.6 Hz, 2H), 7.18 (d, *J* = 8.0 Hz, 1H), 7.07 (d, *J* = 1.7 Hz, 1H), 7.00 (d, *J* = 8.7 Hz, 2H), 6.87 (dd, *J* = 8.1, 1.8 Hz, 1H), 5.05 (d, *J* = 8.2 Hz, 1H), 4.74 – 4.42 (m, 1H), 4.19 (d, *J* = 7.2 Hz, 2H), 3.87 (s, 3H), 3.13 (dd, *J* = 15.0, 5.9 Hz, 2H), 1.43 (s, 9H), 1.26 (t, *J* = 7.2 Hz, 3H).

^{13}C{^{1}H}-NMR (126 MHz, $CDCl_3$) – δ 171.9, 155.3, 148.9, 147.9, 137.0, 130.9, 123.8, 114.1, 113.5, 112.2, 80.1, 61.6, 55.3, 38.4, 28.5, 14.3.

^{11}B NMR (160 MHz, $CDCl_3$) δ 31.89.

HRMS: The boronic ester protecting group seemed to fall off during HRMS analysis using ESI + mode. The only ion observed was at 348.1420, which corresponds to the $[M + Na]^+$ adduct of the deprotected catechol (calculated for $C_{16}H_{23}NaNO_6^+$ 348.1418).

$[\alpha]_D$ = +55.85°.

Synthesis of ethyl (*S*)-2-((*tert*-butoxycarbonyl)amino)-3-(7-(4,4,5,5-tetramethyl-1,3,2-dioxaborolan-2-yl)benzo[*d*][1,3]dioxol-5-yl)propanoate (6a)

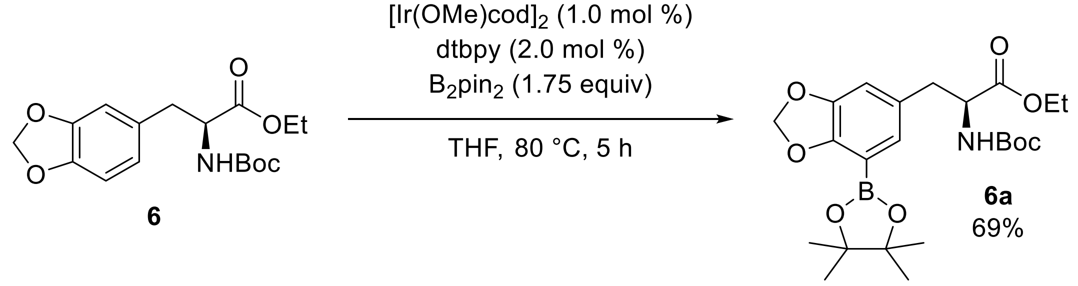

6 **6a** 69%

In a glovebox under N_2 atmosphere, a 5 mL conical vial was charged with 0.5 mL of THF with $[Ir(OMe)cod]_2$ (0.005 mmol, 3.31 mg) and B_2pin_2 (0.875 mmol, 222.2 mg). The solution was stirred briefly and then 0.5 mL of THF with 4,4′-di-*tert*-butyl-2,2′-bipyridine (dtbpy, 0.01 mmol, 2.68 mg) was added. The solution was stirred for 5 minutes, after which **6** (0.5 mmol, 168.7 mg) was added. The conical vial was capped with a Teflon-coated cap and stirred briefly before being removed from the glovebox. The conical vial was placed in a preheated aluminum block at 80 °C and stirred for 5 h. Volatile solvents were removed and ^{1}H-NMR analysis of the crude reaction

mixture showed 100% conversion. The product was purified via silica gel flash column chromatography with 60:20:20 hexane:chloroform:ethyl acetate to yield **6a** as a clear oil (0.159 g, 69%).

^{1}H-NMR (500 MHz, $CDCl_3$): δ 6.95 (s, 1H), 6.68 (s, 1H), 5.99 (s, 2H), 5.01 (d, J = 8.2 Hz, 1H), 4.59 – 4.36 (m, 1H), 4.17 (q, J = 7.2 Hz, 2H), 3.00 (dd, J = 13.8, 5.8 Hz, 1H), 1.42 (s, 9H), 1.34 (s, 12H), 1.26 (t, J = 7.2 Hz, 3H).

^{13}C{^{1}H}-NMR (126 MHz, $CDCl_3$): δ 171.95, 155.2, 152.0, 147.4, 129.1, 128.8, 112.3, 101.3, 84.0, 79.9, 61.6, 54.8, 37.9, 28.5, 25.0, 25.0, 14.3.

^{11}B NMR (160 MHz, $CDCl_3$): δ 29.62.

HRMS (ESI) m/z: $[M + Na]^+$ calculated for $C_{23}H_{34}BNNaO_8^+$ 486.2770, found 486.2274.

$[\alpha]_D = -15.06°$.

Synthesis of (*S*)-1-ethoxy-1-oxo-3-(7-(4,4,5,5-tetramethyl-1,3,2-dioxaborolan-2-yl) benzo[*d*][1,3]dioxol-5-yl)propan-2-aminium trichloroacetate (7a)

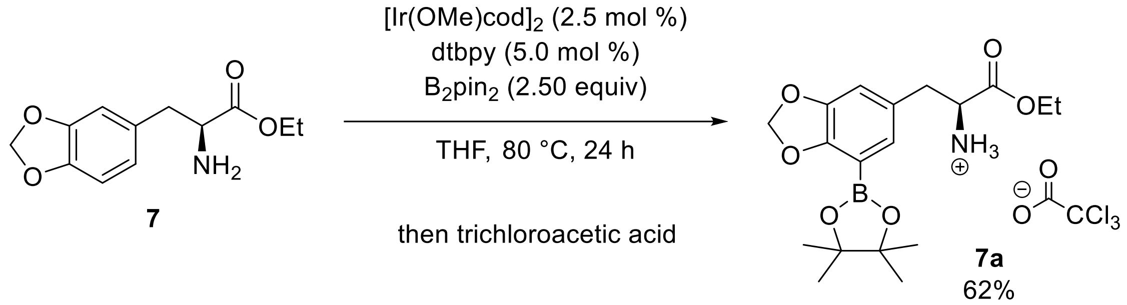

In a glovebox under N_2 atmosphere, a 5 mL conical vial was charged with 0.5 mL of THF with $[Ir(OMe)cod]_2$ (0.0125 mmol, 8.28 mg) and B_2pin_2 (1.250 mmol, 317.0 mg). The solution was stirred briefly and then 0.5 mL of THF with 4,4′-di-*tert*-butyl-2,2′-bipyridine (dtbpy, 0.025 mmol, 6.7 mg) was added. The solution was stirred for 5 minutes, after which **7** (0.5 mmol, 118.6 mg) was added. The conical vial was capped with a Teflon-coated cap and stirred briefly before being removed from the glovebox. The conical vial was placed in a preheated aluminum block at 80 °C and stirred for 24 h. Volatile solvents were removed and ^{1}H-NMR analysis of the crude reaction mixture showed 80% conversion to **7a**. The residue was dissolved in a 1:1 mixture of ethyl acetate and hexane. Trichloroacetic acid was added until a precipitate was observed. The solution was placed in freezer for 24 h and then the precipitate formed was filtered off to yield **7a** as a tan solid

(112.0 mg, 62%).

Melting point: 142-143 °C.

^{1}H-NMR (500 MHz, $CDCl_3$): δ 8.30 (s, 3H), 7.03 (d, J = 1.8 Hz, 1H), 6.90 (d, J = 1.8 Hz, 1H), 5.99 (dd, J = 6.0, 1.4 Hz, 2H), 4.29 – 4.13 (m, 3H), 3.25 (d, J = 6.8 Hz, 2H), 1.33 (s, 12H), 1.25 (t, 3H).

$^{13}C\{^1H\}$-NMR (126 MHz, $CDCl_3$): δ 169.1, 165.6, 152.7, 147.8, 128.9, 126.5, 112.5, 101.6, 94.8, 84.2, 83.4, 63.1, 54.8, 36.1, 25.0, 24.7, 14.1.

^{11}B NMR (160 MHz, $CDCl_3$): δ 22.00.

HRMS (ESI) m/z: $[M + H]^+$ calculated for $C_{18}H_{27}BNO_6^+$ 364.1926; found 364.1934.

$[\alpha]_D = -17.79°$.

Synthesis of ethyl (*S*)-2-(dimethylamino)-3-(7-(4,4,5,5-tetramethyl-1,3,2-dioxaborolan-2-yl) benzo[*d*][1,3]dioxol-5-yl)propanoatetrichloroacetate (8a)

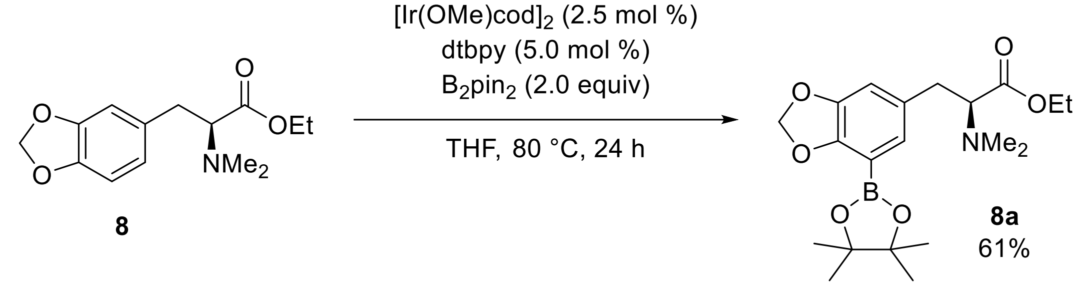

In a glovebox under N_2 atmosphere, a 5 mL conical vial was charged with 0.5 mL of THF with $[Ir(OMe)cod]_2$ (0.0125 mmol, 8.28 mg) and B_2pin_2 (1.00 mmol, 253.9 mg). The solution was stirred briefly and then 0.5 mL of THF with 4,4′-di-*tert*-butyl-2,2′-bipyridine (dtbpy, 0.025 mmol, 6.7 mg) was added. The solution was stirred for 5 minutes, after which **8** (0.5 mmol, 132.7 mg) was added. The conical vial was capped with a Teflon-coated cap and stirred briefly before being removed from the glovebox. The conical vial was placed in a preheated aluminum block at 80 °C for 24 h. Volatile solvents were removed and the product was purified via silica gel flash column chromatography with 70:30 hexane:EtOAc with 1% added triethyl amine to yield **8a** as a clear oil (0.120 g, 61%).

^{1}H-NMR (500 MHz, $CDCl_3$): δ 7.00 (d, J = 1.8 Hz, 1H), 6.74 (d, J = 1.8 Hz, 1H), 5.96 (s, 2H), 4.08 (q, J = 7.1 Hz, 2H), 3.32 (dd, J = 9.6, 5.9 Hz, 1H), 2.93 (dd, J = 13.5, 9.5 Hz, 1H), 2.82 (dd, J = 13.5, 5.8 Hz, 1H), 2.36 (s, 6H), 1.32 (d, J = 2.4 Hz, 12H), 1.20–1.15 (m, 3H).

$^{13}C\{^{1}H\}$-NMR (126 MHz, $CDCl_3$): δ 171.5, 151.5, 147.2, 131.2, 128.2, 112.3, 101.2, 84.0, 69.9, 60.2, 42.0, 35.6, 25.0, 24.9, 14.5.

^{11}B NMR (160 MHz, $CDCl_3$): δ 30.04.

HRMS (ESI) m/z: $[M + H]^+$ calculated for $C_{20}H_{31}BNO_6^+$ 392.2239; found 392.2242.

$[\alpha]_D = -4.37°$.

Synthesis of ethyl (*S*)-2-((*tert*-butoxycarbonyl)(methyl)amino)-3-(7-(4,4,5,5-tetramethyl-1,3,2-dioxaborolan-2-yl)benzo[*d*][1,3]dioxol-5-yl)propanoate (9a)

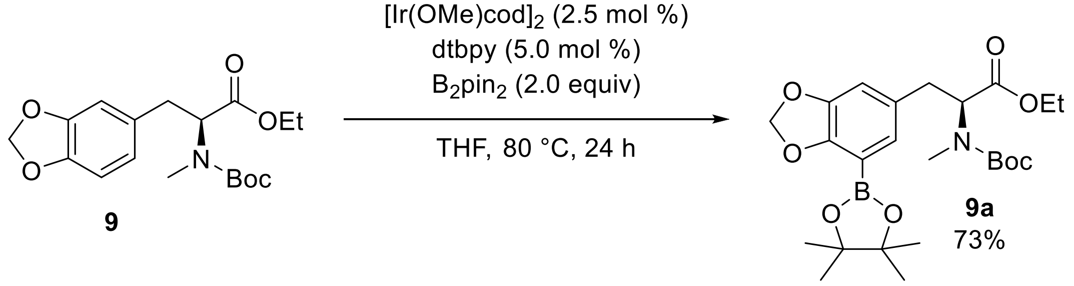

In a glovebox under N_2 atmosphere, a 5 mL conical vial was charged with 0.5 mL of THF with $[Ir(OMe)cod]_2$ (0.0125 mmol, 8.28 mg) and B_2pin_2 (1.00 mmol, 253.9 mg). The solution was stirred briefly and then 0.5 mL of THF with 4,4′-di-*tert*-butyl-2,2′-bipyridine (dtbpy, 0.025 mmol, 6.7 mg) was added. The solution was stirred for 5 minutes, after which **9** (0.5 mmol, 175.7 mg) was added. The conical vial was capped with a Teflon-coated cap and stirred briefly before being removed from the glovebox. The conical vial was placed in a preheated aluminum block at 80 °C and stirred for 24 h. Volatile solvents were removed and the product was purified via silica gel flash column chromatography with 20:80 EtOAc:Hexane to yield **9a** as a clear oil (0.274 g, 73%).

^{1}H-NMR (500 MHz, $CDCl_3$) mixture of rotomers: δ 7.01 (d, J = 7.3 Hz, 1H), 6.71 (d, J = 25.9 Hz, 1H), 5.96 (s, 2H), 4.76 – 4.43 (m, 1H), 4.17 (d, J = 7.1 Hz, 2H), 3.18 (m, 1H), 2.97 (m, 1H), 2.71 (d, J = 12.0 Hz, 3H), 1.44 – 1.30 (m, 21H), 1.25 (dd, J = 15.1, 7.6 Hz, 3H).

$^{13}C\{^{1}H\}$-NMR (126 MHz, $CDCl_3$) mixture of rotomers: δ 171.4, 171.2, 155.8, 155.2, 151.6, 147.3, 130.8, 130.7, 128.2, 128.0, 112.1, 112.0, 101.2, 101.2, 84.0, 84.0, 80.3, 78.0, 61.4, 61.3, 61.1, 60.4, 35.1, 34.6, 32.7, 32.2, 28.4, 28.3, 25.0, 24.9, 14.3.

^{11}B NMR (160 MHz, $CDCl_3$): δ 30.01.

HRMS (ESI) m/z: $[M + Na]^+$ calculated for $C_{24}H_{36}BNNaO_8^+$ 500.2426; found 500.2430.

$[\alpha]_D = -10.08°$.

Synthesis of ethyl (*S*)-2-(methylamino)-3-(7-(4,4,5,5-tetramethyl-1,3,2-dioxaborolan-2-yl) benzo[*d*][1,3]dioxol-5-yl)propanoate (10a)

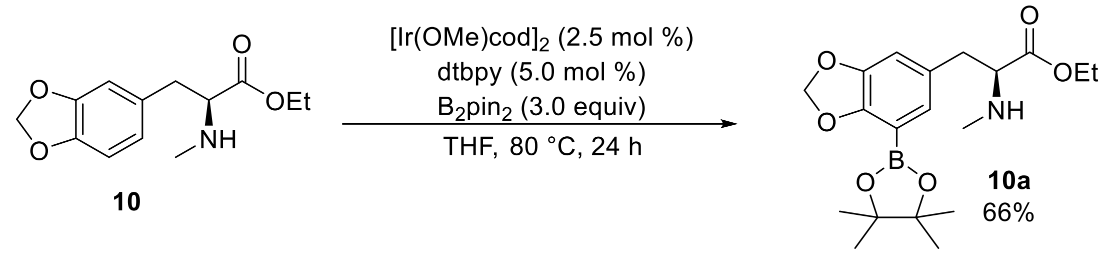

In a glovebox under N_2 atmosphere, a 5 mL conical vial was charged with 0.5 mL of THF with $[Ir(OMe)cod]_2$ (0.0125 mmol, 8.28 mg) and B_2pin_2 (1.50 mmol, 380 mg). The solution was stirred briefly and then 0.5 mL of THF with 4,4′-di-*tert*-butyl-2,2′-bipyridine (dtbpy, 0.025 mmol, 6.7 mg) was added. The solution was stirred for 5 minutes, after which **10** (0.5 mmol, 125.6 mg) was added. The conical vial was capped with a Teflon-coated cap and stirred briefly before being removed from the glovebox. The conical vial was placed in a preheated aluminum block at 80 °C and stirred for 24 h. Volatile solvents were removed and the product was purified via silica gel flash column chromatography with 20:80 EtOAc:Hexane to yield **10a** as a clear oil (0.124 g, 66%).

^{1}H-NMR (500 MHz, $CDCl_3$): δ 6.99 (s, 1H), 6.74 (d, *J* = 1.8 Hz, 1H), 5.98 (s, 2H), 4.14 (dd, *J* = 7.2, 4.7 Hz, 2H), 3.36 (t, *J* = 6.8 Hz, 1H), 2.85 (dd, *J* = 6.9, 4.2 Hz, 2H), 2.35 (s, 3H), 1.33 (s, 12H), 1.21 (t, *J* = 6.7 Hz, 3H).

^{13}C{^{1}H}-NMR (126 MHz, $CDCl_3$): δ 174.5, 151.7, 147.3, 130.4, 128.4, 112.2, 101.3, 84.0, 65.0, 60.8, 39.2, 34.9, 25.0, 25.0, 14.4.

^{11}B NMR (160 MHz, $CDCl_3$): δ 30.19.

HRMS (ESI) m/z : $[M + H]^+$ calculated for $C_{19}H_{29}BNO_6^+$ 378.2082; found 378.2088.

$[\alpha]_D$ = −5.69°.

Synthesis of ethyl (*S*)-2-((*tert*-butoxycarbonyl)amino)-3-(3,4-dihydroxy-5-(4,4,5,5-tetramethyl-1,3,2-dioxaborolan-2-yl)phenyl)propanoate (12)

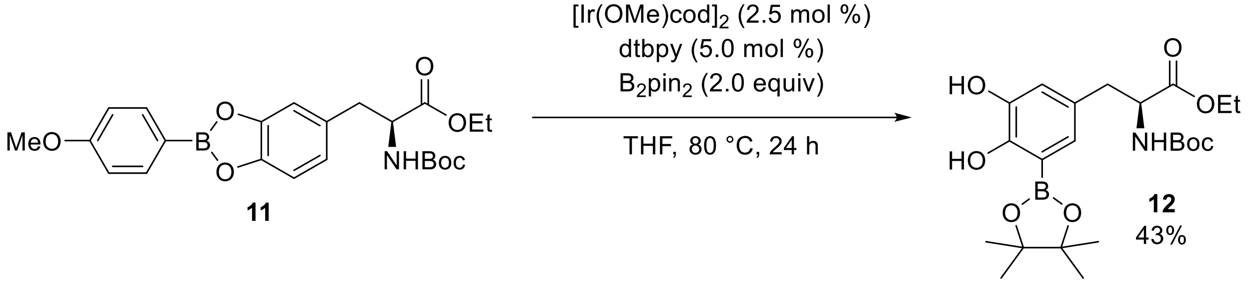

In a glovebox under N_2 atmosphere, a 5 mL conical vial was charged with 1 mL of THF with [Ir(OMe)cod]$_2$ (0.025 mmol, 16.6 mg) and B_2pin_2 (2.00 mmol, 508 mg). The solution was stirred briefly and then 1 mL of THF with 4,4′-di-*tert*-butyl-2,2′-bipyridine (dtbpy, 0.050 mmol, 12.4 mg) was added. The solution was stirred for 5 minutes, after which **11** (1.0 mmol, 442 mg) was added. The conical vial was capped with a Teflon-coated cap and stirred briefly before being removed from the glovebox. The conical vial was placed in a preheated aluminum block at 80 °C and stirred for 24 h. Volatile solvents were removed and the product was purified via silica gel flash column chromatography with 40:60 EtOAc:Hexane to yield **12** as a clear oil (0.093 g, 43%).

^{1}H-NMR (500 MHz, $CDCl_3$): δ 7.78 (s, 2H), 6.89 (d, J = 2.2 Hz, 1H), 6.79 (d, J = 2.1 Hz, 1H), 5.60 (d, J = 2.7 Hz, 1H), 5.00 (d, J = 8.2 Hz, 1H), 4.46 (d, J = 8.2 Hz, 1H), 4.23 – 4.09 (m, 2H), 2.96 (dd, J = 19.4, 5.9 Hz, 2H), 1.42 (s, 9H), 1.34 (s, 12H), 1.25 (t, J = 7.1 Hz, 3H).

^{13}C{^{1}H}-NMR (126 MHz, $CDCl_3$): δ 172.1, 155.3, 149.4, 144.1, 128.2, 127.0, 119.6, 84.7, 79.9, 61.5, 54.8, 37.6, 28.4, 25.0, 24.9, 14.3.

^{11}B NMR (160 MHz, $CDCl_3$): δ 30.67.

HRMS (ESI) m/z: [M + Na]$^+$ calculated for $C_{22}H_{34}BNNaO_8^+$ 474.2270; found 474.2271.

$[\alpha]_D$ = +26.94°.

Synthesis of ethyl (*S*)-3-(benzo[*d*][1,3]dioxol-5-yl-7-d)-2-((tert-butoxycarbonyl)amino) propanoate (13)

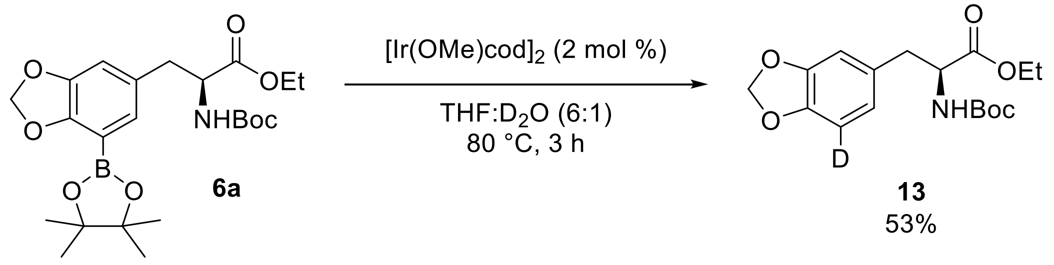

In a glovebox filled with N_2 atmosphere, a 5 mL conical vial was charged with **6a** (0.302 mmol, 140.0 mg) and $[Ir(OMe)cod]_2$ (0.006 mmol, 4.01 mg). 4.5 mL of dry THF was added. The conical vial was removed from the glovebox and briefly uncapped to add 0.75 mL of D_2O. The capped conical vial was placed in a preheated aluminum block at 80 °C and stirred for 3 h. Volatile solvents were removed and the remaining liquid was extracted 3 times with DCM. Organic layers were dried and volatile solvents were removed. The crude product was purified via silica gel chromatography with 60:20:20 hexane:chloroform:ethyl acetate to yield a clear oil (54 mg, 53%). ^{1}H-NMR analysis showed 95% deuteration at the indicated position. This procedure was based on a previously reported reaction.[7]

^{1}H-NMR (500 MHz, $CDCl_3$): δ 6.60 (s, 1H), 6.57 (s, 1H), 5.91 (d, J = 2.0 Hz, 2H), 5.01 (d, J = 8.3 Hz, 1H), 4.49 (dd, J = 8.2, 5.8 Hz, 1H), 4.16 (q, J = 7.2 Hz, 2H), 2.99 (dd, J = 11.5, 5.9 Hz, 2H), 1.41 (s, 9H), 1.24 (t, J = 7.1 Hz, 3H).

^{13}C{^{1}H}-NMR (126 MHz, $CDCl_3$): δ 171.9, 155.2, 147.8, 146.7, 129.8, 122.5, 109.8, 108.4, 108.2, 101.1, 80.0, 61.5, 54.7, 38.1, 28.4, 14.3.

HRMS (ESI) m/z: $[M + Na]^+$ calculated for $C_{17}H_{22}DNNaO_6^+$ 361.1480; found 361.1479.

$[\alpha]_D$ = +18.65°.

Synthesis of (*S*)-1-ethoxy-1-oxo-3-(7-(4,4,5,5-tetramethyl-1,3,2-dioxaborolan-2-yl) benzo[*d*][1,3]dioxol-5-yl)propan-2-aminium trifluoroacetate (14)

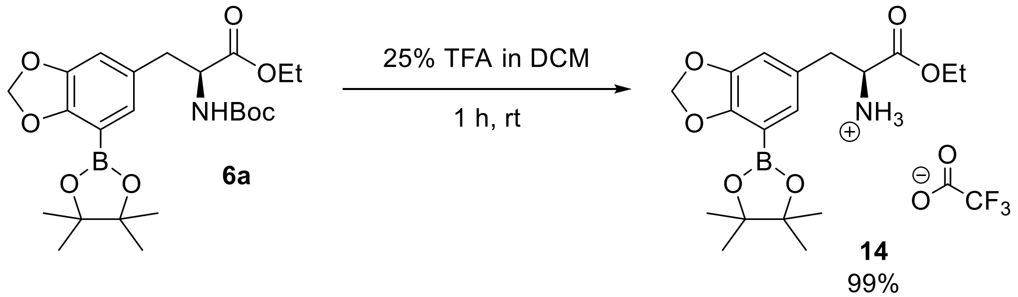

Compound **6a** (0.285 mmol, 132 mg) was dissolved in 4.5 mL of dichloromethane. 1.5 mL of trifluoroacetic acid was added and the reaction stirred at room temperature for 1 hour. The solvent was evaporated under reduced pressure to yield the product **14** as a brown oil (139 mg, 99%). No further purification was necessary.

^{1}H-NMR (500 MHz, $CDCl_3$): δ 8.22 (bs, 3H), 6.99 (s, 1H), 6.79 (s, 1H), 5.96 (d, J = 8.4 Hz, 2H),

4.25 – 4.12 (m, 3H), 3.22 – 3.12 (m, 2H), 1.31 (s, 12H), 1.23 (t, J = 7.2 Hz, 3H).

$^{13}C\{^1H\}$-NMR (126 MHz, $CDCl_3$): δ 169.0, 152.7, 147.8, 128.8, 126.2, 112.1, 101.6, 84.2, 63.1, 54.6, 35.9, 24.8, 13.9.

^{11}B-NMR (126 MHz, $CDCl_3$): δ 30.57.

^{19}F-NMR (470 MHz, $CDCl_3$): δ −75.87.

HRMS (ESI) m/z: $[M + H]^+$ calculated for ammonium cation $C_{18}H_{27}BNO_6^+$ 364.1926; found 364.1932.

$[\alpha]_D = -0.52°$.

Synthesis of ethyl (*S*)-2-((*tert*-butoxycarbonyl)amino)-3-(7-hydroxybenzo[*d*][1,3]dioxol-5-yl) propanoate (15)

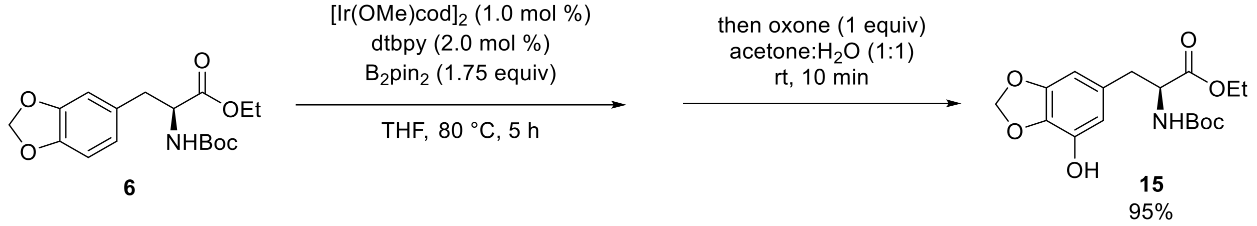

In a glovebox under N_2 atmosphere, a 5 mL conical vial was charged with 1 mL of THF with $[Ir(OMe)cod]_2$ (0.01 mmol, 6.6 mg) and B_2pin_2 (1.75 mmol, 444 mg). The solution was stirred briefly and then 1 mL of THF with 4,4′-di-*tert*-butyl-2,2′-bipyridine (dtbpy, 0.02 mmol, 5.3 mg) was added. The solution was stirred for 5 minutes, after which **6** (1.0 mmol, 337 mg) was added. The conical vial was capped with a Teflon-coated cap and stirred briefly before being removed from the glovebox. The conical vial was placed in a preheated aluminum block at 80 °C and stirred for 5 h. Volatile solvents were removed by a gentle stream of nitrogen. The residue was redissolved in 3 mL of acetone. Oxone (1.0 mmol, 615 mg) in 3.0 mL of water was added dropwise with vigorous stirring. After addition of oxone was complete, the reaction was stirred for a further 10 minutes. $NaHSO_3$ (3 g in 10 mL water) was added to quench the reaction and the resulting mixture was immediately extracted with 3 portions of 10 mL dichloromethane. The organic layers were combined, dried over Na_2SO_4, filtered to remove the Na_2SO_4, and evaporated to yield the crude product. The product was further purified by silica gel column chromatography with 20:20:60 EtOAc:chloroform:hexane to yield **15** as a clear oil (0.345 g, 95%). This procedure was based on a previously reported reaction.[8]

^{1}H-NMR (500 MHz, $CDCl_3$): δ 6.81 (bs, 1H), 6.28 – 6.24 (m, 1H), 6.20 (d, J = 1.6 Hz, 1H), 5.86 (dd, J = 7.7, 1.5 Hz, 2H), 5.18 (d, J = 8.4 Hz, 1H), 4.57 – 4.45 (m, 1H), 4.23 – 4.11 (m, 2H), 2.92 (t, J = 5.9 Hz, 2H), 1.41 (s, 9H), 1.23 (t, J = 7.1 Hz, 3H).

^{13}C{^{1}H}-NMR (126 MHz, $CDCl_3$): δ 172.2, 155.5, 148.8, 139.8, 133.2, 130.1, 112.1, 102.3, 101.2, 80.3, 61.6, 54.6, 28.3, 14.1.

HRMS (ESI) m/z: $[M + Na]^+$ calculated for $C_{17}H_{23}NNaO_7^+$ 376.1367; found 376.1362.

$[\alpha]_D$ = +83.14°.

Synthesis of ethyl (*S*)-4-(6-(2-((*tert*-butoxycarbonyl)amino)-3-ethoxy-3-oxopropyl) benzo[*d*][1,3]dioxol-4-yl)benzoate (16)

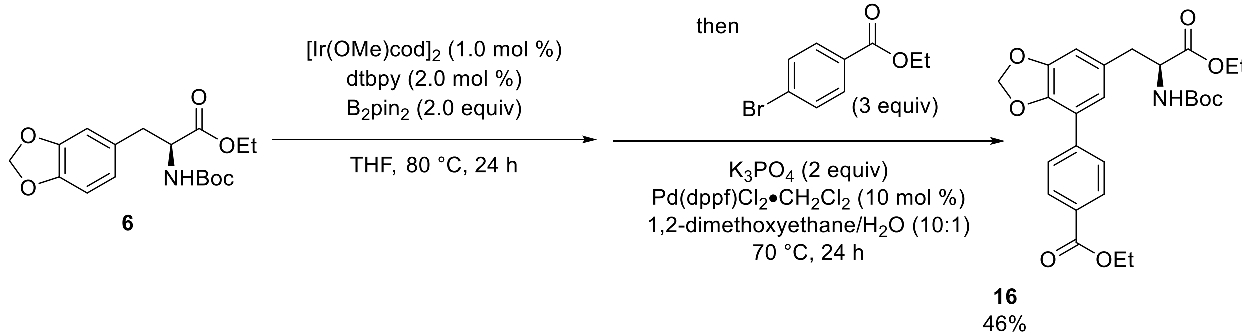

In a glovebox under N_2 atmosphere, a 5 mL conical vial was charged with 1 mL of THF with $[Ir(OMe)cod]_2$ (0.01 mmol, 6.6 mg) and B_2pin_2 (2.0 mmol, 508 mg). The solution was stirred briefly and then 1 mL of THF with 4,4′-di-*tert*-butyl-2,2′-bipyridine (dtbpy, 0.02 mmol, 5.3 mg) was added. The solution was stirred for 5 minutes, after which **6** (1.0 mmol, 337 mg) was added. The conical vial was capped with a Teflon-coated cap and stirred briefly before being removed from the glovebox. The conical vial was placed in a preheated aluminum block at 80 °C and stirred for 24 h to ensure complete conversion to the borylated amino acid derivative. The reaction was cooled to room temperature and filtered through a short silica plug. The plug was washed with ethyl acetate. Organic layers were combined and evaporated to yield the crude borylated amino acid derivative. The residue was redissolved in 8 mL of 1,2-dimethoxyethane in an oven dried round bottom flask. Ethyl 4-bromobenzoate (3 mmol, 687 mg), K_3PO_4 (2 mmol, 513 mg), $Pd(dppf)Cl_2 \cdot CH_2Cl_2$ (0.1 mmol, 82 mg), and 0.8 mL of water were added. The round bottom flask was attached to a condenser and sealed with a septum. The entire apparatus was purged with N_2 for 10 minutes before being heated at 70 °C in an oil bath for 24 hours. After cooling, 60 mL of water were added and the mixture was extracted 3 times with 60 mL portions of ethyl acetate. The

organic layers were combined, dried over Na_2SO_4, filtered to remove the Na_2SO_4, and evaporated to yield the crude product. The product was further purified by silica gel column chromatography with 20:20:60 EtOAc:chloroform:hexane to yield **16** as a brown oil (0.227 g, 46%). This procedure was based on a previously reported reaction.[9]

^{1}H-NMR (500 MHz, $CDCl_3$): δ 8.07 (d, *J* = 8.4 Hz, 2H), 7.74 (d, *J* = 8.5 Hz, 2H), 6.82 (d, *J* = 1.6 Hz, 1H), 6.63 (d, *J* = 1.6 Hz, 1H), 6.02 – 5.98 (m, 2H), 5.10 (d, *J* = 8.2 Hz, 1H), 4.54 (d, *J* = 7.8 Hz, 1H), 4.38 (q, *J* = 7.1 Hz, 2H), 4.17 (q, *J* = 7.1 Hz, 2H), 3.10 (dd, *J* = 13.9, 5.8 Hz, 1H), 3.02 (dd, *J* = 14.0, 6.0 Hz, 1H), 1.44 – 1.36 (m, 12H), 1.23 (t, *J* = 7.1 Hz, 3H).

^{13}C{^{1}H}-NMR (126 MHz, $CDCl_3$): δ 171.8, 166.5, 155.2, 148.4, 144.2, 140.2, 130.4, 129.9, 129.5, 127.7, 122.1, 121.4, 109.5, 101.3, 80.0, 61.6, 61.1, 54.6, 38.2, 28.4, 14.5, 14.3.

HRMS (ESI) m/z: $[M + Na]^+$ calculated for $C_{26}H_{31}NNaO_8^+$ 508.1942; found 508.1936.

$[\alpha]_D$ = +28.31°.

References

(1) Fulmer, G. R.; Miller, A. J. M.; Sherden, N. H.; Gottlieb, H. E.; Nudelman, A.; Stoltz, B. M.; Bercaw, J. E.; Goldberg, K. I. NMR Chemical Shifts of Trace Impurities: Common Laboratory Solvents, Organics, and Gases in Deuterated Solvents Relevant to the Organometallic Chemist. *Organometallics* **2010**, *29* (9), 2176–2179. https://doi.org/10.1021/om100106e.

(2) Stenhagen, I. S. R.; Kirjavainen, A. K.; Forsback, S. J.; Jørgensen, C. G.; Robins, E. G.; Luthra, S. K.; Solin, O.; Gouverneur, V. [18F]Fluorination of an Arylboronic Ester Using [18F]Selectfluor Bis(Triflate): Application to 6-[18F]Fluoro-l-DOPA. *Chemical Communications* **2013**, *49* (14), 1386–1388. https://doi.org/10.1039/c2cc38646a.

(3) Dickner, T.; Laschat, S. Pictet-Spengler Cyclization vs. Aminal Formation: Competing Reaction Pathways of Benzo[b][1,7]Naphthyridines Controlled by the Configuration. *Helv Chim Acta* **2001**, *84* (7), 2064–2070. https://doi.org/10.1002/1522-2675(20010711)84:7<2064::AID-HLCA2064>3.0.CO;2-Y.

(4) Samper Barceló, V.; Bienz, S. Synthesis of Highly Substituted 3-Pyrrolin-2-Ones from N,N-Disubstituted α-Amino Acids. *Journal of Organic Chemistry* **2018**, *83* (5), 2734–2743. https://doi.org/10.1021/acs.joc.7b03187.

(5) Sayyed, I. A.; Sudalai, A. Asymmetric Synthesis of L-DOPA and (R)-Selegiline via, OsO 4-Catalyzed Asymmetric Dihydroxylation. *Tetrahedron Asymmetry* **2004**, *15* (19), 3111–3116. https://doi.org/10.1016/j.tetasy.2004.08.007.

(6) Prashad, M.; Har, D.; Hu, B.; Kim, H. Y.; Repic, O.; Blacklock, T. J. An Efficient and Practical N-Methylation of Amino Acid Derivatives. *Org Lett* **2003**, *5* (2), 125–128. https://doi.org/10.1021/ol0268440.

(7) Kallepalli, V. A.; Gore, K. A.; Shi, F.; Sanchez, L.; Chotana, G. A.; Miller, S. L.; Maleczka, R. E.; Smith, M. R. Harnessing C-H Borylation/Deborylation for Selective Deuteration, Synthesis of Boronate Esters, and Late Stage Functionalization. *Journal of Organic Chemistry* **2015**, *80* (16), 8341–8353. https://doi.org/10.1021/acs.joc.5b01588.

(8) Shi, F.; Smith, M. R.; Maleczka, R. E. Aromatic Borylation/Amidation/Oxidation: A Rapid Route to 5-Substituted 3-Amidophenols. *Org Lett* **2006**, *8* (7), 1411–1414. https://doi.org/10.1021/ol060207i.

(9) Robinson, H.; Stillibrand, J.; Simelis, K.; MacDonald, S. J. F.; Nortcliffe, A. Iridium-Catalysed C-H Borylation of β-Aryl-Aminopropionic Acids. *Org Biomol Chem* **2020**, *18* (34), 6696–6701. https://doi.org/10.1039/d0ob01495h.

NMR Spectra

^{1}H-NMR of ethyl (*S*)-2-((*tert*-butoxycarbonyl)amino)-3-(3,4-dihydroxyphenyl)propanoate (**1**) – (500 MHz, $CDCl_3$) 32
^{13}C{^{1}H}-NMR of ethyl (*S*)-2-((*tert*-butoxycarbonyl)amino)-3-(3,4-dihydroxyphenyl)propanoate (**1**) – (126 MHz, $CDCl_3$) 33
^{1}H-NMR of ethyl (*S*)-2-((*tert*-butoxycarbonyl)amino)-3-(3,4-dimethoxyphenyl)propanoate (**2**) – (500 MHz, $CDCl_3$) 34
^{13}C{^{1}H}-NMR of ethyl (*S*)-2-((*tert*-butoxycarbonyl)amino)-3-(3,4-dimethoxyphenyl)propanoate (**2**) – (126 MHz, $CDCl_3$) 35
^{1}H-NMR of ethyl (*S*)-2-((*tert*-butoxycarbonyl)amino)-3-(3,4-diethoxyphenyl)propanoate (**3**) – (500 MHz, $CDCl_3$) 36
^{13}C{^{1}H}-NMR of ethyl (*S*)-2-((*tert*-butoxycarbonyl)amino)-3-(3,4-diethoxyphenyl)propanoate (**3**) – (126 MHz, $CDCl_3$) 37
^{1}H-NMR of ethyl (*S*)-2-amino-3-(3,4-dimethoxyphenyl)propanoate (**4**) – (500 MHz, $CDCl_3$) 38
^{13}C{^{1}H}-NMR of ethyl (*S*)-2-amino-3-(3,4-dimethoxyphenyl)propanoate (**4**) – (126 MHz, $CDCl_3$) 39
^{1}H-NMR of ethyl (*S*)-3-(3,4-dimethoxyphenyl)-2-(dimethylamino)propanoate (**5**) – (500 MHz, $CDCl_3$)..................................... 40
^{13}C{^{1}H}-NMR of ethyl (*S*)-3-(3,4-dimethoxyphenyl)-2-(dimethylamino)propanoate (**5**) – (126MHz, $CDCl_3$) 41
HSQC of ethyl (*S*)-3-(3,4-dimethoxyphenyl)-2-(dimethylamino)propanoate (**5**) – ($CDCl_3$)... 42
HMBC of ethyl (*S*)-3-(3,4-dimethoxyphenyl)-2-(dimethylamino)propanoate (**5**) – ($CDCl_3$)... 43
^{1}H-NMR of ethyl (*S*)-3-(benzo[*d*][1,3]dioxol-5-yl)-2-((*tert*-butoxycarbonyl)amino) propanoate (**6**) – (500 MHz, $CDCl_3$)........... 44
^{13}C{^{1}H}-NMR of ethyl (*S*)-3-(benzo[*d*][1,3]dioxol-5-yl)-2-((*tert*-butoxycarbonyl)amino) propanoate (**6**) – (126 MHz, $CDCl_3$) . 45
HSQC of ethyl (*S*)-3-(benzo[*d*][1,3]dioxol-5-yl)-2-((*tert*-butoxycarbonyl)amino) propanoate (**6**) – ($CDCl_3$)............................... 46
HSQC of ethyl (*S*)-3-(benzo[*d*][1,3]dioxol-5-yl)-2-((*tert*-butoxycarbonyl)amino) propanoate (**6**) – ($CDCl_3$) – 5.5 to 7.5 ppm..... 47
HMBC of ethyl (*S*)-3-(benzo[*d*][1,3]dioxol-5-yl)-2-((*tert*-butoxycarbonyl)amino) propanoate (**6**) – ($CDCl_3$).............................. 48
HMBC of ethyl (*S*)-3-(benzo[*d*][1,3]dioxol-5-yl)-2-((*tert*-butoxycarbonyl)amino) propanoate (**6**) – ($CDCl_3$) – 5.5 to 7.5 ppm.... 49
^{1}H-NMR of ethyl (*S*)-2-amino-3-(benzo[*d*][1,3]dioxol-5-yl)propanoate (**7**) – (500 MHz, $CDCl_3$)... 50
^{13}C{^{1}H}-NMR of ethyl (*S*)-2-amino-3-(benzo[*d*][1,3]dioxol-5-yl)propanoate (**7**) – (126 MHz, $CDCl_3$).. 51
HSQC of ethyl (*S*)-2-amino-3-(benzo[*d*][1,3]dioxol-5-yl)propanoate (**7**) – ($CDCl_3$) 52
HSQC of ethyl (*S*)-2-amino-3-(benzo[*d*][1,3]dioxol-5-yl)propanoate (**7**) – ($CDCl_3$) – 5.5 to 7.5 ppm 53

HMBC of ethyl (*S*)-2-amino-3-(benzo[*d*][1,3]dioxol-5-yl)propanoate (**7**) – ($CDCl_3$) 54
HMBC of ethyl (*S*)-2-amino-3-(benzo[*d*][1,3]dioxol-5-yl)propanoate (**7**) – ($CDCl_3$) – 5.5 to 7.5 ppm 55
^{1}H-NMR of ethyl (*S*)-3-(benzo[*d*][1,3]dioxol-5-yl)-2-(dimethylamino)propanoate (**8**) – (500 MHz, $CDCl_3$) 56
^{13}C{^{1}H}-NMR of ethyl (*S*)-3-(benzo[*d*][1,3]dioxol-5-yl)-2-(dimethylamino)propanoate (**8**) – (126 MHz, $CDCl_3$) 57
HSQC of ethyl (*S*)-3-(benzo[*d*][1,3]dioxol-5-yl)-2-(dimethylamino)propanoate (**8**) – ($CDCl_3$) 58
HSQC of ethyl (*S*)-3-(benzo[*d*][1,3]dioxol-5-yl)-2-(dimethylamino)propanoate (**8**) – ($CDCl_3$) – 5.5 to 7.5 ppm 59
HMBC of ethyl (*S*)-3-(benzo[*d*][1,3]dioxol-5-yl)-2-(dimethylamino)propanoate (**8**) – ($CDCl_3$) 60
HMBC of ethyl (*S*)-3-(benzo[*d*][1,3]dioxol-5-yl)-2-(dimethylamino)propanoate (**8**) – ($CDCl_3$) – 5.5 to 7.5 ppm 61
^{1}H-NMR of ethyl (*S*)-3-(benzo[*d*][1,3]dioxol-5-yl)-2-((*tert*-butoxycarbonyl)(methyl)amino) propanoate (**9**) – (500 MHz, $CDCl_3$) 62
^{13}C{^{1}H}-NMR of ethyl (*S*)-3-(benzo[*d*][1,3]dioxol-5-yl)-2-((*tert*-butoxycarbonyl)(methyl)amino) propanoate (**9**) – (126 MHz, $CDCl_3$) 63
HSQC of ethyl (*S*)-3-(benzo[*d*][1,3]dioxol-5-yl)-2-((*tert*-butoxycarbonyl)(methyl)amino) propanoate (**9**) – ($CDCl_3$) 64
HSQC of ethyl (*S*)-3-(benzo[*d*][1,3]dioxol-5-yl)-2-((*tert*-butoxycarbonyl)(methyl)amino) propanoate (**9**) – ($CDCl_3$) – 5.5 to 7.5 ppm 65
HMBC of ethyl (*S*)-3-(benzo[*d*][1,3]dioxol-5-yl)-2-((*tert*-butoxycarbonyl)(methyl)amino) propanoate (**9**) – ($CDCl_3$) 66
HMBC of ethyl (*S*)-3-(benzo[*d*][1,3]dioxol-5-yl)-2-((*tert*-butoxycarbonyl)(methyl)amino) propanoate (**9**) – ($CDCl_3$) – 5.5 to 7.5 ppm 67
Variable Temperature 1H-NMR of ethyl (*S*)-3-(benzo[*d*][1,3]dioxol-5-yl)-2-((*tert*-butoxycarbonyl)(methyl)amino) propanoate (**9**) – (126 MHz, Benzene-*d*6) 68
^{1}H-NMR of ethyl (*S*)-3-(benzo[*d*][1,3]dioxol-5-yl)-2-(methylamino)propanoate (**10**) – (500 MHz, $CDCl_3$) 69
^{13}C{^{1}H}-NMR of ethyl (*S*)-3-(benzo[*d*][1,3]dioxol-5-yl)-2-(methylamino)propanoate (**10**) – (126 MHz, $CDCl_3$) 70
HSQC of ethyl (*S*)-3-(benzo[*d*][1,3]dioxol-5-yl)-2-(methylamino)propanoate (**10**) – ($CDCl_3$) 71
HSQC of ethyl (*S*)-3-(benzo[*d*][1,3]dioxol-5-yl)-2-(methylamino)propanoate (**10**) – ($CDCl_3$) – 5.5 to 7.5 ppm 72

HMBC of ethyl (*S*)-3-(benzo[*d*][1,3]dioxol-5-yl)-2-(methylamino)propanoate (**10**) – ($CDCl_3$) 73
HMBC of ethyl (*S*)-3-(benzo[*d*][1,3]dioxol-5-yl)-2-(methylamino)propanoate (**10**) – ($CDCl_3$) – 5.5 to 7.5 ppm 74
^{1}H-NMR of ethyl (*S*)-2-((*tert*-butoxycarbonyl)amino)-3-(2-(4-methoxyphenyl) benzo[*d*][1,3,2]dioxaborol-5-yl)propanoate (**11**) – (500 MHz, $CDCl_3$) 75
^{13}C{^{1}H}-NMR of ethyl (*S*)-2-((*tert*-butoxycarbonyl)amino)-3-(2-(4-methoxyphenyl) benzo[*d*][1,3,2]dioxaborol-5-yl)propanoate (**11**) – (126 MHz, $CDCl_3$) 76
^{11}B-NMR of ethyl (*S*)-2-((*tert*-butoxycarbonyl)amino)-3-(2-(4-methoxyphenyl) benzo[*d*][1,3,2]dioxaborol-5-yl)propanoate (**11**) – (160 MHz, $CDCl_3$) 77
HSQC of ethyl (*S*)-2-((*tert*-butoxycarbonyl)amino)-3-(2-(4-methoxyphenyl) benzo[*d*][1,3,2]dioxaborol-5-yl)propanoate (**11**) – ($CDCl_3$) 78
HSQC of ethyl (*S*)-2-((*tert*-butoxycarbonyl)amino)-3-(2-(4-methoxyphenyl) benzo[*d*][1,3,2]dioxaborol-5-yl)propanoate (**11**) – ($CDCl_3$) – 6.5 to 8.5 ppm 79
HMBC of ethyl (*S*)-2-((*tert*-butoxycarbonyl)amino)-3-(2-(4-methoxyphenyl) benzo[*d*][1,3,2]dioxaborol-5-yl)propanoate (**11**) – ($CDCl_3$) 80
HMBC of ethyl (*S*)-2-((*tert*-butoxycarbonyl)amino)-3-(2-(4-methoxyphenyl) benzo[*d*][1,3,2]dioxaborol-5-yl)propanoate (**11**) – ($CDCl_3$) – 6.5 to 8.5 ppm 81
^{1}H-NMR of ethyl (*S*)-2-((*tert*-butoxycarbonyl)amino)-3-(7-(4,4,5,5-tetramethyl-1,3,2-dioxaborolan-2-yl)benzo[*d*][1,3]dioxol-5-yl)propanoate (**6a**) – (500 MHz, $CDCl_3$) 82
^{13}C{^{1}H}-NMR of ethyl (*S*)-2-((*tert*-butoxycarbonyl)amino)-3-(7-(4,4,5,5-tetramethyl-1,3,2-dioxaborolan-2-yl)benzo[*d*][1,3]dioxol-5-yl)propanoate (**6a**) – (126 MHz, $CDCl_3$) 83
^{11}B-NMR of ethyl (*S*)-2-((*tert*-butoxycarbonyl)amino)-3-(7-(4,4,5,5-tetramethyl-1,3,2-dioxaborolan-2-yl)benzo[*d*][1,3]dioxol-5-yl)propanoate (**6a**) – (160 MHz, $CDCl_3$) 84
HSQC of ethyl (*S*)-2-((*tert*-butoxycarbonyl)amino)-3-(7-(4,4,5,5-tetramethyl-1,3,2-dioxaborolan-2-yl)benzo[*d*][1,3]dioxol-5-yl)propanoate (**6a**) – ($CDCl_3$) 85
HSQC of ethyl (*S*)-2-((*tert*-butoxycarbonyl)amino)-3-(7-(4,4,5,5-tetramethyl-1,3,2-dioxaborolan-2-yl)benzo[*d*][1,3]dioxol-5-yl)propanoate (**6a**) – ($CDCl_3$) – 5.5 to 7.5 ppm 86

HMBC of ethyl (*S*)-2-((*tert*-butoxycarbonyl)amino)-3-(7-(4,4,5,5-tetramethyl-1,3,2-dioxaborolan-2-yl)benzo[*d*][1,3]dioxol-5-yl)propanoate (**6a**) – ($CDCl_3$) 87

HMBC of ethyl (*S*)-2-((*tert*-butoxycarbonyl)amino)-3-(7-(4,4,5,5-tetramethyl-1,3,2-dioxaborolan-2-yl)benzo[*d*][1,3]dioxol-5-yl)propanoate (**6a**) – ($CDCl_3$) – 5.5 to 7.5 ppm 88

^{1}H-NMR of (*S*)-1-ethoxy-1-oxo-3-(7-(4,4,5,5-tetramethyl-1,3,2-dioxaborolan-2-yl)benzo[*d*][1,3]dioxol-5-yl)propan-2-aminium trichloroacetate (**7a**) – (500 MHz, $CDCl_3$) 89

^{13}C{^{1}H}-NMR of (*S*)-1-ethoxy-1-oxo-3-(7-(4,4,5,5-tetramethyl-1,3,2-dioxaborolan-2-yl)benzo[*d*][1,3]dioxol-5-yl)propan-2-aminium trichloroacetate (**7a**) – (126 MHz, $CDCl_3$) 90

^{11}B-NMR of (*S*)-1-ethoxy-1-oxo-3-(7-(4,4,5,5-tetramethyl-1,3,2-dioxaborolan-2-yl)benzo[*d*][1,3]dioxol-5-yl)propan-2-aminium trichloroacetate (**7a**) – (160 MHz, $CDCl_3$) 91

HSQC of (*S*)-1-ethoxy-1-oxo-3-(7-(4,4,5,5-tetramethyl-1,3,2-dioxaborolan-2-yl)benzo[*d*][1,3]dioxol-5-yl)propan-2-aminium trichloroacetate (**7a**) – ($CDCl_3$).......... 92

HSQC of (*S*)-1-ethoxy-1-oxo-3-(7-(4,4,5,5-tetramethyl-1,3,2-dioxaborolan-2-yl)benzo[*d*][1,3]dioxol-5-yl)propan-2-aminium trichloroacetate (**7a**) – ($CDCl_3$) – 5.5 to 8.5 ppm 93

HMBC of (*S*)-1-ethoxy-1-oxo-3-(7-(4,4,5,5-tetramethyl-1,3,2-dioxaborolan-2-yl)benzo[*d*][1,3]dioxol-5-yl)propan-2-aminium trichloroacetate (**7a**) – ($CDCl_3$).......... 94

HMBC of (*S*)-1-ethoxy-1-oxo-3-(7-(4,4,5,5-tetramethyl-1,3,2-dioxaborolan-2-yl)benzo[*d*][1,3]dioxol-5-yl)propan-2-aminium trichloroacetate (**7a**) – ($CDCl_3$) – 5.5 to 8.5 ppm 95

^{1}H-NMR of ethyl (*S*)-2-(dimethylamino)-3-(7-(4,4,5,5-tetramethyl-1,3,2-dioxaborolan-2-yl)benzo[*d*][1,3]dioxol-5-yl) propanoatetrichloroacetate (**8a**) – (500 MHz, $CDCl_3$).......... 96

^{13}C{^{1}H}-NMR of ethyl (*S*)-2-(dimethylamino)-3-(7-(4,4,5,5-tetramethyl-1,3,2-dioxaborolan-2-yl)benzo[*d*][1,3]dioxol-5-yl) propanoatetrichloroacetate (**8a**) – (126 MHz, $CDCl_3$).......... 97

^{11}B-NMR of ethyl (*S*)-2-(dimethylamino)-3-(7-(4,4,5,5-tetramethyl-1,3,2-dioxaborolan-2-yl)benzo[*d*][1,3]dioxol-5-yl) propanoatetrichloroacetate (**8a**) – (160 MHz, $CDCl_3$).......... 98

HSQC of ethyl (*S*)-2-(dimethylamino)-3-(7-(4,4,5,5-tetramethyl-1,3,2-dioxaborolan-2-yl)benzo[*d*][1,3]dioxol-5-yl) propanoatetrichloroacetate (**8a**) – ($CDCl_3$).......... 99

HSQC of ethyl (*S*)-2-(dimethylamino)-3-(7-(4,4,5,5-tetramethyl-1,3,2-dioxaborolan-2-yl)benzo[*d*][1,3]dioxol-5-yl)

propanoatetrichloroacetate (**8a**) – ($CDCl_3$) – 5.5 to 7.5 ppm 100

HMBC of ethyl (*S*)-2-(dimethylamino)-3-(7-(4,4,5,5-tetramethyl-1,3,2-dioxaborolan-2-yl)benzo[*d*][1,3]dioxol-5-yl) propanoatetrichloroacetate (**8a**) – ($CDCl_3$) 101

HMBC of ethyl (*S*)-2-(dimethylamino)-3-(7-(4,4,5,5-tetramethyl-1,3,2-dioxaborolan-2-yl)benzo[*d*][1,3]dioxol-5-yl) propanoatetrichloroacetate (**8a**) – ($CDCl_3$) – 5.5 to 7.5 ppm 102

^{1}H-NMR of ethyl (*S*)-2-((*tert*-butoxycarbonyl)(methyl)amino)-3-(7-(4,4,5,5-tetramethyl-1,3,2-dioxaborolan-2-yl) benzo[*d*][1,3]dioxol-5-yl)propanoate (**9a**) – (500 MHz, $CDCl_3$) 103

^{13}C{^{1}H}-NMR of ethyl (*S*)-2-((*tert*-butoxycarbonyl)(methyl)amino)-3-(7-(4,4,5,5-tetramethyl-1,3,2-dioxaborolan-2-yl) benzo[*d*][1,3]dioxol-5-yl)propanoate (**9a**) – (126 MHz, $CDCl_3$) 104

^{11}B-NMR of ethyl (*S*)-2-((*tert*-butoxycarbonyl)(methyl)amino)-3-(7-(4,4,5,5-tetramethyl-1,3,2-dioxaborolan-2-yl) benzo[*d*][1,3]dioxol-5-yl)propanoate (**9a**) – (160 MHz, $CDCl_3$) 105

HSQC of ethyl (*S*)-2-((*tert*-butoxycarbonyl)(methyl)amino)-3-(7-(4,4,5,5-tetramethyl-1,3,2-dioxaborolan-2-yl) benzo[*d*][1,3]dioxol-5-yl)propanoate (**9a**) – ($CDCl_3$) 106

HSQC of ethyl (*S*)-2-((*tert*-butoxycarbonyl)(methyl)amino)-3-(7-(4,4,5,5-tetramethyl-1,3,2-dioxaborolan-2-yl) benzo[*d*][1,3]dioxol-5-yl)propanoate (**9a**) – ($CDCl_3$) – 5.5 to 7.5 ppm 107

HMBC of ethyl (*S*)-2-((*tert*-butoxycarbonyl)(methyl)amino)-3-(7-(4,4,5,5-tetramethyl-1,3,2-dioxaborolan-2-yl) benzo[*d*][1,3]dioxol-5-yl)propanoate (**9a**) – ($CDCl_3$) 108

HMBC of ethyl (*S*)-2-((*tert*-butoxycarbonyl)(methyl)amino)-3-(7-(4,4,5,5-tetramethyl-1,3,2-dioxaborolan-2-yl) benzo[*d*][1,3]dioxol-5-yl)propanoate (**9a**) – ($CDCl_3$) – 5.5 to 7.5 ppm 109

^{1}H-NMR of ethyl (*S*)-2-(methylamino)-3-(7-(4,4,5,5-tetramethyl-1,3,2-dioxaborolan-2-yl)benzo[*d*][1,3]dioxol-5-yl)propanoate (**10a**) – (500 MHz, $CDCl_3$) 110

^{13}C{^{1}H}-NMR of ethyl (*S*)-2-(methylamino)-3-(7-(4,4,5,5-tetramethyl-1,3,2-dioxaborolan-2-yl)benzo[*d*][1,3]dioxol-5-yl)propanoate (**10a**) – (126 MHz, $CDCl_3$) 111

^{11}B-NMR of ethyl (*S*)-2-(methylamino)-3-(7-(4,4,5,5-tetramethyl-1,3,2-dioxaborolan-2-yl)benzo[*d*][1,3]dioxol-5-yl)propanoate (**10a**) – (160 MHz, $CDCl_3$) 112

HSQC of ethyl (*S*)-2-(methylamino)-3-(7-(4,4,5,5-tetramethyl-1,3,2-dioxaborolan-2-yl)benzo[*d*][1,3]dioxol-5-yl)propanoate (**10a**) – ($CDCl_3$) 113

HSQC of ethyl (*S*)-2-(methylamino)-3-(7-(4,4,5,5-tetramethyl-1,3,2-dioxaborolan-2-yl)benzo[*d*][1,3]dioxol-5-yl)propanoate (**10a**) – ($CDCl_3$) – 5.5 to 7.5 ppm 114

HMBC of ethyl (*S*)-2-(methylamino)-3-(7-(4,4,5,5-tetramethyl-1,3,2-dioxaborolan-2-yl)benzo[*d*][1,3]dioxol-5-yl)propanoate (**10a**) – ($CDCl_3$) 115

HMBC of ethyl (*S*)-2-(methylamino)-3-(7-(4,4,5,5-tetramethyl-1,3,2-dioxaborolan-2-yl)benzo[*d*][1,3]dioxol-5-yl)propanoate (**10a**) – ($CDCl_3$) – 5.5 to 7.5 ppm 116

^{1}H-NMR of ethyl (*S*)-2-((*tert*-butoxycarbonyl)amino)-3-(3,4-dihydroxy-5-(4,4,5,5-tetramethyl-1,3,2-dioxaborolan-2-yl) phenyl)propanoate (**12**) – (500 MHz, $CDCl_3$) 117

^{13}C{^{1}H}-NMR of ethyl (*S*)-2-((*tert*-butoxycarbonyl)amino)-3-(3,4-dihydroxy-5-(4,4,5,5-tetramethyl-1,3,2-dioxaborolan-2-yl) phenyl)propanoate (**12**) – (126 MHz, $CDCl_3$) 118

^{11}B-NMR of ethyl (*S*)-2-((*tert*-butoxycarbonyl)amino)-3-(3,4-dihydroxy-5-(4,4,5,5-tetramethyl-1,3,2-dioxaborolan-2-yl) phenyl)propanoate (**12**) – (160 MHz, $CDCl_3$) 119

HSQC of ethyl (*S*)-2-((*tert*-butoxycarbonyl)amino)-3-(3,4-dihydroxy-5-(4,4,5,5-tetramethyl-1,3,2-dioxaborolan-2-yl) phenyl)propanoate (**12**) – ($CDCl_3$) 120

HSQC of ethyl (*S*)-2-((*tert*-butoxycarbonyl)amino)-3-(3,4-dihydroxy-5-(4,4,5,5-tetramethyl-1,3,2-dioxaborolan-2-yl) phenyl)propanoate (**12**) – ($CDCl_3$) – 5.5 to 7.5 ppm 121

HMBC of ethyl (*S*)-2-((*tert*-butoxycarbonyl)amino)-3-(3,4-dihydroxy-5-(4,4,5,5-tetramethyl-1,3,2-dioxaborolan-2-yl) phenyl)propanoate (**12**) – ($CDCl_3$) 122

HMBC of ethyl (*S*)-2-((*tert*-butoxycarbonyl)amino)-3-(3,4-dihydroxy-5-(4,4,5,5-tetramethyl-1,3,2-dioxaborolan-2-yl) phenyl)propanoate (**12**) – ($CDCl_3$) – 5.5 to 7.5 ppm 123

^{1}H-NMR of ethyl (*S*)-3-(benzo[*d*][1,3]dioxol-5-yl-7-d)-2-((tert-butoxycarbonyl)amino) propanoate (**13**) – (500 MHz, $CDCl_3$) 124

^{1}H-NMR of ethyl (*S*)-3-(benzo[*d*][1,3]dioxol-5-yl-7-d)-2-((tert-butoxycarbonyl)amino) propanoate (**13**) – (500 MHz, $CDCl_3$) – 5.5 to 7.5 ppm 125

^{13}C{^{1}H}-NMR of ethyl (*S*)-3-(benzo[*d*][1,3]dioxol-5-yl-7-d)-2-((tert-butoxycarbonyl)amino) propanoate (**13**) – (126 MHz, $CDCl_3$) 126

^{1}H-NMR of (*S*)-1-ethoxy-1-oxo-3-(7-(4,4,5,5-tetramethyl-1,3,2-dioxaborolan-2-yl) benzo[*d*][1,3]dioxol-5-yl)propan-2-aminium trifluoroacetate (**14**) – (500 MHz, $CDCl_3$) 127

$^{13}C\{^{1}H\}$-NMR of (*S*)-1-ethoxy-1-oxo-3-(7-(4,4,5,5-tetramethyl-1,3,2-dioxaborolan-2-yl) benzo[*d*][1,3]dioxol-5-yl)propan-2-aminium trifluoroacetate (**14**) – (126 MHz, $CDCl_3$) 128

^{11}B-NMR of (*S*)-1-ethoxy-1-oxo-3-(7-(4,4,5,5-tetramethyl-1,3,2-dioxaborolan-2-yl) benzo[*d*][1,3]dioxol-5-yl)propan-2-aminium trifluoroacetate (**14**) – (160 MHz, $CDCl_3$) 129

^{19}F-NMR of (*S*)-1-ethoxy-1-oxo-3-(7-(4,4,5,5-tetramethyl-1,3,2-dioxaborolan-2-yl) benzo[*d*][1,3]dioxol-5-yl)propan-2-aminium trifluoroacetate (**14**) – (470 MHz, $CDCl_3$) 130

^{1}H-NMR of ethyl (*S*)-2-((*tert*-butoxycarbonyl)amino)-3-(7-hydroxybenzo[*d*][1,3]dioxol-5-yl)propanoate (**15**) – (500 MHz, $CDCl_3$) 131

^{13}C-NMR of ethyl (*S*)-2-((*tert*-butoxycarbonyl)amino)-3-(7-hydroxybenzo[*d*][1,3]dioxol-5-yl)propanoate (**15**) – (126 MHz, $CDCl_3$) 132

HSQC of ethyl (*S*)-2-((*tert*-butoxycarbonyl)amino)-3-(7-hydroxybenzo[*d*][1,3]dioxol-5-yl)propanoate (**15**) – ($CDCl_3$) 133

HSQC of ethyl (*S*)-2-((*tert*-butoxycarbonyl)amino)-3-(7-hydroxybenzo[*d*][1,3]dioxol-5-yl)propanoate (**15**) – ($CDCl_3$) – 5.5 to 7.5 ppm 134

HMBC of ethyl (*S*)-2-((*tert*-butoxycarbonyl)amino)-3-(7-hydroxybenzo[*d*][1,3]dioxol-5-yl)propanoate (**15**) – ($CDCl_3$) 135

HMBC of ethyl (*S*)-2-((*tert*-butoxycarbonyl)amino)-3-(7-hydroxybenzo[*d*][1,3]dioxol-5-yl)propanoate (**15**) – ($CDCl_3$) – 5.5 to 7.5 ppm 136

^{1}H-NMR of ethyl (*S*)-4-(6-(2-((*tert*-butoxycarbonyl)amino)-3-ethoxy-3-oxopropyl) benzo[*d*][1,3]dioxol-4-yl)benzoate (**16**) – (500 MHz, $CDCl_3$) 137

$^{13}C\{^{1}H\}$-NMR of ethyl (*S*)-4-(6-(2-((*tert*-butoxycarbonyl)amino)-3-ethoxy-3-oxopropyl) benzo[*d*][1,3]dioxol-4-yl)benzoate (**16**) – (126 MHz, $CDCl_3$) 138

HSQC of ethyl (*S*)-4-(6-(2-((*tert*-butoxycarbonyl)amino)-3-ethoxy-3-oxopropyl) benzo[*d*][1,3]dioxol-4-yl)benzoate (**16**) – ($CDCl_3$) 139

HSQC of ethyl (*S*)-4-(6-(2-((*tert*-butoxycarbonyl)amino)-3-ethoxy-3-oxopropyl) benzo[*d*][1,3]dioxol-4-yl)benzoate (**16**) – ($CDCl_3$) – 5.5 to 8.5 ppm 140

HMBC of ethyl (*S*)-4-(6-(2-((*tert*-butoxycarbonyl)amino)-3-ethoxy-3-oxopropyl) benzo[*d*][1,3]dioxol-4-yl)benzoate (**16**) – ($CDCl_3$) 141

HMBC of ethyl (*S*)-4-(6-(2-((*tert*-butoxycarbonyl)amino)-3-ethoxy-3-oxopropyl) benzo[*d*][1,3]dioxol-4-yl)benzoate (**16**) – ($CDCl_3$) – 5.5 to 8.5 ppm 142

^{1}H-NMR of ethyl (*S*)-2-((*tert*-butoxycarbonyl)amino)-3-(3,4-dihydroxyphenyl)propanoate (1) – (500 MHz, $CDCl_3$)

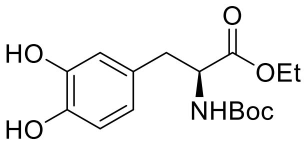

1

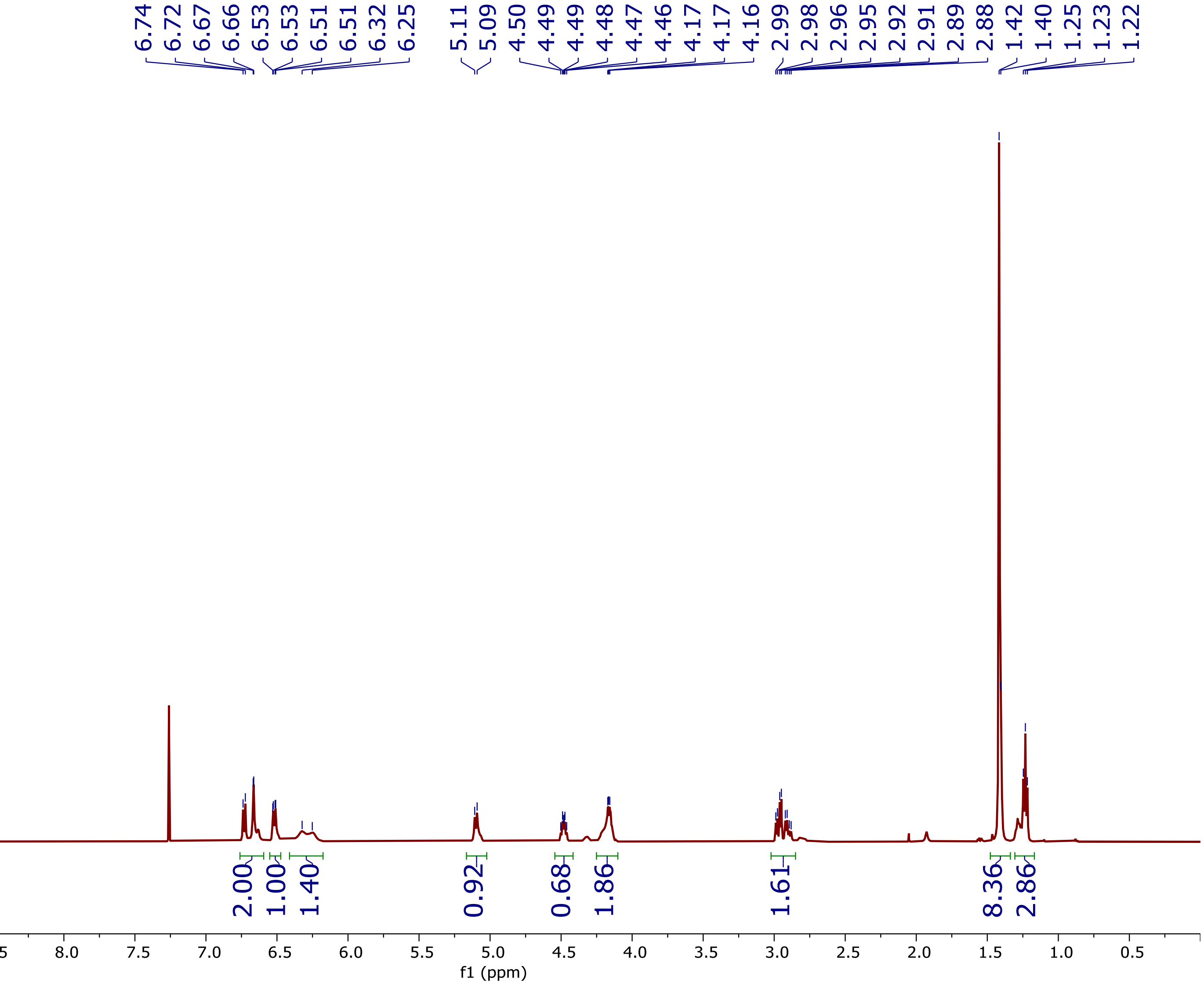

$^{13}C\{^{1}H\}$-NMR of ethyl (*S*)-2-((*tert*-butoxycarbonyl)amino)-3-(3,4-dihydroxyphenyl)propanoate (1) – (126 MHz, $CDCl_3$)

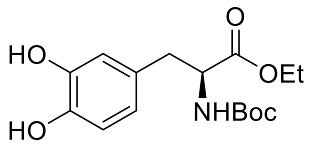

1

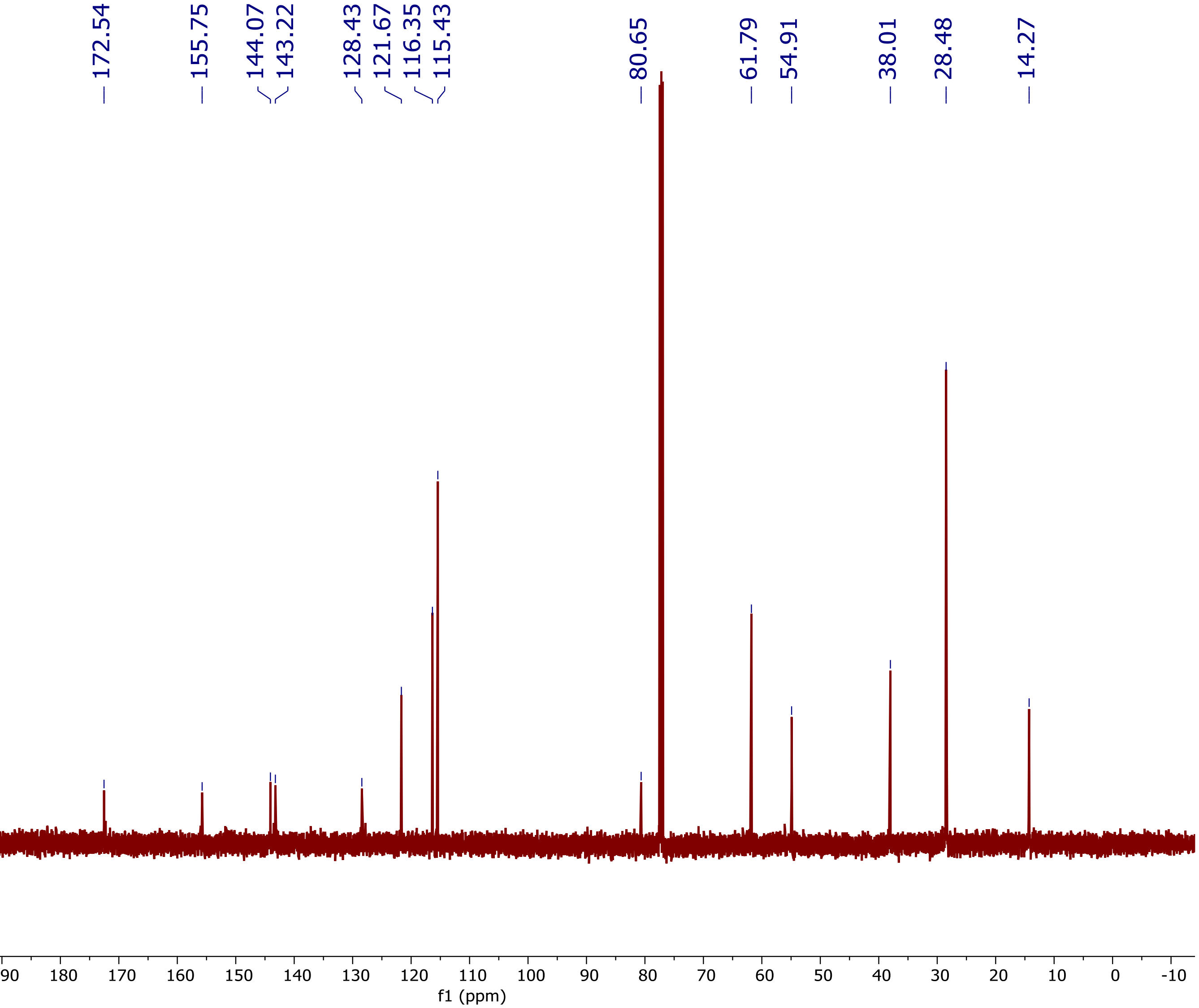

^{1}H-NMR of ethyl (*S*)-2-((*tert*-butoxycarbonyl)amino)-3-(3,4-dimethoxyphenyl)propanoate (2) – (500 MHz, $CDCl_3$)

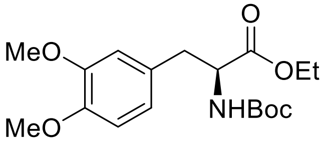

2

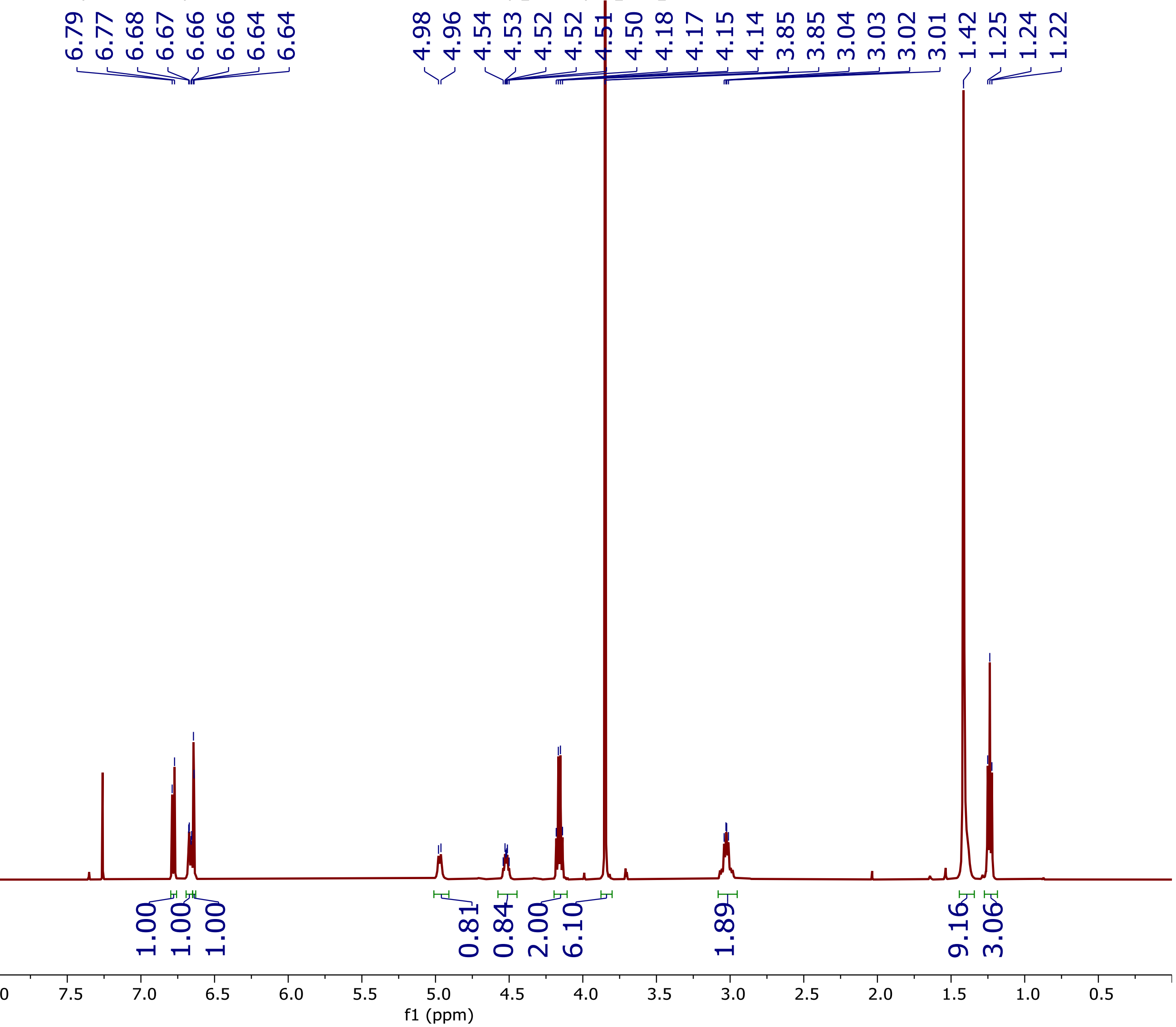

$^{13}C\{^{1}H\}$-NMR of ethyl (*S*)-2-((*tert*-butoxycarbonyl)amino)-3-(3,4-dimethoxyphenyl)propanoate (2) – (126 MHz, $CDCl_3$)

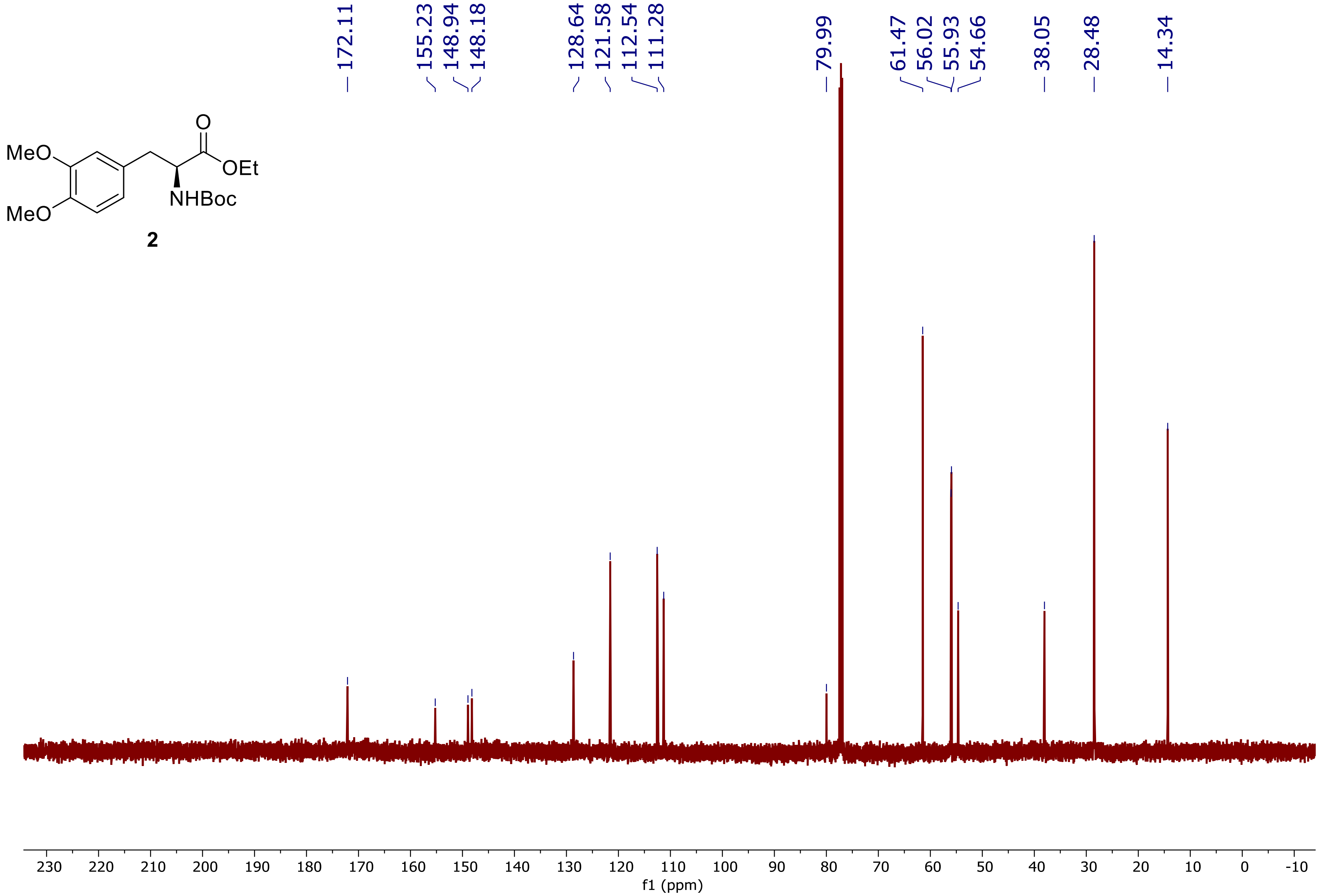

^{1}H-NMR of ethyl (*S*)-2-((*tert*-butoxycarbonyl)amino)-3-(3,4-diethoxyphenyl)propanoate (3) – (500 MHz, $CDCl_3$)

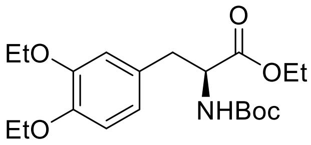

3

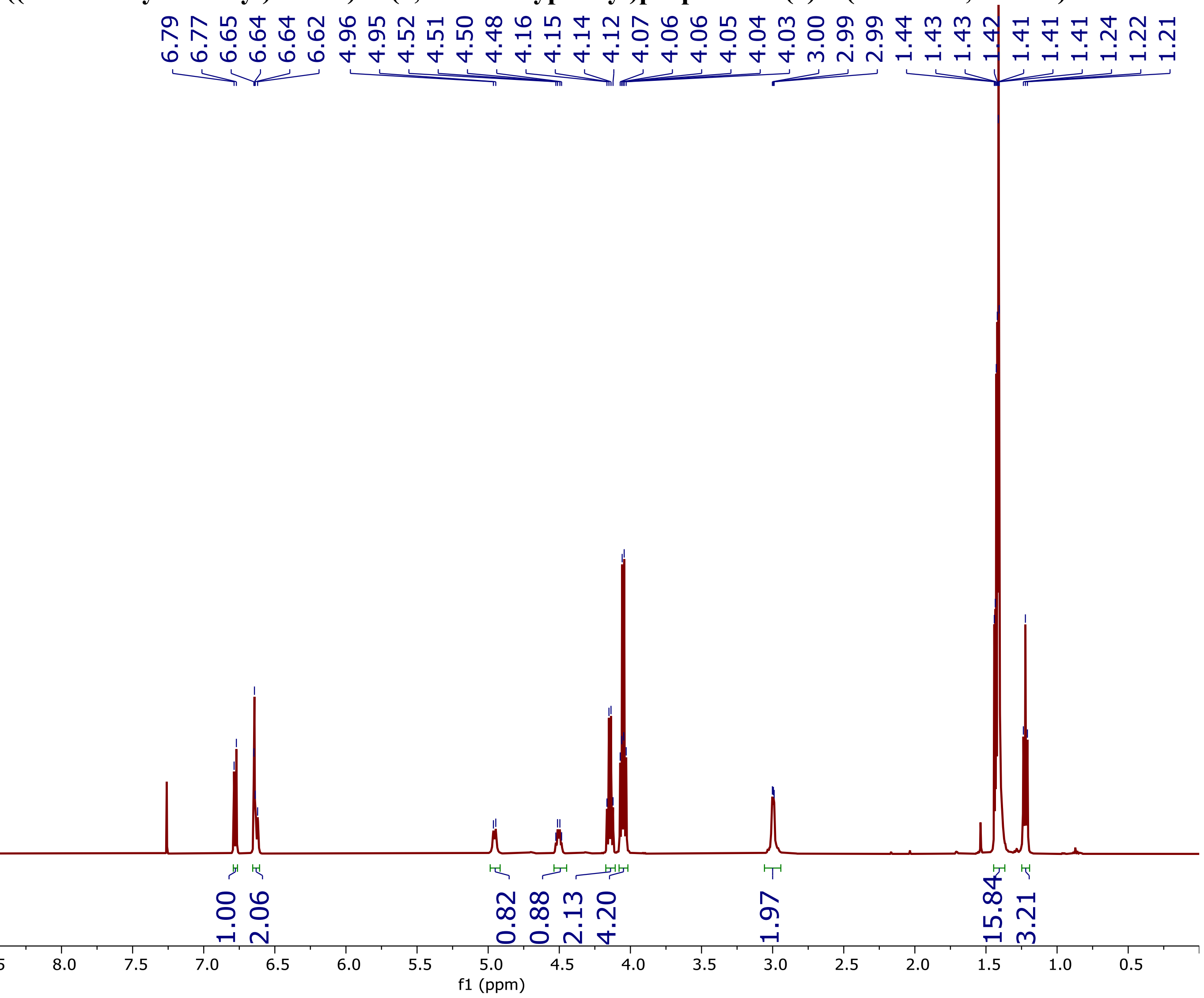

$^{13}C\{^1H\}$-NMR of ethyl (*S*)-2-((*tert*-butoxycarbonyl)amino)-3-(3,4-diethoxyphenyl)propanoate (3) – (126 MHz, $CDCl_3$)

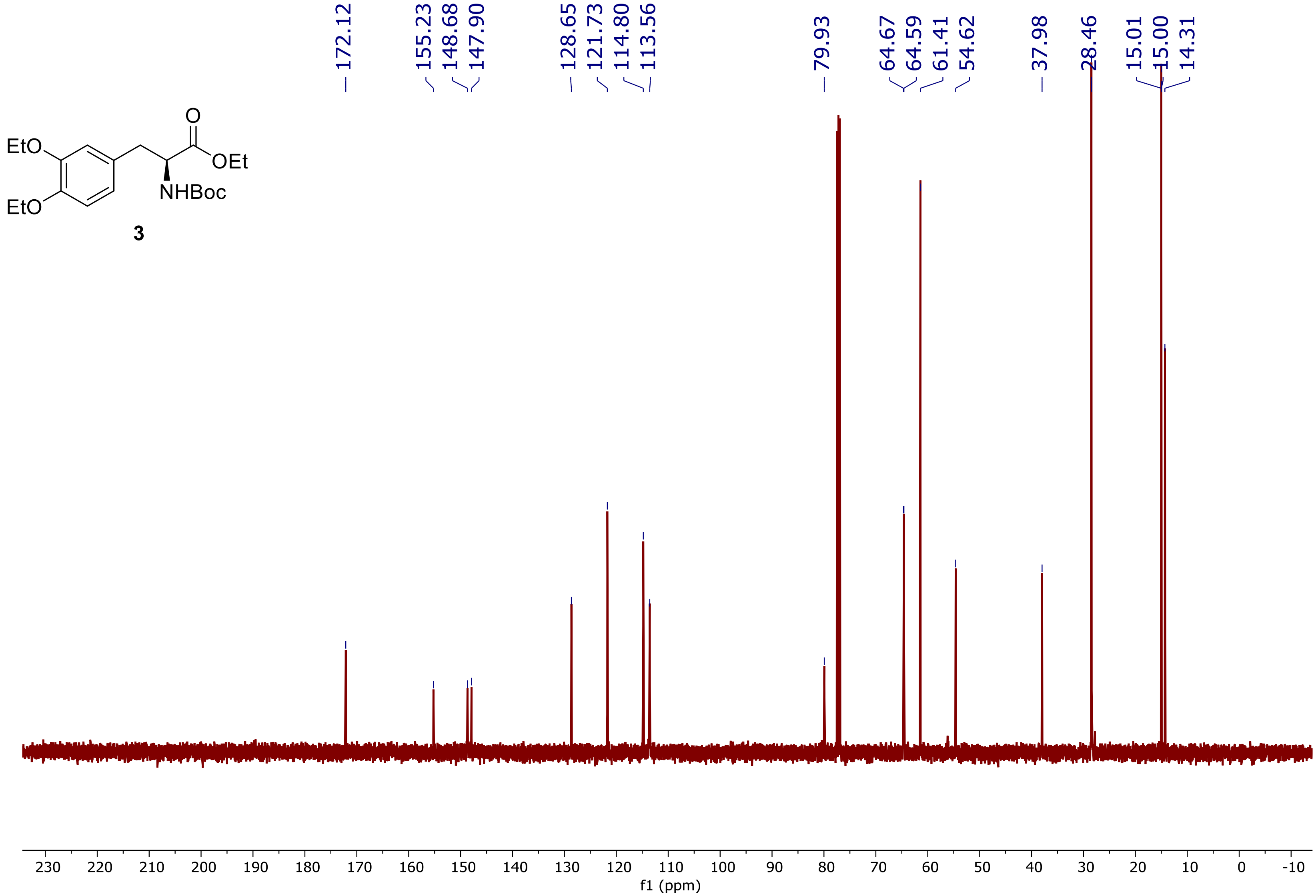

^{1}H-NMR of ethyl (*S*)-2-amino-3-(3,4-dimethoxyphenyl)propanoate (4) – (500 MHz, $CDCl_3$)

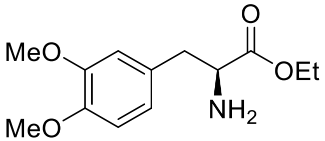

4

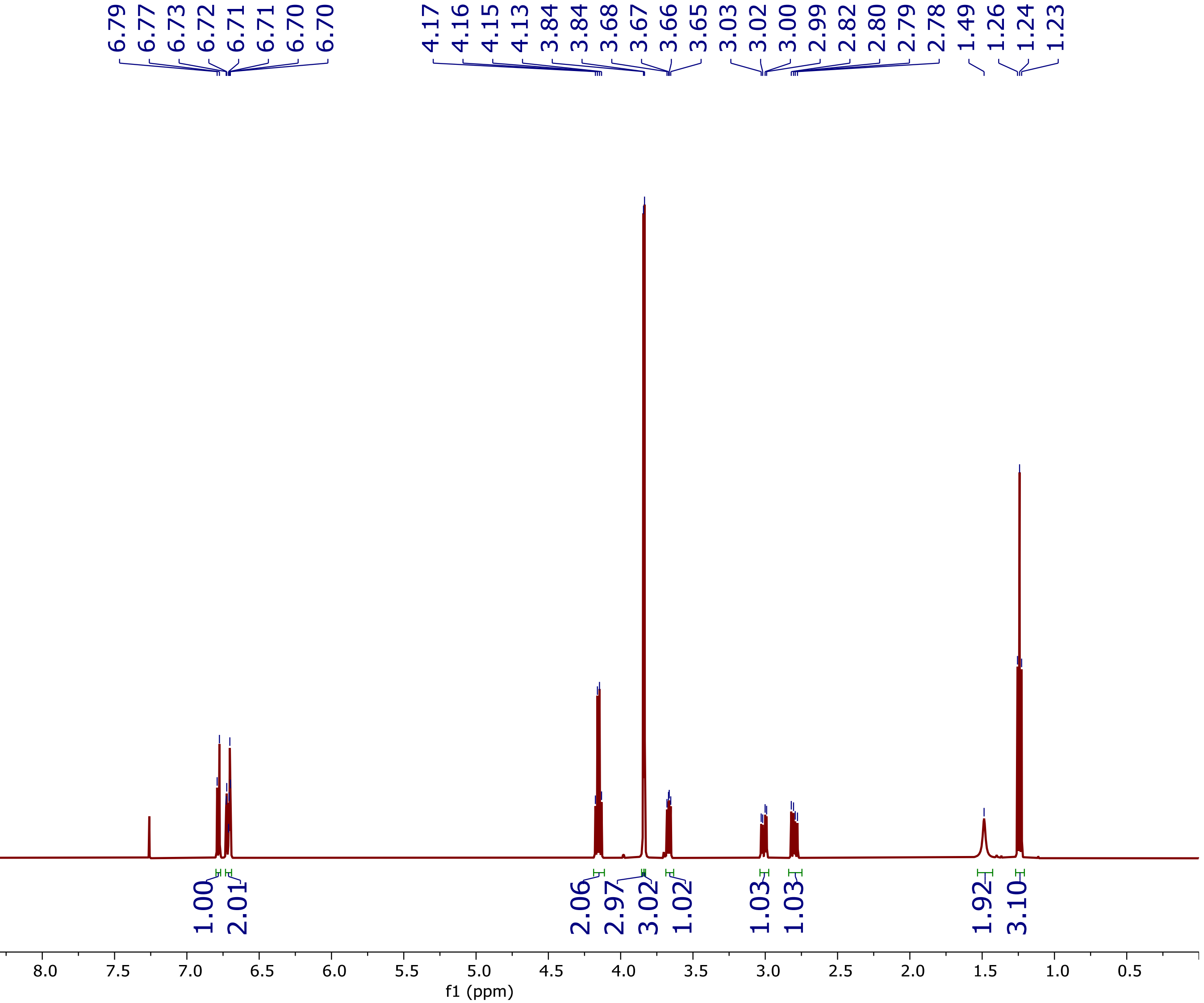

$^{13}C\{^1H\}$-NMR of ethyl (*S*)-2-amino-3-(3,4-dimethoxyphenyl)propanoate (4) – (126 MHz, $CDCl_3$)

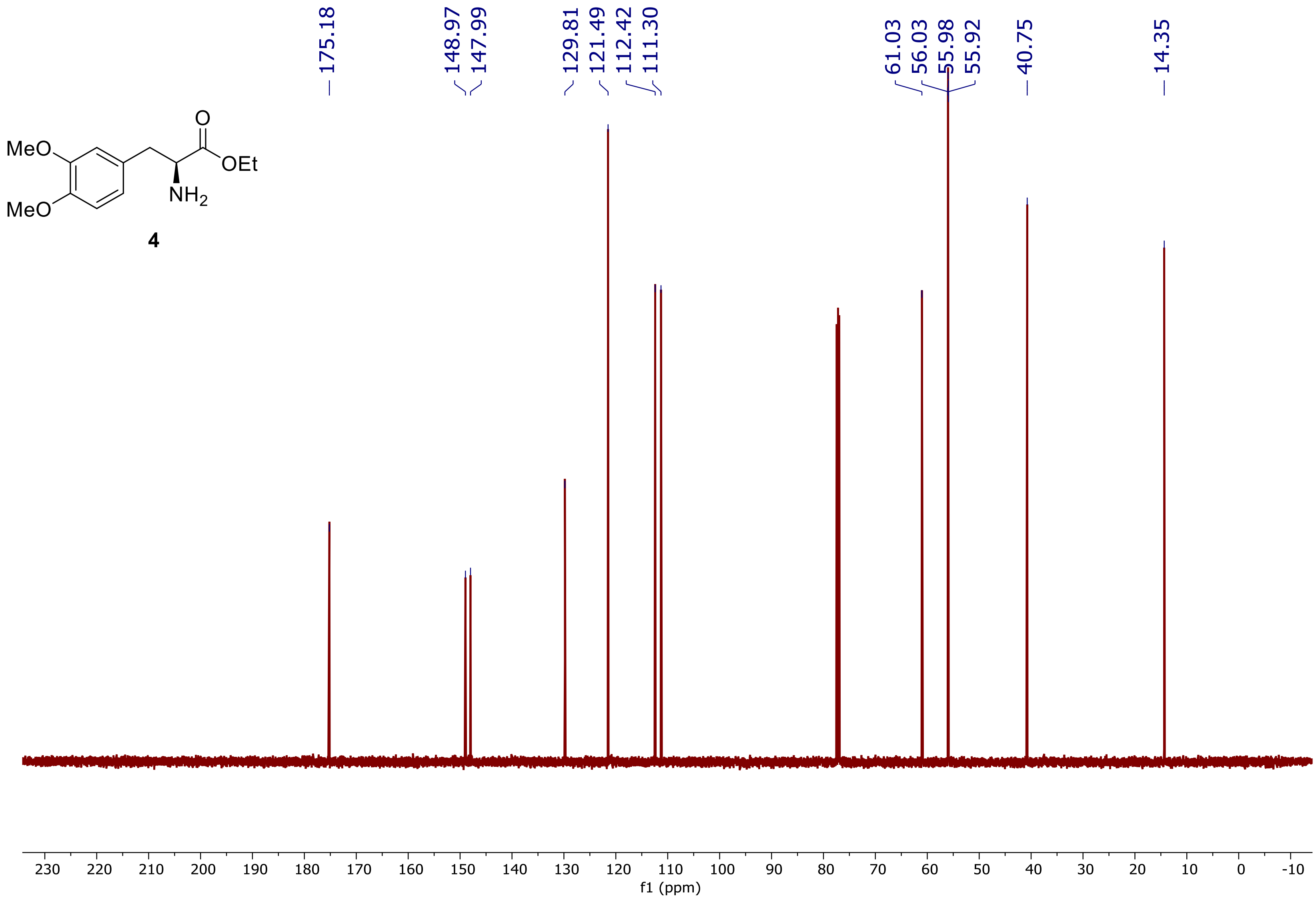

^{1}H-NMR of ethyl (*S*)-3-(3,4-dimethoxyphenyl)-2-(dimethylamino)propanoate (5) – (500 MHz, $CDCl_3$)

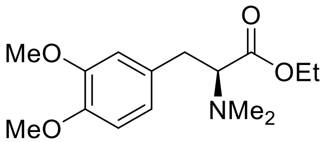

5

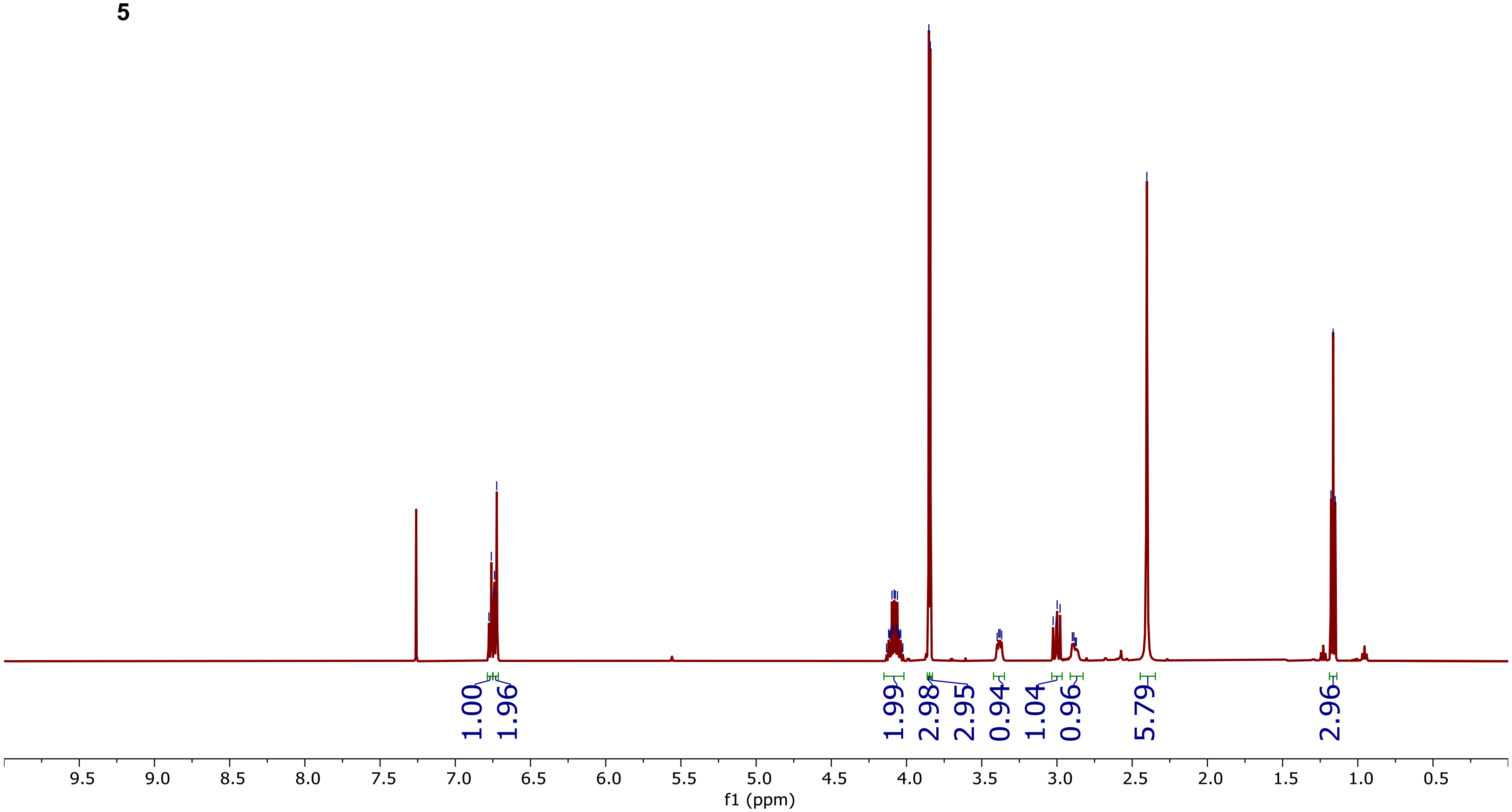

$^{13}C\{^{1}H\}$-NMR of ethyl (*S*)-3-(3,4-dimethoxyphenyl)-2-(dimethylamino)propanoate (5) – (126MHz, $CDCl_3$)

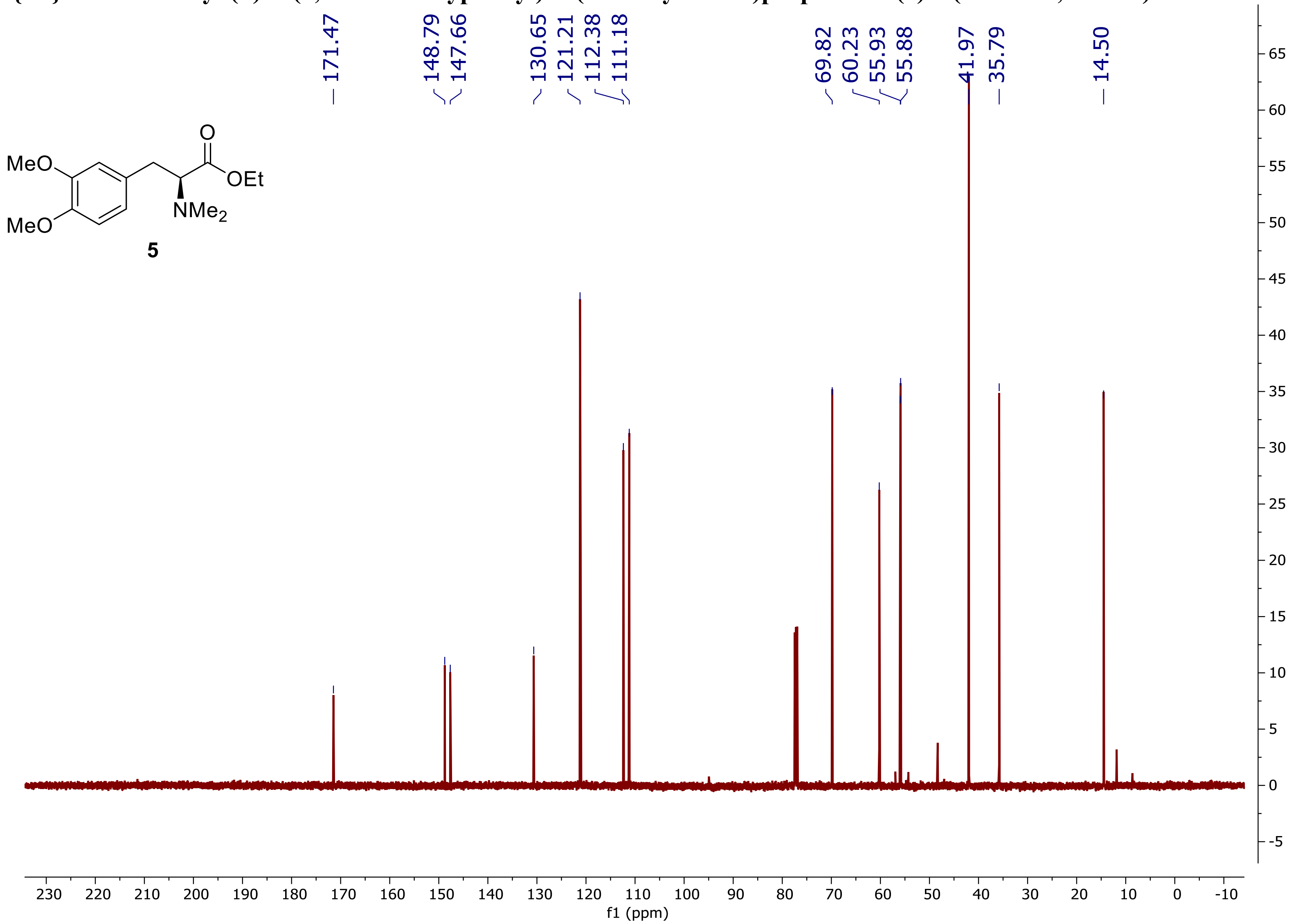

HSQC of ethyl (*S*)-3-(3,4-dimethoxyphenyl)-2-(dimethylamino)propanoate (5) – ($CDCl_3$)

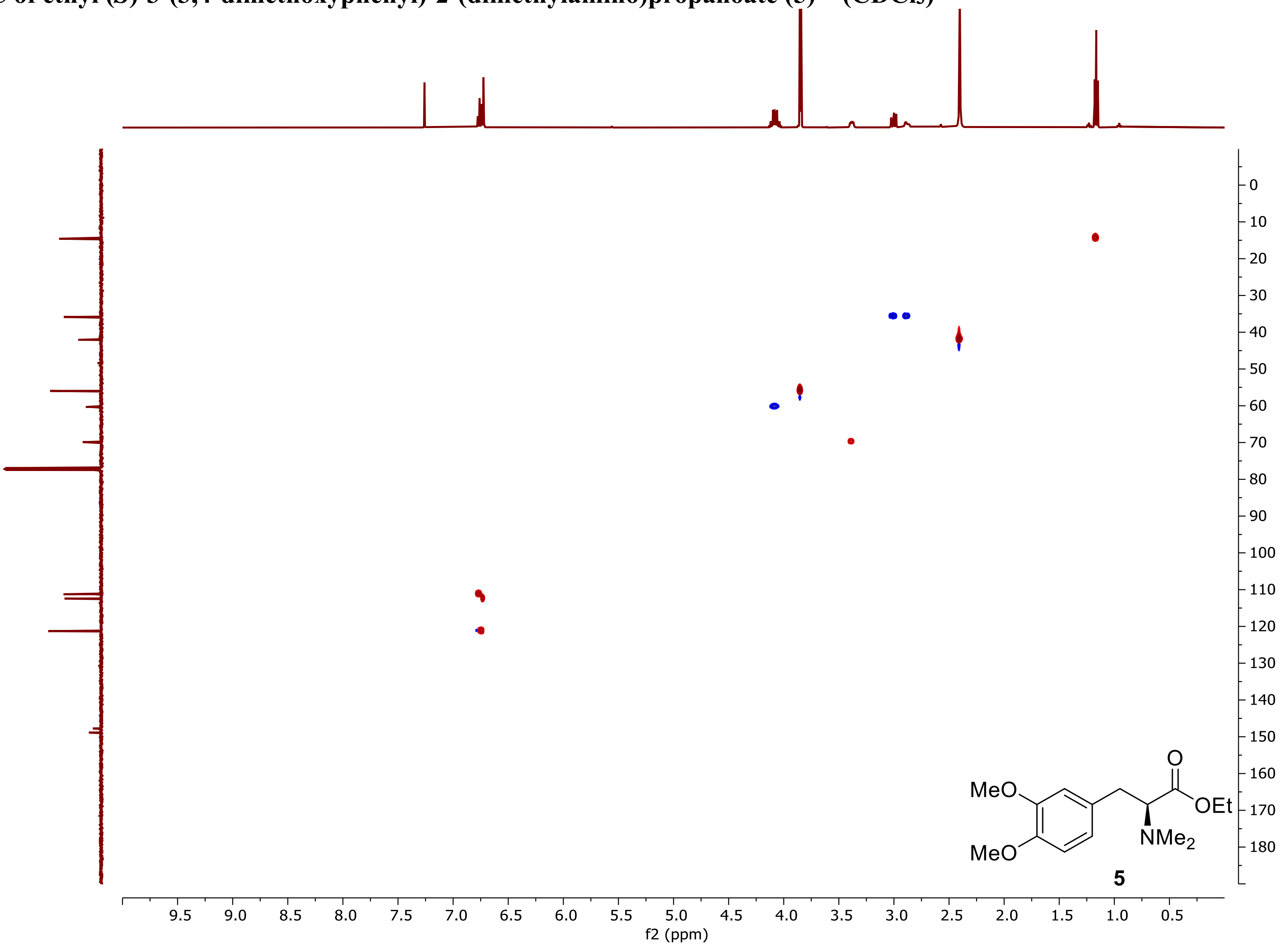

HMBC of ethyl (*S*)-3-(3,4-dimethoxyphenyl)-2-(dimethylamino)propanoate (5) – ($CDCl_3$)

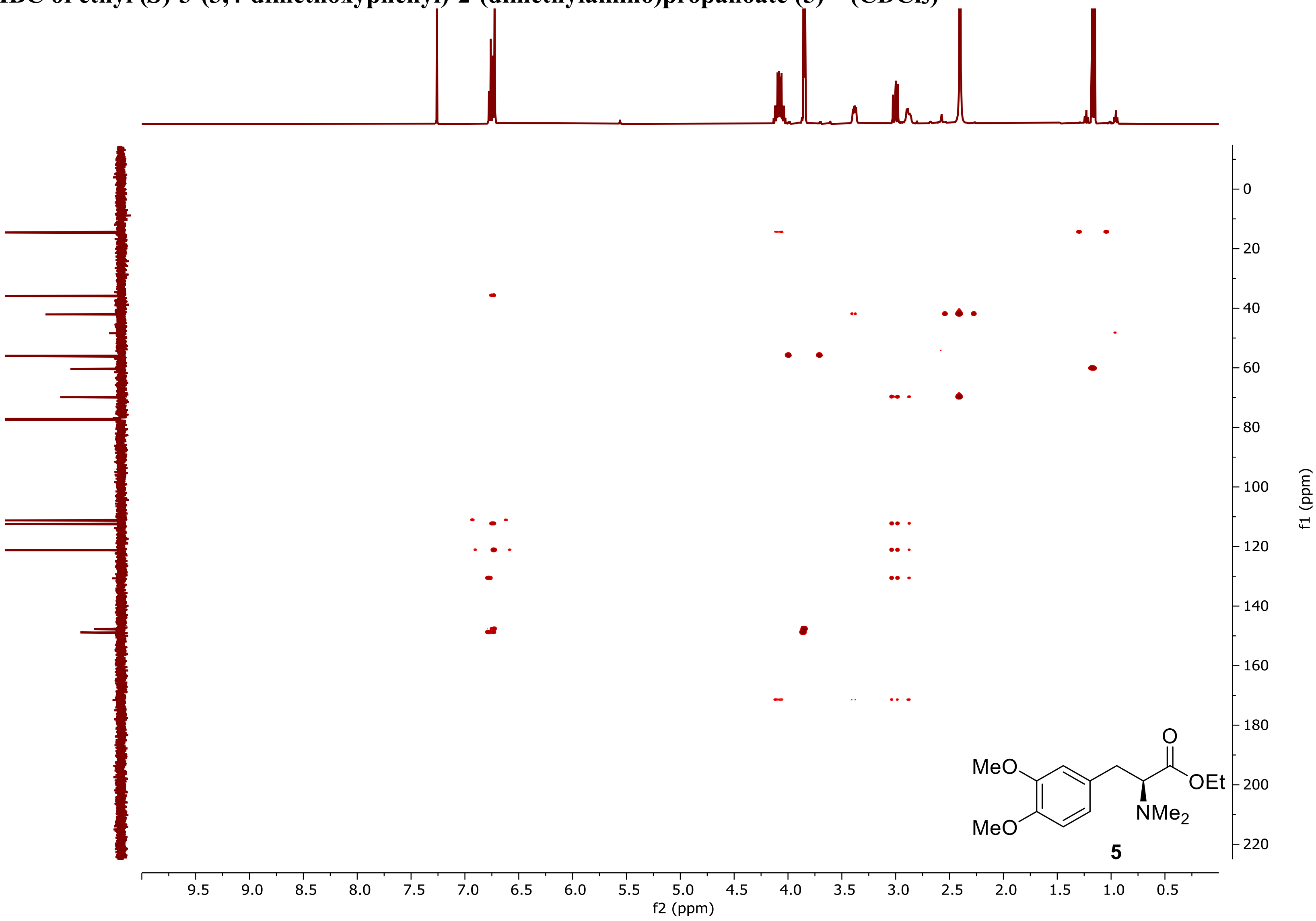

^{1}H-NMR of ethyl (*S*)-3-(benzo[*d*][1,3]dioxol-5-yl)-2-((*tert*-butoxycarbonyl)amino) propanoate (6) – (500 MHz, $CDCl_3$)

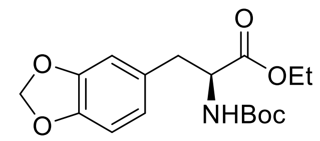

6

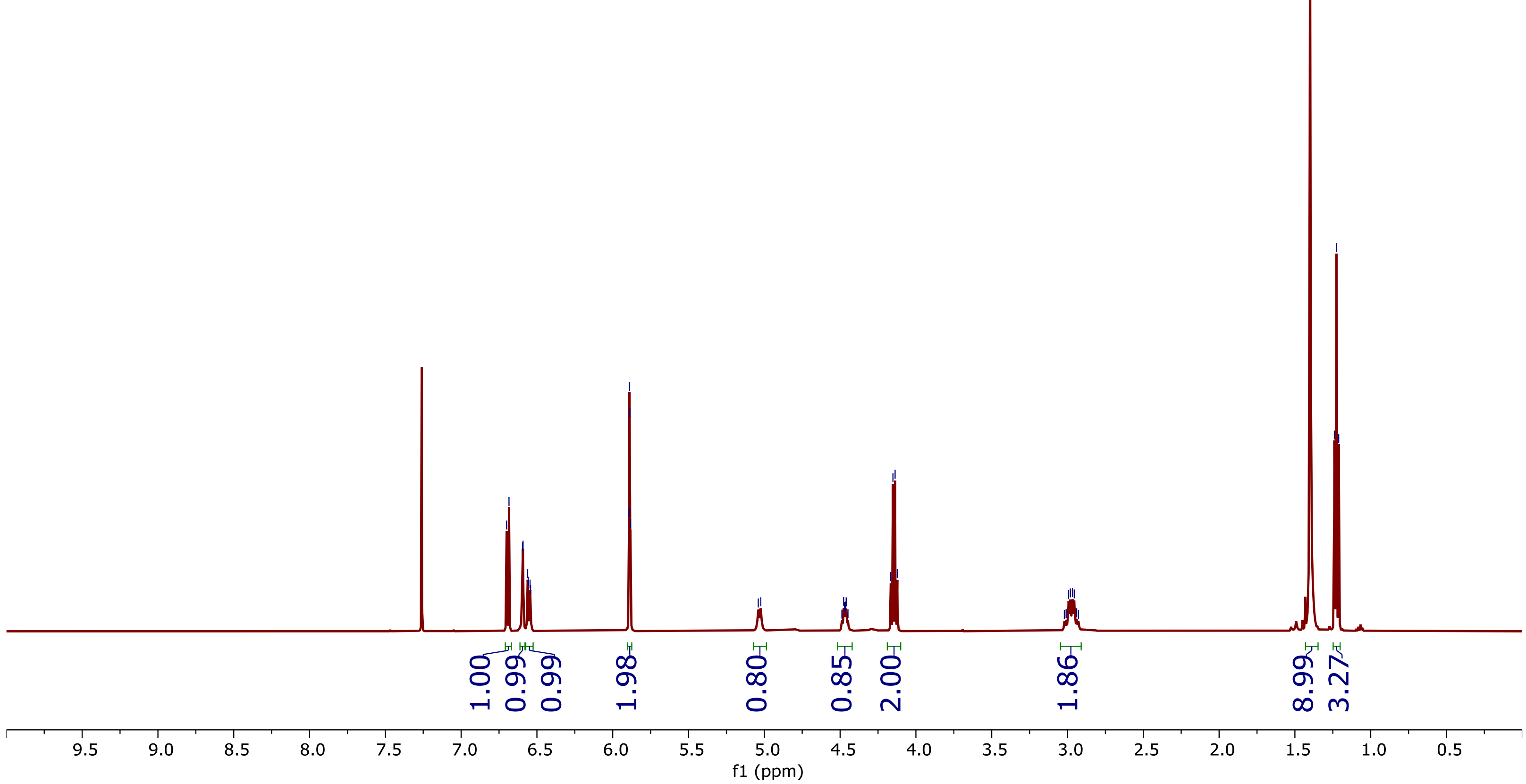

$^{13}C\{^{1}H\}$-NMR of ethyl (*S*)-3-(benzo[*d*][1,3]dioxol-5-yl)-2-((*tert*-butoxycarbonyl)amino) propanoate (6) – (126 MHz, $CDCl_3$)

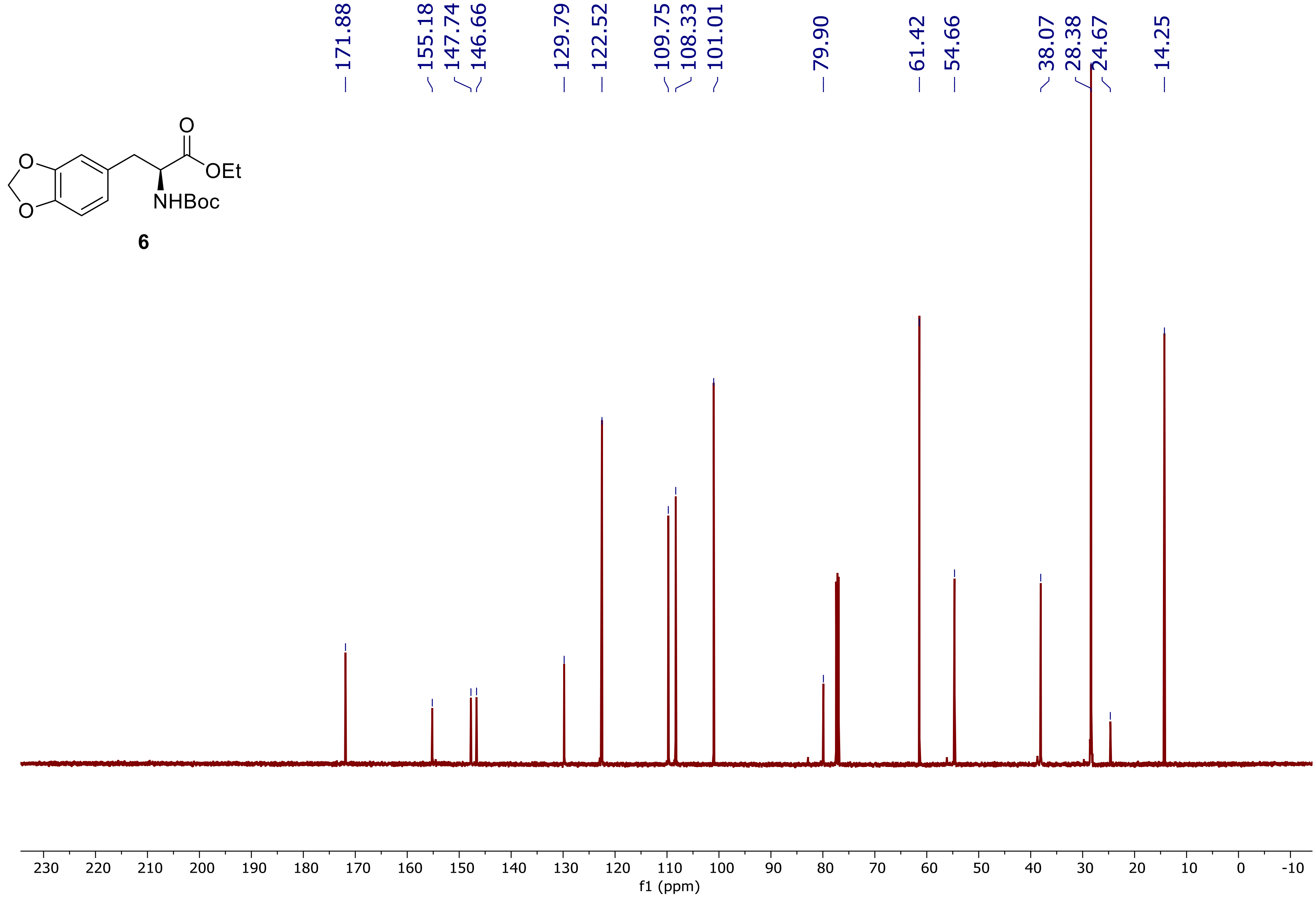

HSQC of ethyl (*S*)-3-(benzo[*d*][1,3]dioxol-5-yl)-2-((*tert*-butoxycarbonyl)amino) propanoate (6) – ($CDCl_3$)

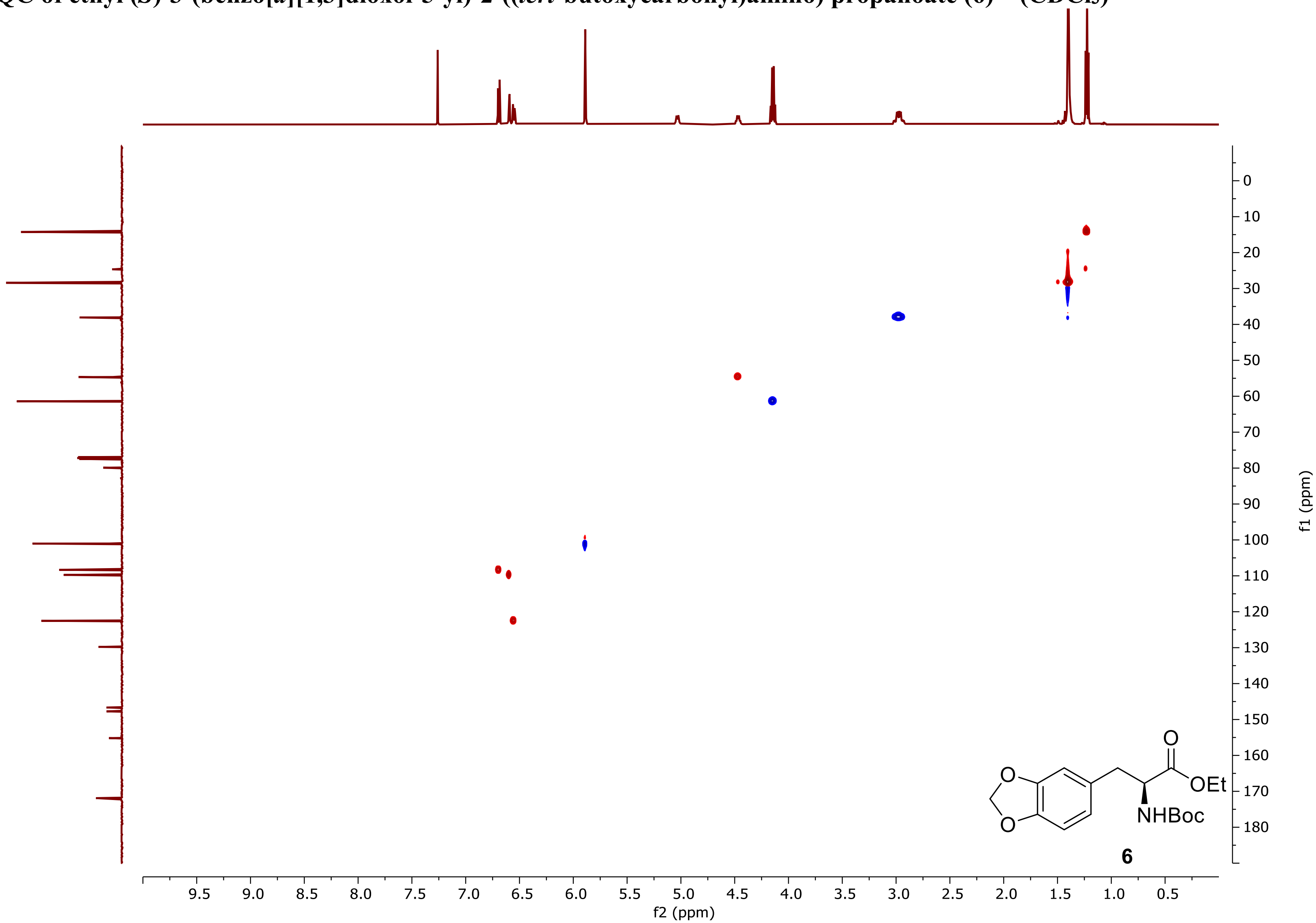

HSQC of ethyl (*S*)-3-(benzo[*d*][1,3]dioxol-5-yl)-2-((*tert*-butoxycarbonyl)amino) propanoate (6) – ($CDCl_3$) – 5.5 to 7.5 ppm

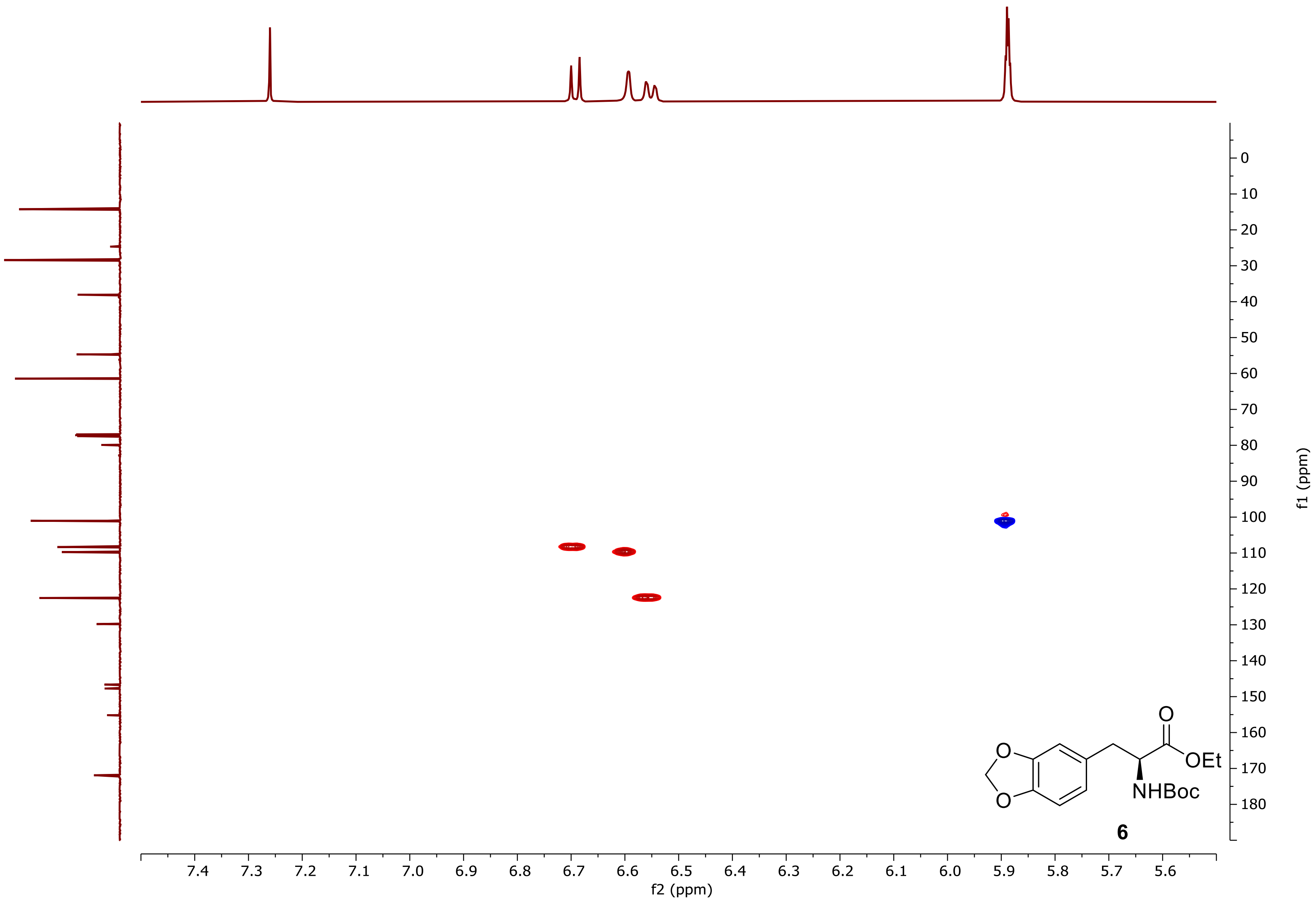

HMBC of ethyl (*S*)-3-(benzo[*d*][1,3]dioxol-5-yl)-2-((*tert*-butoxycarbonyl)amino) propanoate (6) – ($CDCl_3$)

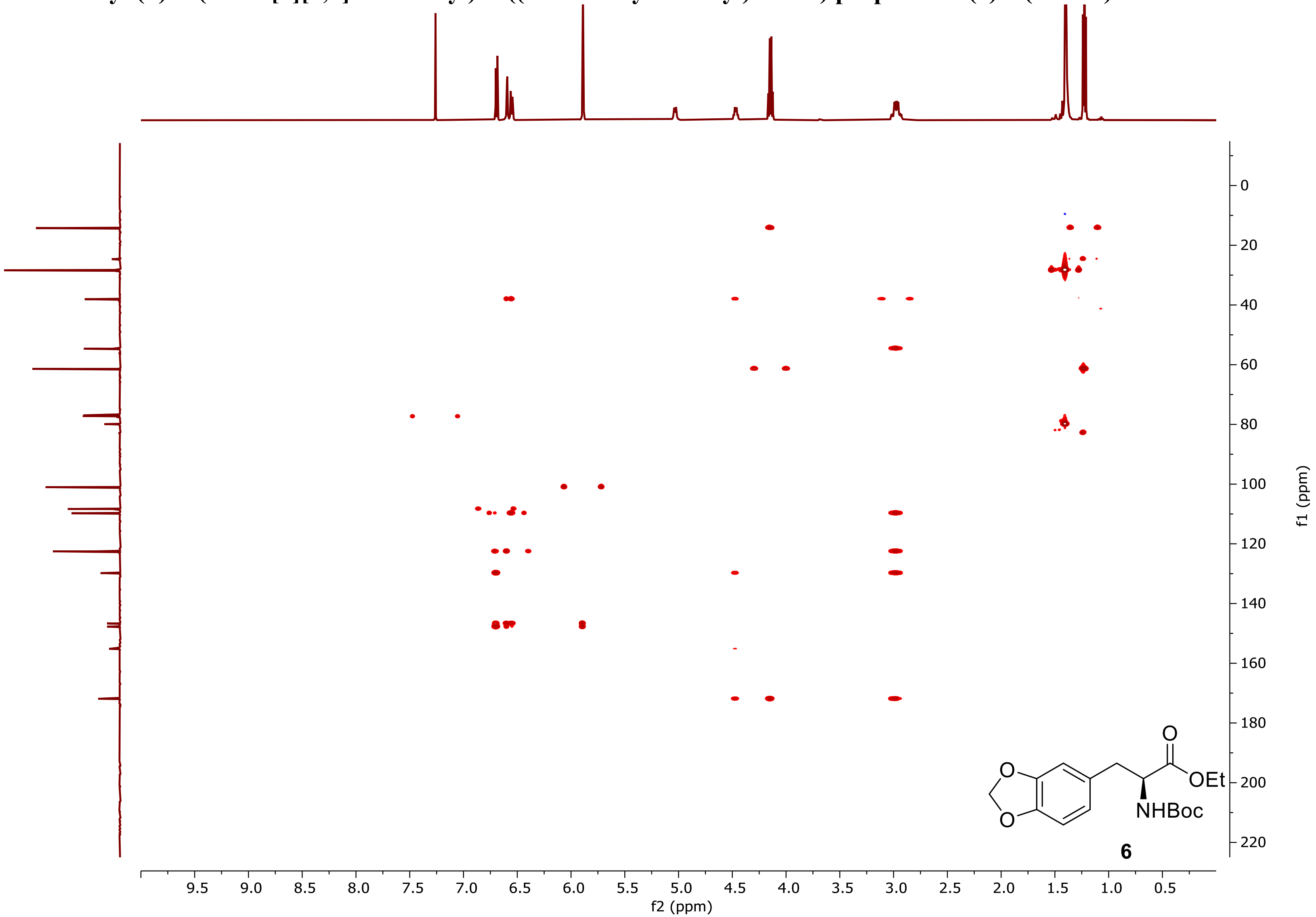

HMBC of ethyl (*S*)-3-(benzo[*d*][1,3]dioxol-5-yl)-2-((*tert*-butoxycarbonyl)amino) propanoate (6) – ($CDCl_3$) – 5.5 to 7.5 ppm

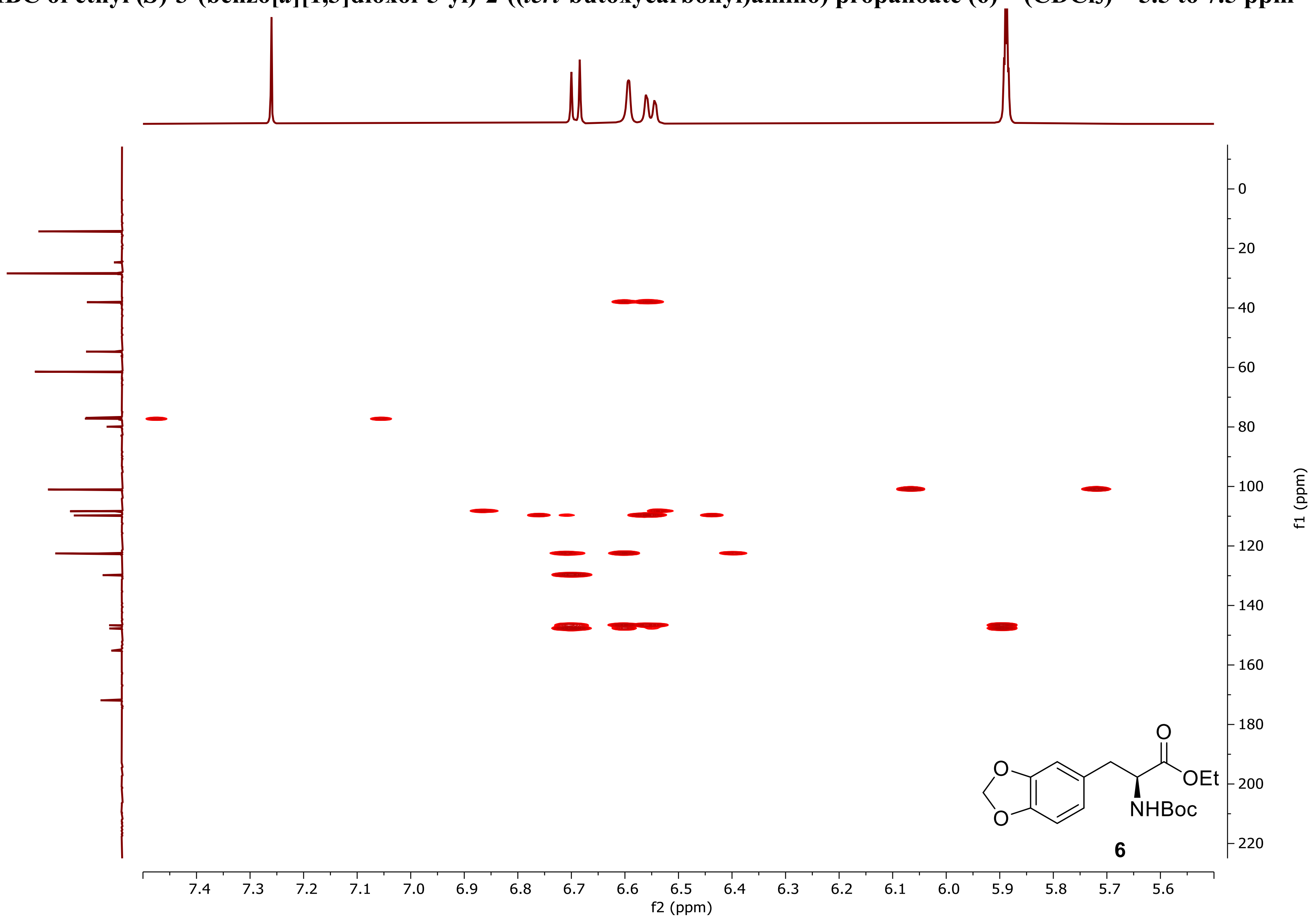

^{1}H-NMR of ethyl (*S*)-2-amino-3-(benzo[*d*][1,3]dioxol-5-yl)propanoate (7) – (500 MHz, $CDCl_3$)

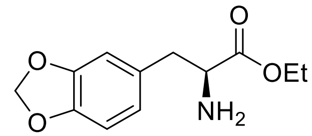

7

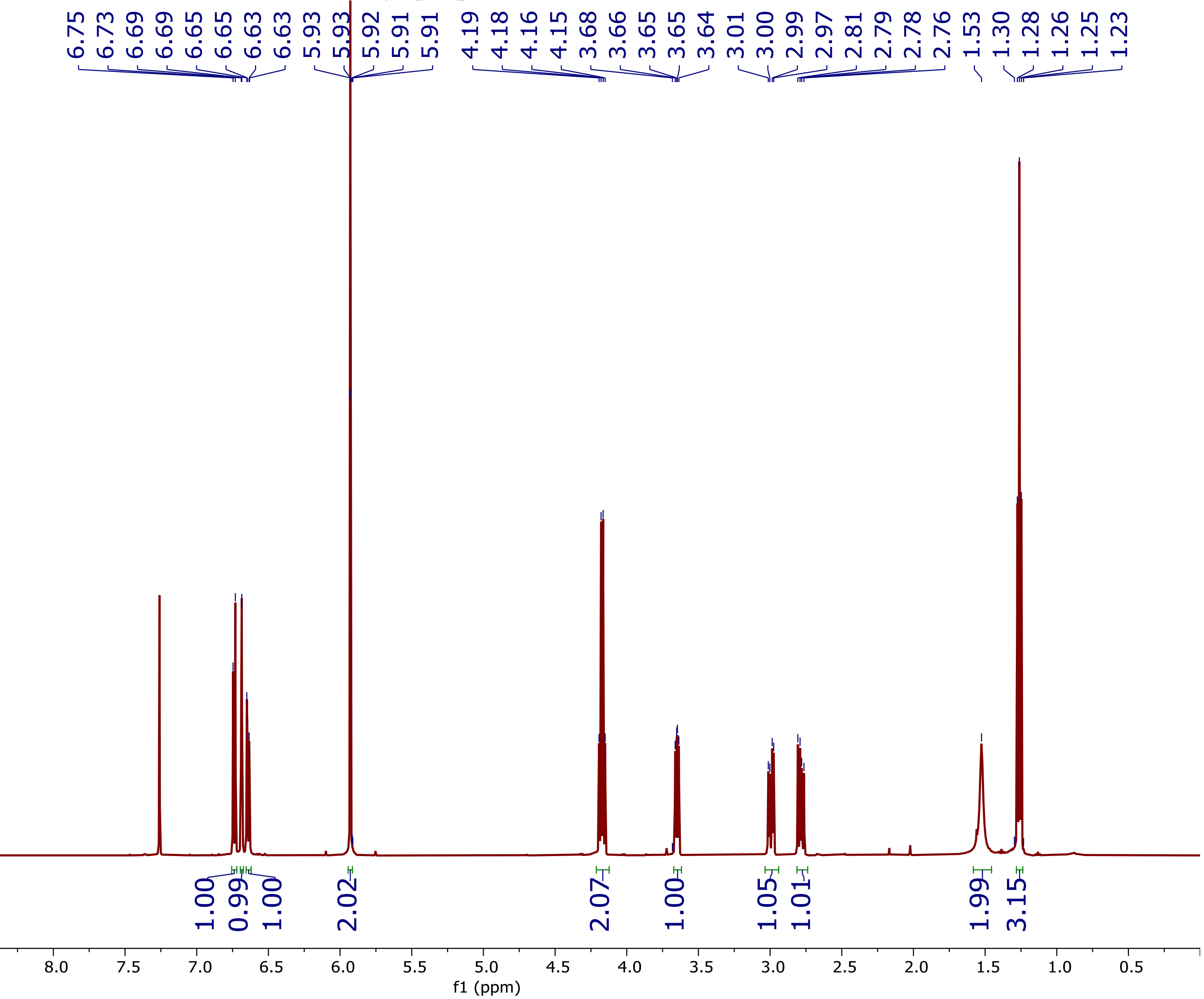

$^{13}C\{^1H\}$-NMR of ethyl (*S*)-2-amino-3-(benzo[*d*][1,3]dioxol-5-yl)propanoate (7) – (126 MHz, CDCl$_3$)

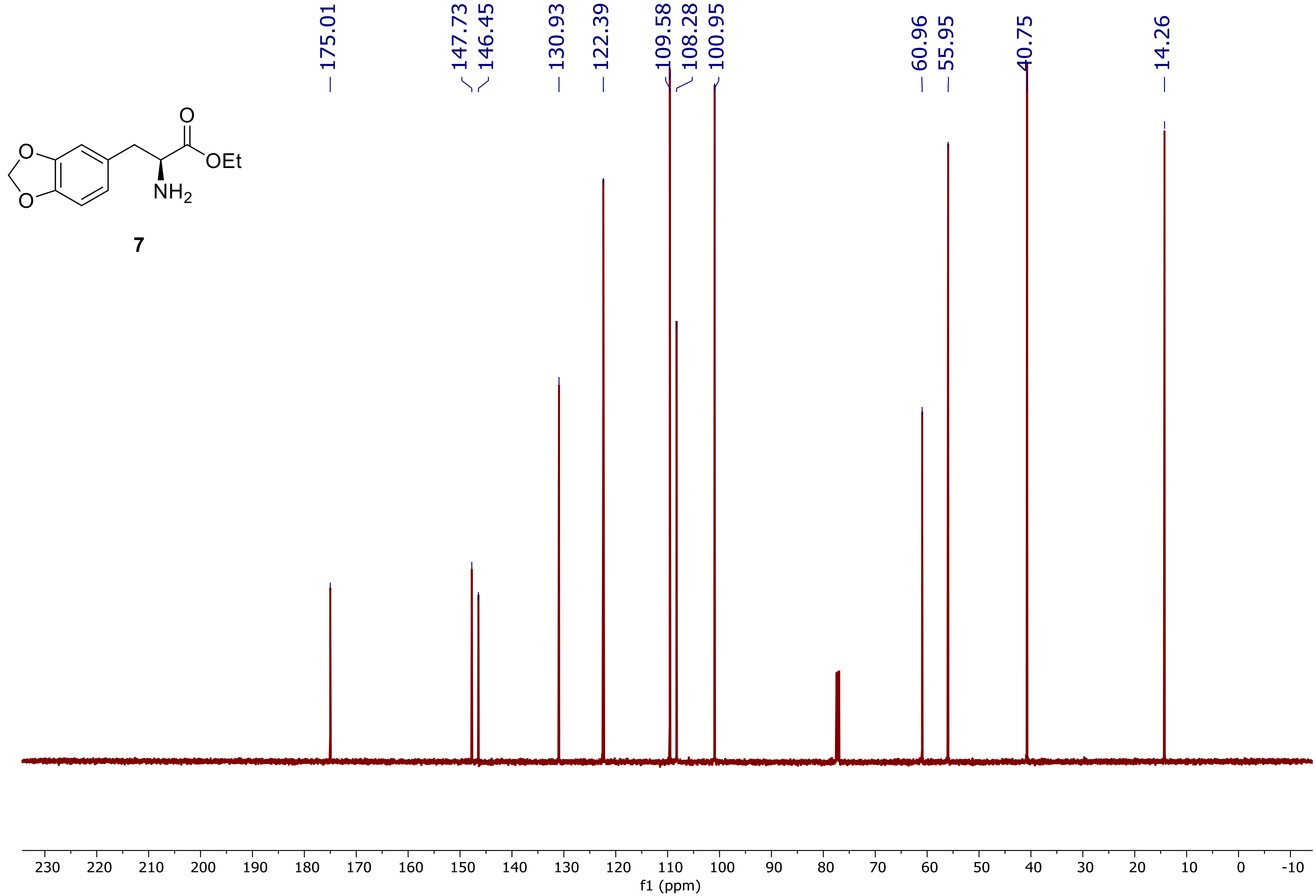

HSQC of ethyl (*S*)-2-amino-3-(benzo[*d*][1,3]dioxol-5-yl)propanoate (7) – ($CDCl_3$)

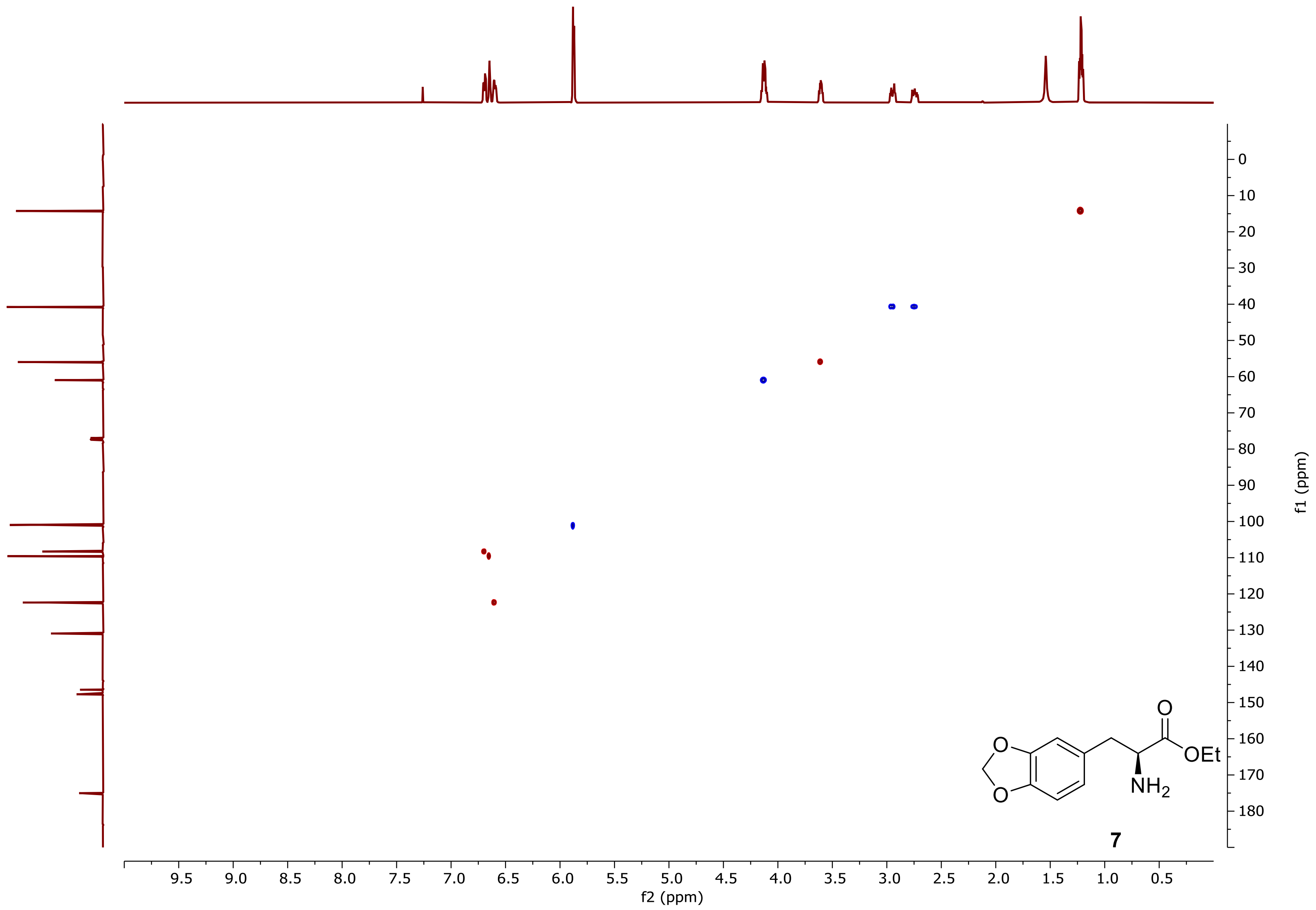

HSQC of ethyl (*S*)-2-amino-3-(benzo[*d*][1,3]dioxol-5-yl)propanoate (7) – ($CDCl_3$) – 5.5 to 7.5 ppm

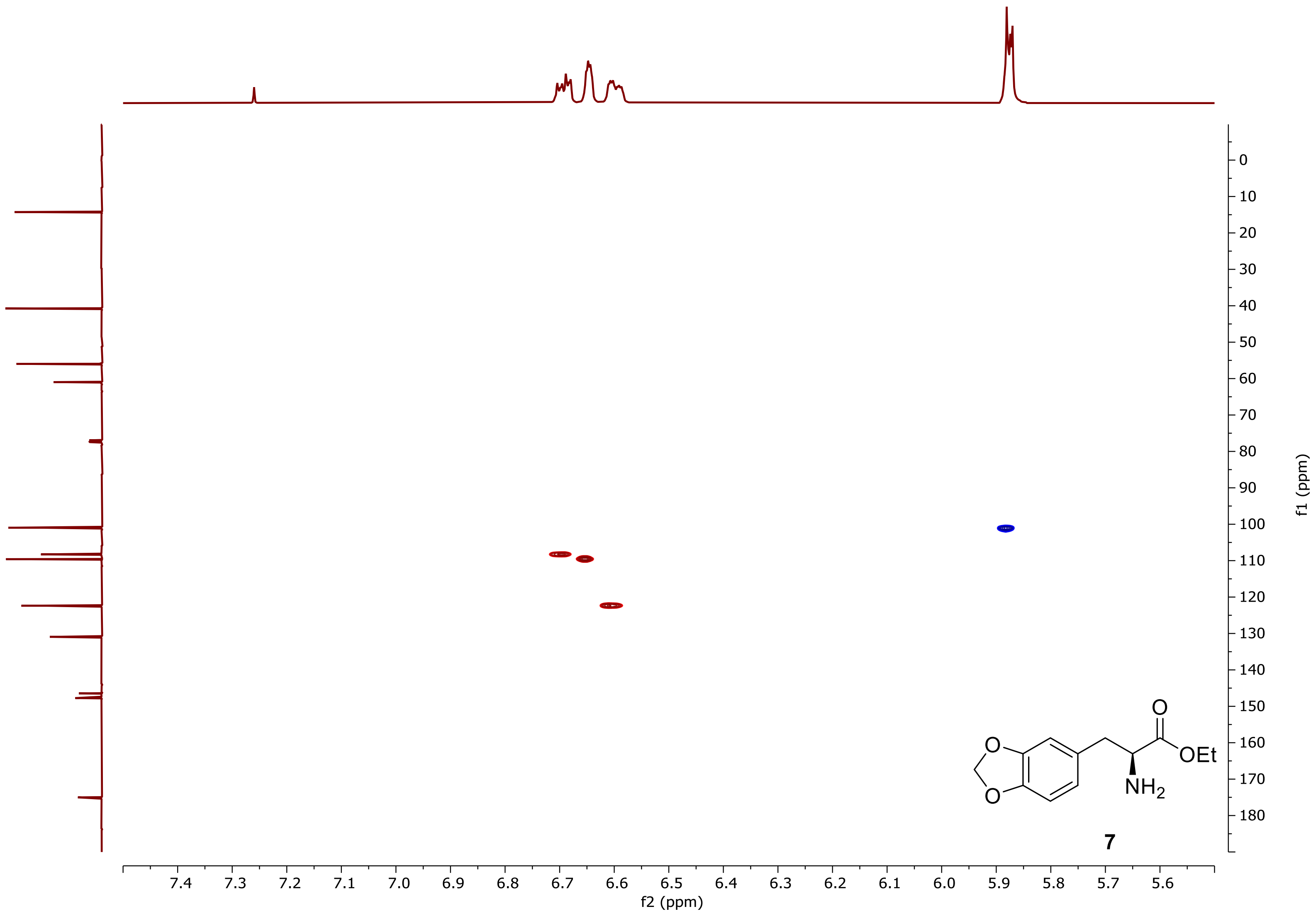

HMBC of ethyl (*S*)-2-amino-3-(benzo[*d*][1,3]dioxol-5-yl)propanoate (7) – ($CDCl_3$)

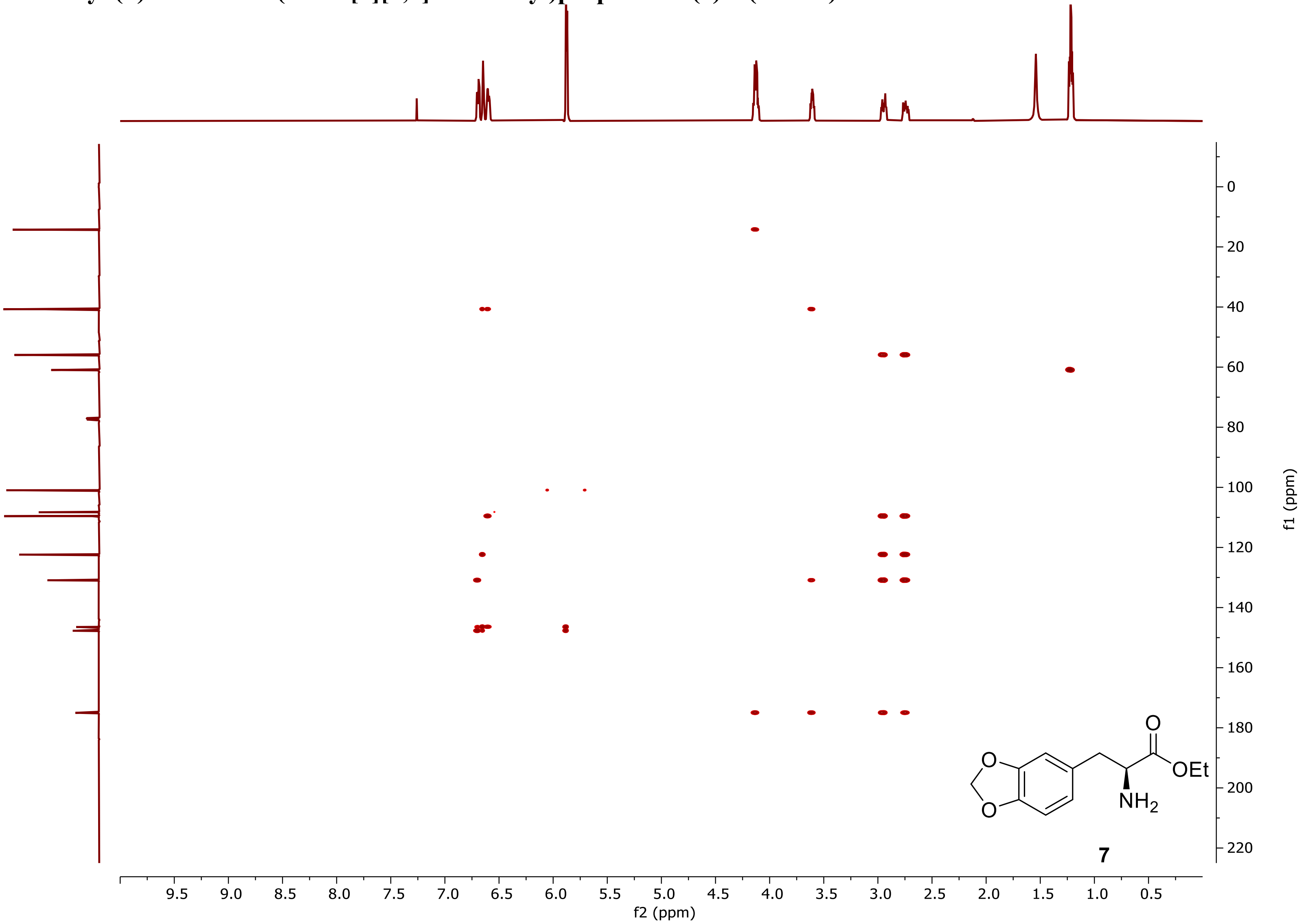

7

HMBC of ethyl (*S*)-2-amino-3-(benzo[*d*][1,3]dioxol-5-yl)propanoate (7) – ($CDCl_3$) – 5.5 to 7.5 ppm

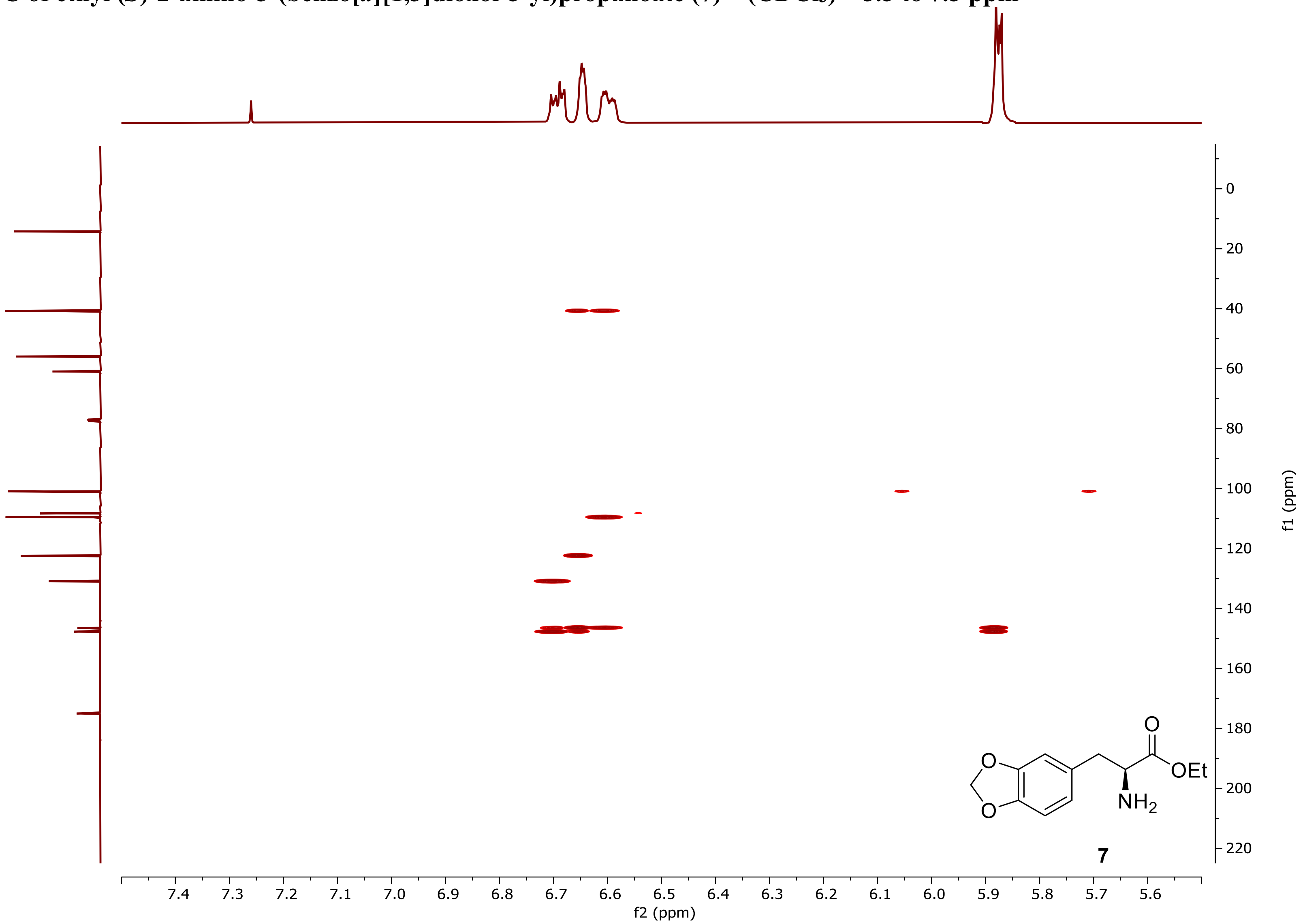

^{1}H-NMR of ethyl (*S*)-3-(benzo[*d*][1,3]dioxol-5-yl)-2-(dimethylamino)propanoate (8) – (500 MHz, CDCl$_3$)

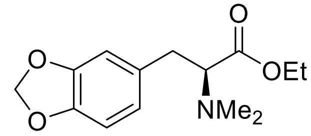

8

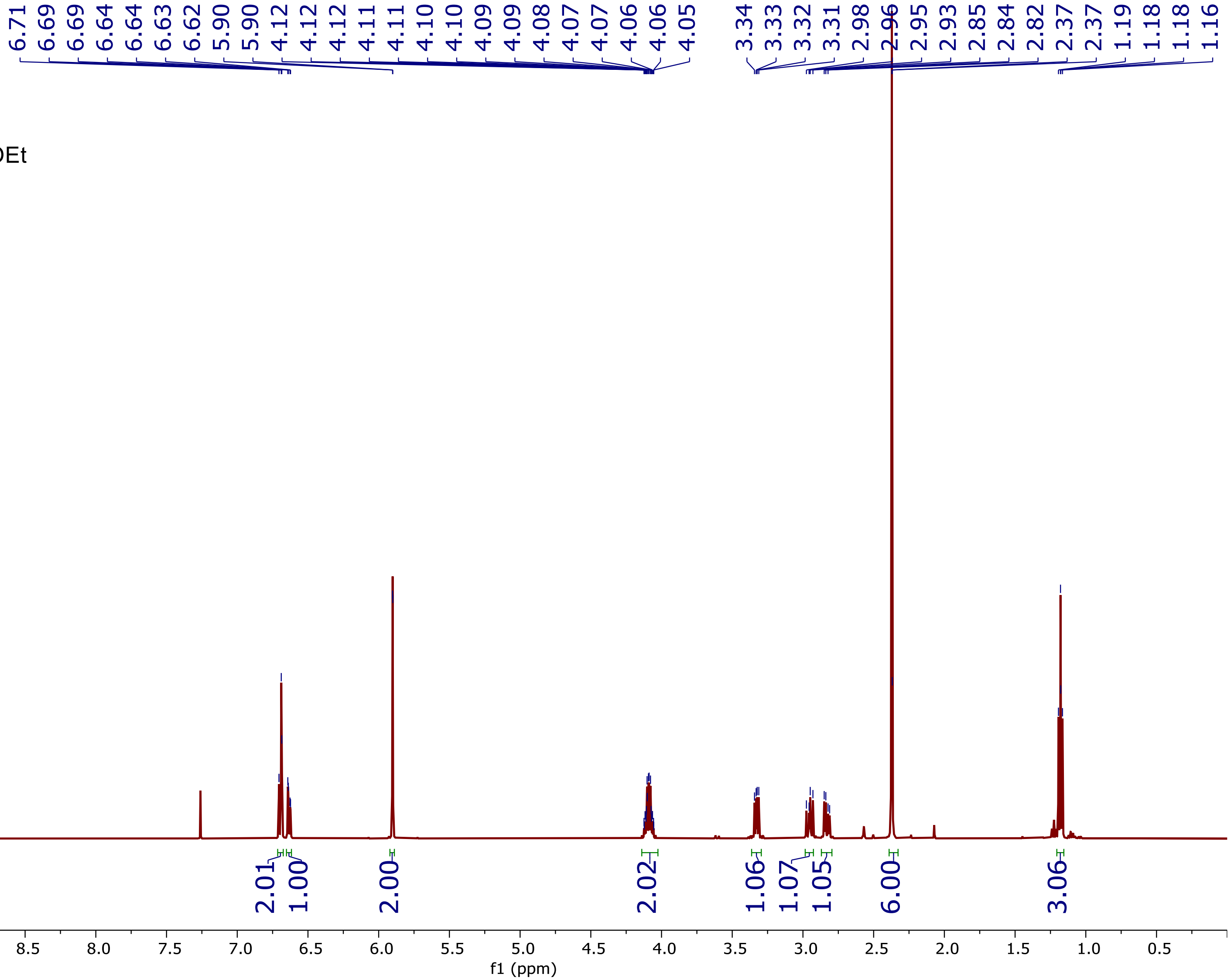

$^{13}C\{^1H\}$-NMR of ethyl (*S*)-3-(benzo[*d*][1,3]dioxol-5-yl)-2-(dimethylamino)propanoate (8) – (126 MHz, $CDCl_3$)

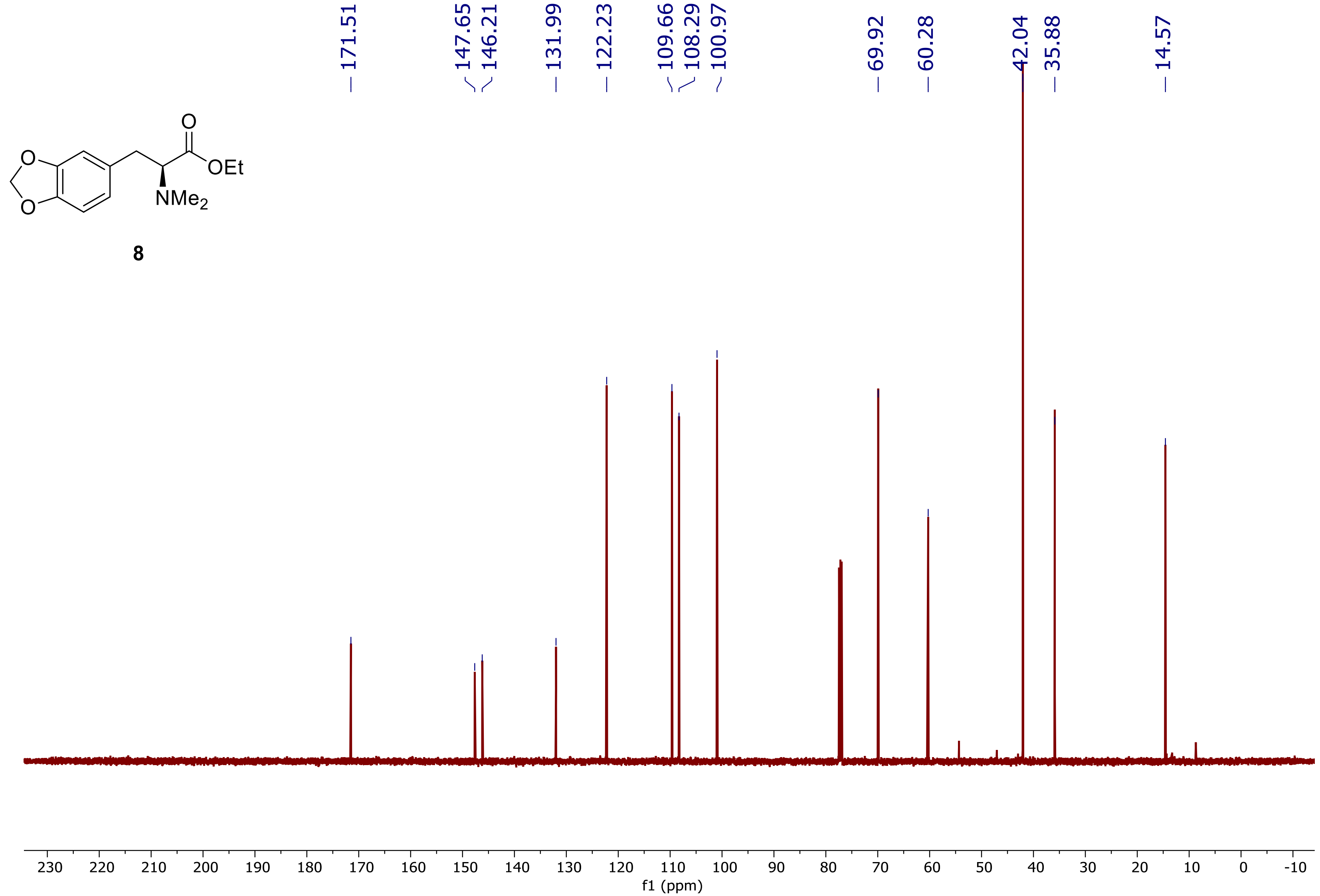

HSQC of ethyl (*S*)-3-(benzo[*d*][1,3]dioxol-5-yl)-2-(dimethylamino)propanoate (8) – ($CDCl_3$)

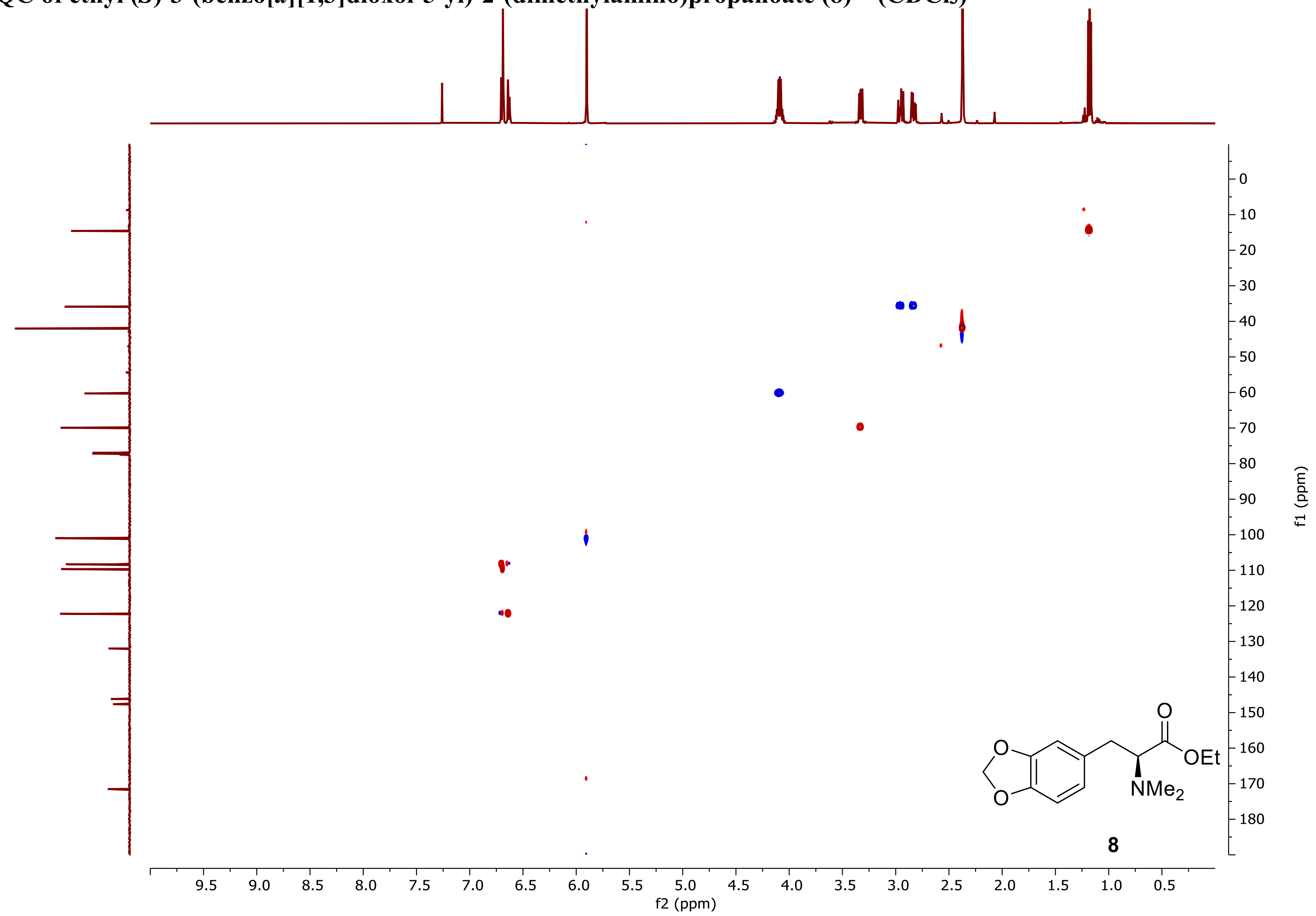

HSQC of ethyl (*S*)-3-(benzo[*d*][1,3]dioxol-5-yl)-2-(dimethylamino)propanoate (8) – ($CDCl_3$) – 5.5 to 7.5 ppm

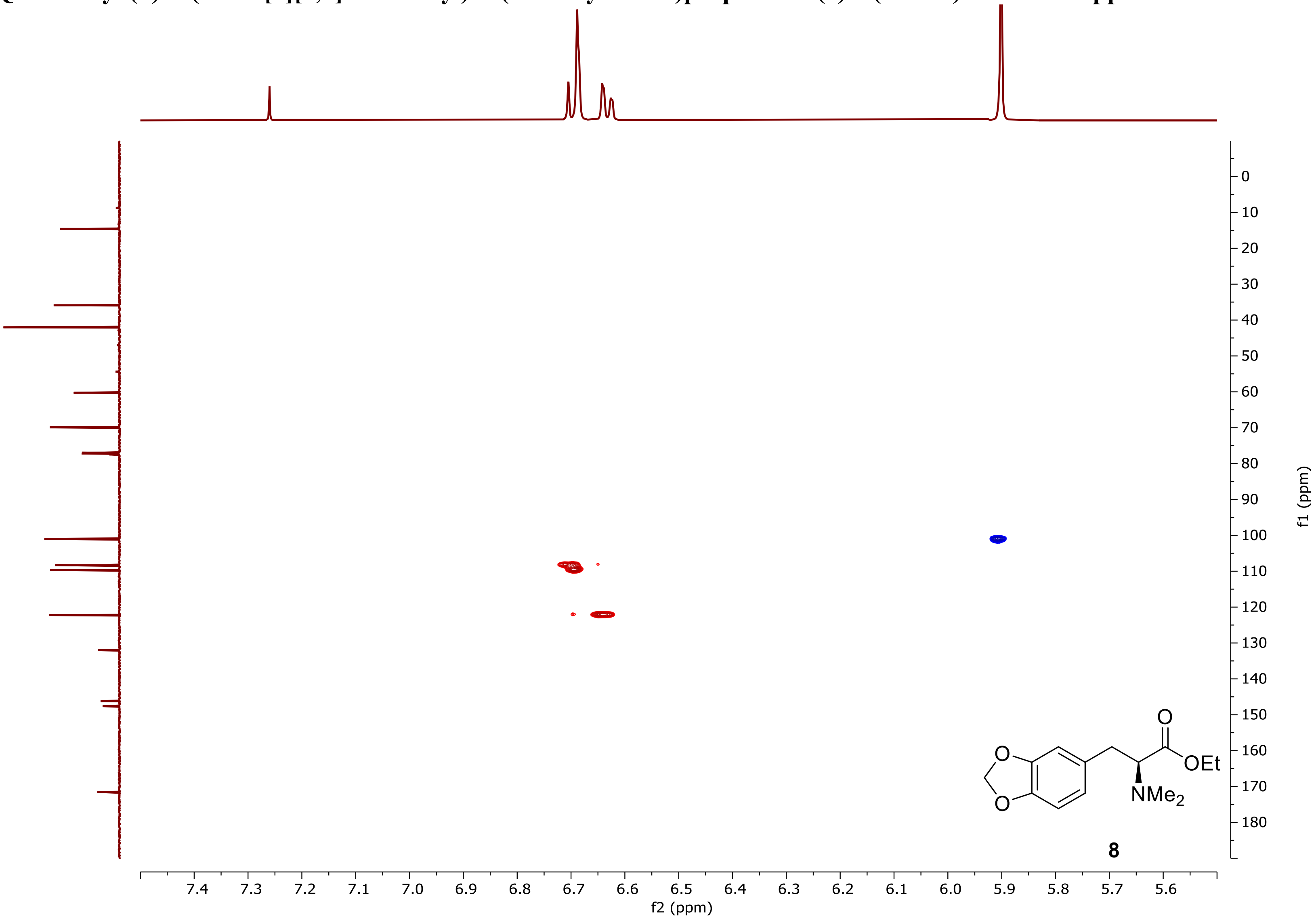

HMBC of ethyl (*S*)-3-(benzo[*d*][1,3]dioxol-5-yl)-2-(dimethylamino)propanoate (8) – ($CDCl_3$)

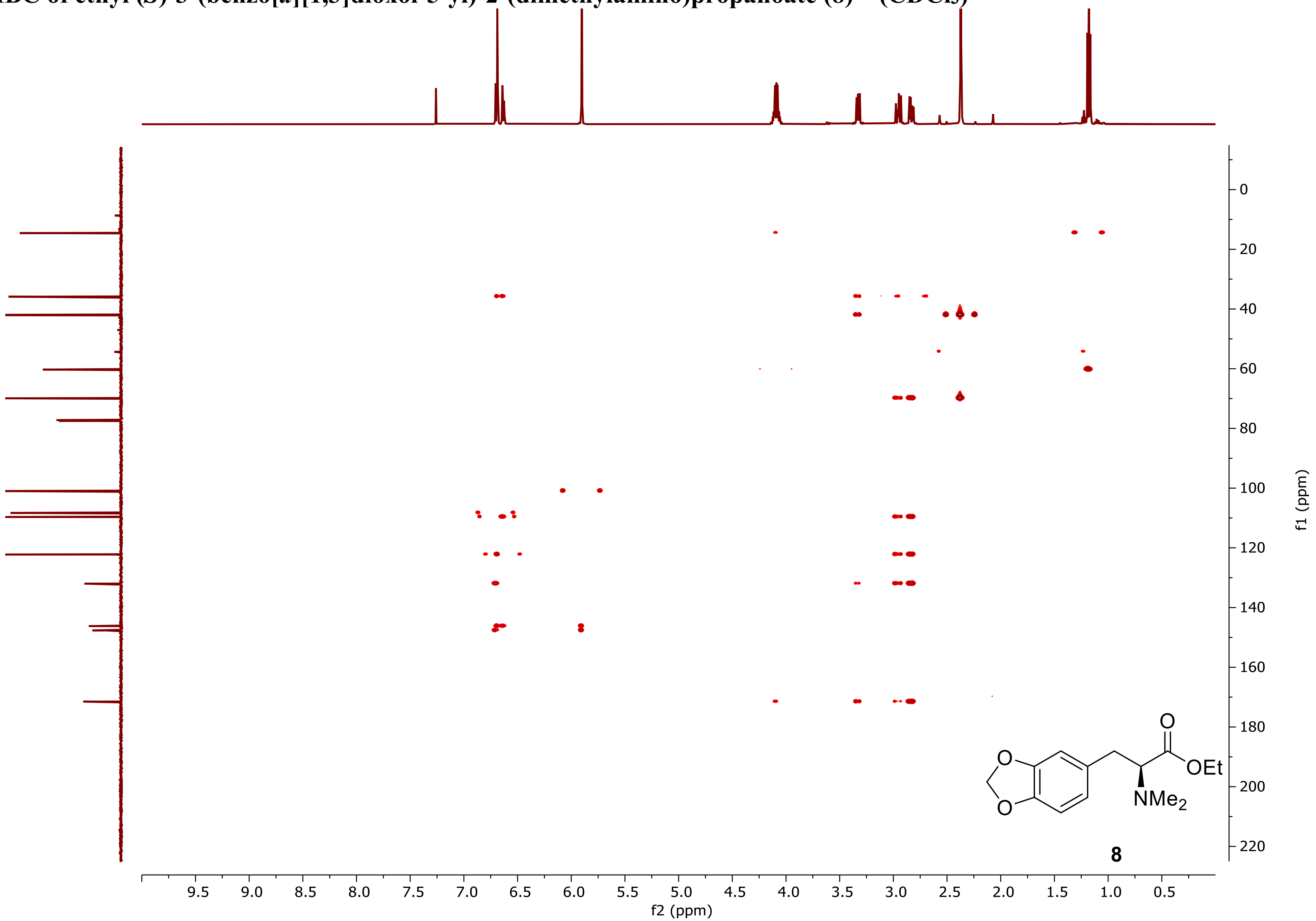

HMBC of ethyl (*S*)-3-(benzo[*d*][1,3]dioxol-5-yl)-2-(dimethylamino)propanoate (8) – ($CDCl_3$) – 5.5 to 7.5 ppm

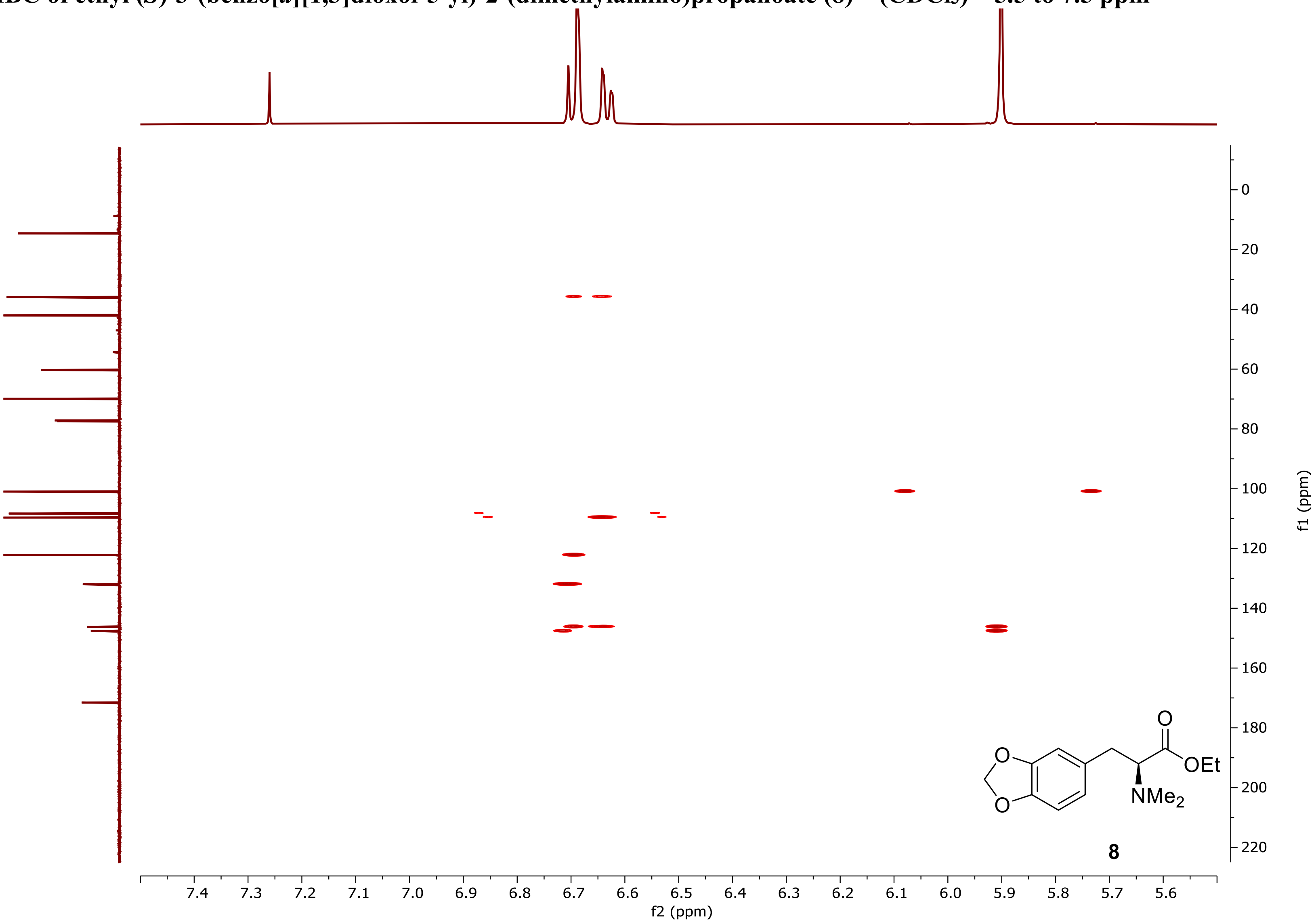

^{1}H-NMR of ethyl (*S*)-3-(benzo[*d*][1,3]dioxol-5-yl)-2-((*tert*-butoxycarbonyl)(methyl)amino) propanoate (9) – (500 MHz, $CDCl_3$)

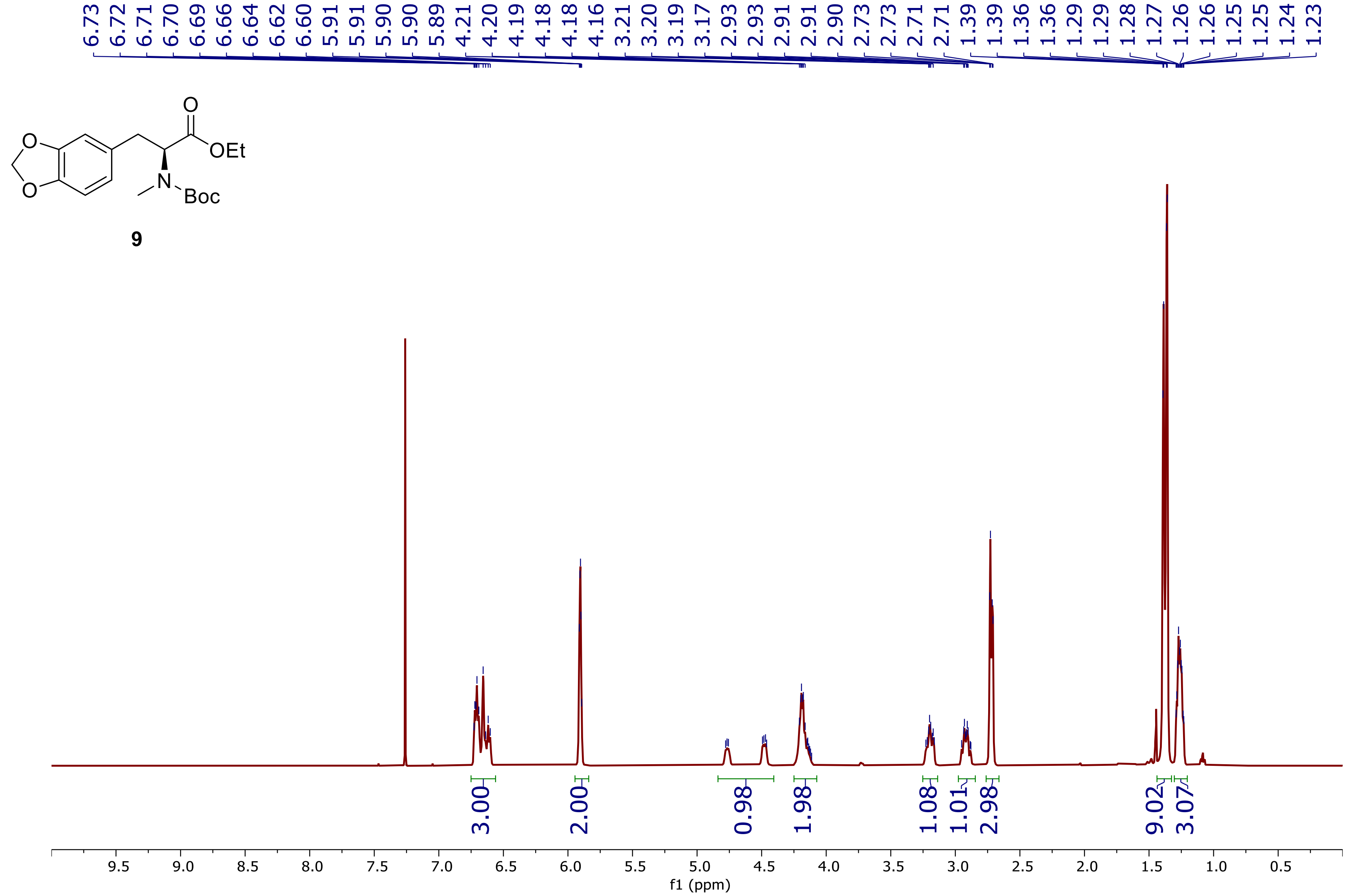

$^{13}C\{^{1}H\}$-NMR of ethyl (*S*)-3-(benzo[*d*][1,3]dioxol-5-yl)-2-((*tert*-butoxycarbonyl)(methyl)amino) propanoate (9) – (126 MHz, $CDCl_3$)

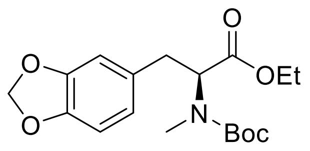

9

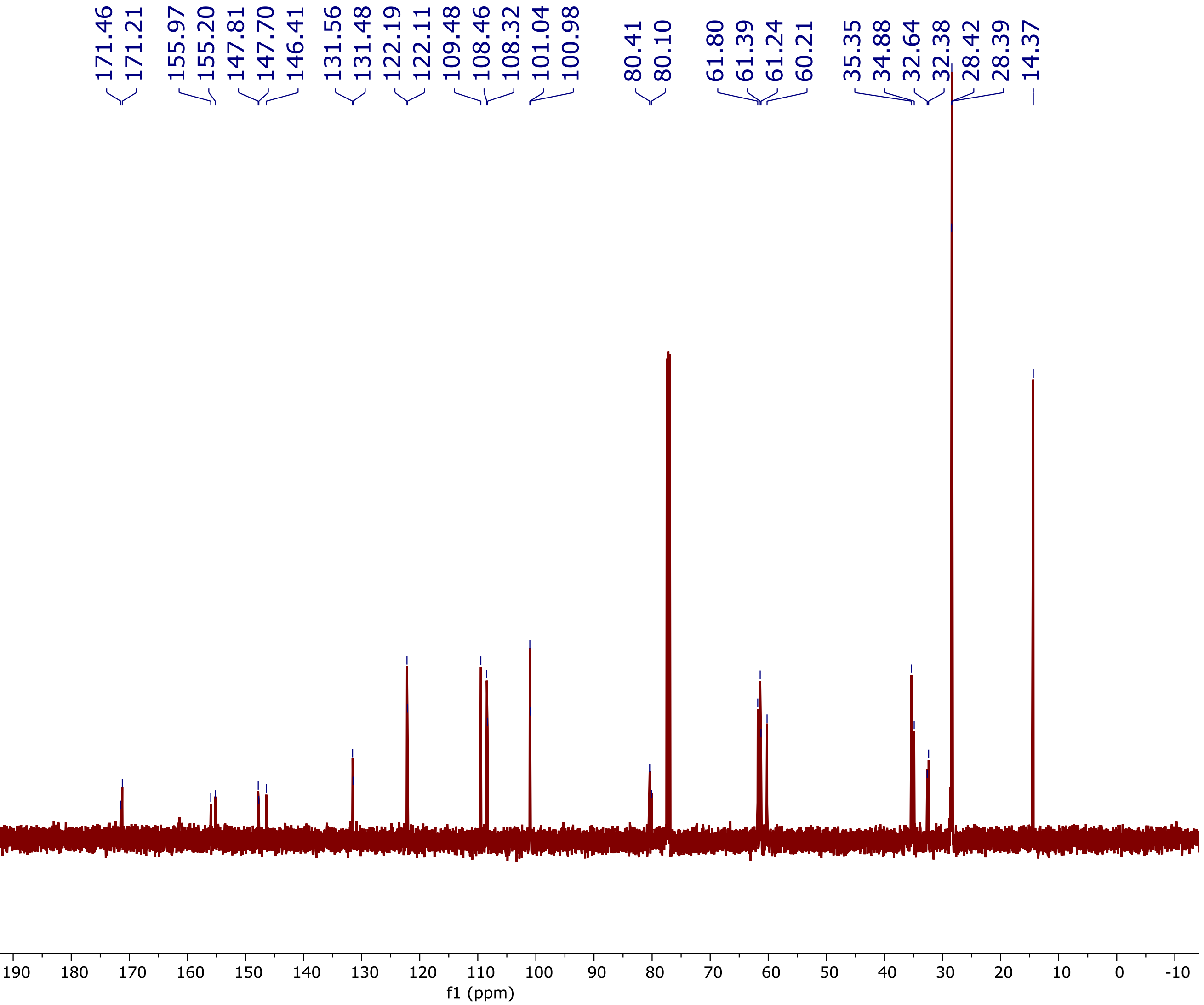

HSQC of ethyl (*S*)-3-(benzo[*d*][1,3]dioxol-5-yl)-2-((*tert*-butoxycarbonyl)(methyl)amino) propanoate (9) – ($CDCl_3$)

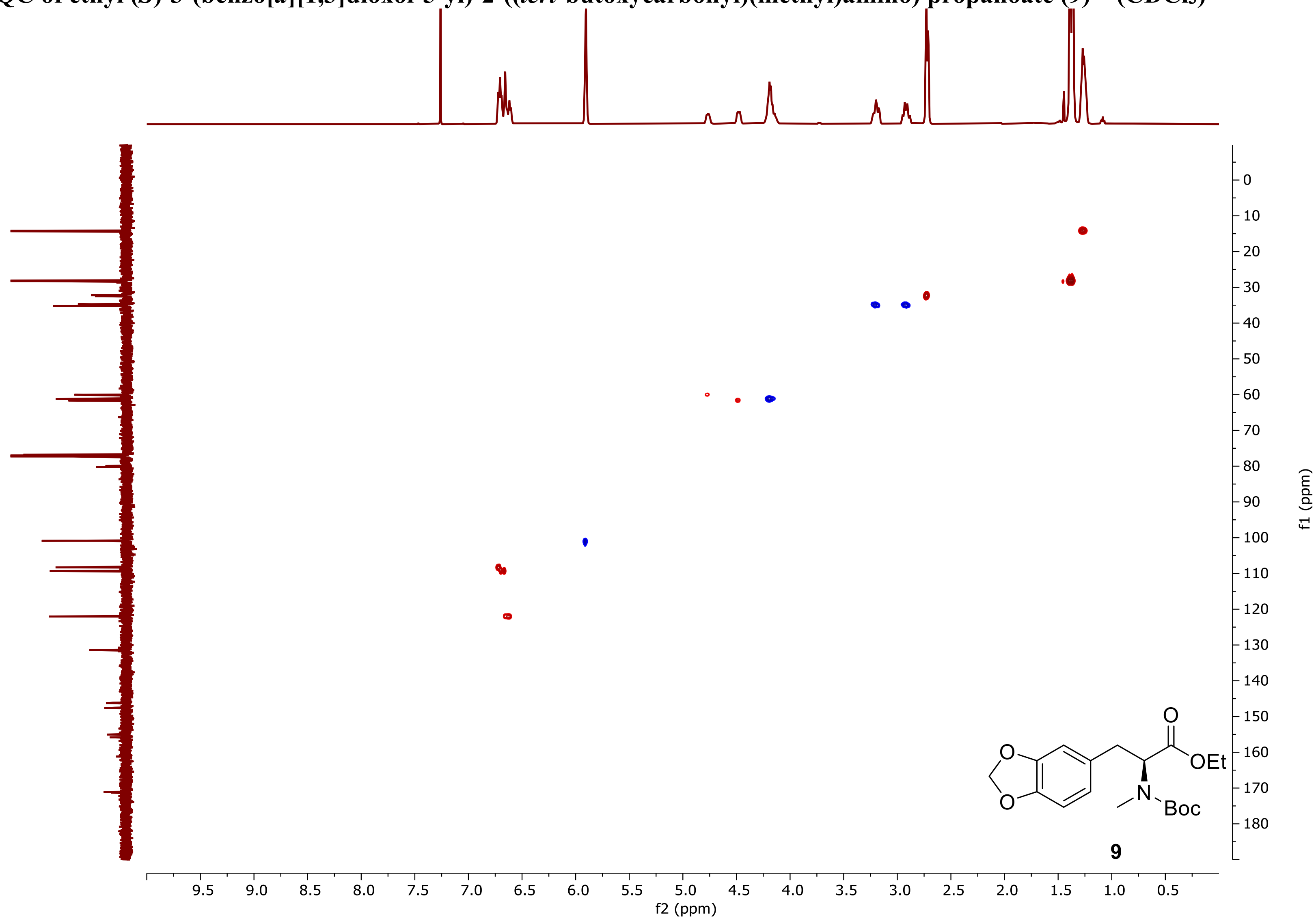

HSQC of ethyl (*S*)-3-(benzo[*d*][1,3]dioxol-5-yl)-2-((*tert*-butoxycarbonyl)(methyl)amino) propanoate (9) – ($CDCl_3$) – 5.5 to 7.5 ppm

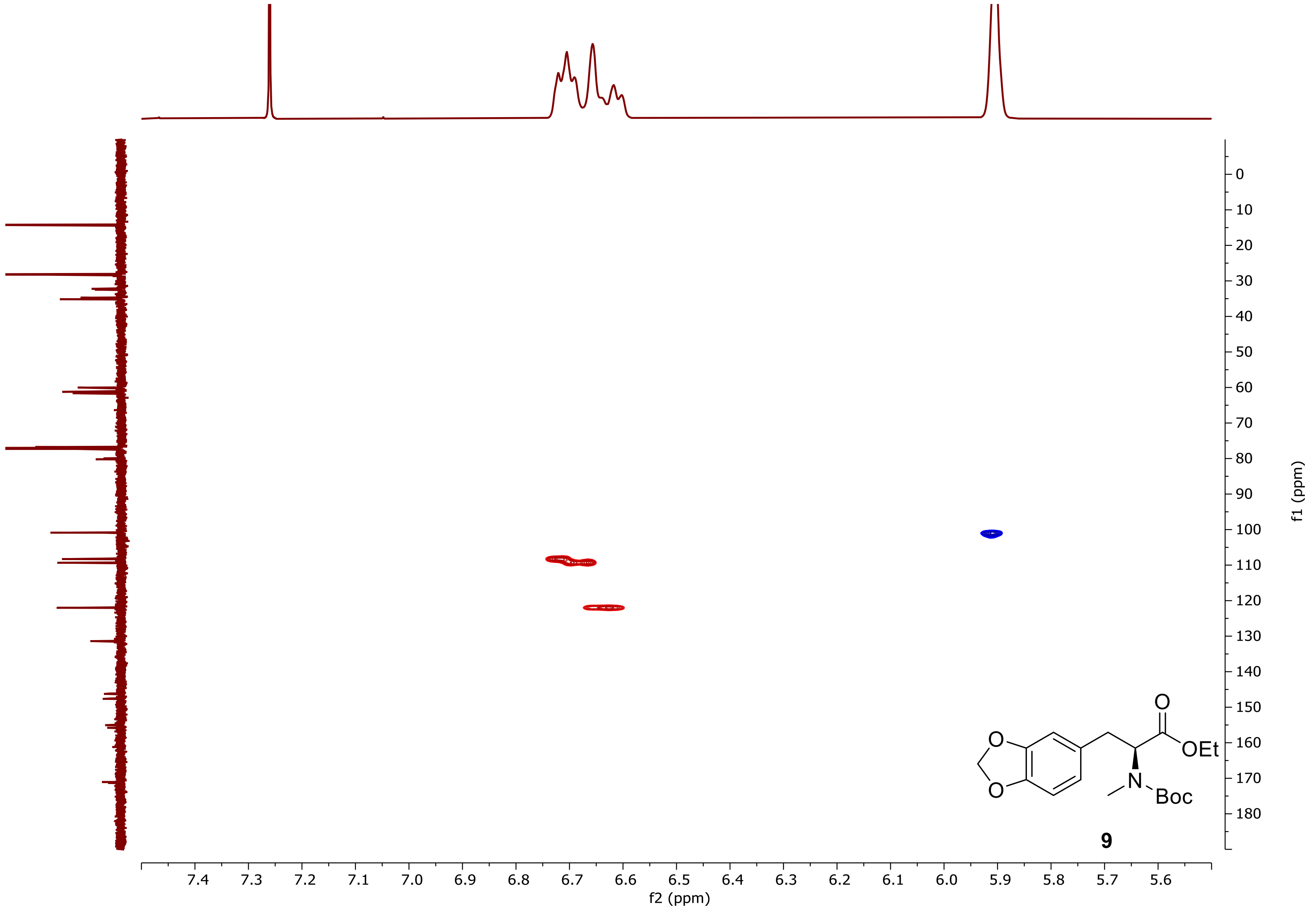

HMBC of ethyl (*S*)-3-(benzo[*d*][1,3]dioxol-5-yl)-2-((*tert*-butoxycarbonyl)(methyl)amino) propanoate (9) – (CDCl$_3$)

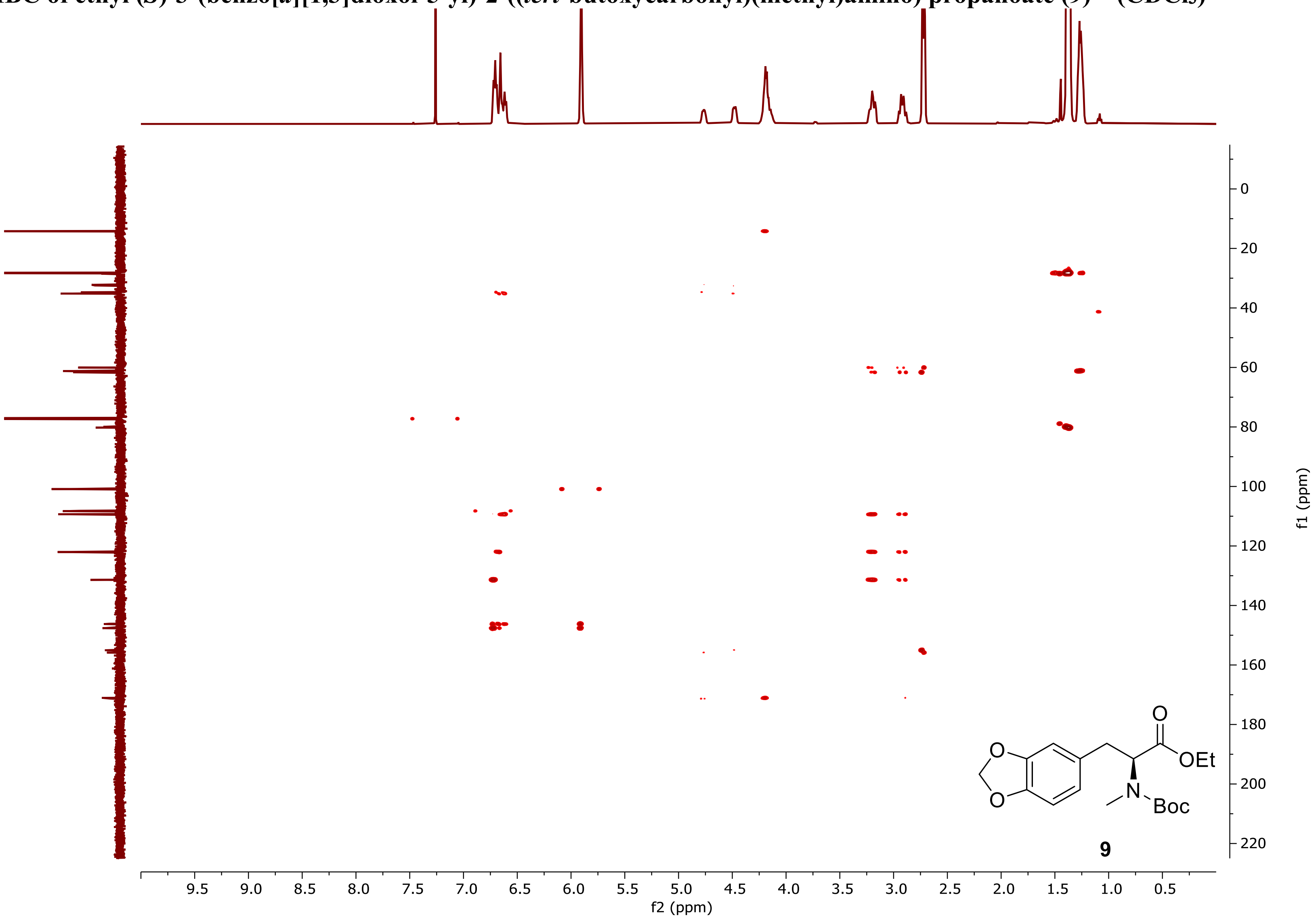

HMBC of ethyl (*S*)-3-(benzo[*d*][1,3]dioxol-5-yl)-2-((*tert*-butoxycarbonyl)(methyl)amino) propanoate (9) – ($CDCl_3$) – 5.5 to 7.5 ppm

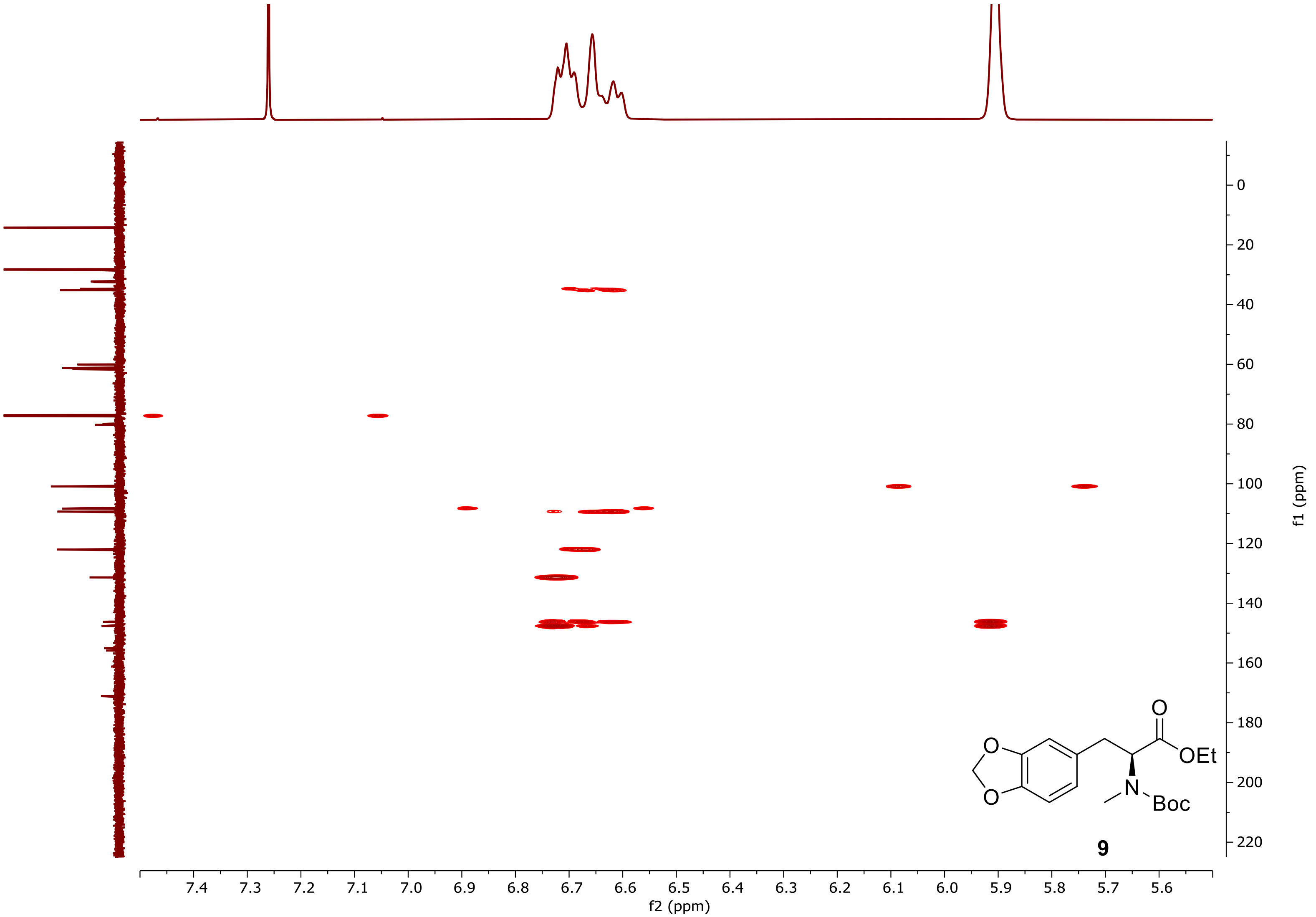

Variable Temperature 1H-NMR of ethyl (*S*)-3-(benzo[*d*][1,3]dioxol-5-yl)-2-((*tert*-butoxycarbonyl)(methyl)amino) propanoate (9) – (126 MHz, Benzene-*d*6)

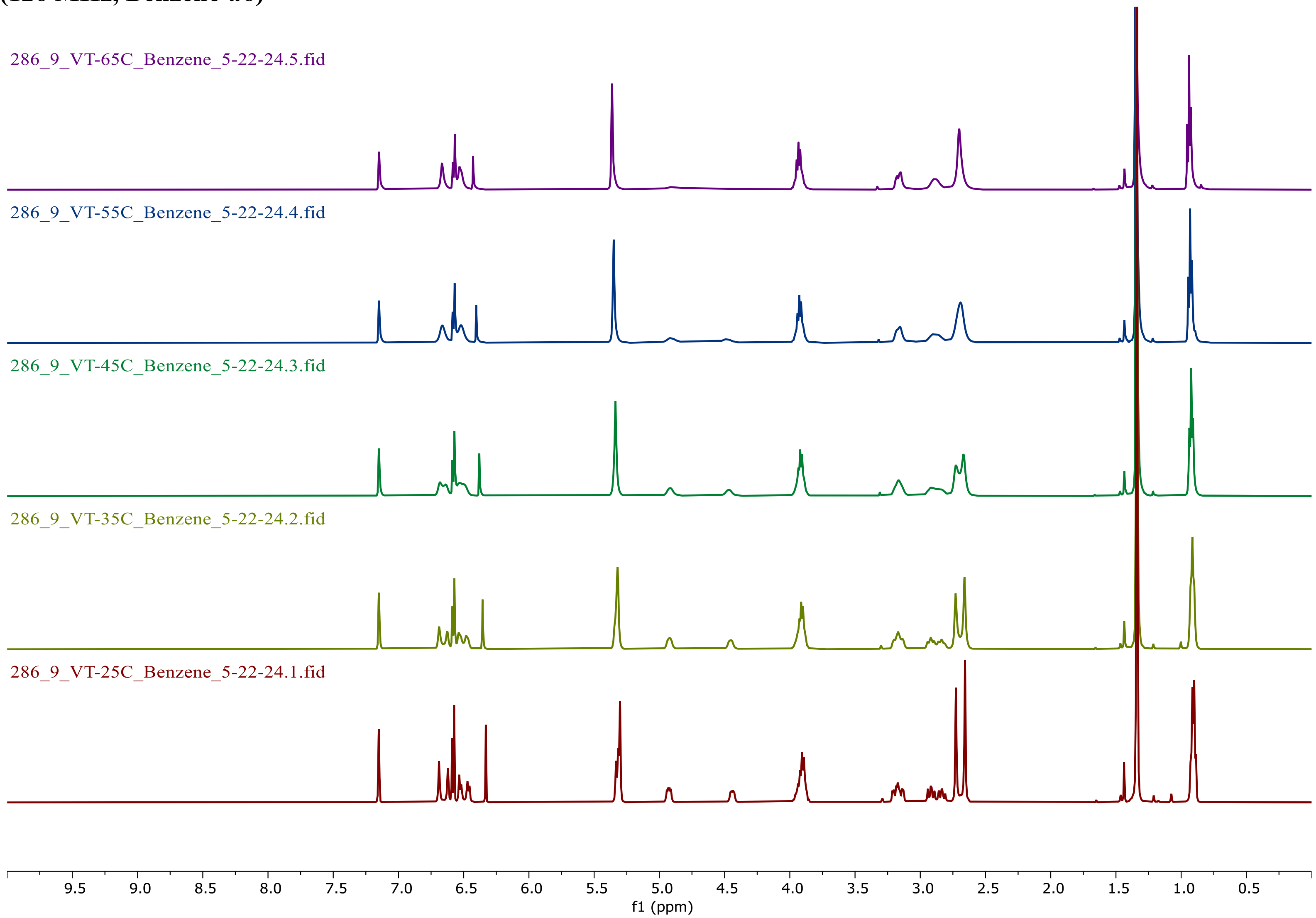

^{1}H-NMR of ethyl (*S*)-3-(benzo[*d*][1,3]dioxol-5-yl)-2-(methylamino)propanoate (10) – (500 MHz, CDCl₃)

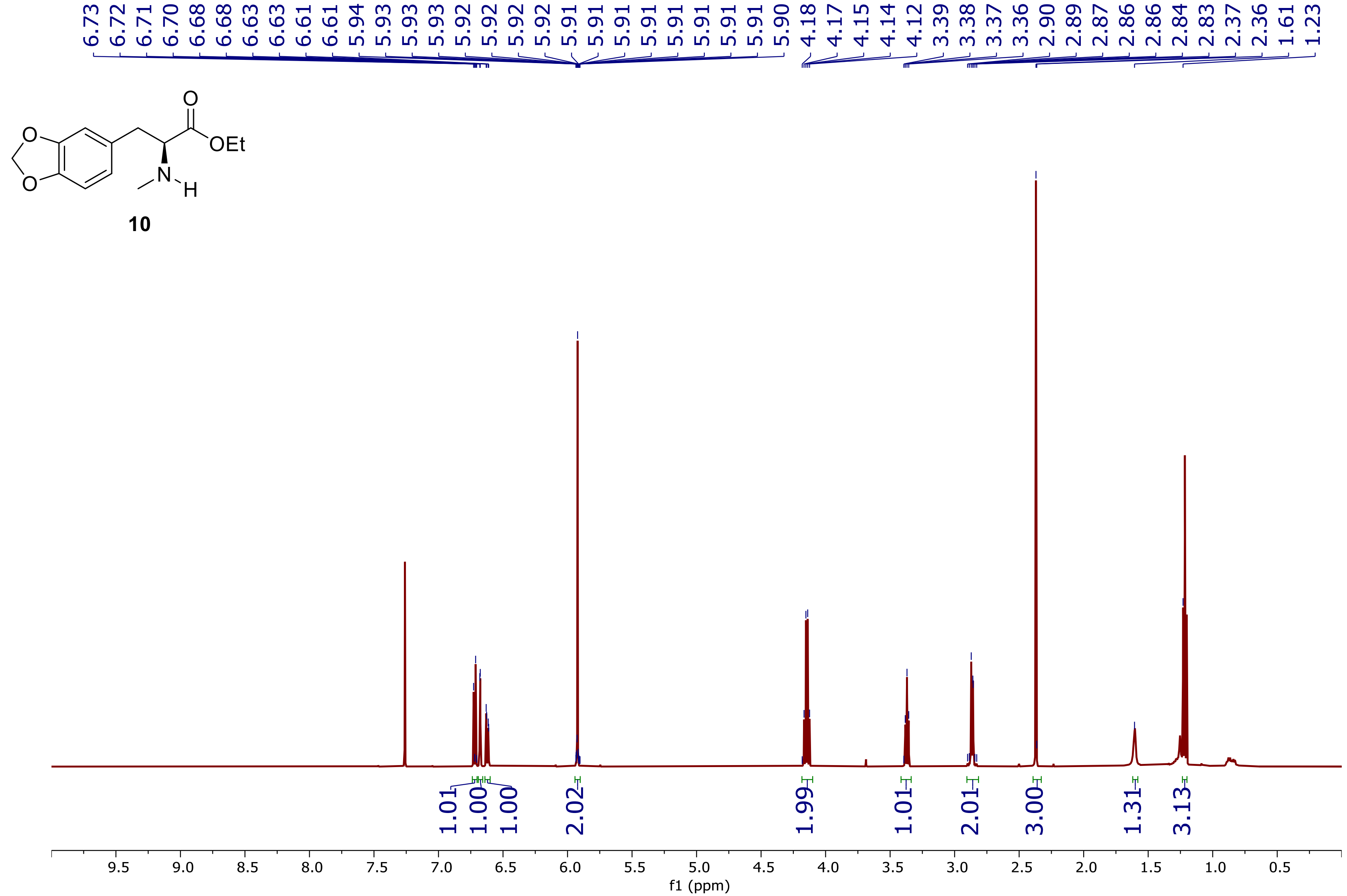

$^{13}C\{^{1}H\}$-NMR of ethyl (*S*)-3-(benzo[*d*][1,3]dioxol-5-yl)-2-(methylamino)propanoate (10) – (126 MHz, $CDCl_3$)

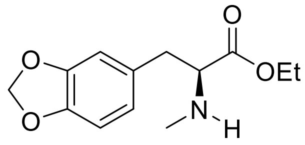

10

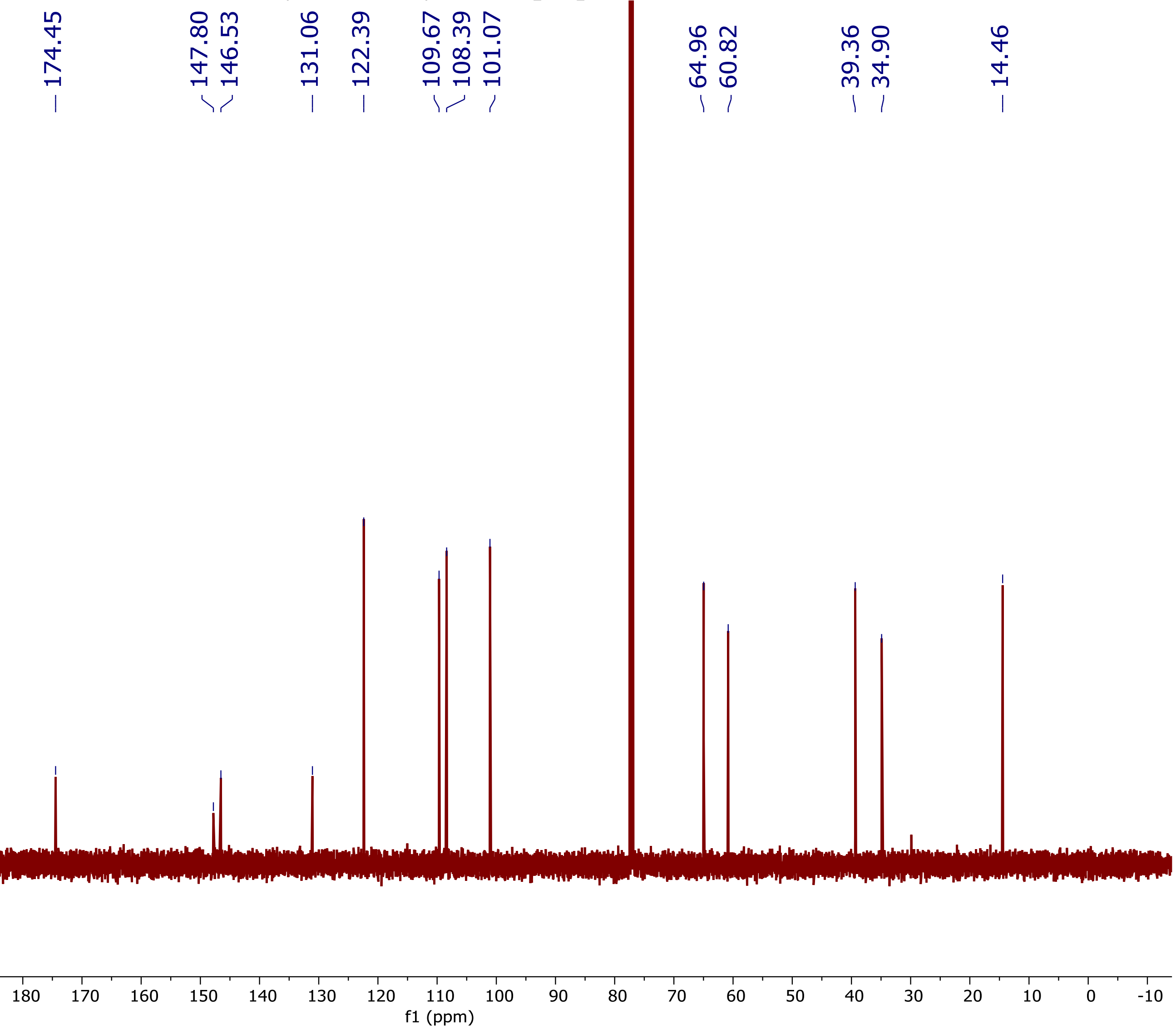

HSQC of ethyl (*S*)-3-(benzo[*d*][1,3]dioxol-5-yl)-2-(methylamino)propanoate (10) – ($CDCl_3$)

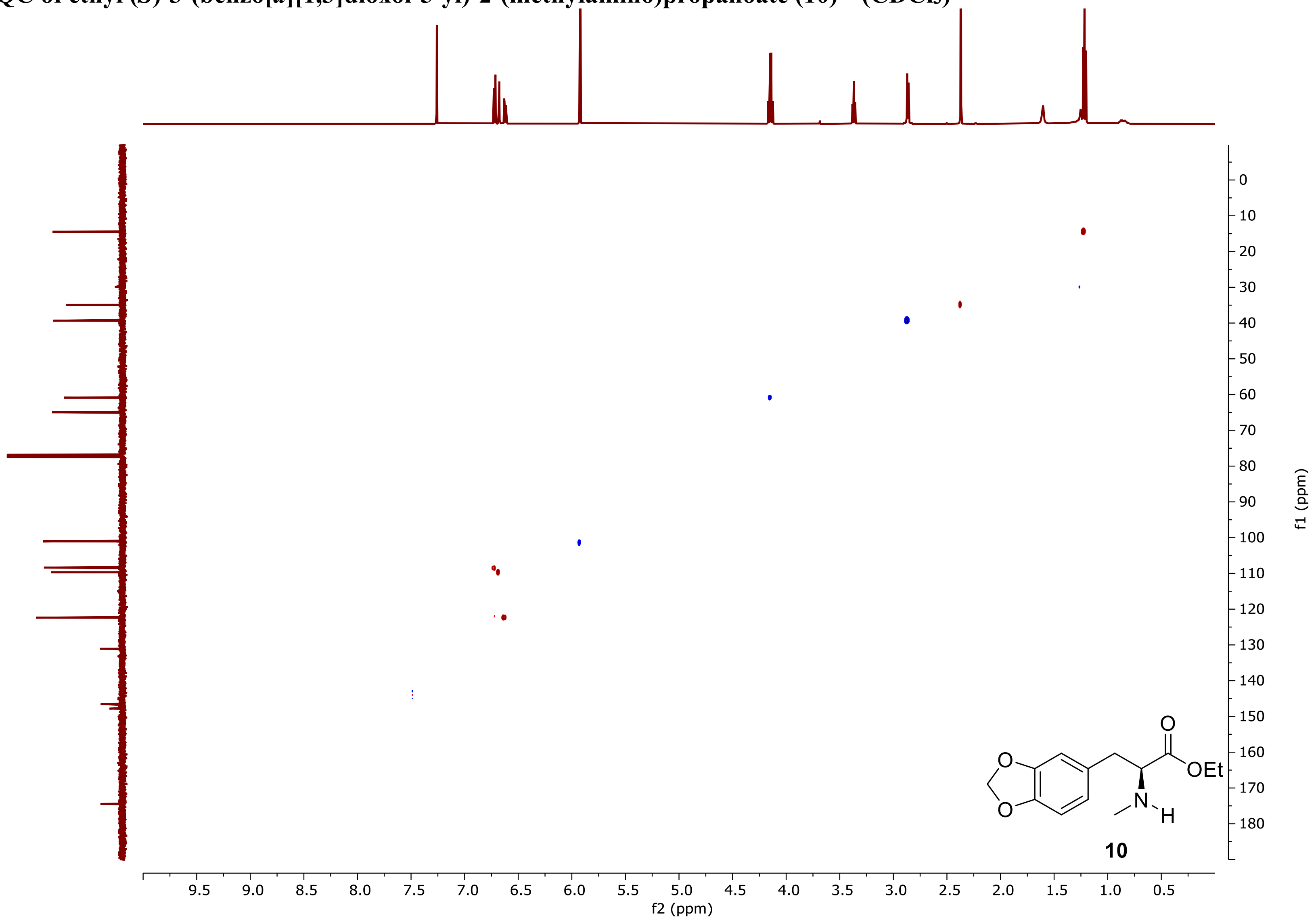

HSQC of ethyl (*S*)-3-(benzo[*d*][1,3]dioxol-5-yl)-2-(methylamino)propanoate (10) – ($CDCl_3$) – 5.5 to 7.5 ppm

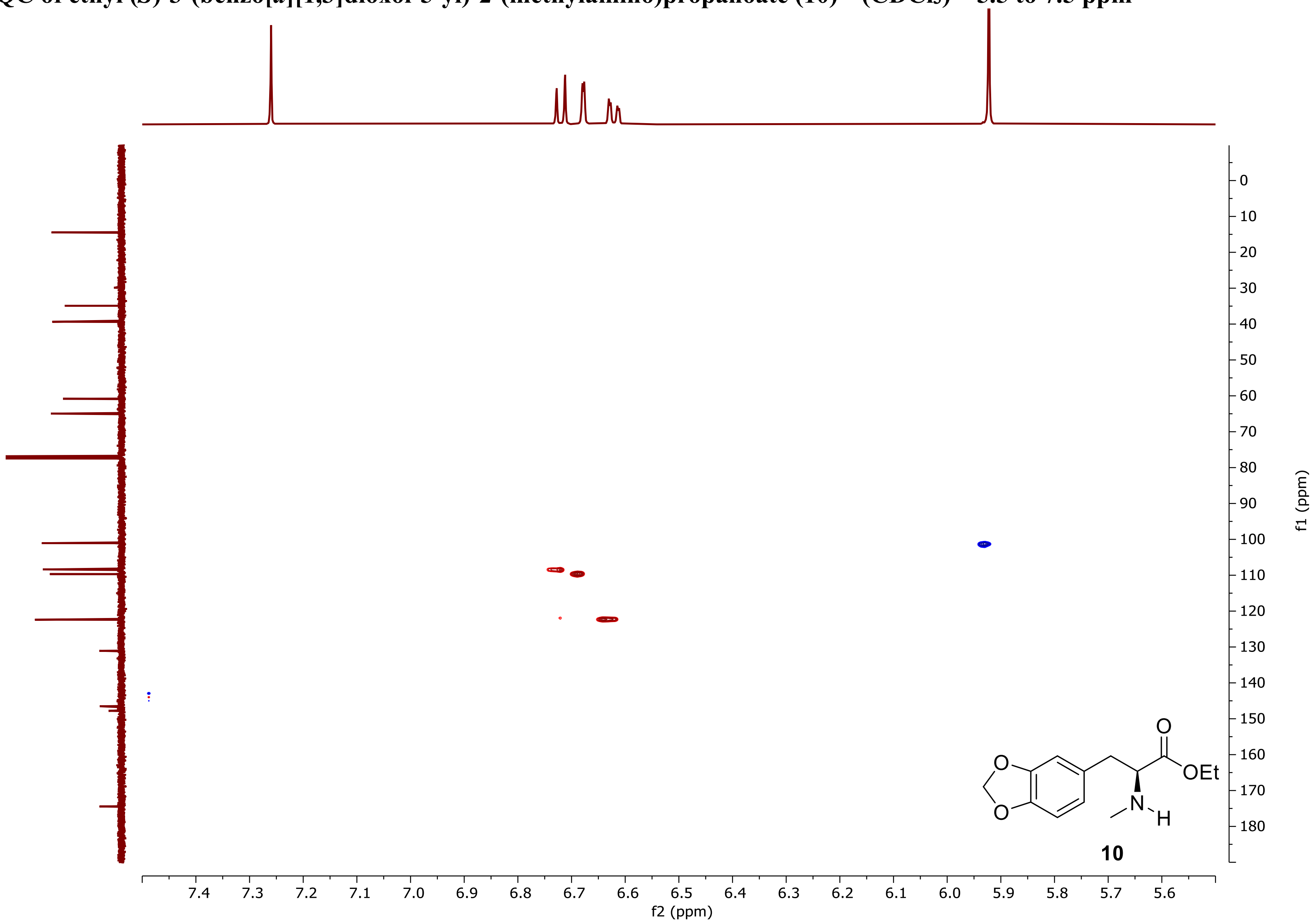

HMBC of ethyl (*S*)-3-(benzo[*d*][1,3]dioxol-5-yl)-2-(methylamino)propanoate (10) – ($CDCl_3$)

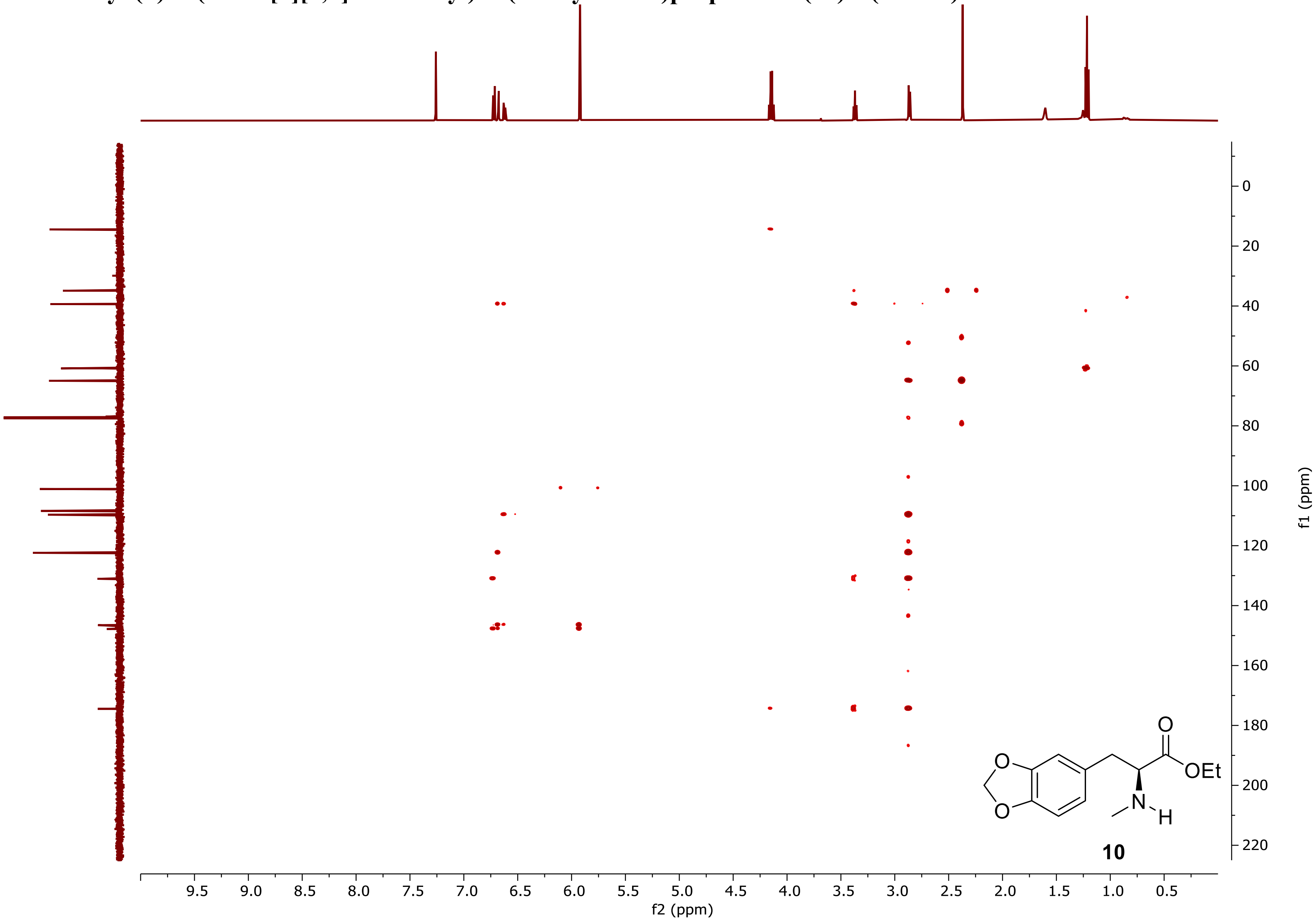

10

HMBC of ethyl (*S*)-3-(benzo[*d*][1,3]dioxol-5-yl)-2-(methylamino)propanoate (10) – ($CDCl_3$) – 5.5 to 7.5 ppm

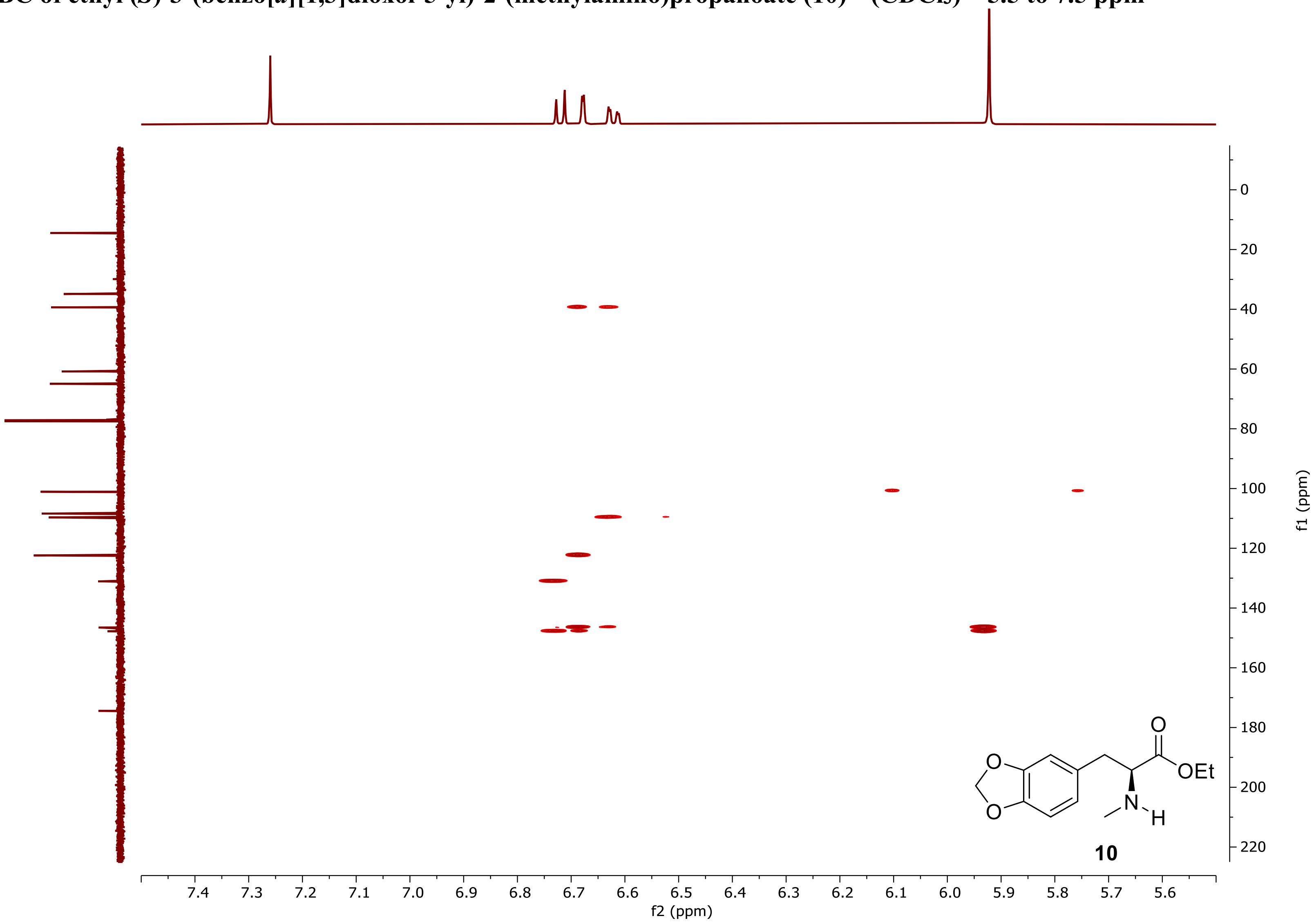

^{1}H-NMR of ethyl (*S*)-2-((*tert*-butoxycarbonyl)amino)-3-(2-(4-methoxyphenyl) benzo[*d*][1,3,2]dioxaborol-5-yl)propanoate (11) – (500 MHz, $CDCl_3$)

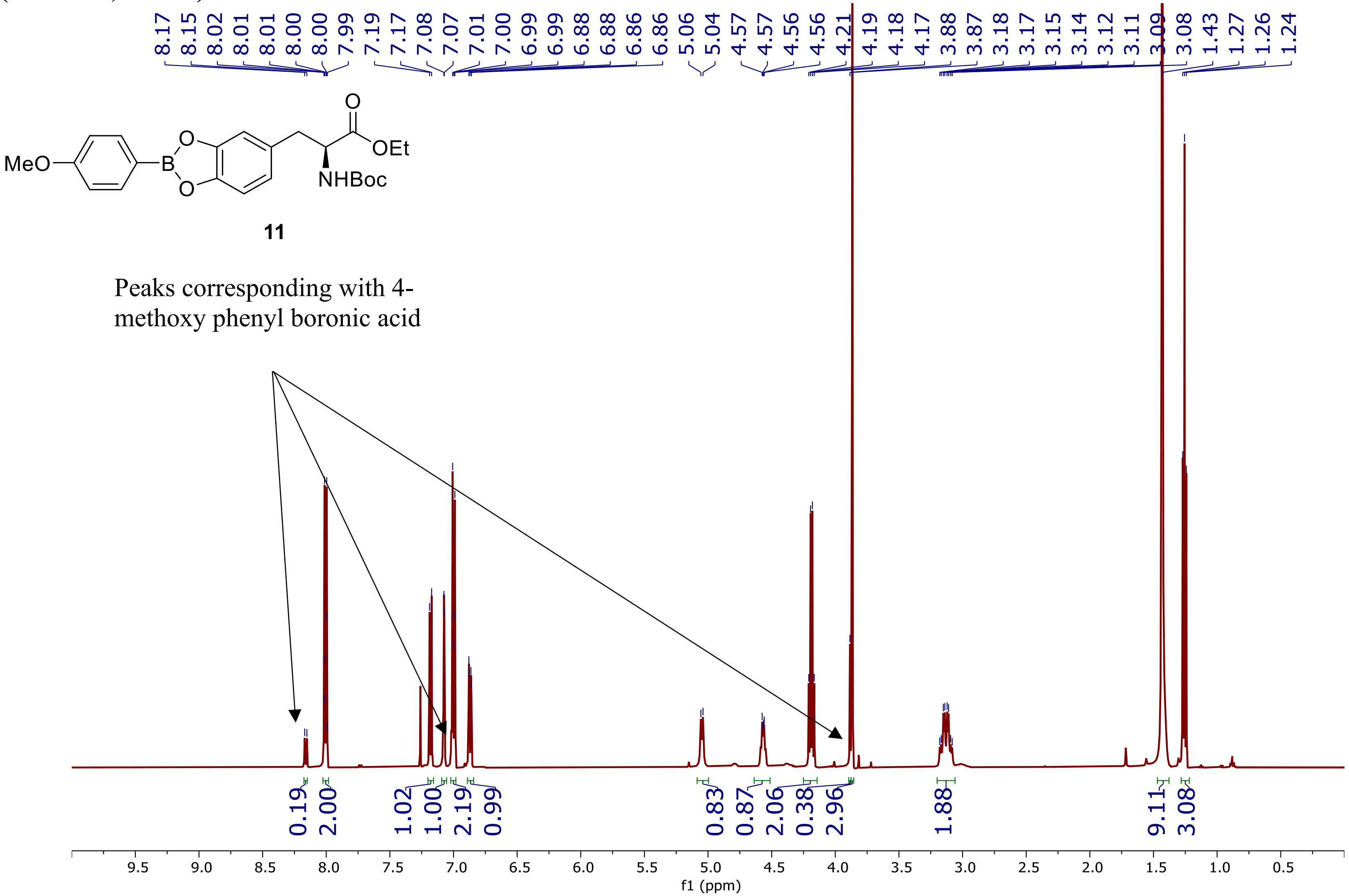

$^{13}C\{^1H\}$-NMR of ethyl (*S*)-2-((*tert*-butoxycarbonyl)amino)-3-(2-(4-methoxyphenyl) benzo[*d*][1,3,2]dioxaborol-5-yl)propanoate (11) – (126 MHz, $CDCl_3$)

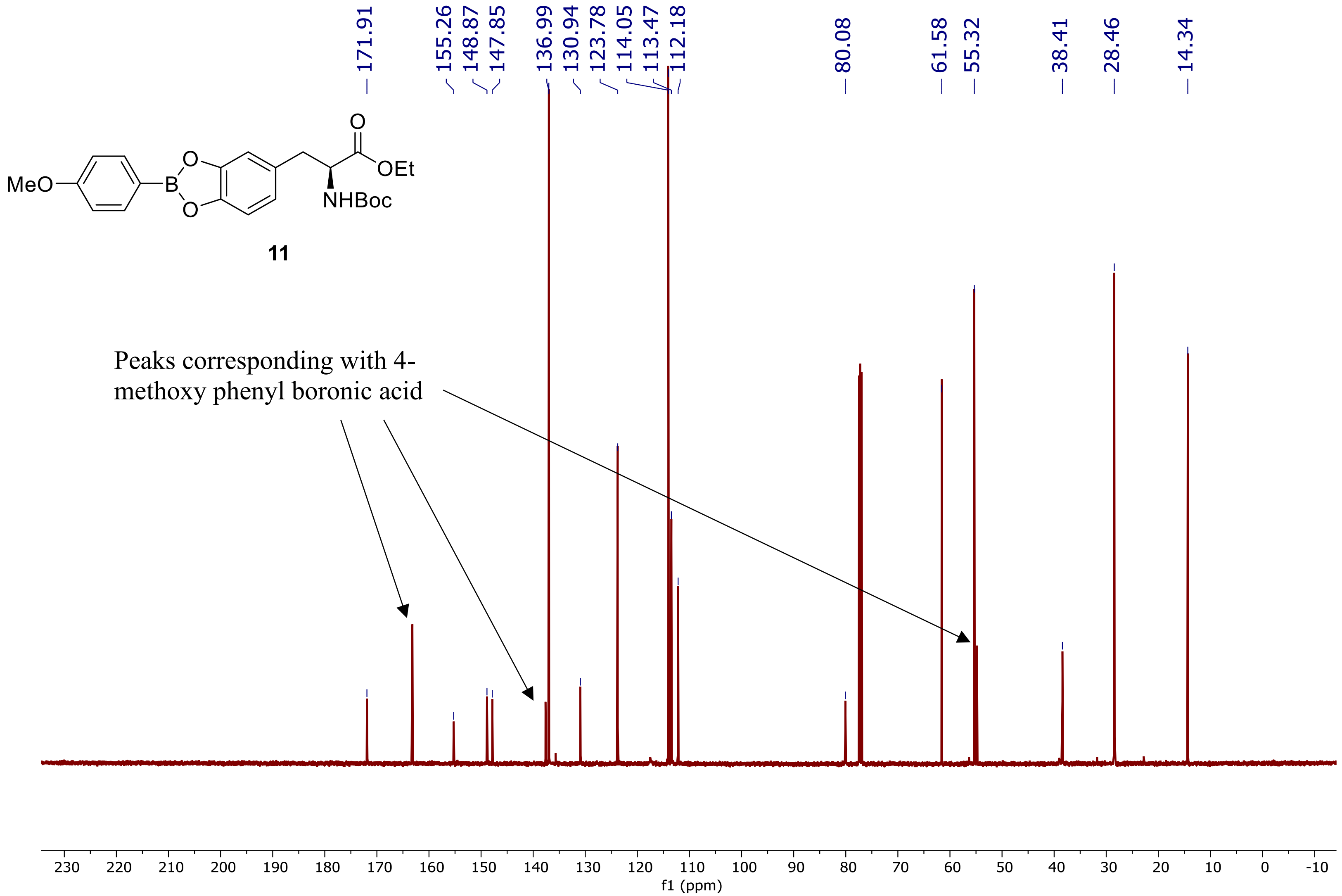

^{11}B-NMR of ethyl (*S*)-2-((*tert*-butoxycarbonyl)amino)-3-(2-(4-methoxyphenyl) benzo[*d*][1,3,2]dioxaborol-5-yl)propanoate (11) – (160 MHz, $CDCl_3$)

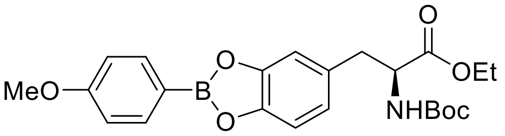

11

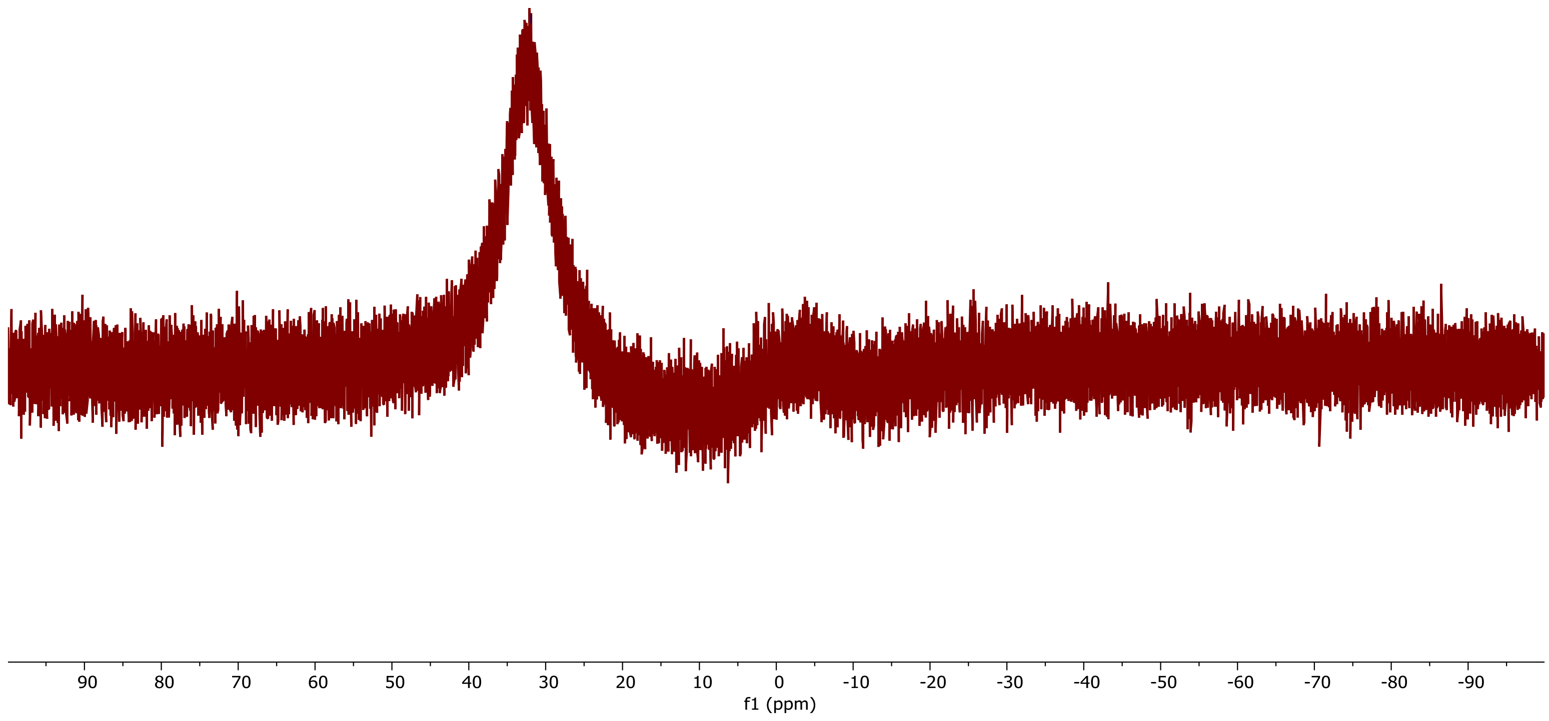

HSQC of ethyl (*S*)-2-((*tert*-butoxycarbonyl)amino)-3-(2-(4-methoxyphenyl) benzo[*d*][1,3,2]dioxaborol-5-yl)propanoate (11) – ($CDCl_3$)

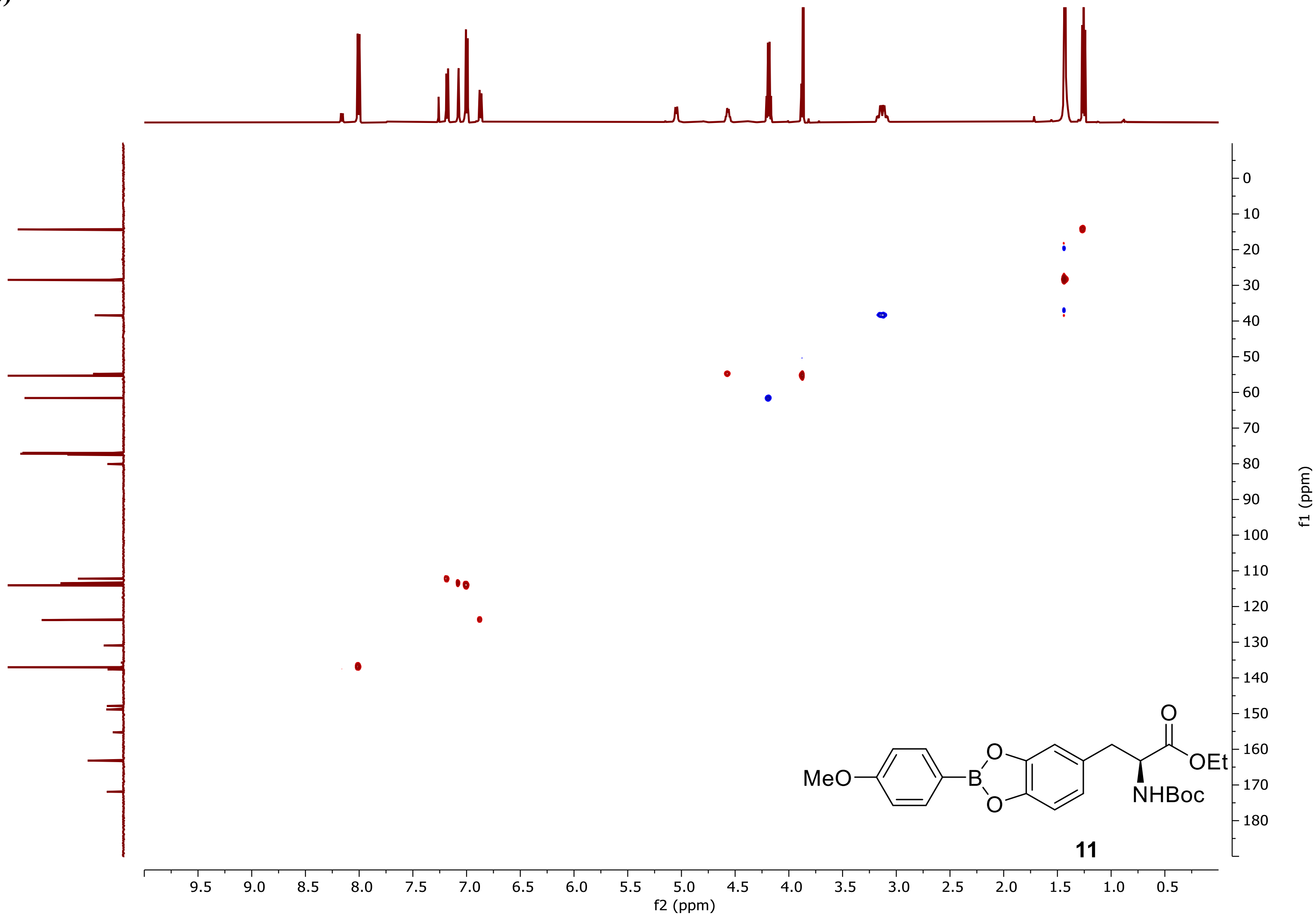

HSQC of ethyl (*S*)-2-((*tert*-butoxycarbonyl)amino)-3-(2-(4-methoxyphenyl) benzo[*d*][1,3,2]dioxaborol-5-yl)propanoate (11) – ($CDCl_3$) – 6.5 to 8.5 ppm

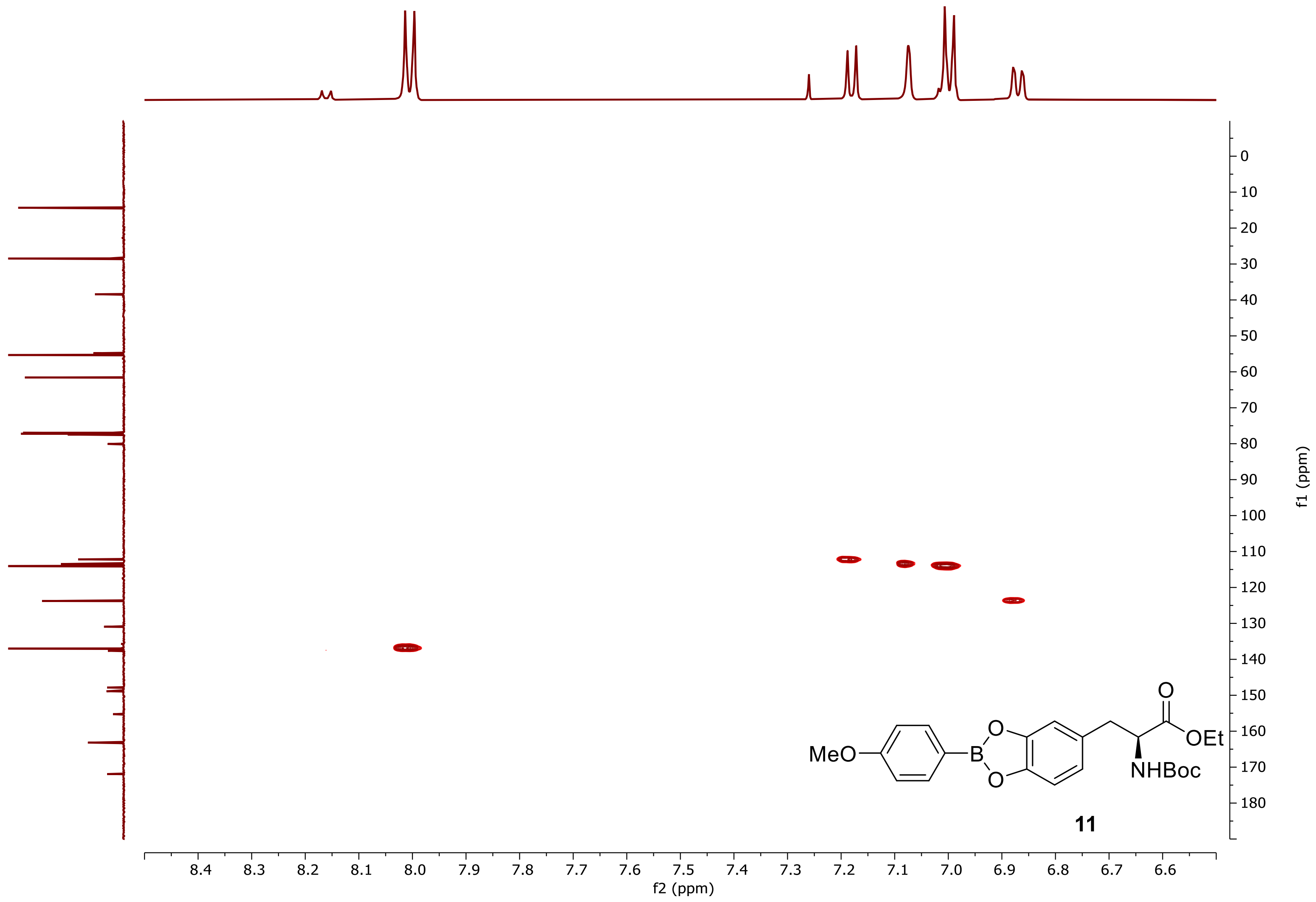

HMBC of ethyl (*S*)-2-((*tert*-butoxycarbonyl)amino)-3-(2-(4-methoxyphenyl) benzo[*d*][1,3,2]dioxaborol-5-yl)propanoate (11) – (CDCl$_3$)

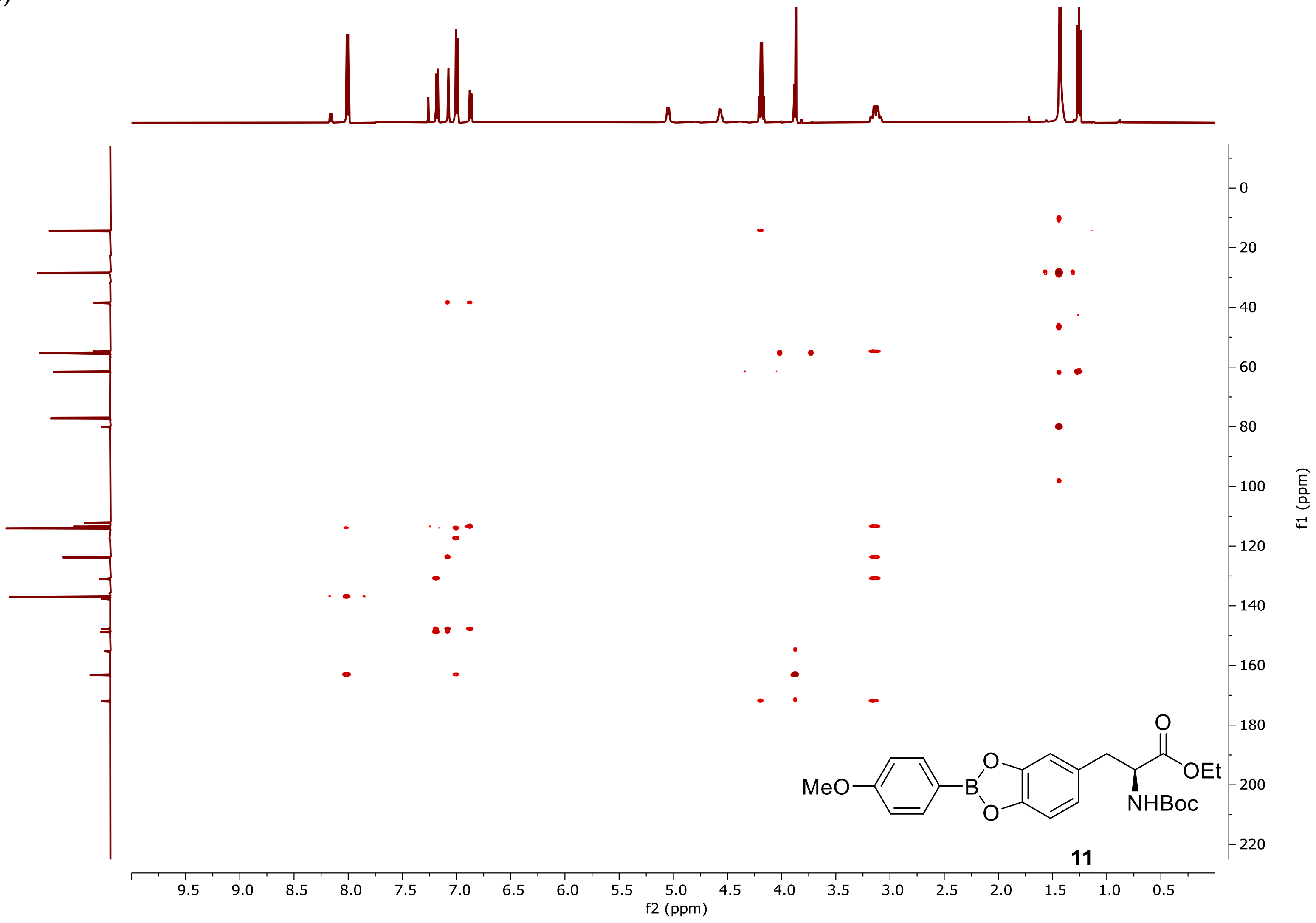

HMBC of ethyl (*S*)-2-((*tert*-butoxycarbonyl)amino)-3-(2-(4-methoxyphenyl) benzo[*d*][1,3,2]dioxaborol-5-yl)propanoate (11) – ($CDCl_3$) – 6.5 to 8.5 ppm

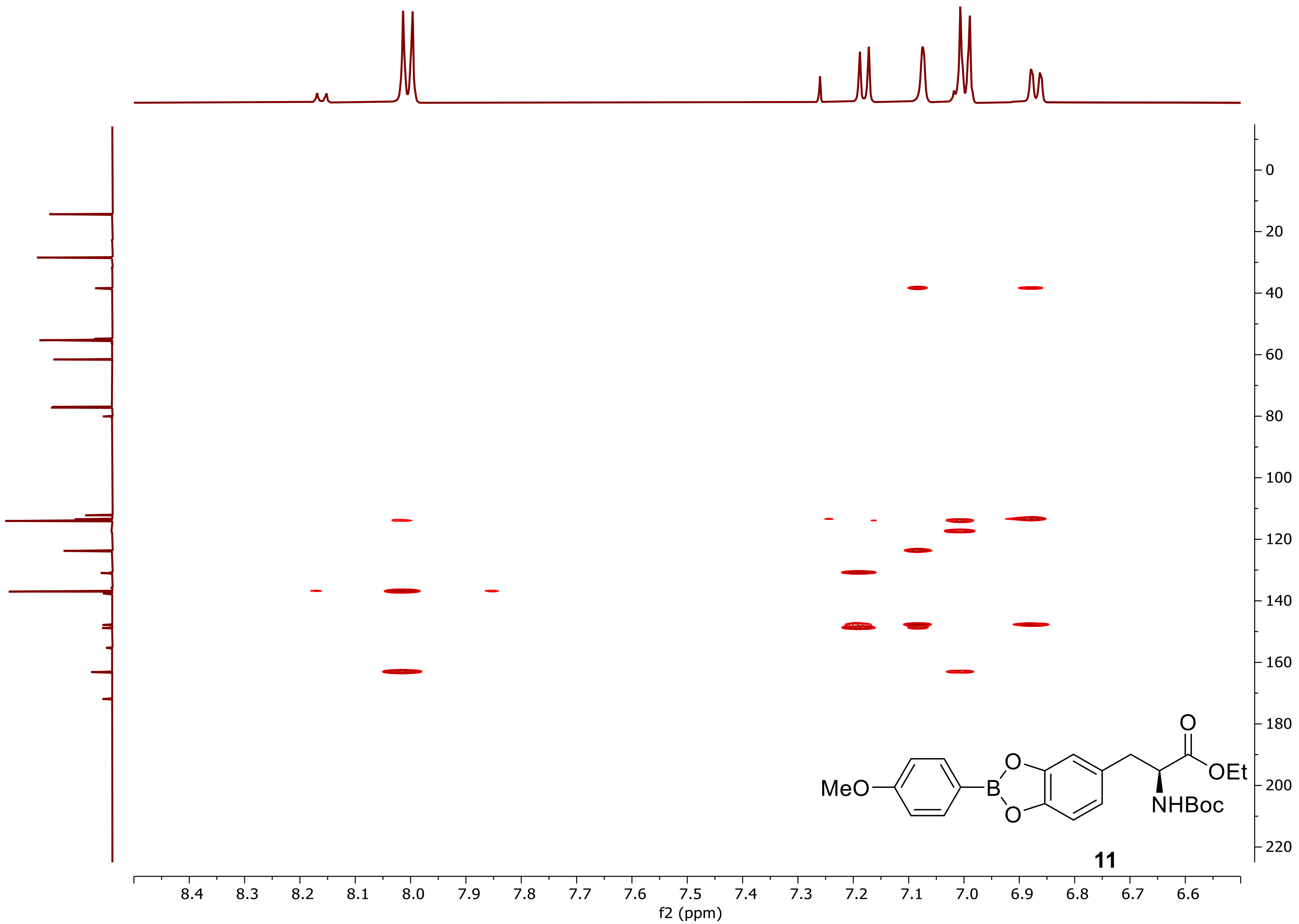

^{1}H-NMR of ethyl (*S*)-2-((*tert*-butoxycarbonyl)amino)-3-(7-(4,4,5,5-tetramethyl-1,3,2-dioxaborolan-2-yl)benzo[*d*][1,3]dioxol-5-yl)propanoate (6a) – (500 MHz, CDCl$_3$)

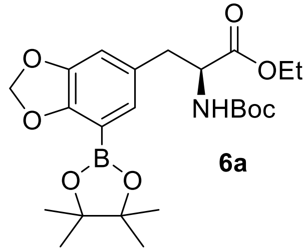

6a

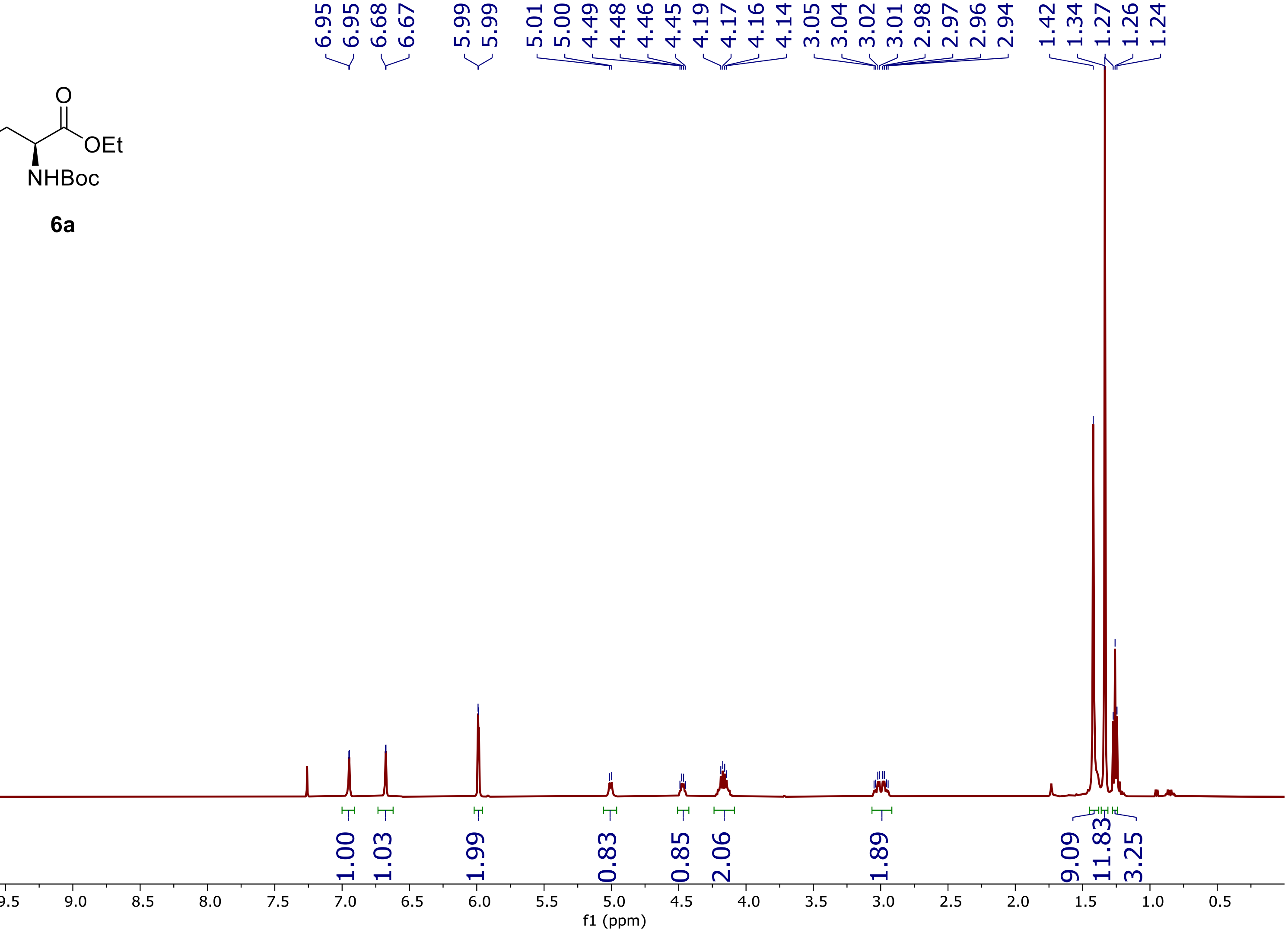

$^{13}C\{^{1}H\}$-NMR of ethyl (*S*)-2-((*tert*-butoxycarbonyl)amino)-3-(7-(4,4,5,5-tetramethyl-1,3,2-dioxaborolan-2-yl)benzo[*d*][1,3]dioxol-5-yl)propanoate (6a) – (126 MHz, $CDCl_3$)

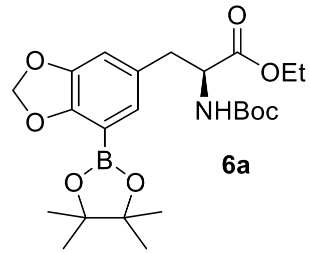

6a

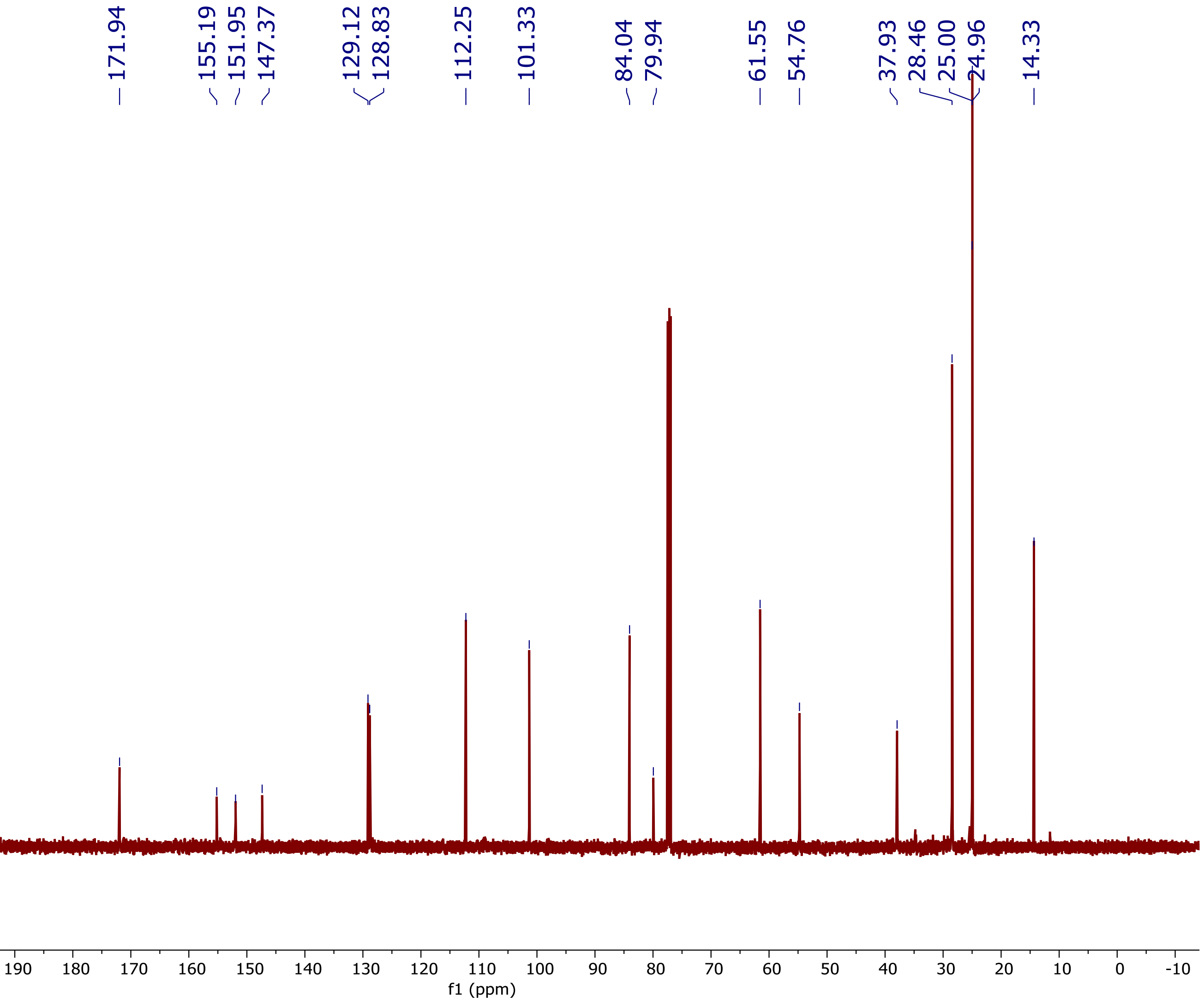

^{11}B-NMR of ethyl (*S*)-2-((*tert*-butoxycarbonyl)amino)-3-(7-(4,4,5,5-tetramethyl-1,3,2-dioxaborolan-2-yl)benzo[*d*][1,3]dioxol-5-yl)propanoate (6a) – (160 MHz, $CDCl_3$)

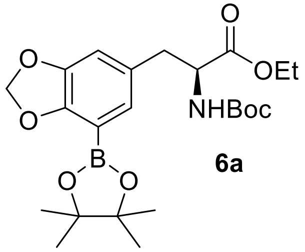

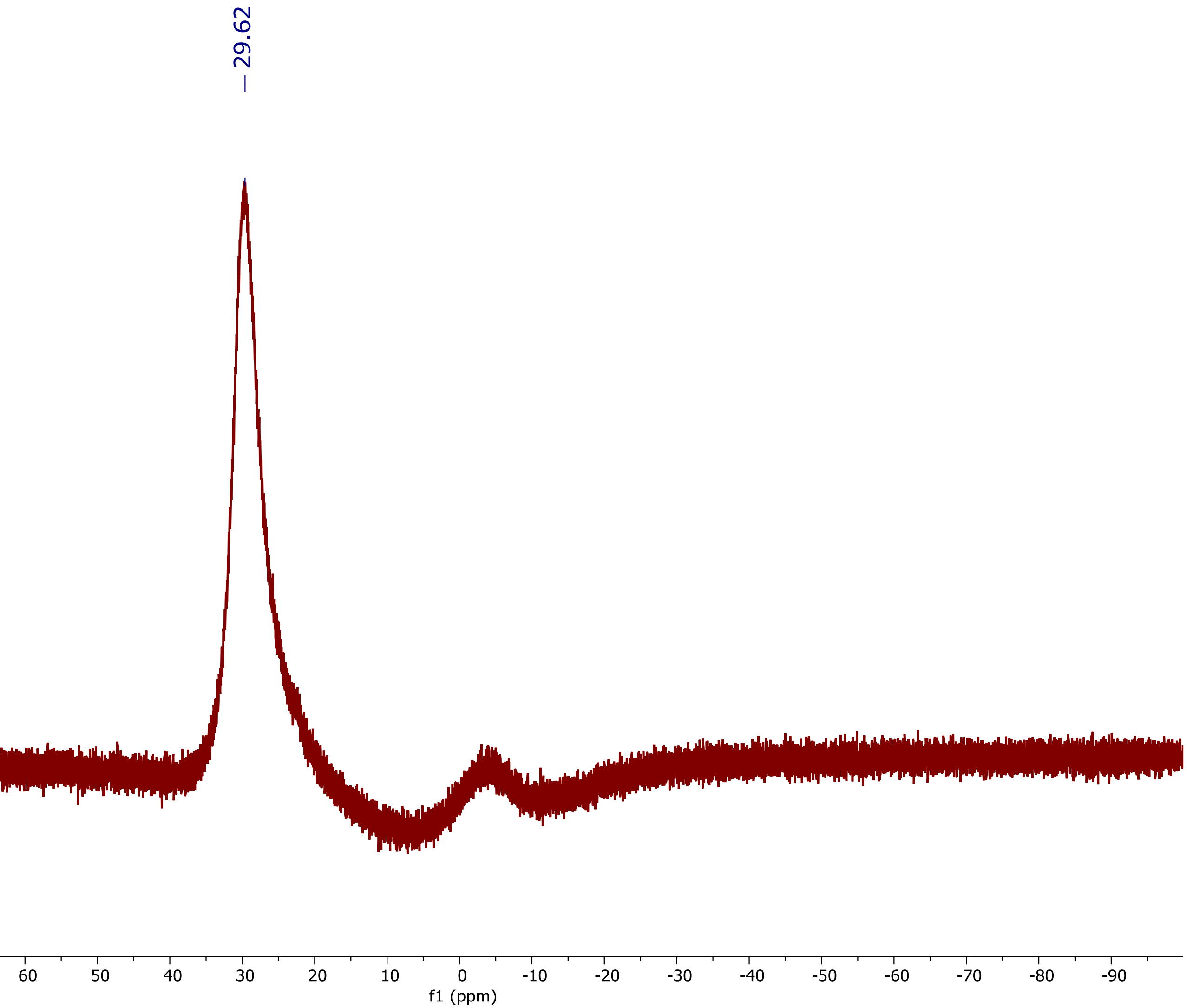

HSQC of ethyl (*S*)-2-((*tert*-butoxycarbonyl)amino)-3-(7-(4,4,5,5-tetramethyl-1,3,2-dioxaborolan-2-yl)benzo[*d*][1,3]dioxol-5-yl)propanoate (6a) – ($CDCl_3$)

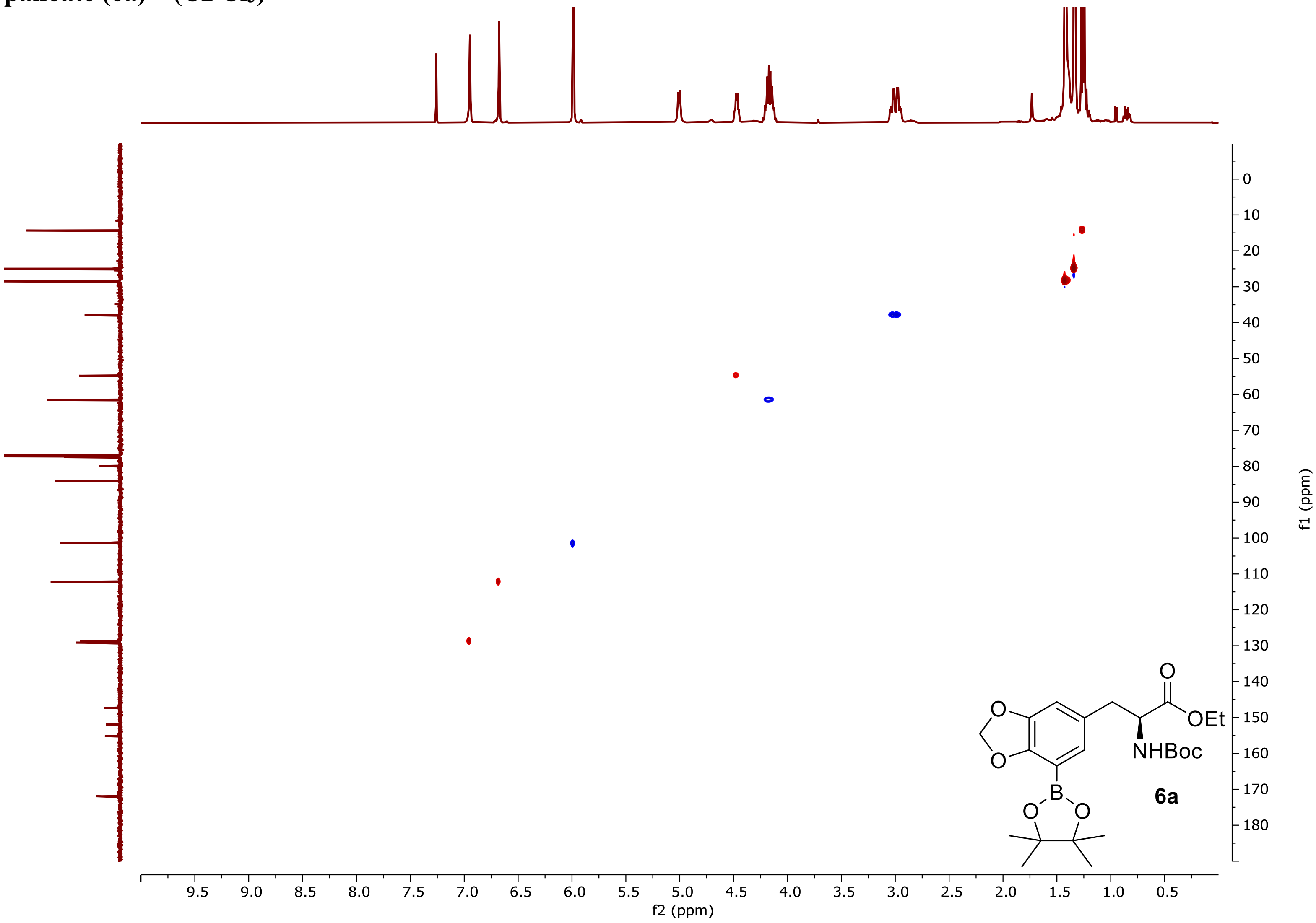

HSQC of ethyl (*S*)-2-((*tert*-butoxycarbonyl)amino)-3-(7-(4,4,5,5-tetramethyl-1,3,2-dioxaborolan-2-yl)benzo[*d*][1,3]dioxol-5-yl)propanoate (6a) – ($CDCl_3$) – 5.5 to 7.5 ppm

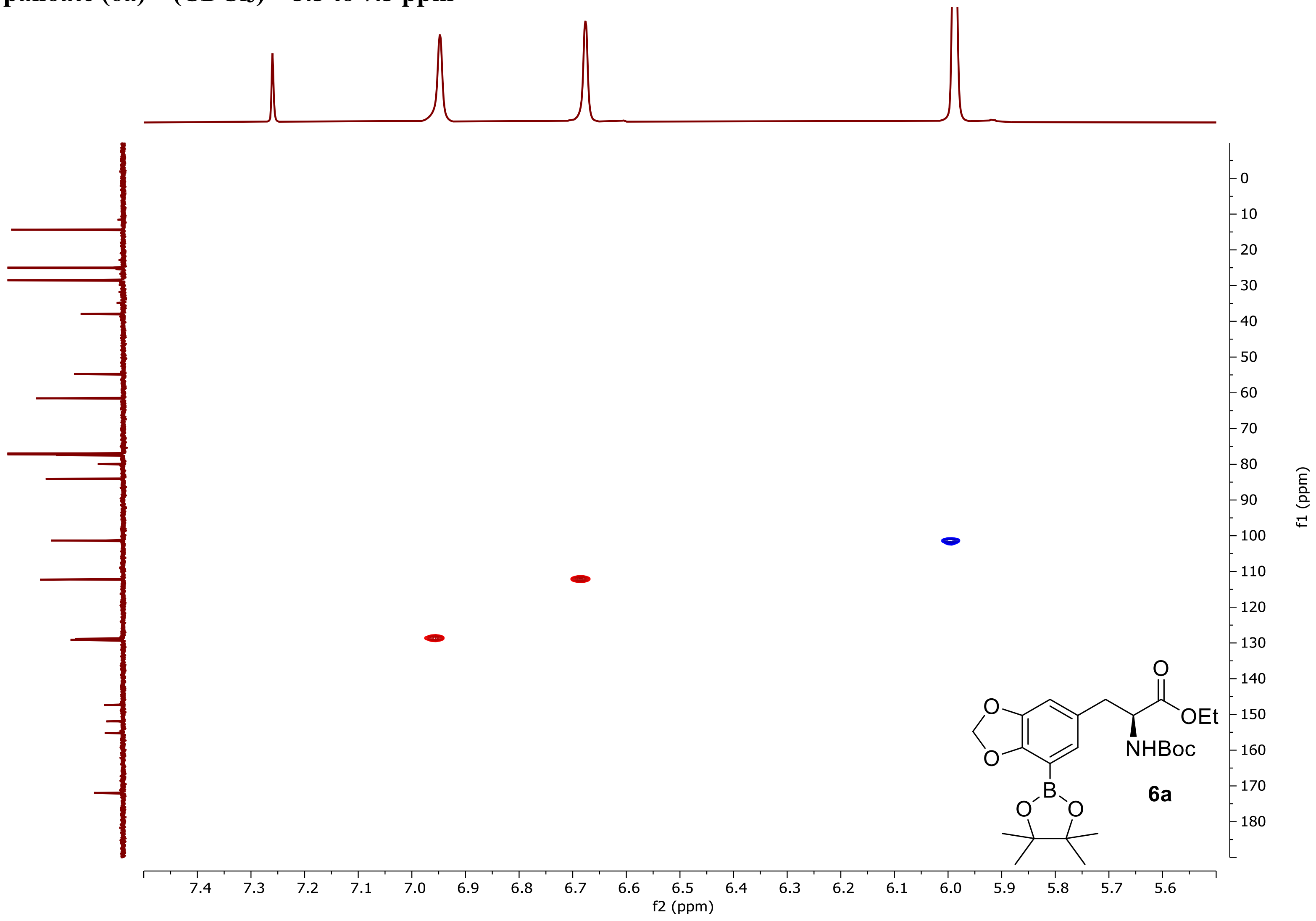

HMBC of ethyl (*S*)-2-((*tert*-butoxycarbonyl)amino)-3-(7-(4,4,5,5-tetramethyl-1,3,2-dioxaborolan-2-yl)benzo[*d*][1,3]dioxol-5-yl)propanoate (6a) – ($CDCl_3$)

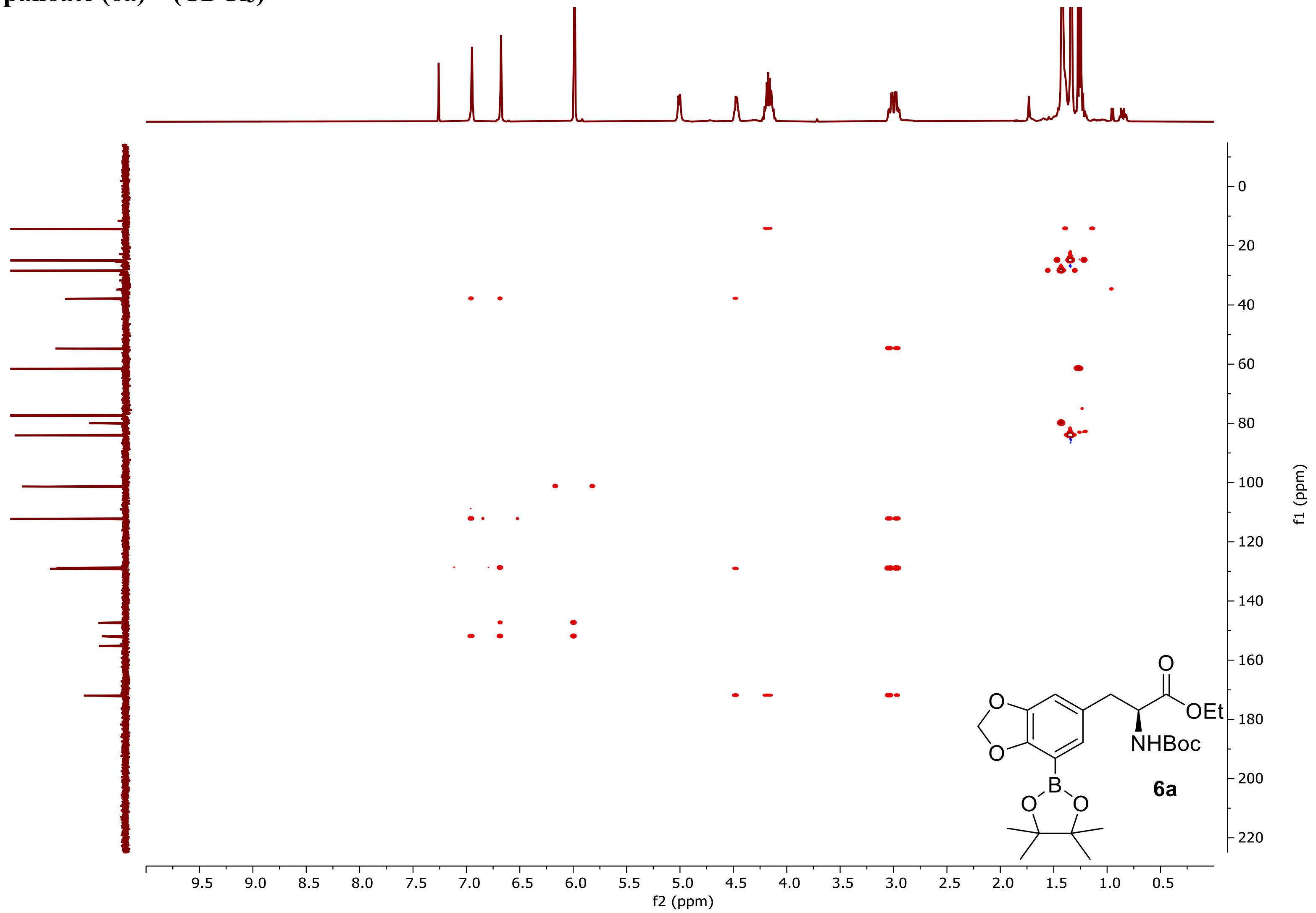

HMBC of ethyl (*S*)-2-((*tert*-butoxycarbonyl)amino)-3-(7-(4,4,5,5-tetramethyl-1,3,2-dioxaborolan-2-yl)benzo[*d*][1,3]dioxol-5-yl)propanoate (6a) – ($CDCl_3$) – 5.5 to 7.5 ppm

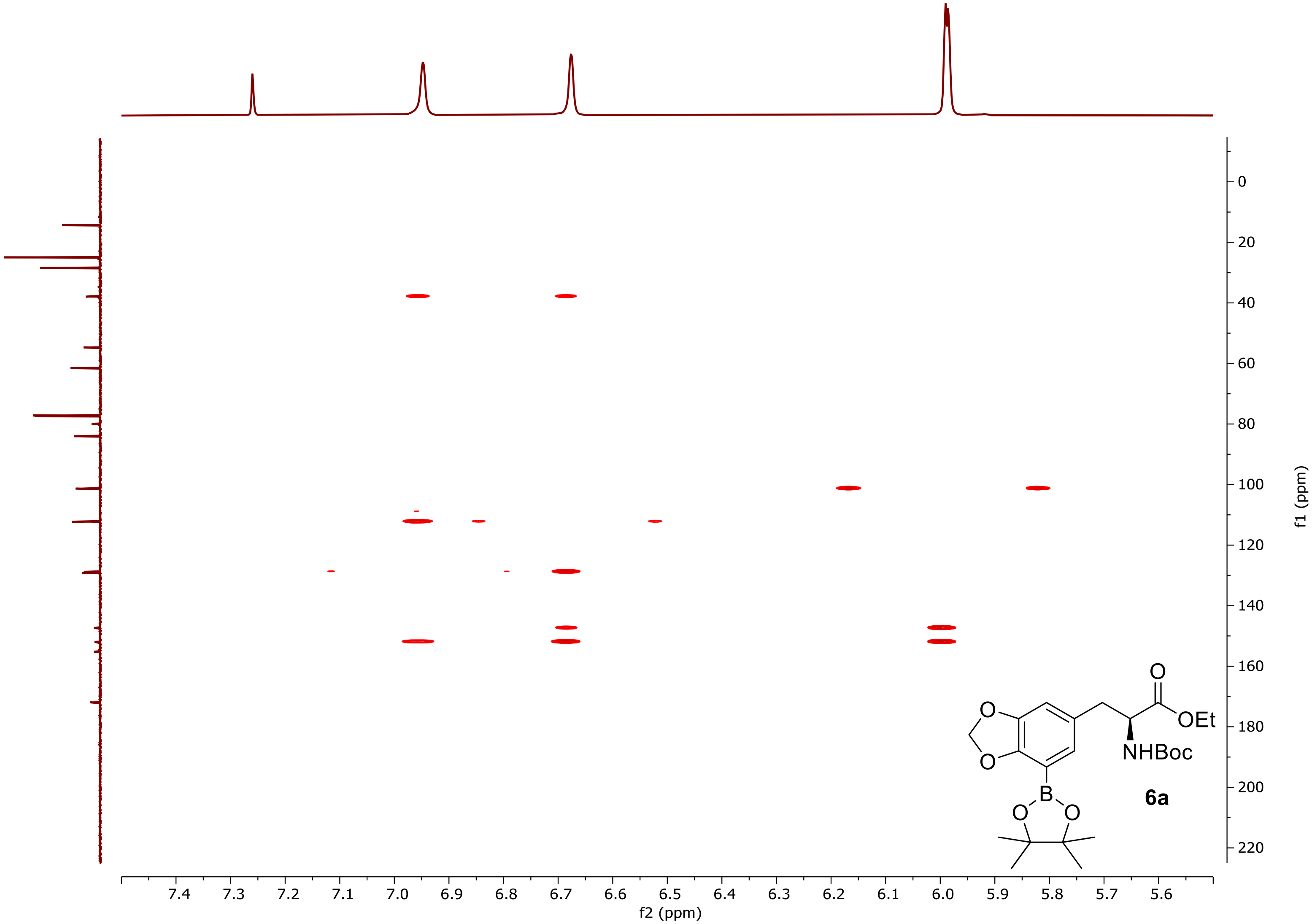

^{1}H-NMR of (*S*)-1-ethoxy-1-oxo-3-(7-(4,4,5,5-tetramethyl-1,3,2-dioxaborolan-2-yl)benzo[*d*][1,3]dioxol-5-yl)propan-2-aminium trichloroacetate (7a) – (500 MHz, $CDCl_3$)

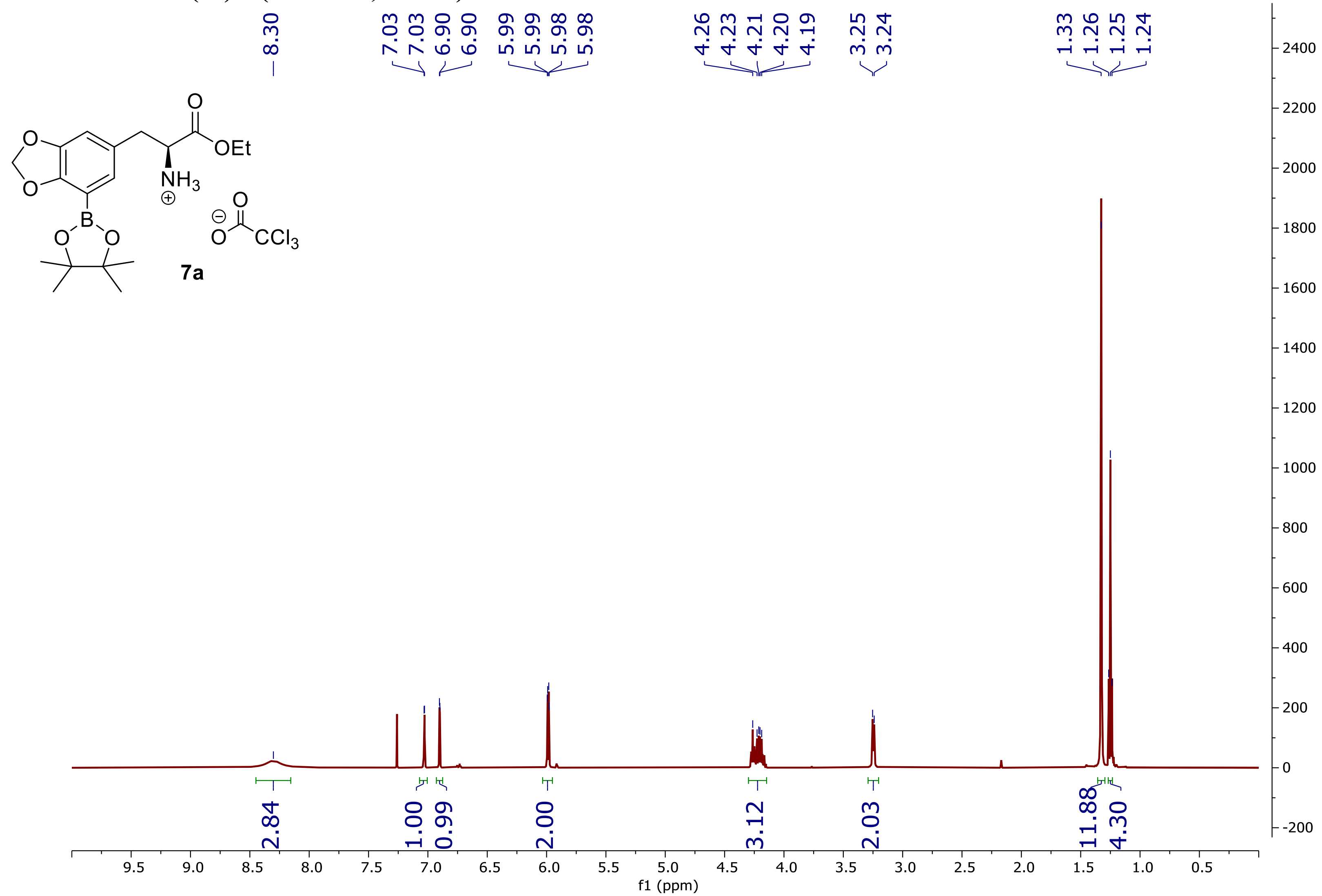

$^{13}C\{^{1}H\}$-NMR of (*S*)-1-ethoxy-1-oxo-3-(7-(4,4,5,5-tetramethyl-1,3,2-dioxaborolan-2-yl)benzo[*d*][1,3]dioxol-5-yl)propan-2-aminium trichloroacetate (7a) – (126 MHz, CDCl₃)

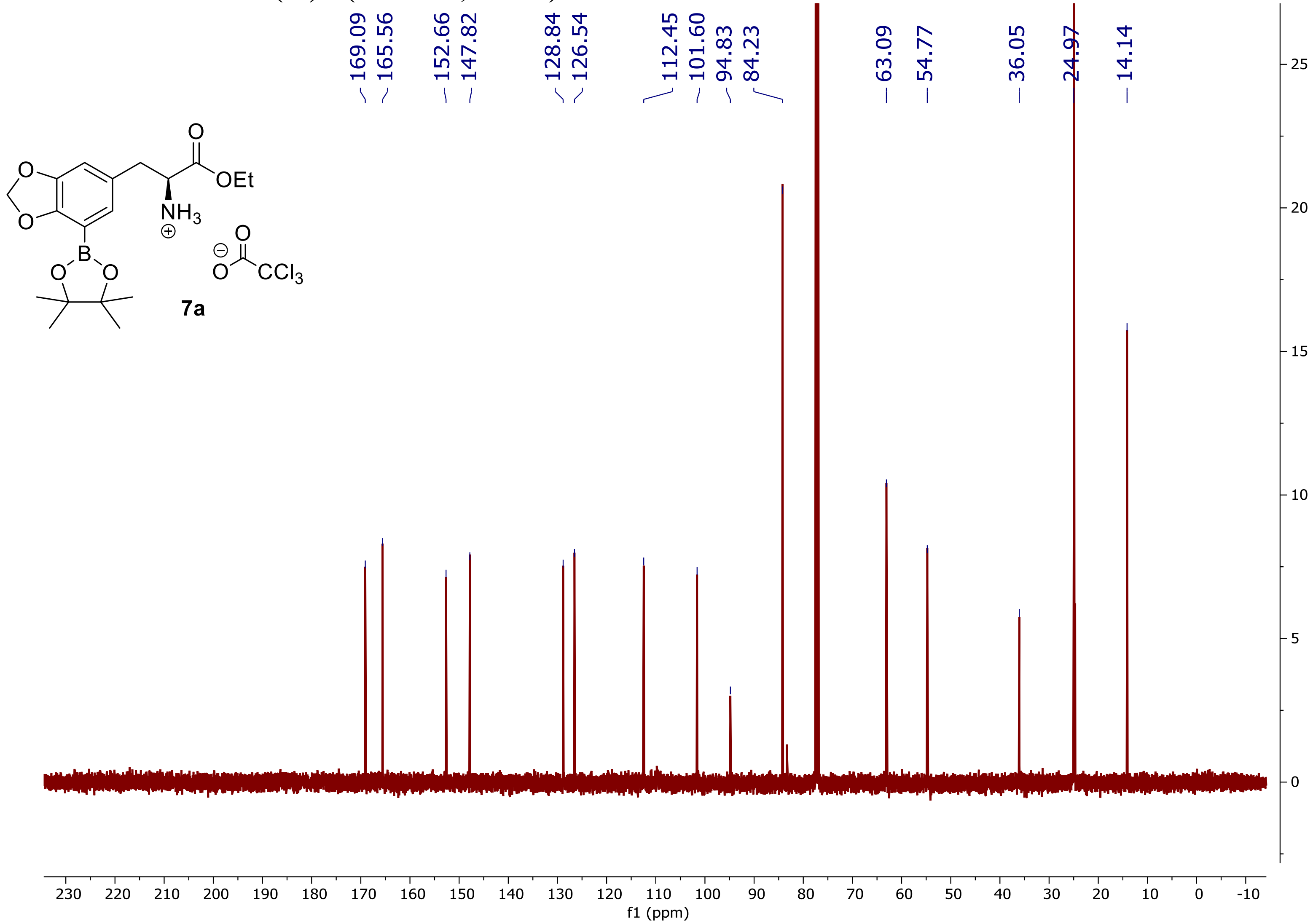

^{11}B-NMR of (*S*)-1-ethoxy-1-oxo-3-(7-(4,4,5,5-tetramethyl-1,3,2-dioxaborolan-2-yl)benzo[*d*][1,3]dioxol-5-yl)propan-2-aminium trichloroacetate (7a) – (160 MHz, $CDCl_3$)

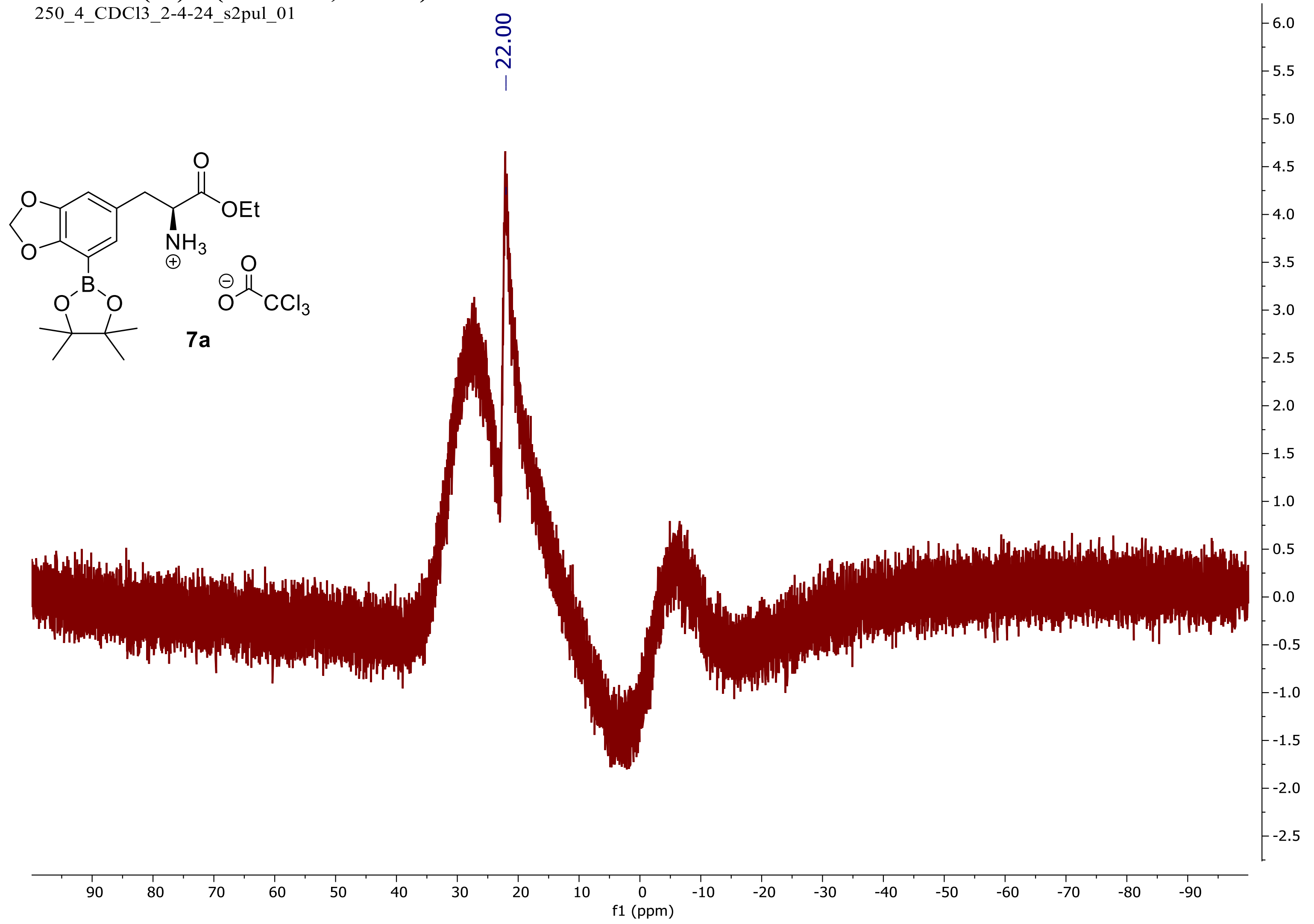

HSQC of (*S*)-1-ethoxy-1-oxo-3-(7-(4,4,5,5-tetramethyl-1,3,2-dioxaborolan-2-yl)benzo[*d*][1,3]dioxol-5-yl)propan-2-aminium trichloroacetate (7a) – ($CDCl_3$)

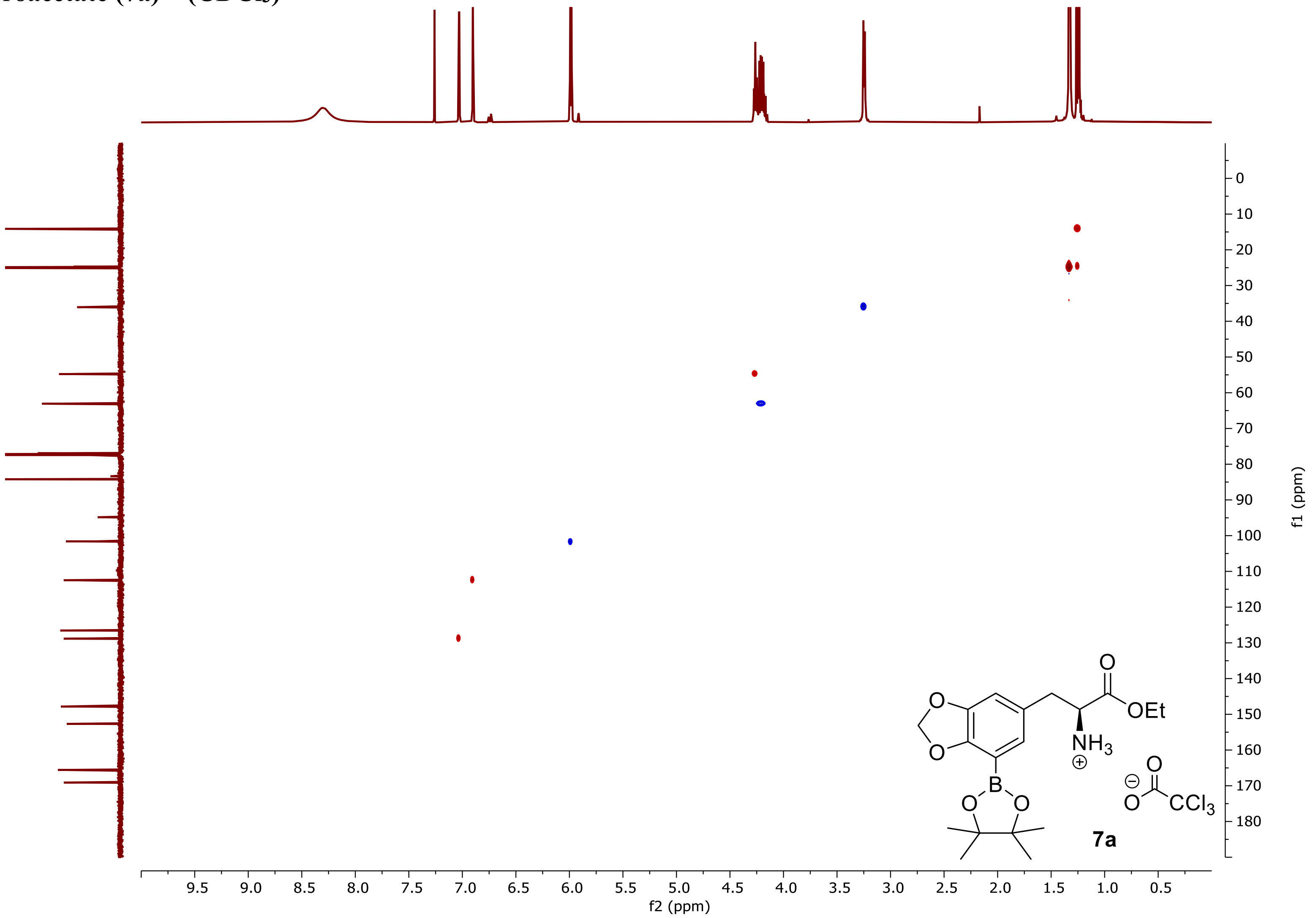

HSQC of (*S*)-1-ethoxy-1-oxo-3-(7-(4,4,5,5-tetramethyl-1,3,2-dioxaborolan-2-yl)benzo[*d*][1,3]dioxol-5-yl)propan-2-aminium trichloroacetate (7a) – ($CDCl_3$) – 5.5 to 8.5 ppm

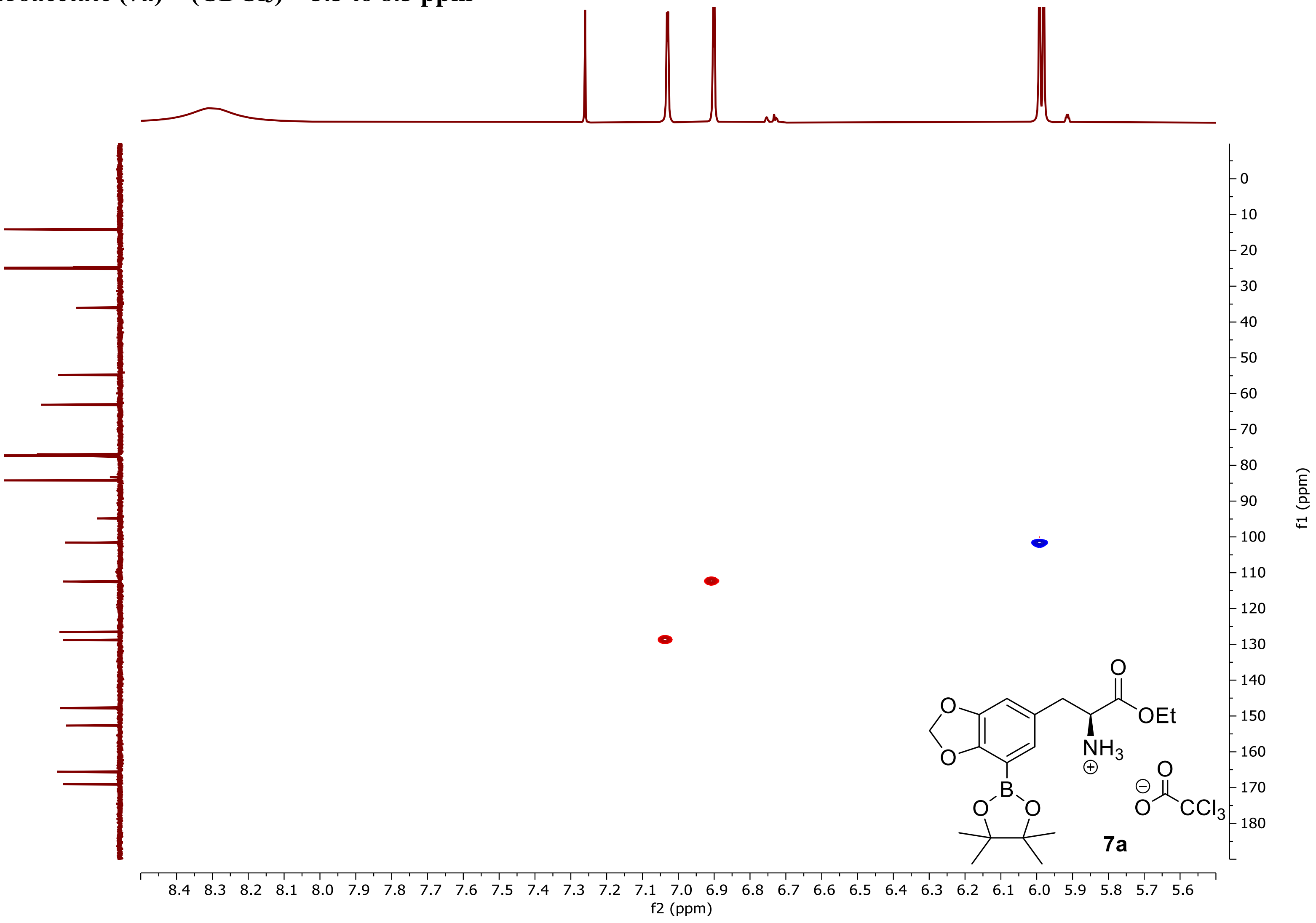

HMBC of (*S*)-1-ethoxy-1-oxo-3-(7-(4,4,5,5-tetramethyl-1,3,2-dioxaborolan-2-yl)benzo[*d*][1,3]dioxol-5-yl)propan-2-aminium trichloroacetate (7a) – ($CDCl_3$)

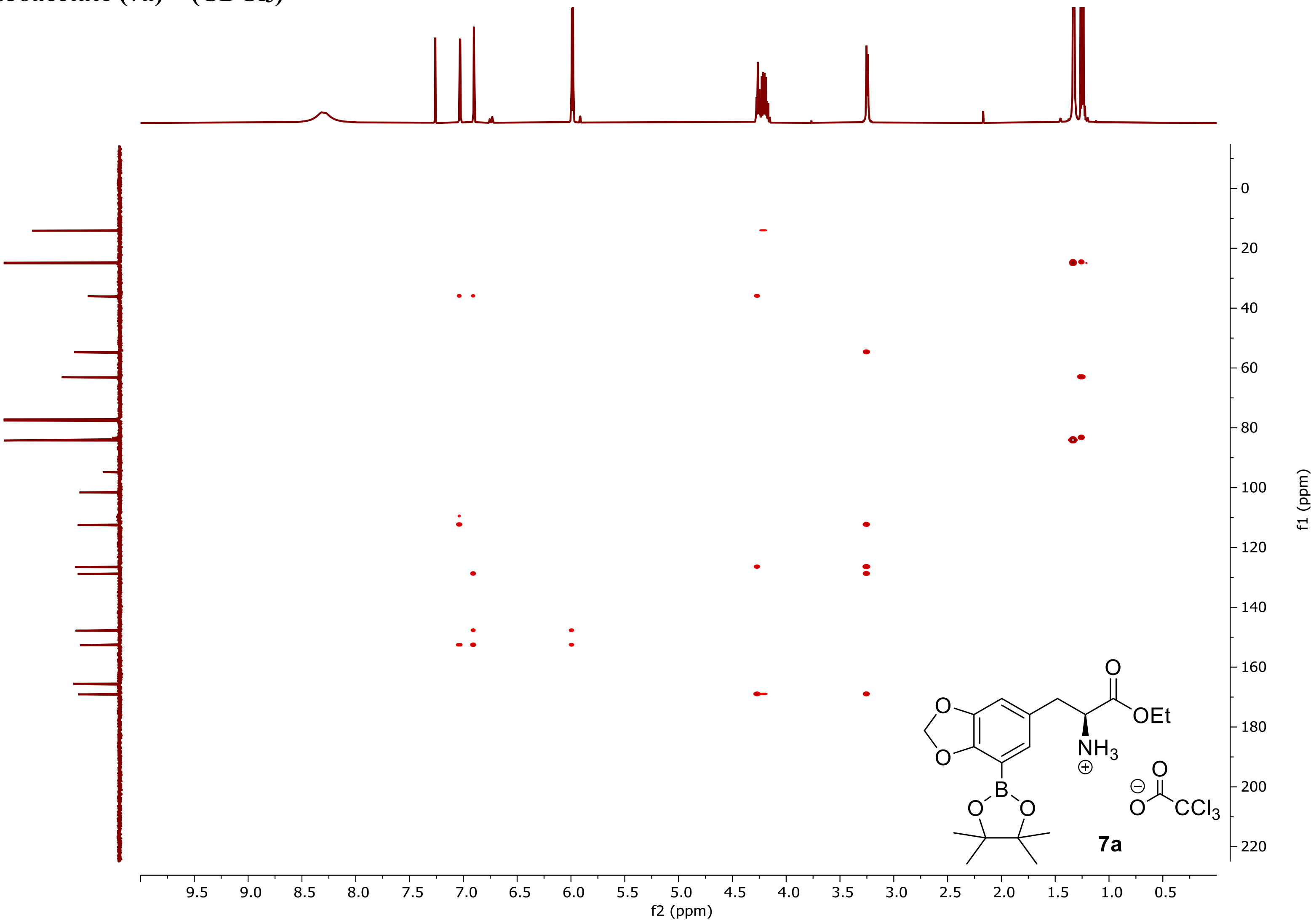

HMBC of (*S*)-1-ethoxy-1-oxo-3-(7-(4,4,5,5-tetramethyl-1,3,2-dioxaborolan-2-yl)benzo[*d*][1,3]dioxol-5-yl)propan-2-aminium trichloroacetate (7a) – ($CDCl_3$) – 5.5 to 8.5 ppm

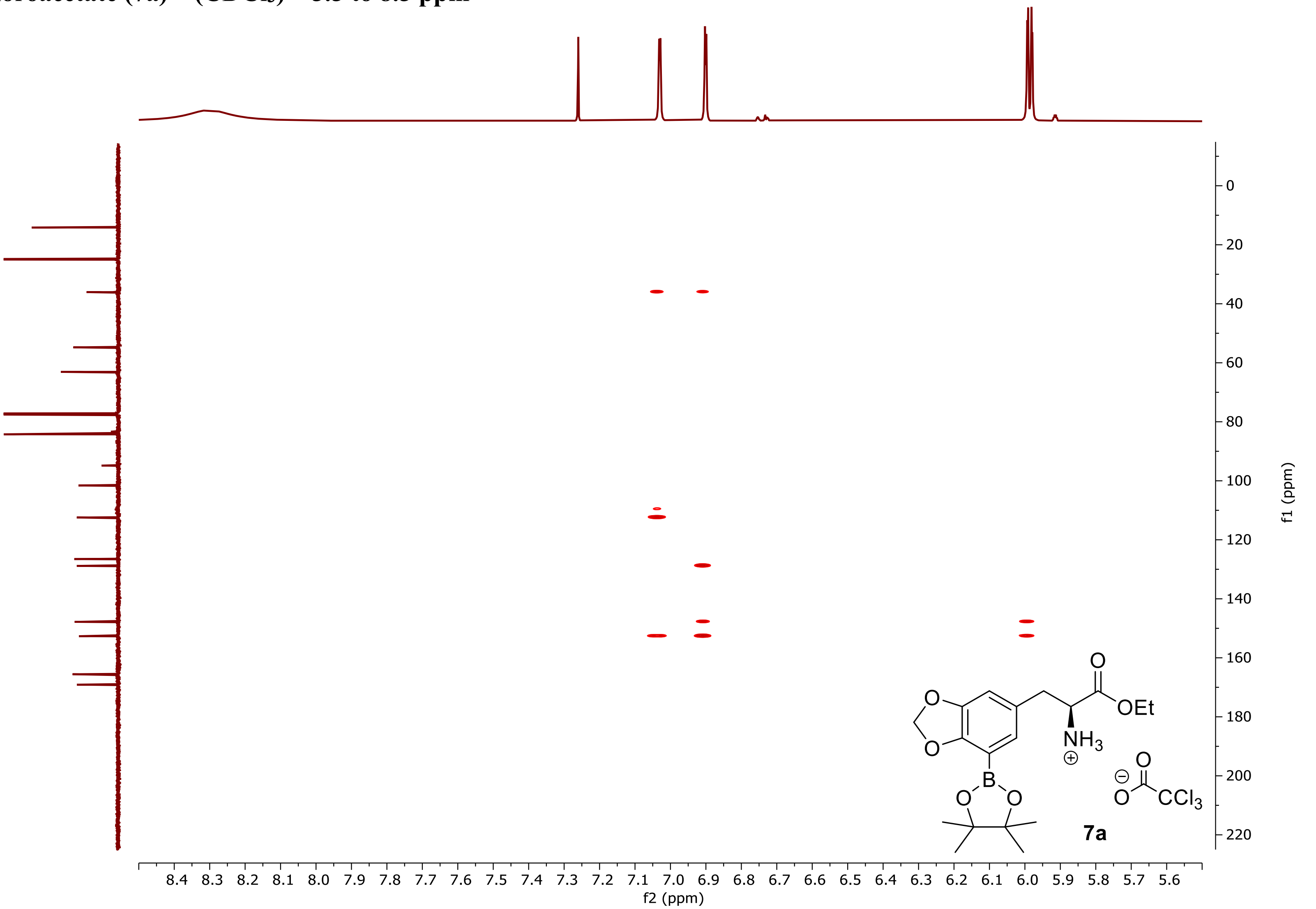

^{1}H-NMR of ethyl (*S*)-2-(dimethylamino)-3-(7-(4,4,5,5-tetramethyl-1,3,2-dioxaborolan-2-yl)benzo[*d*][1,3]dioxol-5-yl) propanoatetrichloroacetate (8a) – (500 MHz, $CDCl_3$)

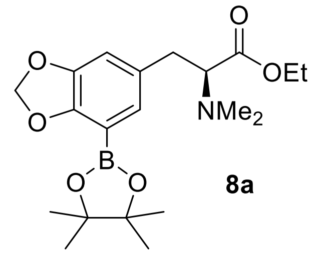

8a

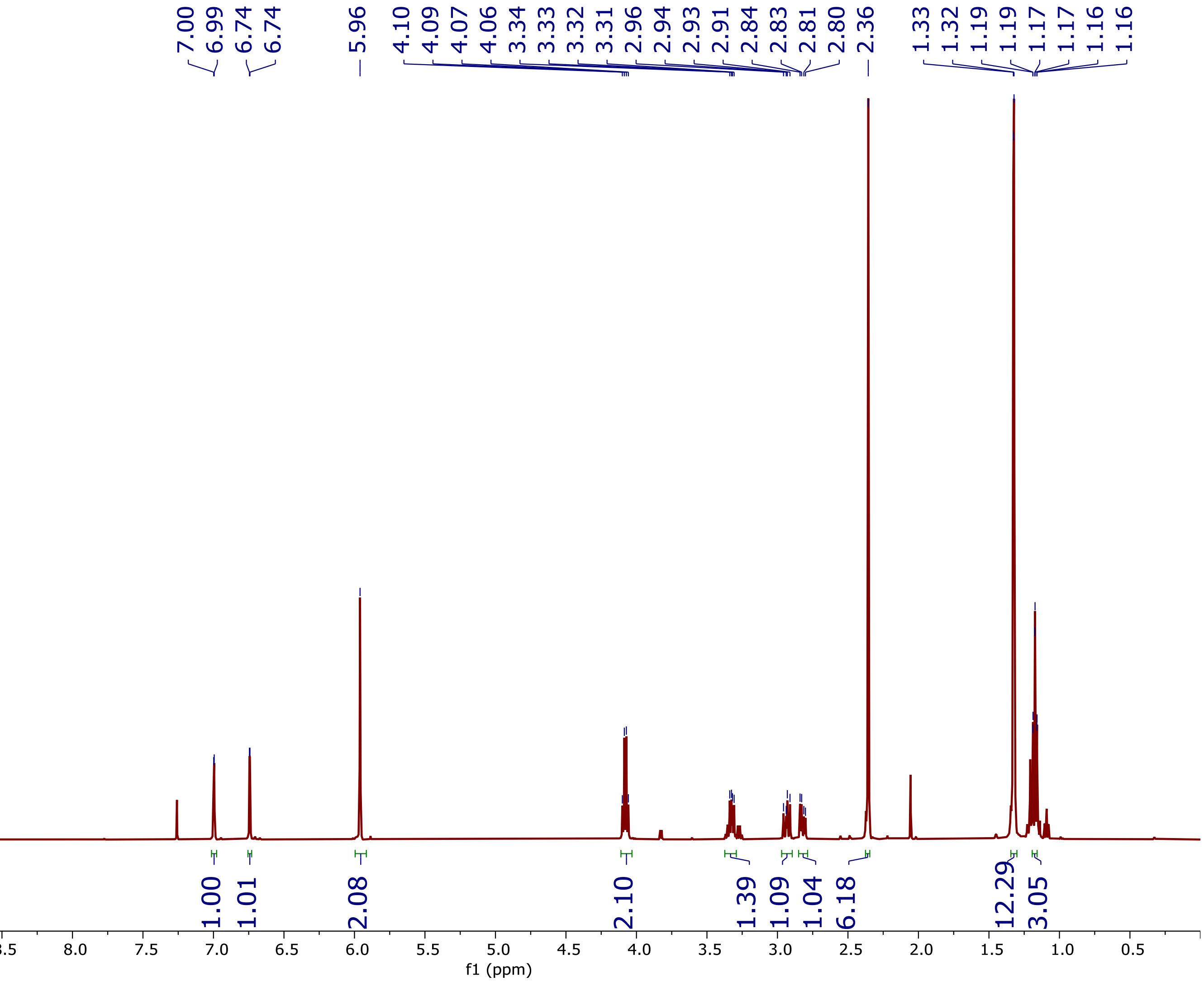

$^{13}C\{^{1}H\}$-NMR of ethyl (*S*)-2-(dimethylamino)-3-(7-(4,4,5,5-tetramethyl-1,3,2-dioxaborolan-2-yl)benzo[*d*][1,3]dioxol-5-yl) propanoatetrichloroacetate (8a) – (126 MHz, CDCl3)

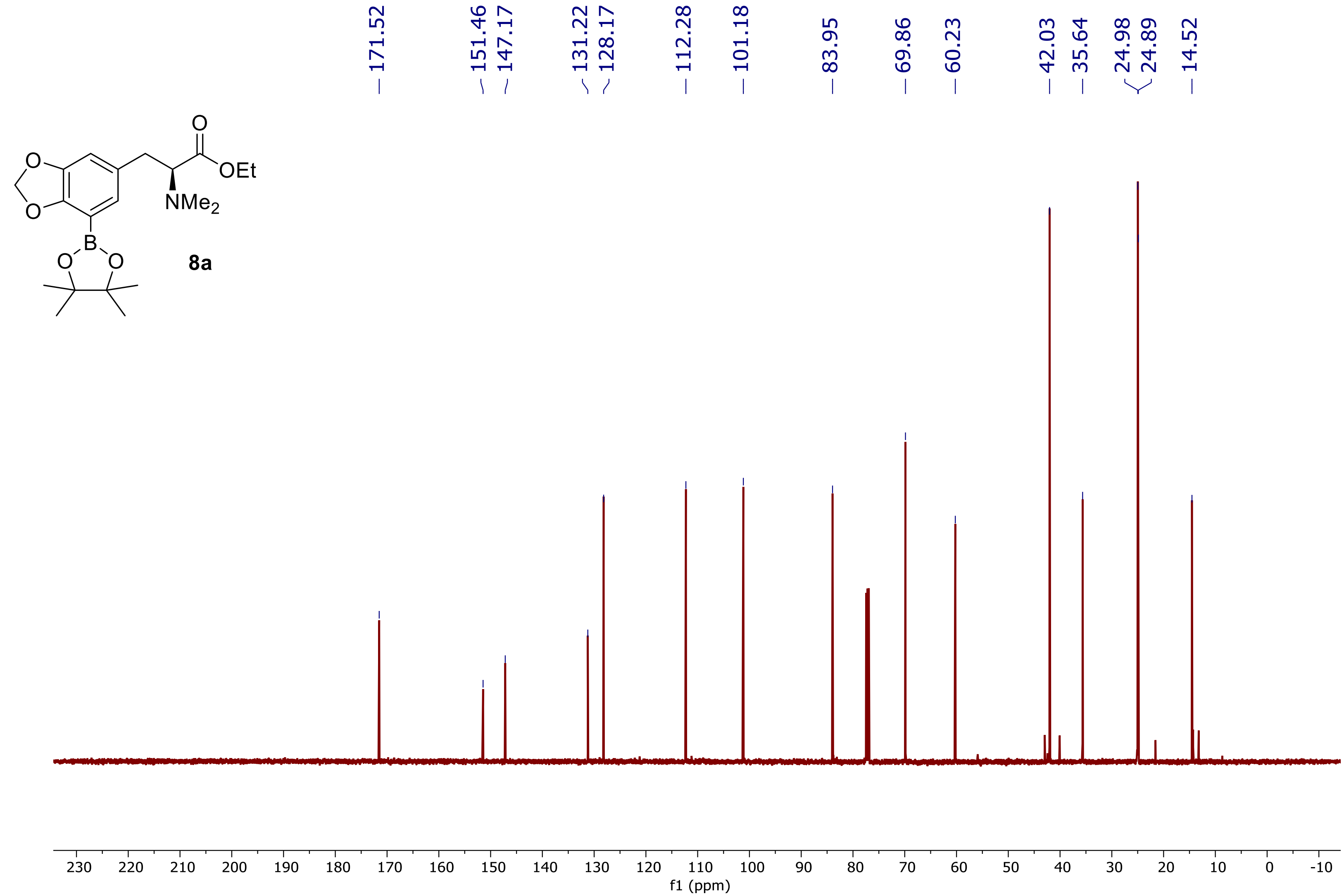

^{11}B-NMR of ethyl (*S*)-2-(dimethylamino)-3-(7-(4,4,5,5-tetramethyl-1,3,2-dioxaborolan-2-yl)benzo[*d*][1,3]dioxol-5-yl) propanoatetrichloroacetate (8a) – (160 MHz, CDCl3)

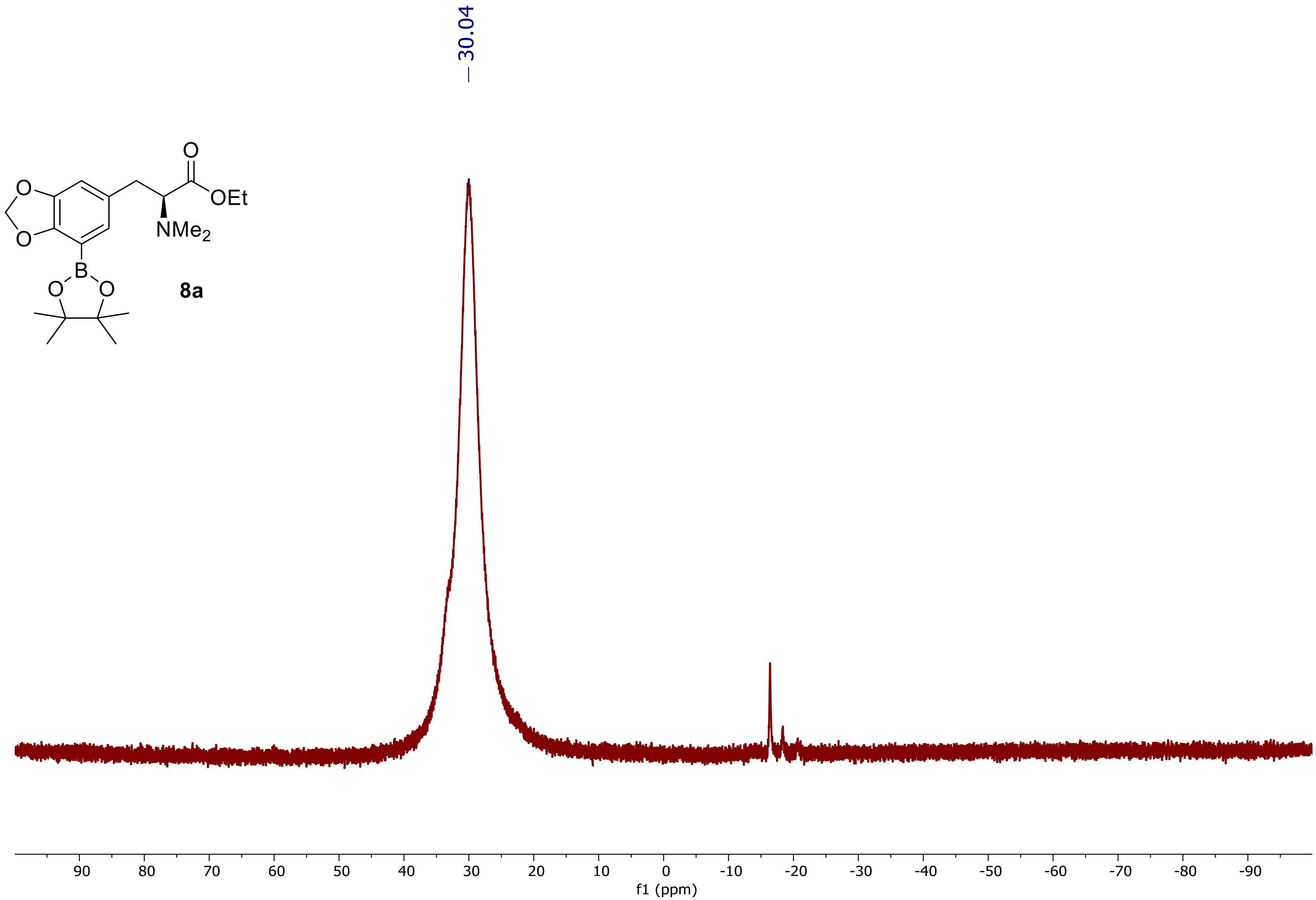

HSQC of ethyl (*S*)-2-(dimethylamino)-3-(7-(4,4,5,5-tetramethyl-1,3,2-dioxaborolan-2-yl)benzo[*d*][1,3]dioxol-5-yl) propanoatetrichloroacetate (8a) – ($CDCl_3$)

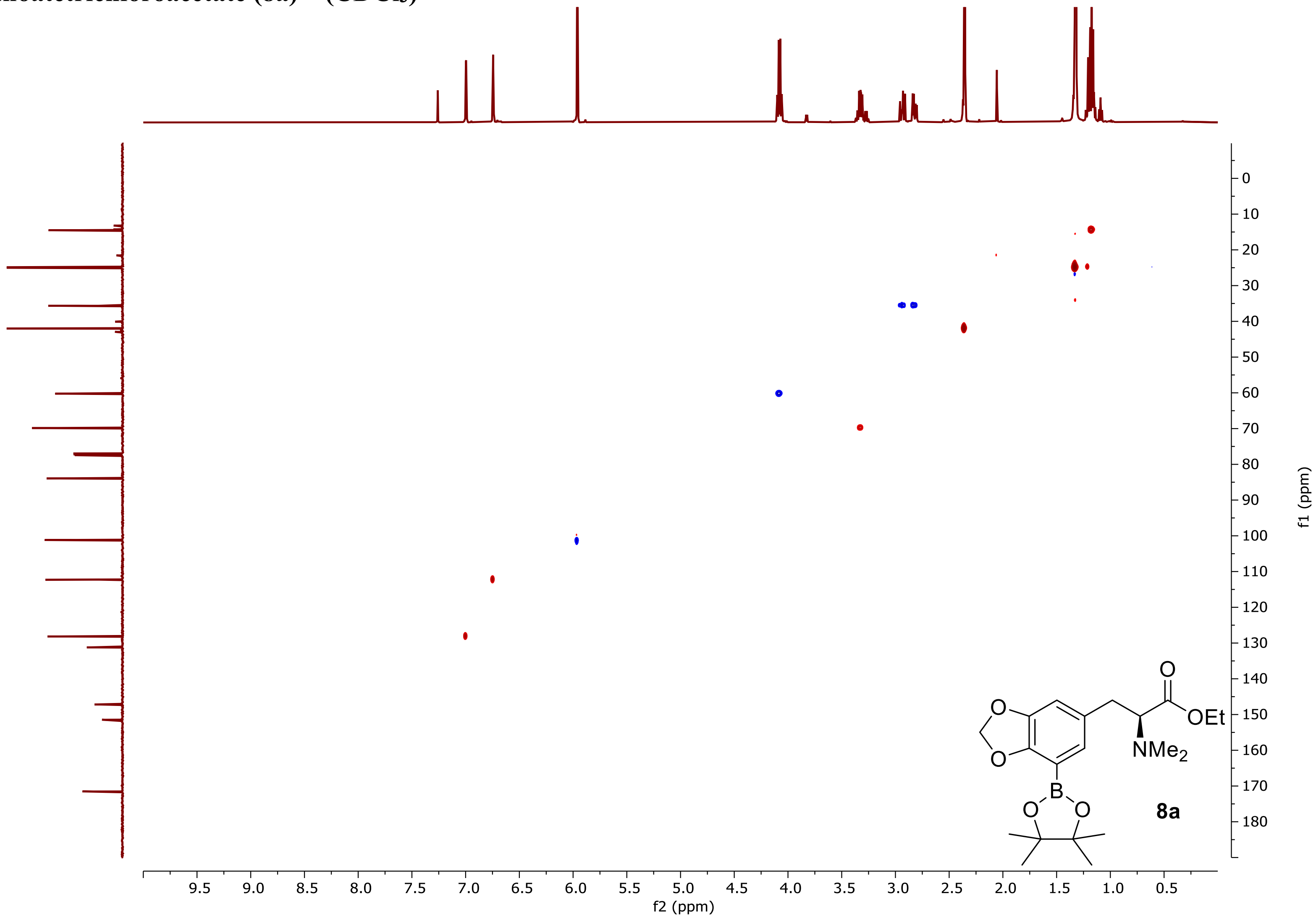

HSQC of ethyl (*S*)-2-(dimethylamino)-3-(7-(4,4,5,5-tetramethyl-1,3,2-dioxaborolan-2-yl)benzo[*d*][1,3]dioxol-5-yl) propanoatetrichloroacetate (8a) – ($CDCl_3$) – 5.5 to 7.5 ppm

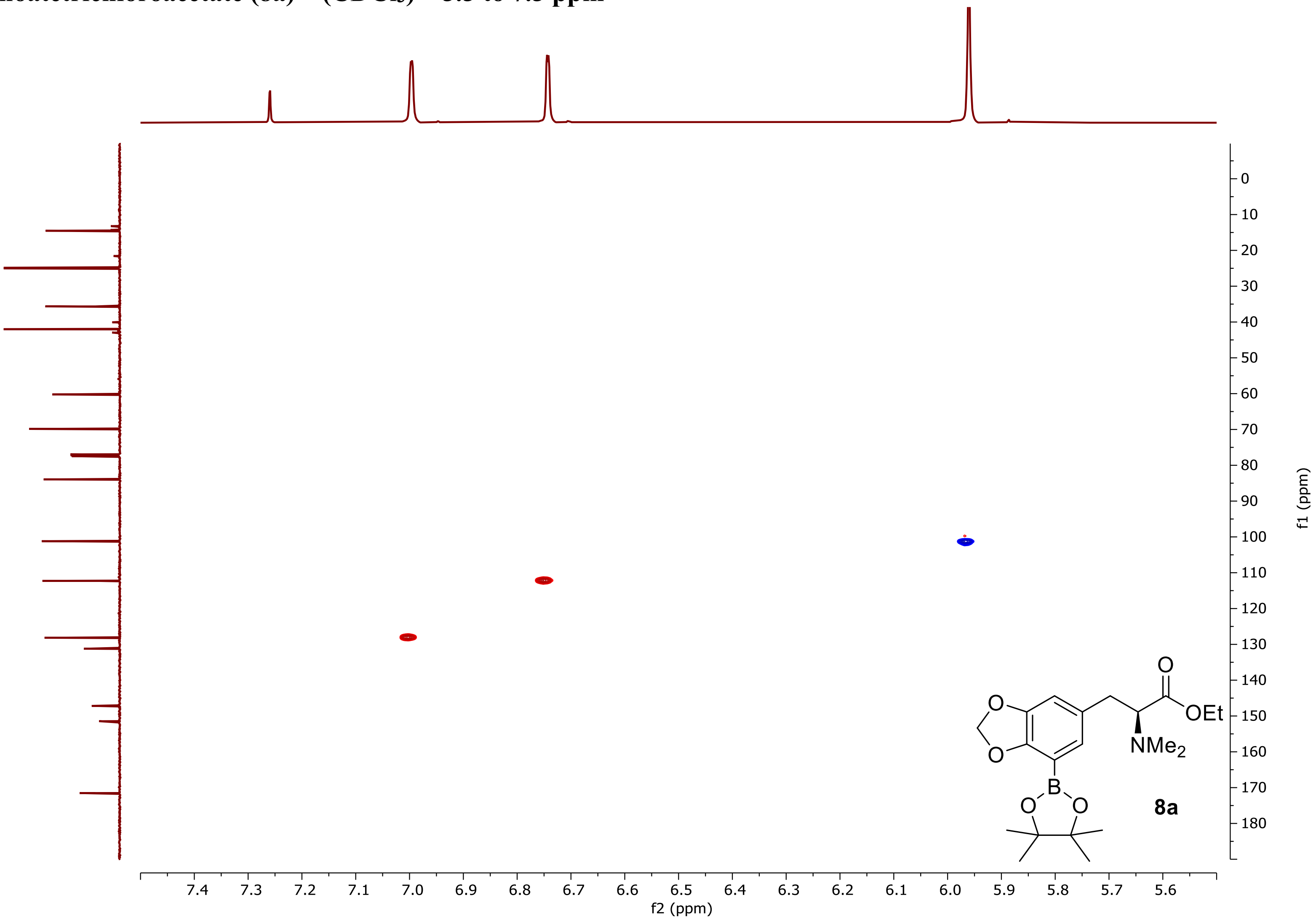

HMBC of ethyl (*S*)-2-(dimethylamino)-3-(7-(4,4,5,5-tetramethyl-1,3,2-dioxaborolan-2-yl)benzo[*d*][1,3]dioxol-5-yl) propanoatetrichloroacetate (8a) – ($CDCl_3$)

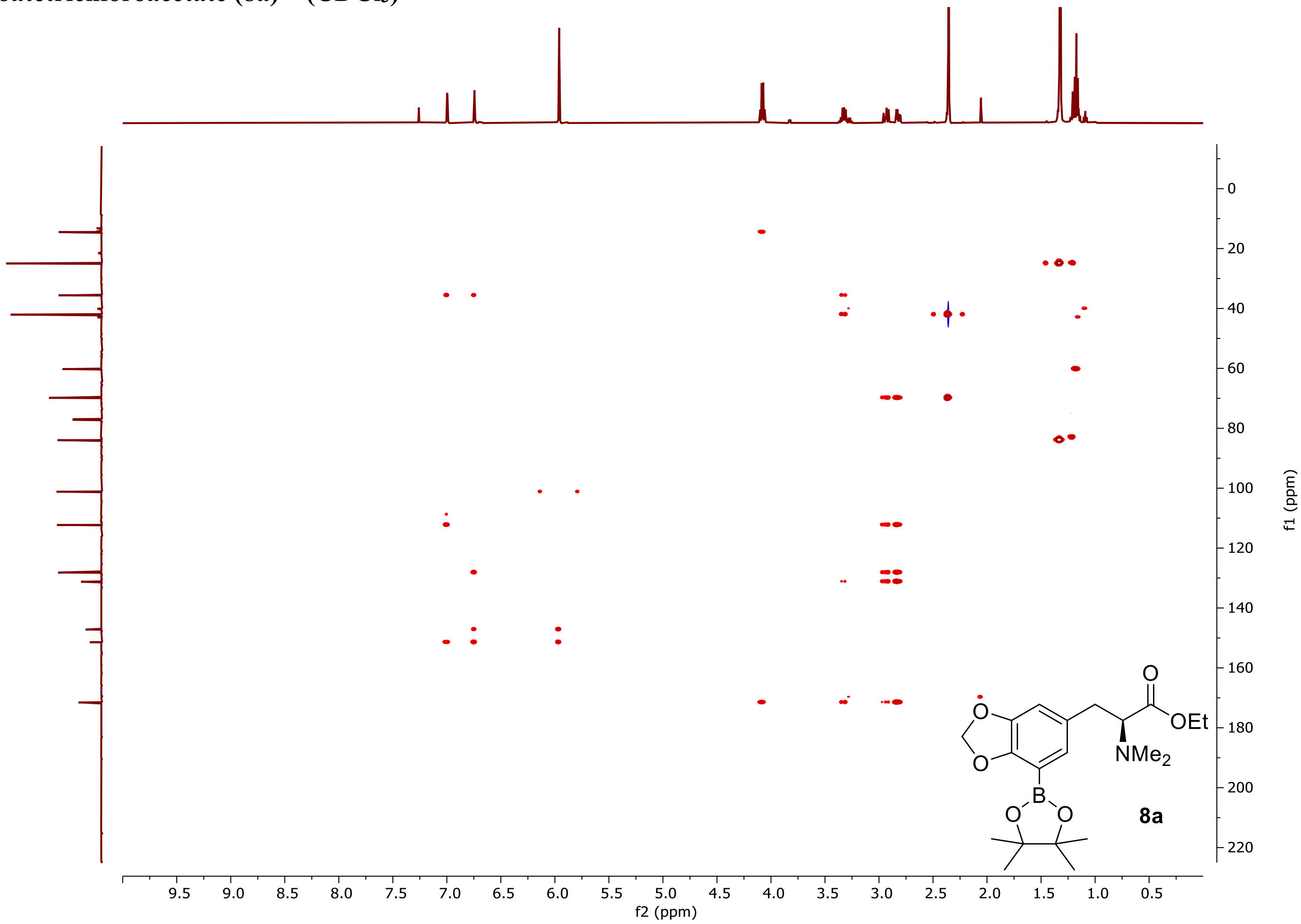

HMBC of ethyl (*S*)-2-(dimethylamino)-3-(7-(4,4,5,5-tetramethyl-1,3,2-dioxaborolan-2-yl)benzo[*d*][1,3]dioxol-5-yl) propanoatetrichloroacetate (8a) – ($CDCl_3$) – 5.5 to 7.5 ppm

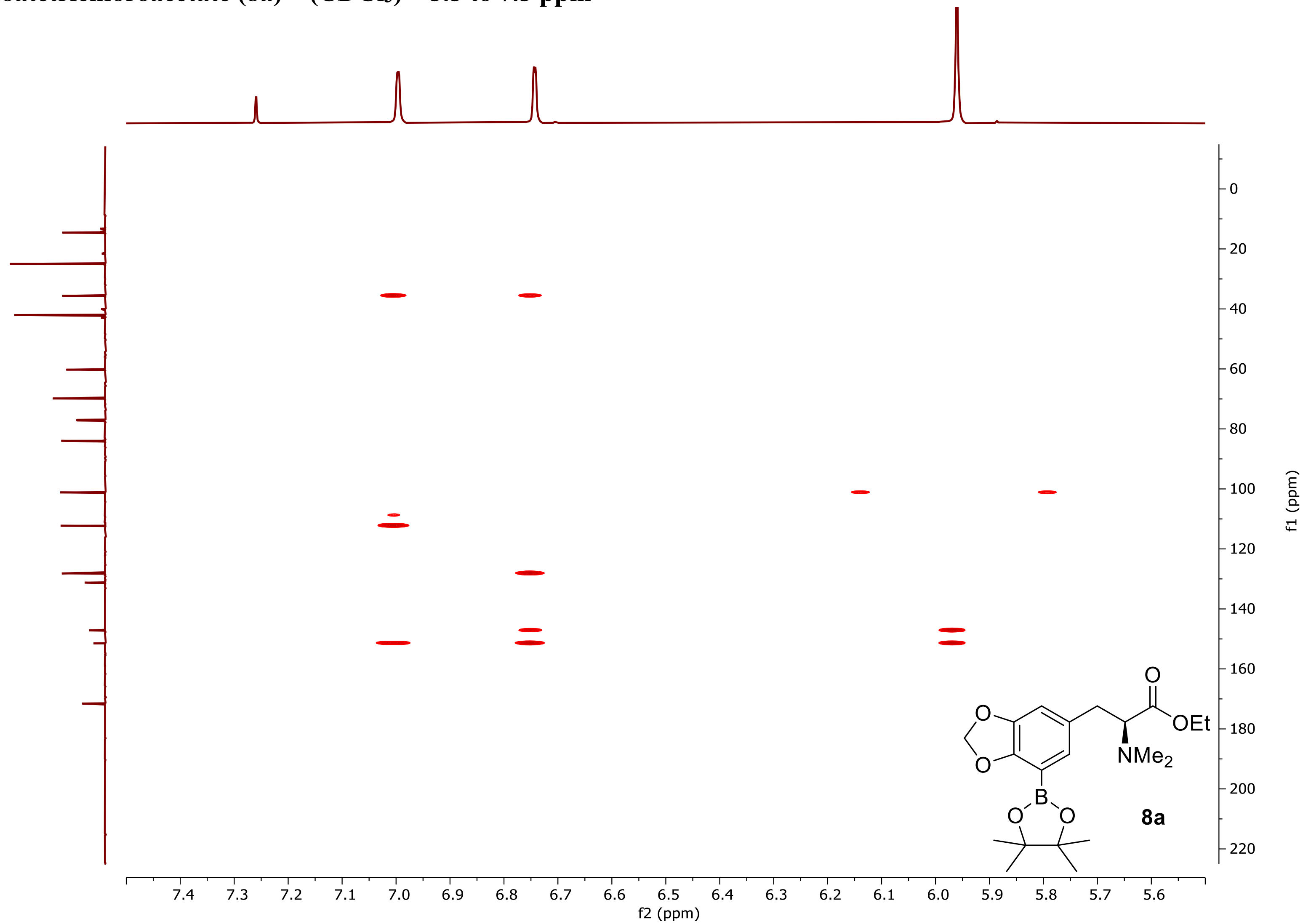

^{1}H-NMR of ethyl (*S*)-2-((*tert*-butoxycarbonyl)(methyl)amino)-3-(7-(4,4,5,5-tetramethyl-1,3,2-dioxaborolan-2-yl) benzo[*d*][1,3]dioxol-5-yl)propanoate (9a) – (500 MHz, $CDCl_3$)

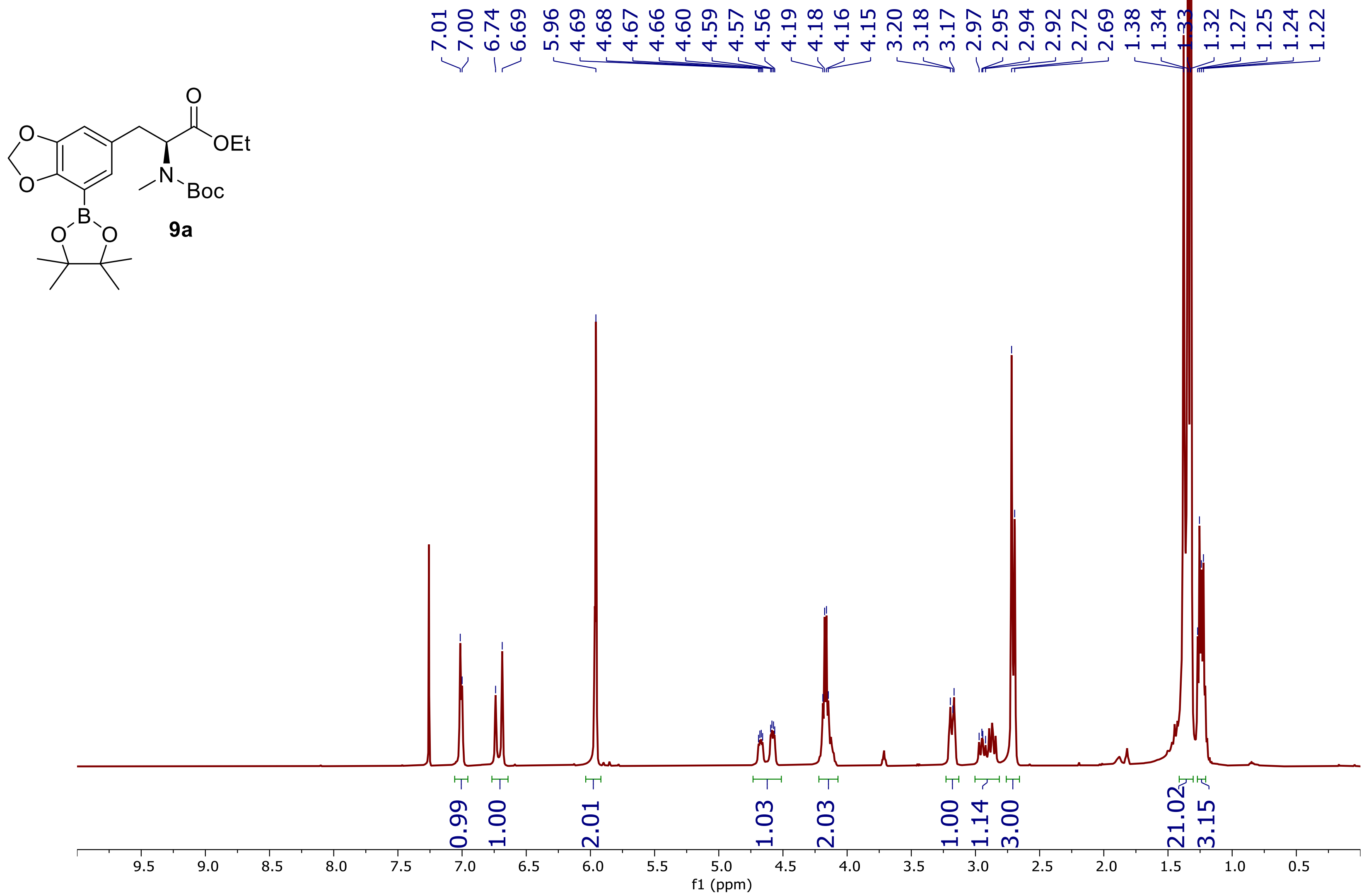

$^{13}C\{^1H\}$-NMR of ethyl (*S*)-2-((*tert*-butoxycarbonyl)(methyl)amino)-3-(7-(4,4,5,5-tetramethyl-1,3,2-dioxaborolan-2-yl) benzo[*d*][1,3]dioxol-5-yl)propanoate (9a) – (126 MHz, CDCl₃)

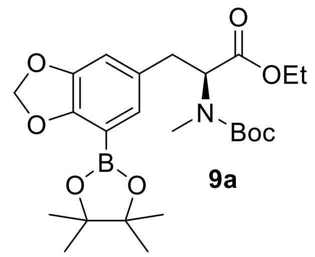

9a

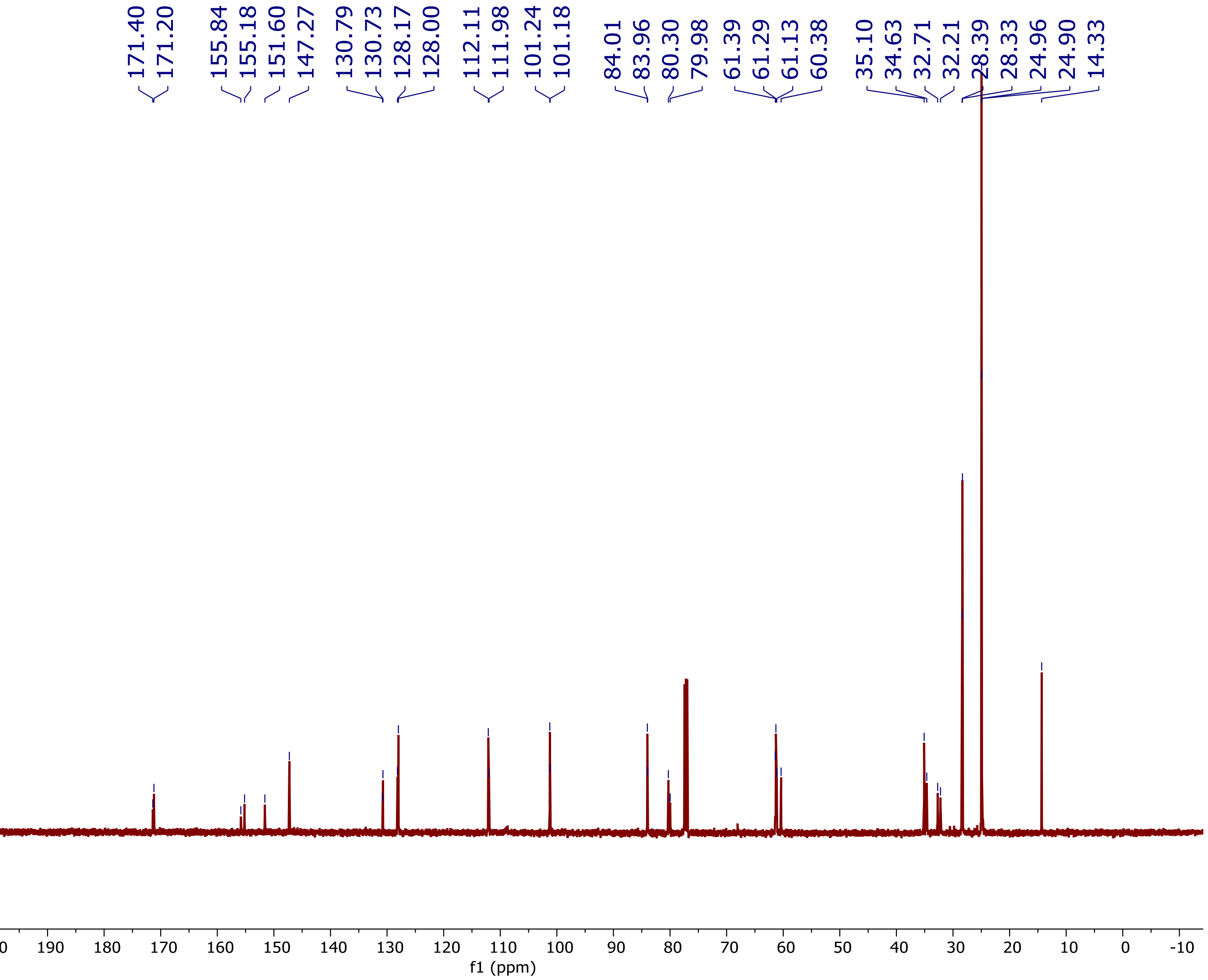

^{11}B-NMR of ethyl (*S*)-2-((*tert*-butoxycarbonyl)(methyl)amino)-3-(7-(4,4,5,5-tetramethyl-1,3,2-dioxaborolan-2-yl)benzo[*d*][1,3]dioxol-5-yl)propanoate (9a) – (160 MHz, $CDCl_3$)

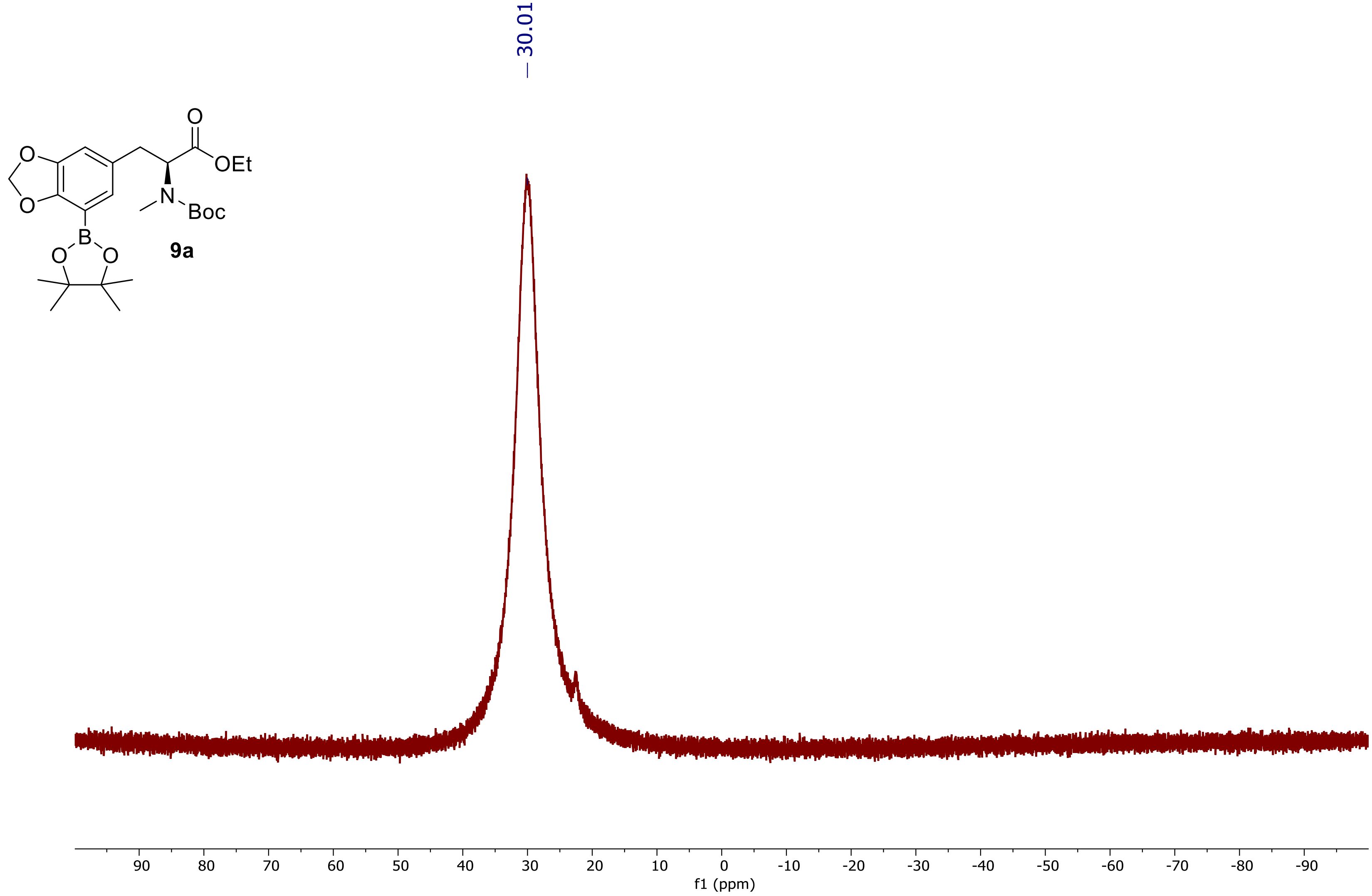

HSQC of ethyl (*S*)-2-((*tert*-butoxycarbonyl)(methyl)amino)-3-(7-(4,4,5,5-tetramethyl-1,3,2-dioxaborolan-2-yl) benzo[*d*][1,3]dioxol-5-yl)propanoate (9a) – ($CDCl_3$)

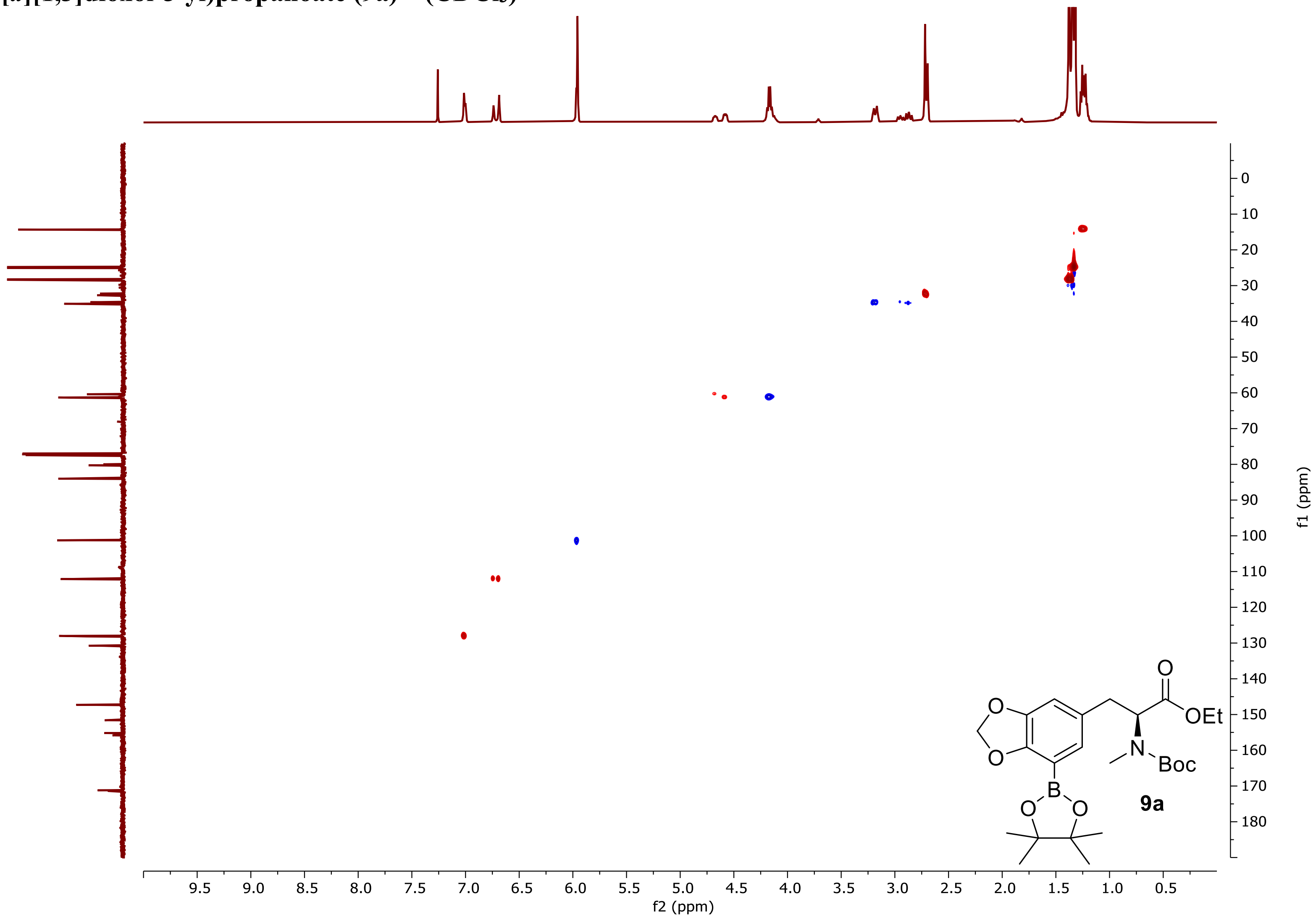

HSQC of ethyl (*S*)-2-((*tert*-butoxycarbonyl)(methyl)amino)-3-(7-(4,4,5,5-tetramethyl-1,3,2-dioxaborolan-2-yl) benzo[*d*][1,3]dioxol-5-yl)propanoate (9a) – ($CDCl_3$) – 5.5 to 7.5 ppm

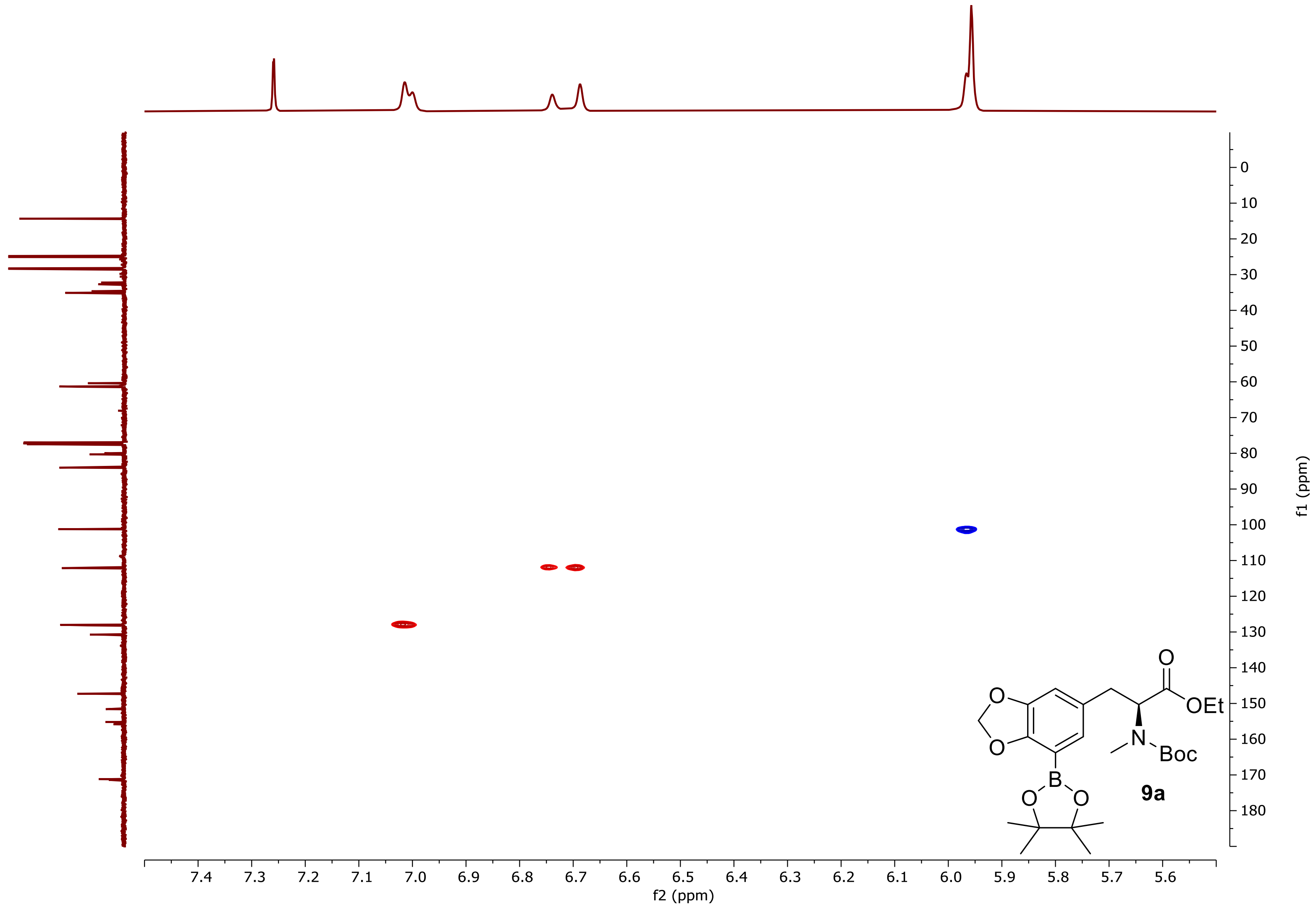

HMBC of ethyl (*S*)-2-((*tert*-butoxycarbonyl)(methyl)amino)-3-(7-(4,4,5,5-tetramethyl-1,3,2-dioxaborolan-2-yl) benzo[*d*][1,3]dioxol-5-yl)propanoate (9a) – ($CDCl_3$)

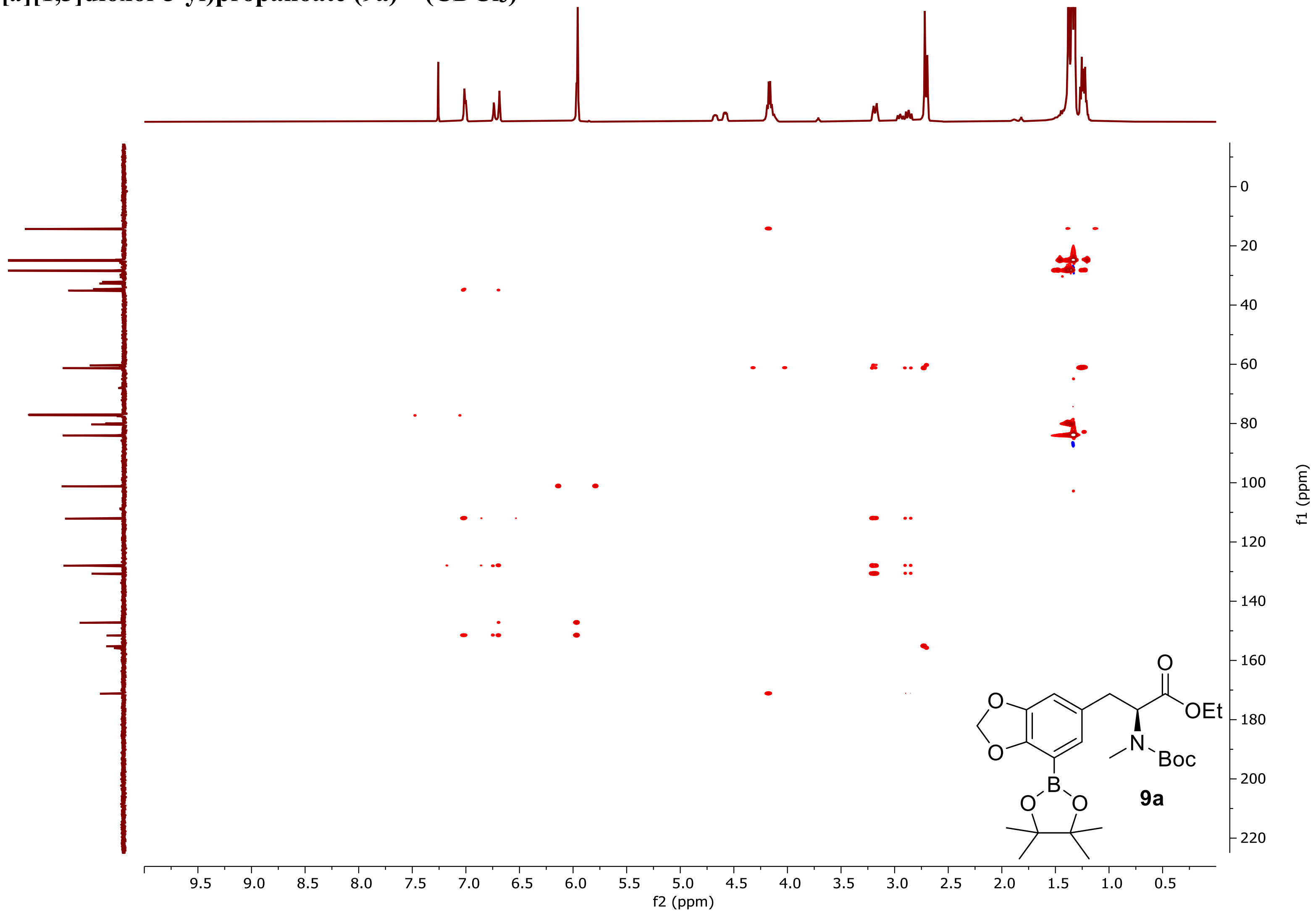

HMBC of ethyl (*S*)-2-((*tert*-butoxycarbonyl)(methyl)amino)-3-(7-(4,4,5,5-tetramethyl-1,3,2-dioxaborolan-2-yl) benzo[*d*][1,3]dioxol-5-yl)propanoate (9a) – ($CDCl_3$) – 5.5 to 7.5 ppm

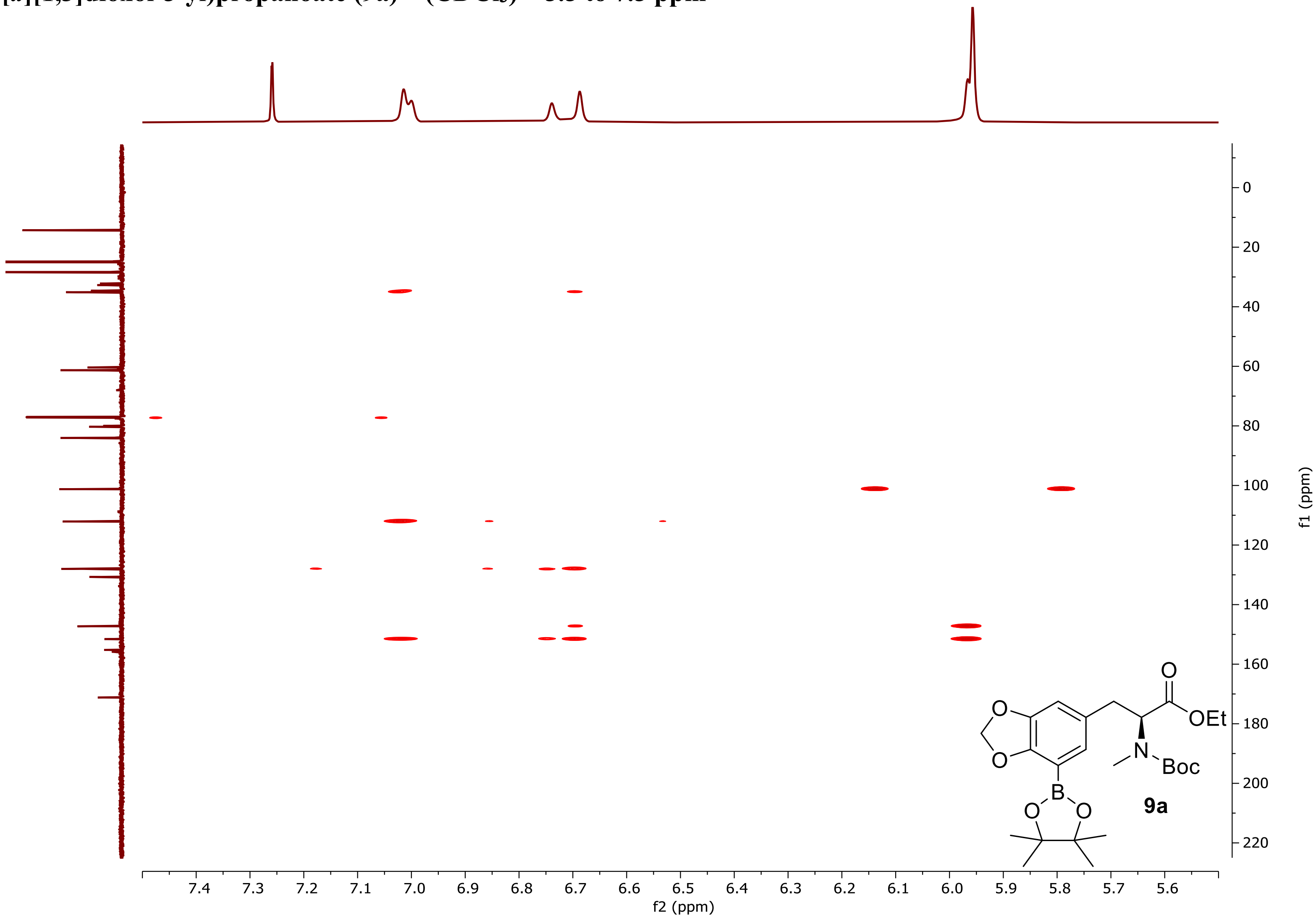

^{1}H-NMR of ethyl (*S*)-2-(methylamino)-3-(7-(4,4,5,5-tetramethyl-1,3,2-dioxaborolan-2-yl)benzo[*d*][1,3]dioxol-5-yl)propanoate (10a) – (500 MHz, $CDCl_3$)

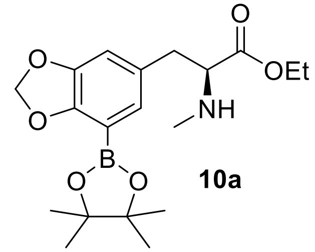

10a

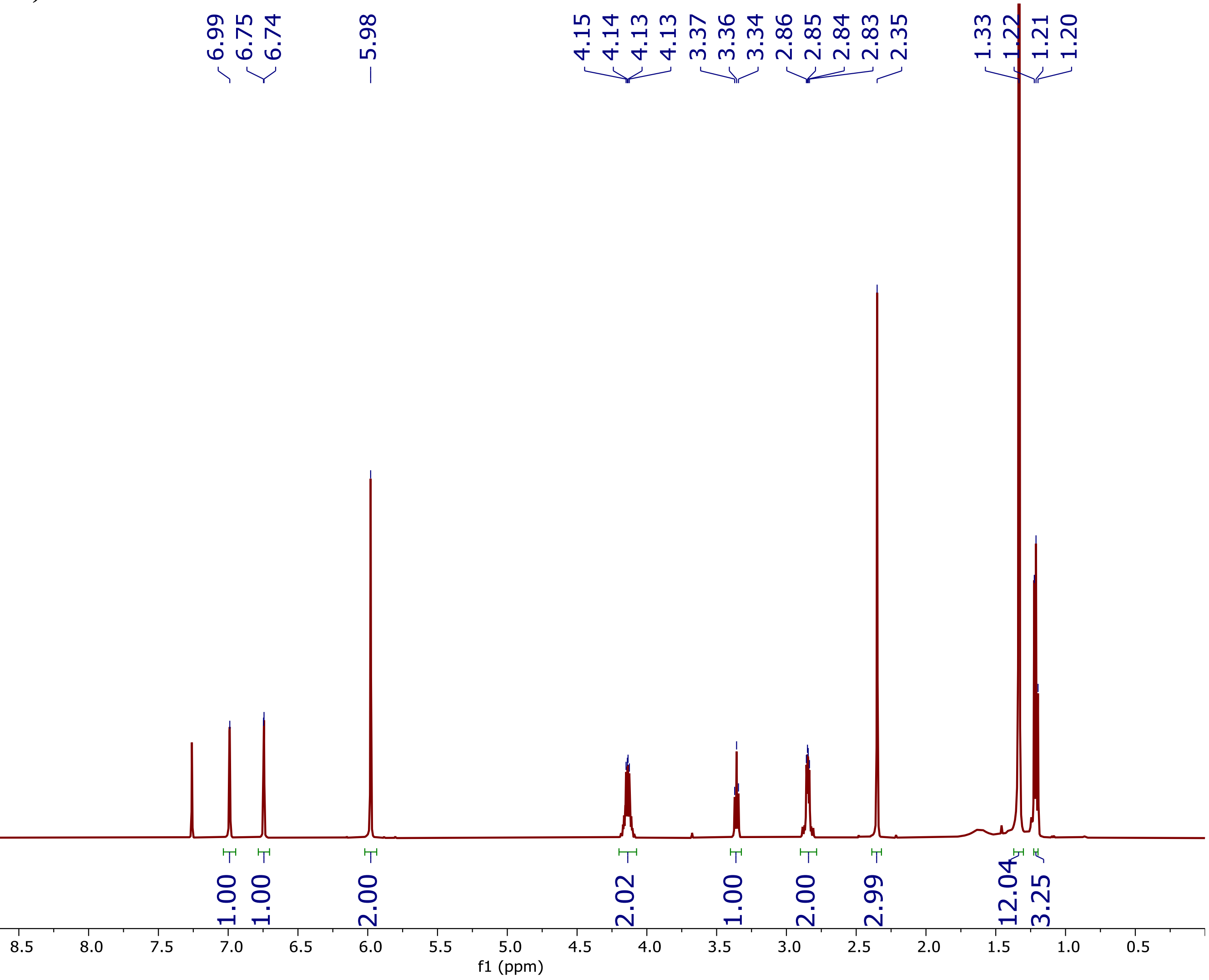

$^{13}C\{^{1}H\}$-NMR of ethyl (*S*)-2-(methylamino)-3-(7-(4,4,5,5-tetramethyl-1,3,2-dioxaborolan-2-yl)benzo[*d*][1,3]dioxol-5-yl)propanoate (10a) – (126 MHz, CDCl₃)

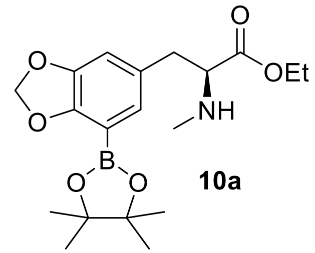

10a

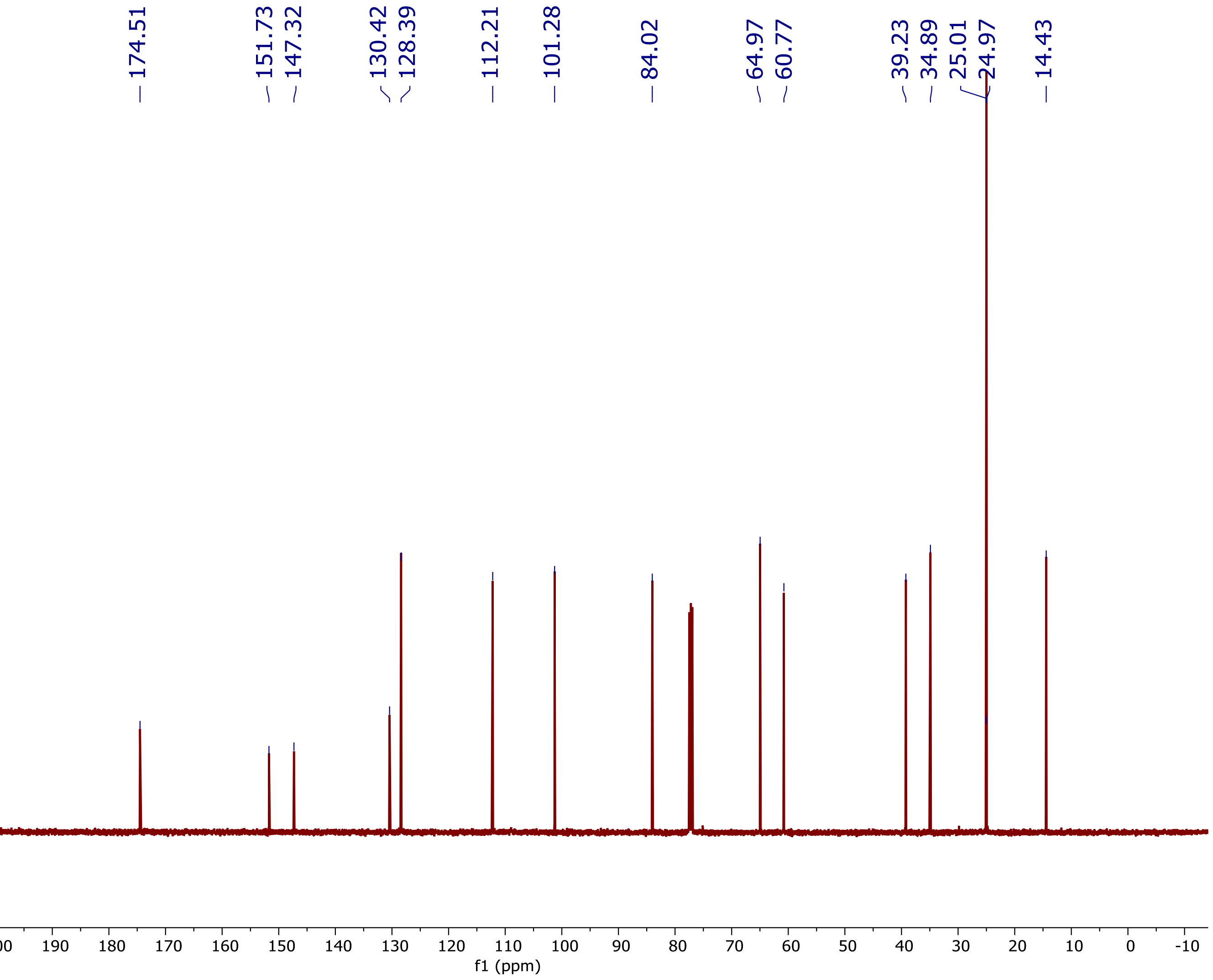

^{11}B-NMR of ethyl (*S*)-2-(methylamino)-3-(7-(4,4,5,5-tetramethyl-1,3,2-dioxaborolan-2-yl)benzo[*d*][1,3]dioxol-5-yl)propanoate (10a) – (160 MHz, $CDCl_3$)

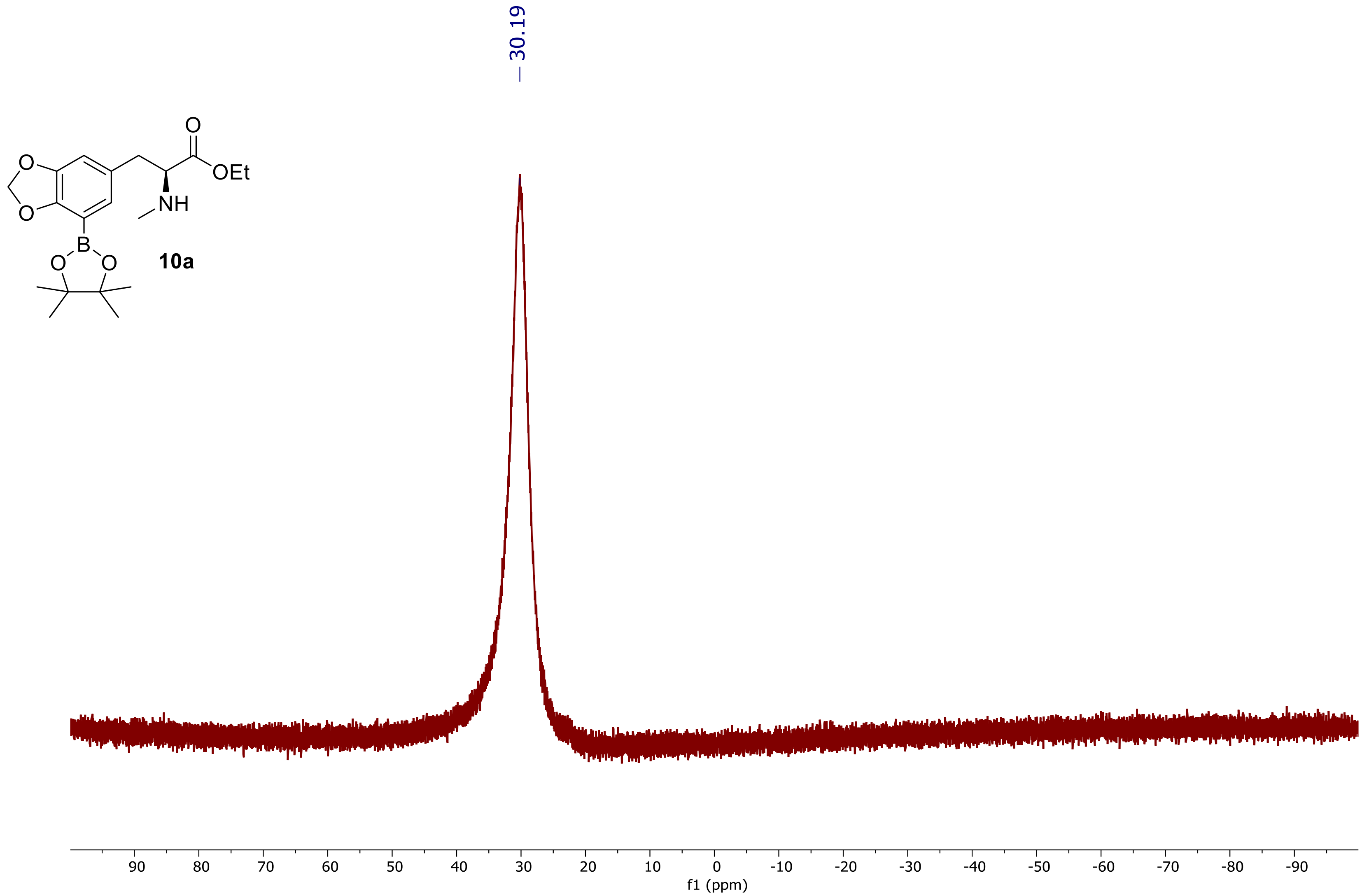

HSQC of ethyl (*S*)-2-(methylamino)-3-(7-(4,4,5,5-tetramethyl-1,3,2-dioxaborolan-2-yl)benzo[*d*][1,3]dioxol-5-yl)propanoate (10a) – ($CDCl_3$)

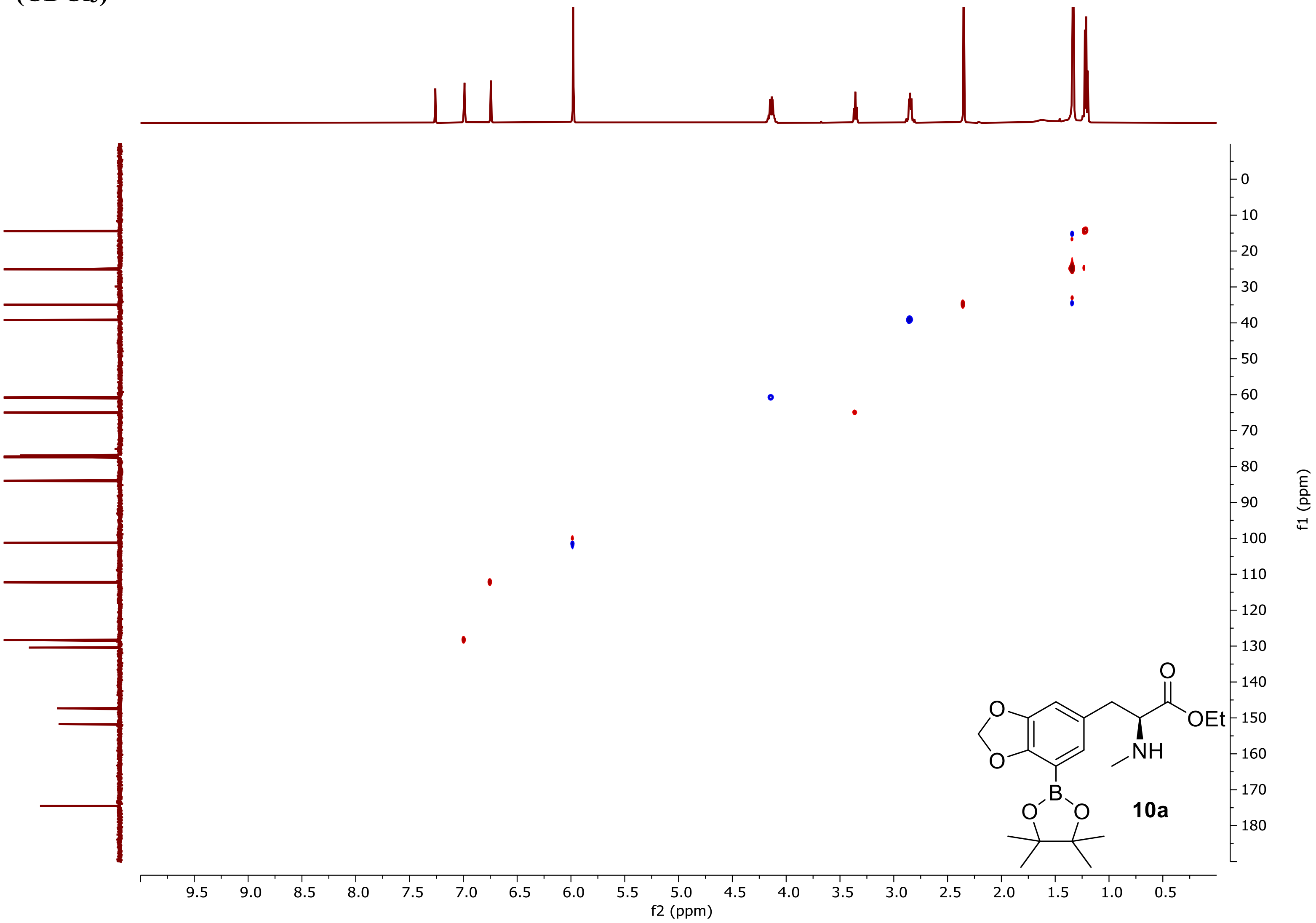

HSQC of ethyl (*S*)-2-(methylamino)-3-(7-(4,4,5,5-tetramethyl-1,3,2-dioxaborolan-2-yl)benzo[*d*][1,3]dioxol-5-yl)propanoate (10a) – ($CDCl_3$) – 5.5 to 7.5 ppm

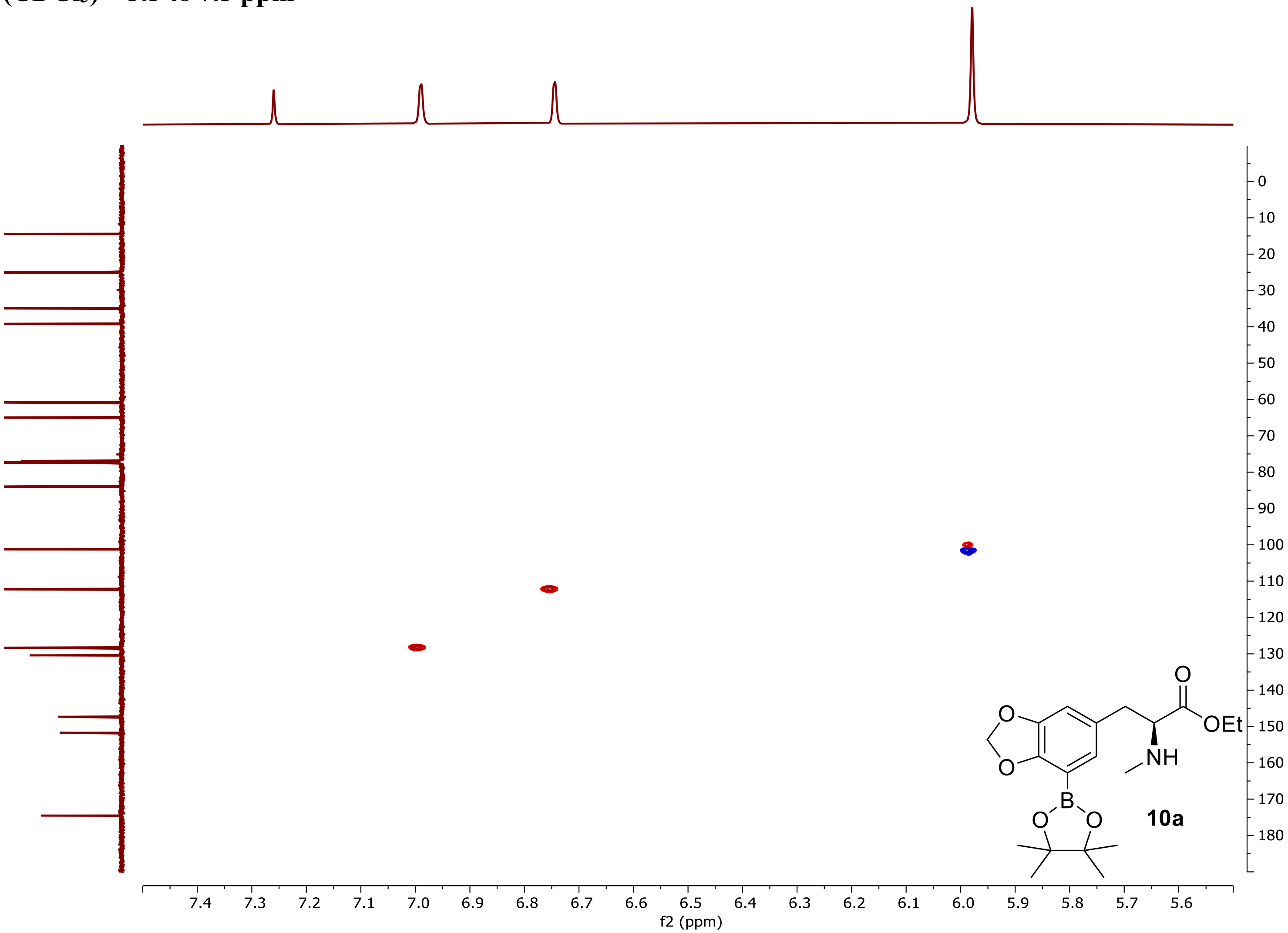

HMBC of ethyl (*S*)-2-(methylamino)-3-(7-(4,4,5,5-tetramethyl-1,3,2-dioxaborolan-2-yl)benzo[*d*][1,3]dioxol-5-yl)propanoate (10a) – (CDCl3)

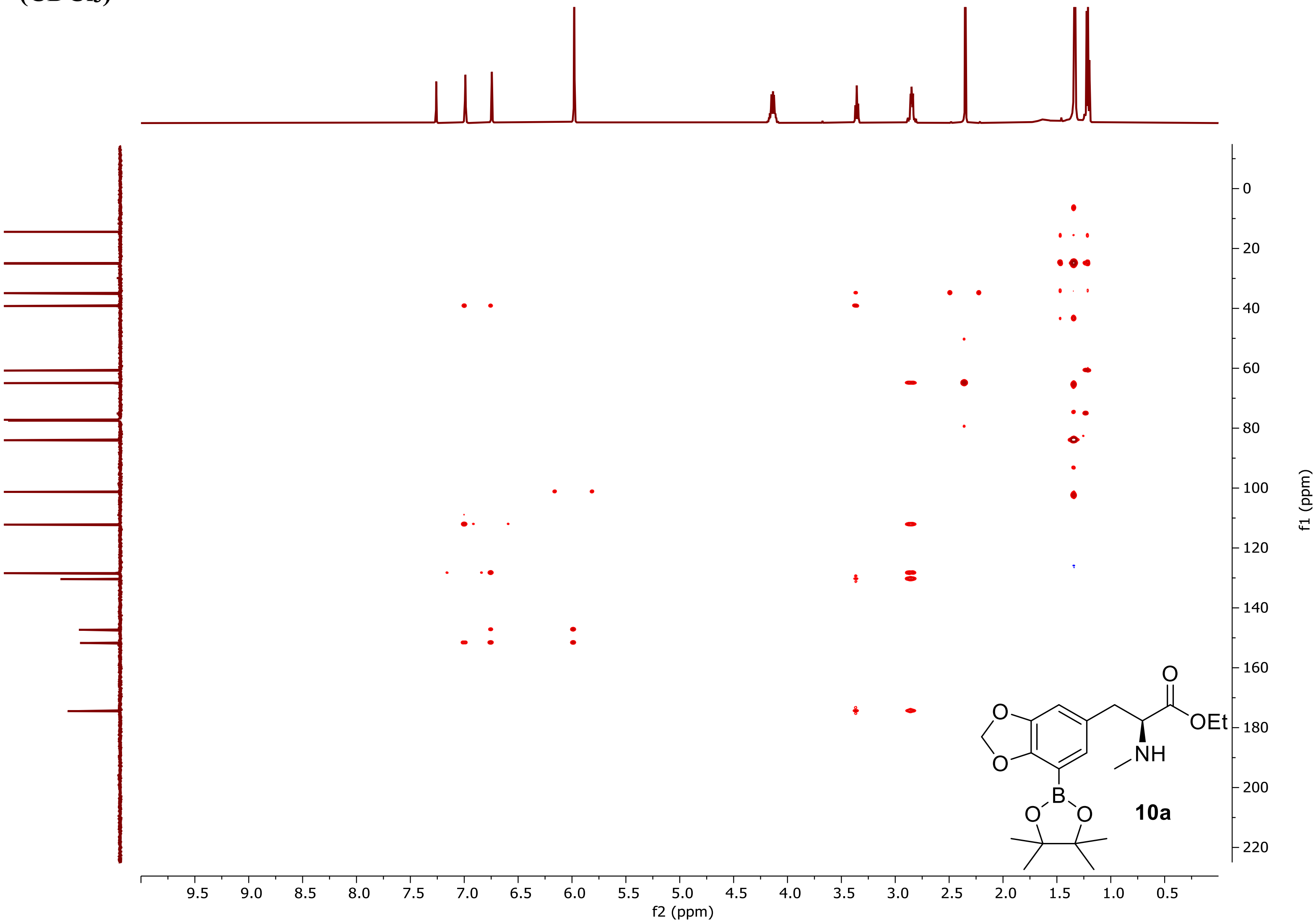

HMBC of ethyl (*S*)-2-(methylamino)-3-(7-(4,4,5,5-tetramethyl-1,3,2-dioxaborolan-2-yl)benzo[*d*][1,3]dioxol-5-yl)propanoate (10a) – ($CDCl_3$) – 5.5 to 7.5 ppm

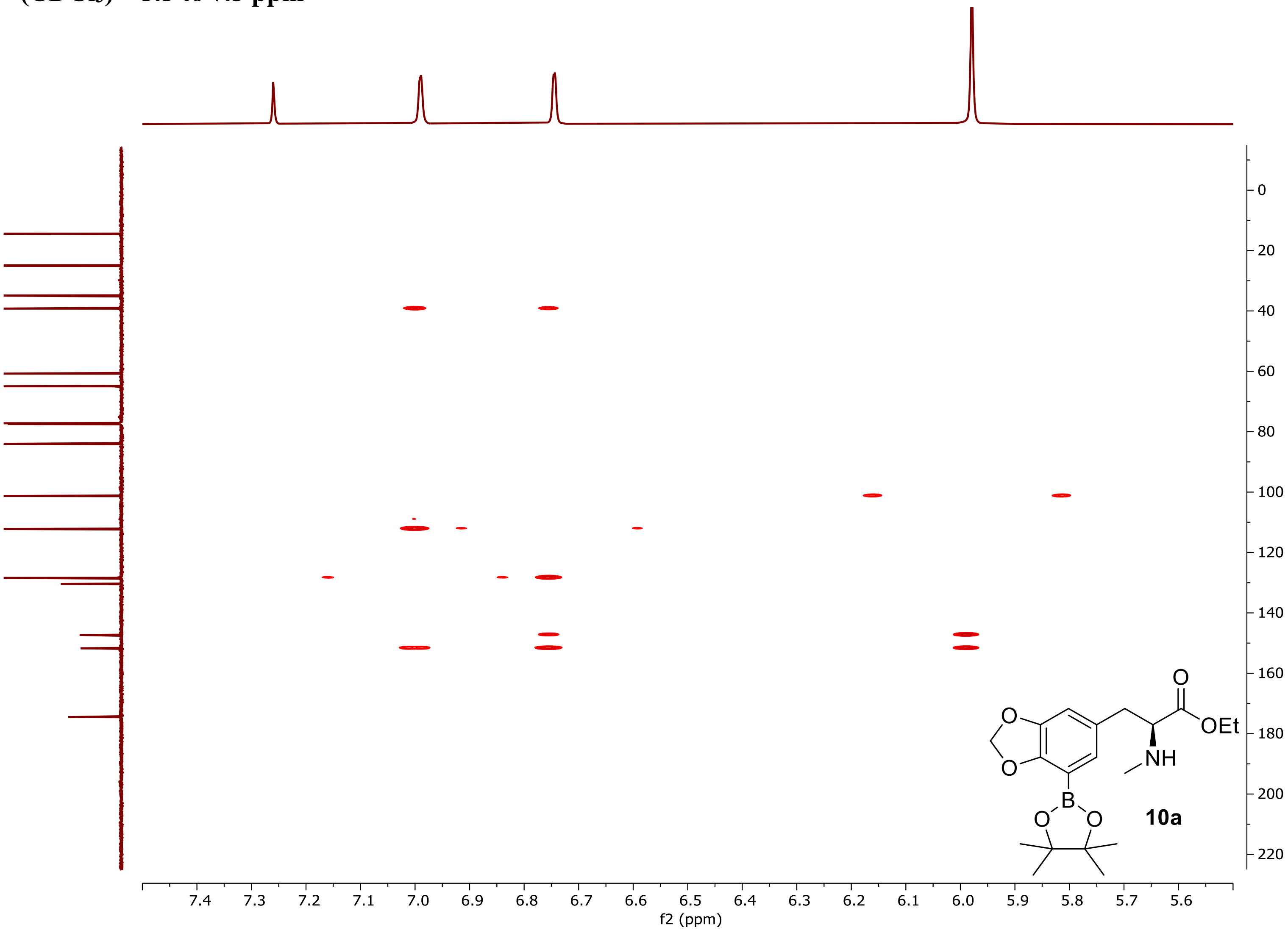

^{1}H-NMR of ethyl (*S*)-2-((*tert*-butoxycarbonyl)amino)-3-(3,4-dihydroxy-5-(4,4,5,5-tetramethyl-1,3,2-dioxaborolan-2-yl) phenyl)propanoate (12) – (500 MHz, $CDCl_3$)

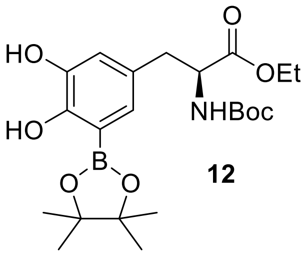

12

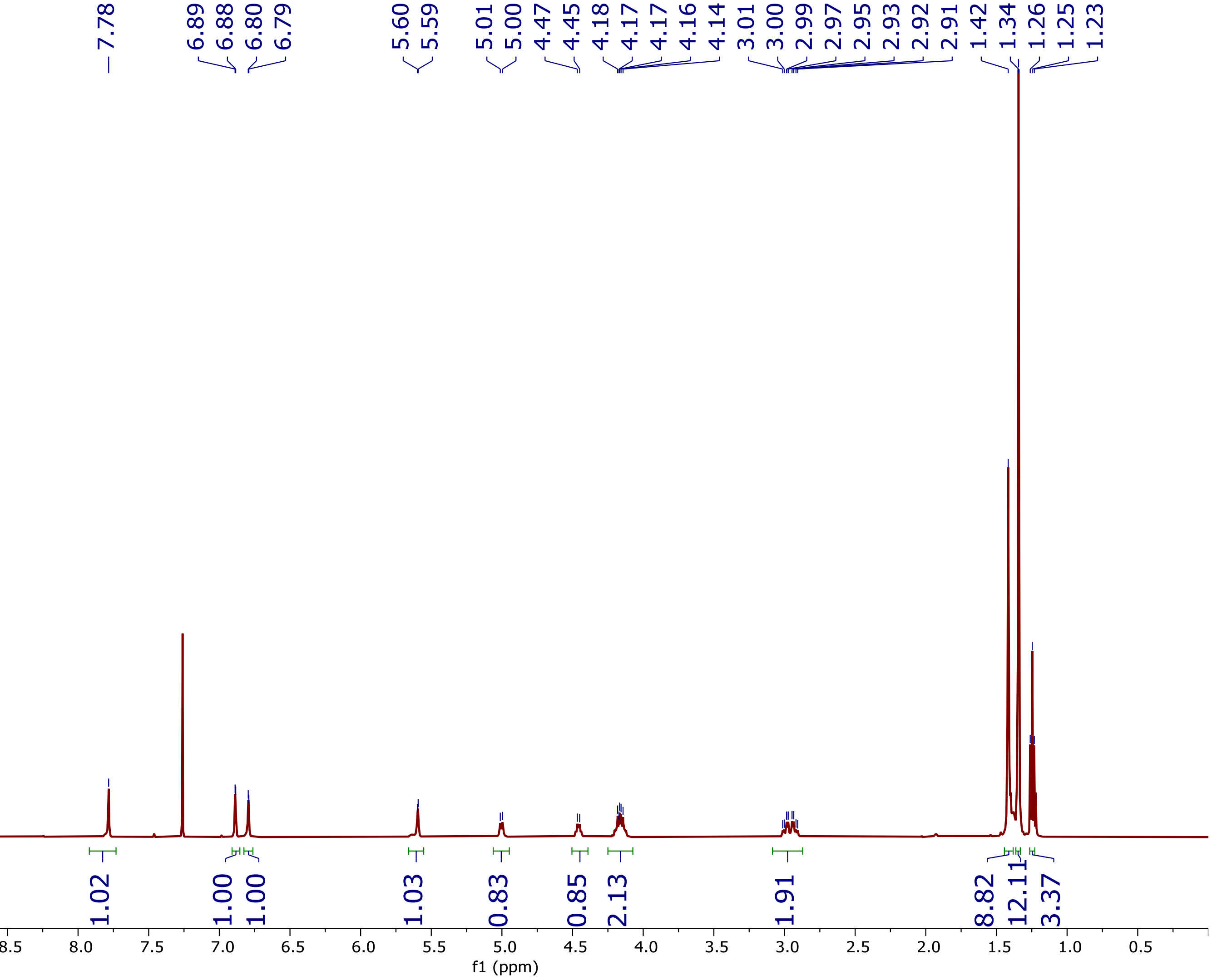

$^{13}C\{^1H\}$-NMR of ethyl (*S*)-2-((*tert*-butoxycarbonyl)amino)-3-(3,4-dihydroxy-5-(4,4,5,5-tetramethyl-1,3,2-dioxaborolan-2-yl) phenyl)propanoate (12) – (126 MHz, $CDCl_3$)

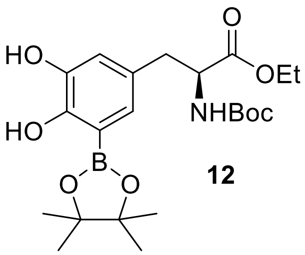

12

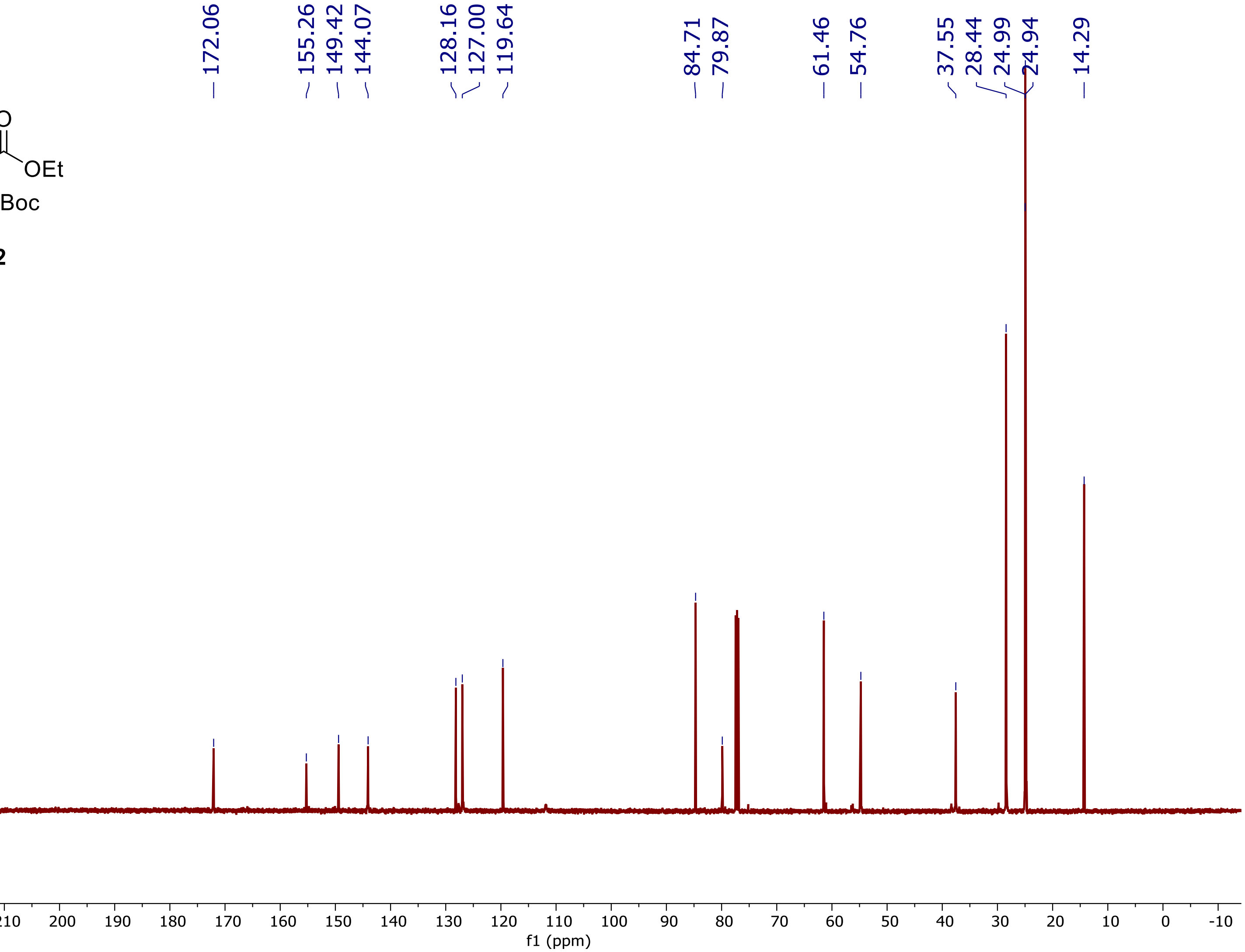

^{11}B-NMR of ethyl (*S*)-2-((*tert*-butoxycarbonyl)amino)-3-(3,4-dihydroxy-5-(4,4,5,5-tetramethyl-1,3,2-dioxaborolan-2-yl) phenyl)propanoate (12) – (160 MHz, $CDCl_3$)

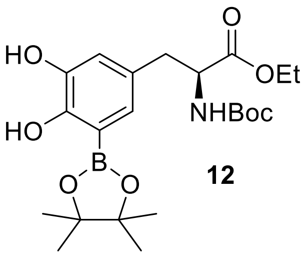

12

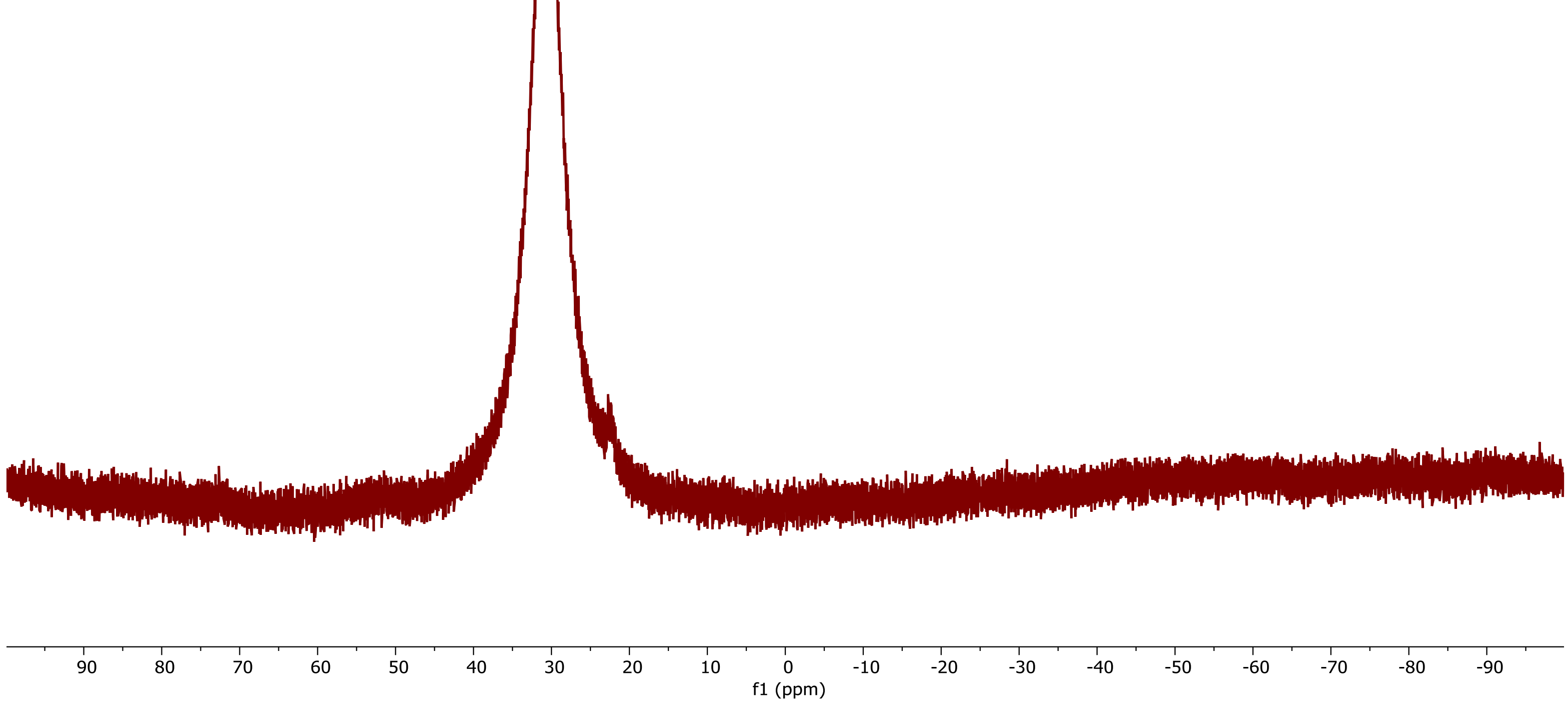

HSQC of ethyl (*S*)-2-((*tert*-butoxycarbonyl)amino)-3-(3,4-dihydroxy-5-(4,4,5,5-tetramethyl-1,3,2-dioxaborolan-2-yl) phenyl)propanoate (12) – ($CDCl_3$)

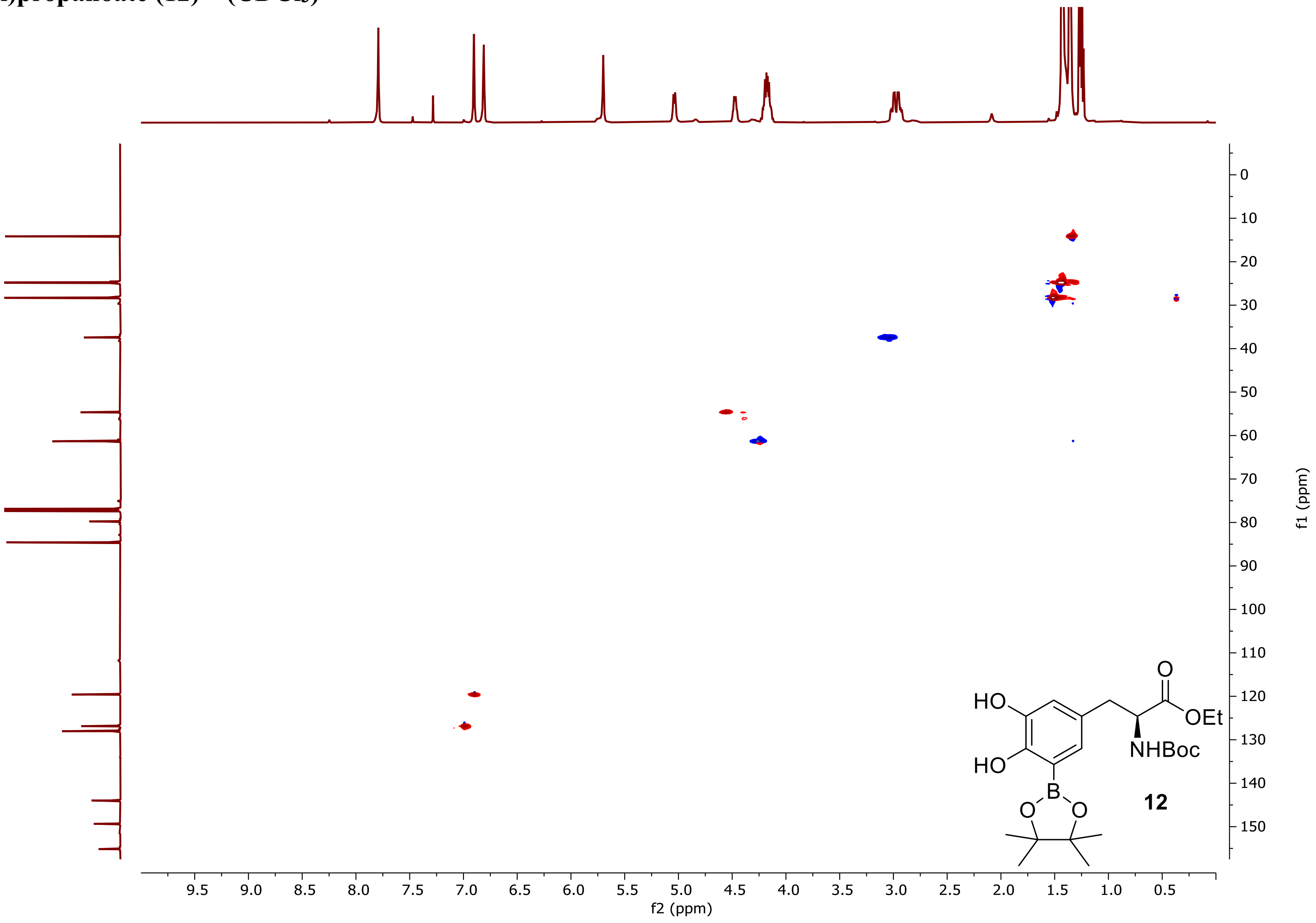

HSQC of ethyl (*S*)-2-((*tert*-butoxycarbonyl)amino)-3-(3,4-dihydroxy-5-(4,4,5,5-tetramethyl-1,3,2-dioxaborolan-2-yl)phenyl)propanoate (12) – ($CDCl_3$) – 5.5 to 7.5 ppm

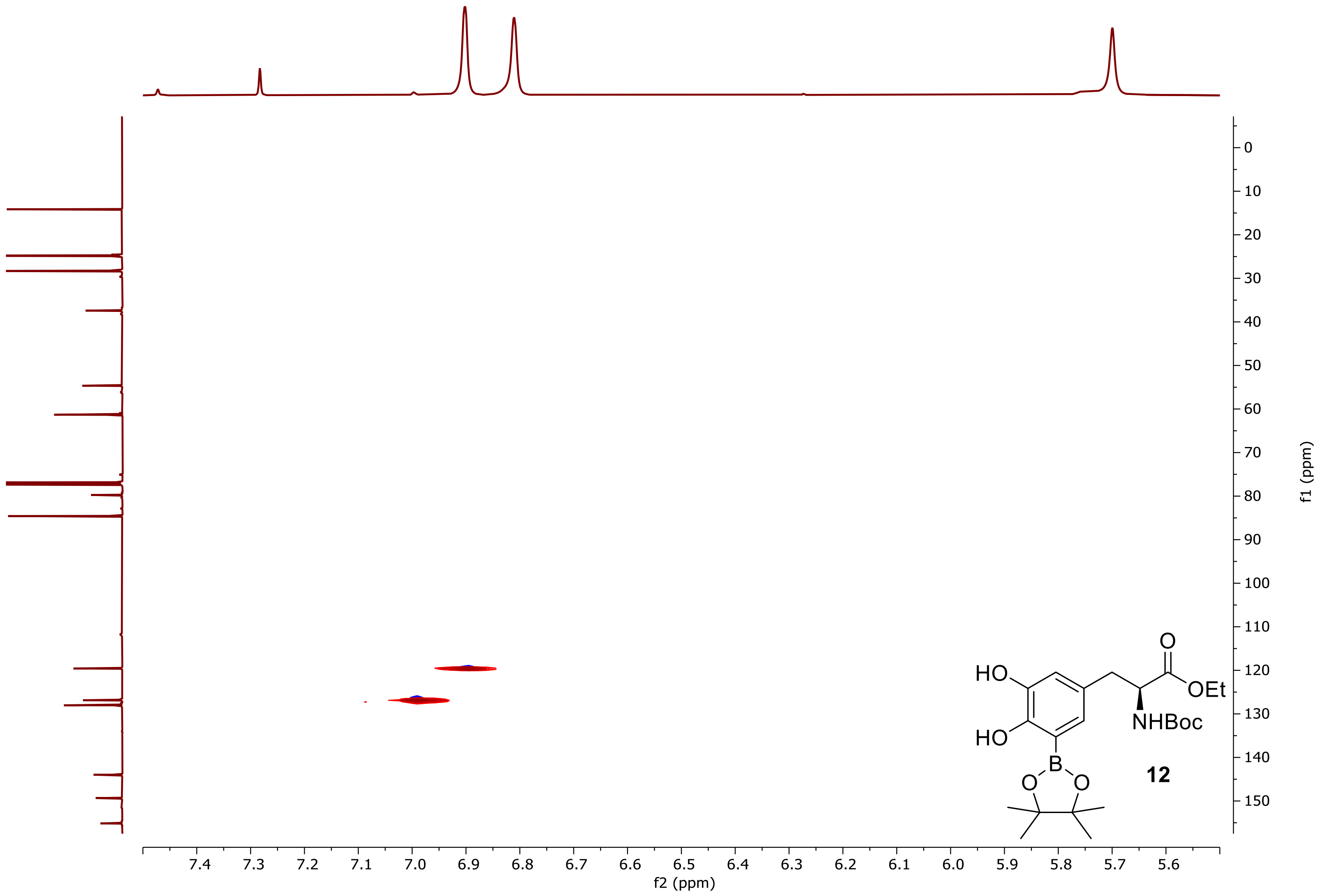

HMBC of ethyl (*S*)-2-((*tert*-butoxycarbonyl)amino)-3-(3,4-dihydroxy-5-(4,4,5,5-tetramethyl-1,3,2-dioxaborolan-2-yl)phenyl)propanoate (12) – ($CDCl_3$)

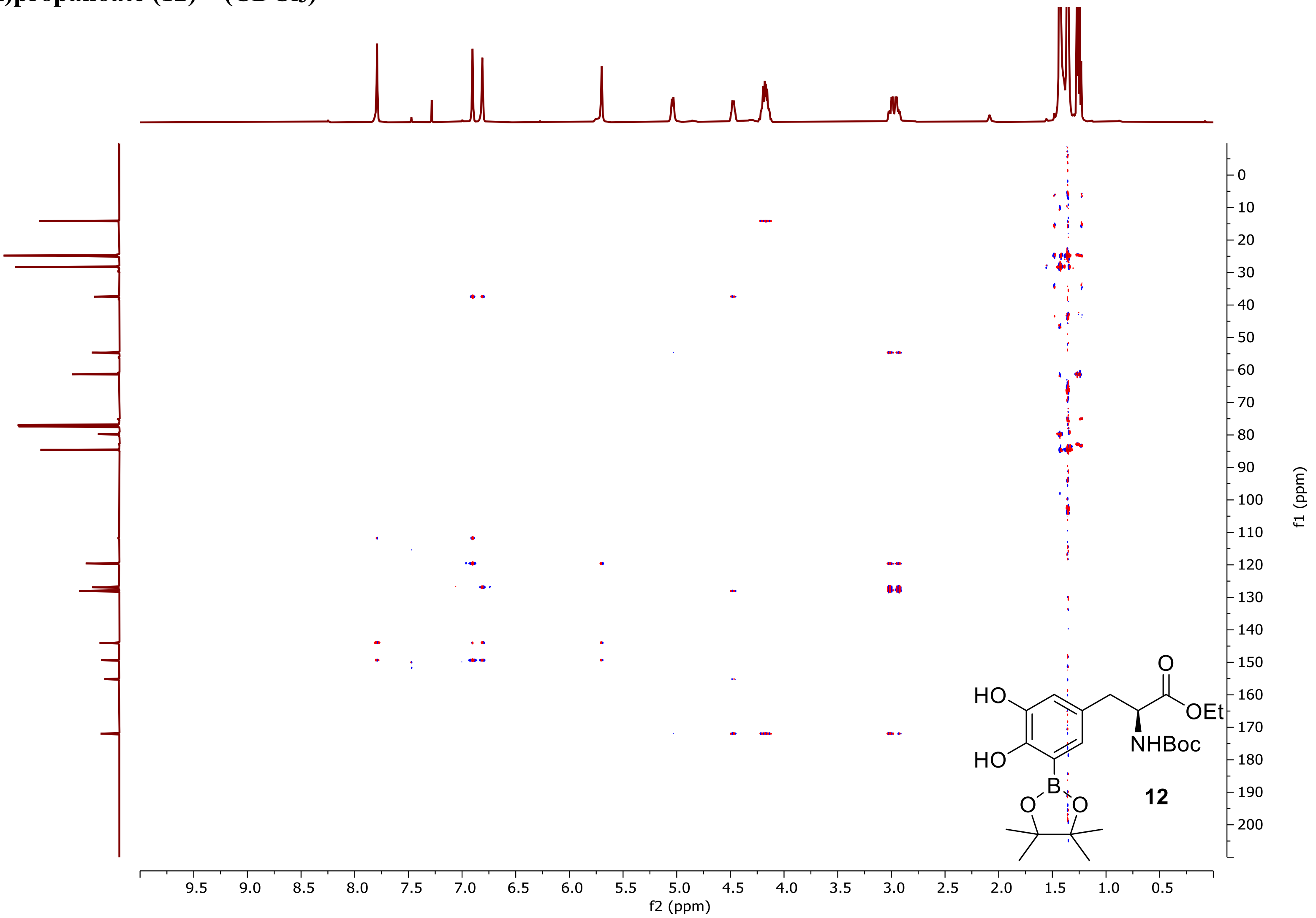

HMBC of ethyl (*S*)-2-((*tert*-butoxycarbonyl)amino)-3-(3,4-dihydroxy-5-(4,4,5,5-tetramethyl-1,3,2-dioxaborolan-2-yl) phenyl)propanoate (12) – ($CDCl_3$) – 5.5 to 7.5 ppm

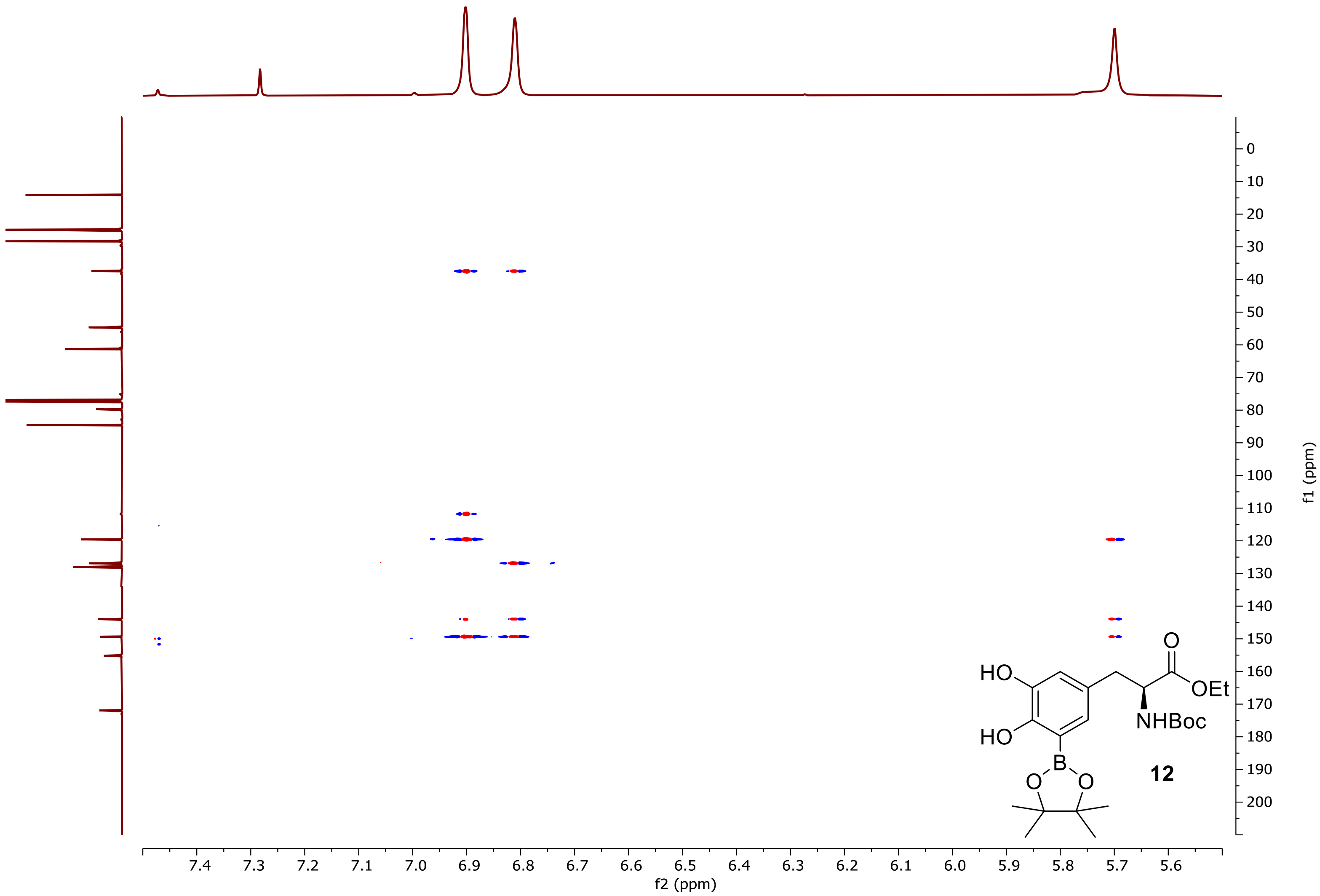

^{1}H-NMR of ethyl (*S*)-3-(benzo[*d*][1,3]dioxol-5-yl-7-d)-2-((tert-butoxycarbonyl)amino) propanoate (13) – (500 MHz, CDCl₃)

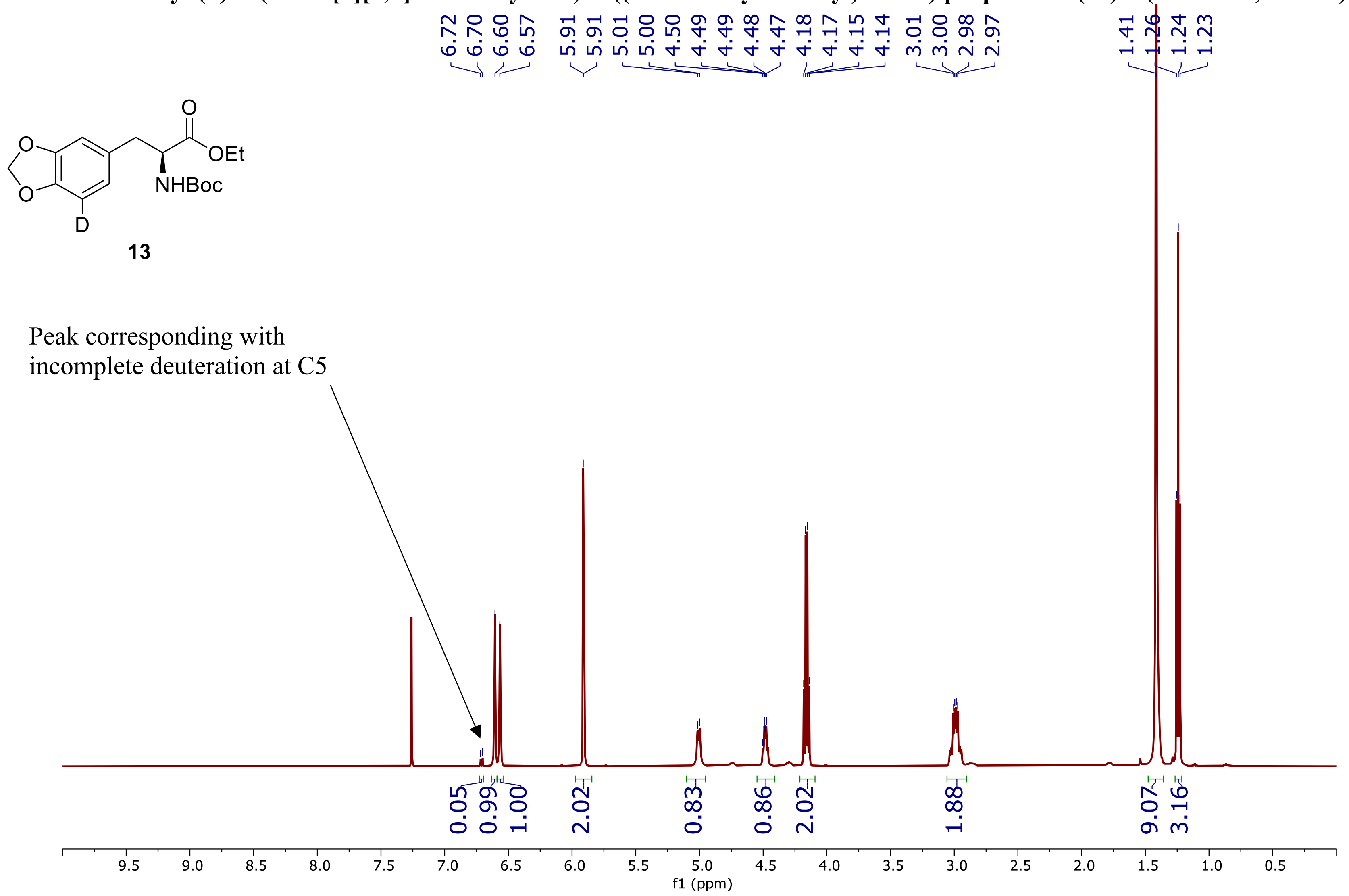

^{1}H-NMR of ethyl (*S*)-3-(benzo[*d*][1,3]dioxol-5-yl-7-d)-2-((tert-butoxycarbonyl)amino) propanoate (13) – (500 MHz, $CDCl_3$) – 5.5 to 7.5 ppm

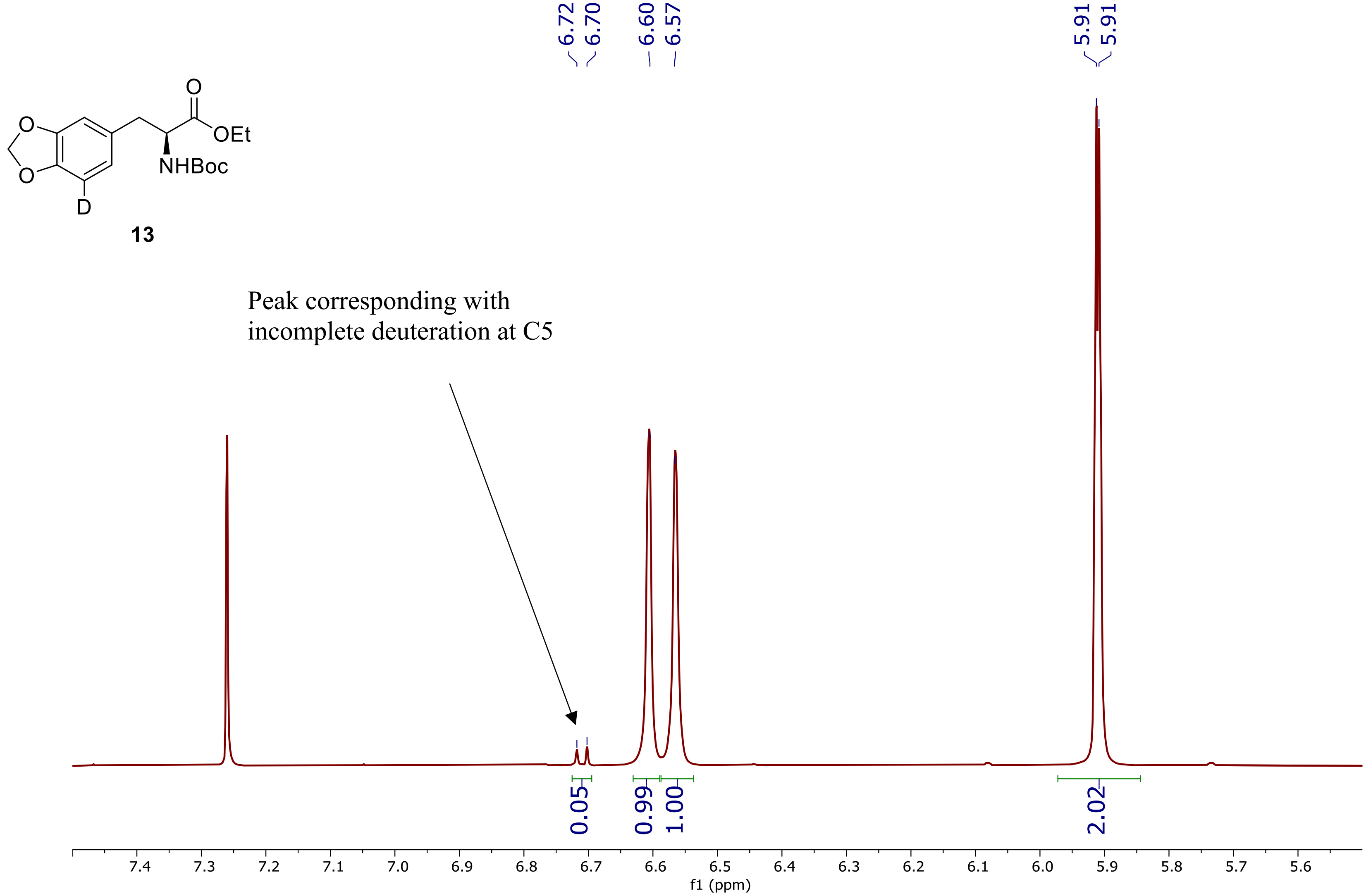

$^{13}C\{^{1}H\}$-NMR of ethyl (*S*)-3-(benzo[*d*][1,3]dioxol-5-yl-7-d)-2-((tert-butoxycarbonyl)amino) propanoate (13) – (126 MHz, $CDCl_3$)

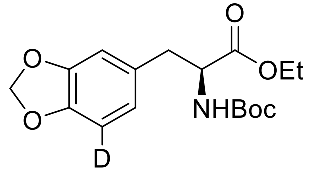

13

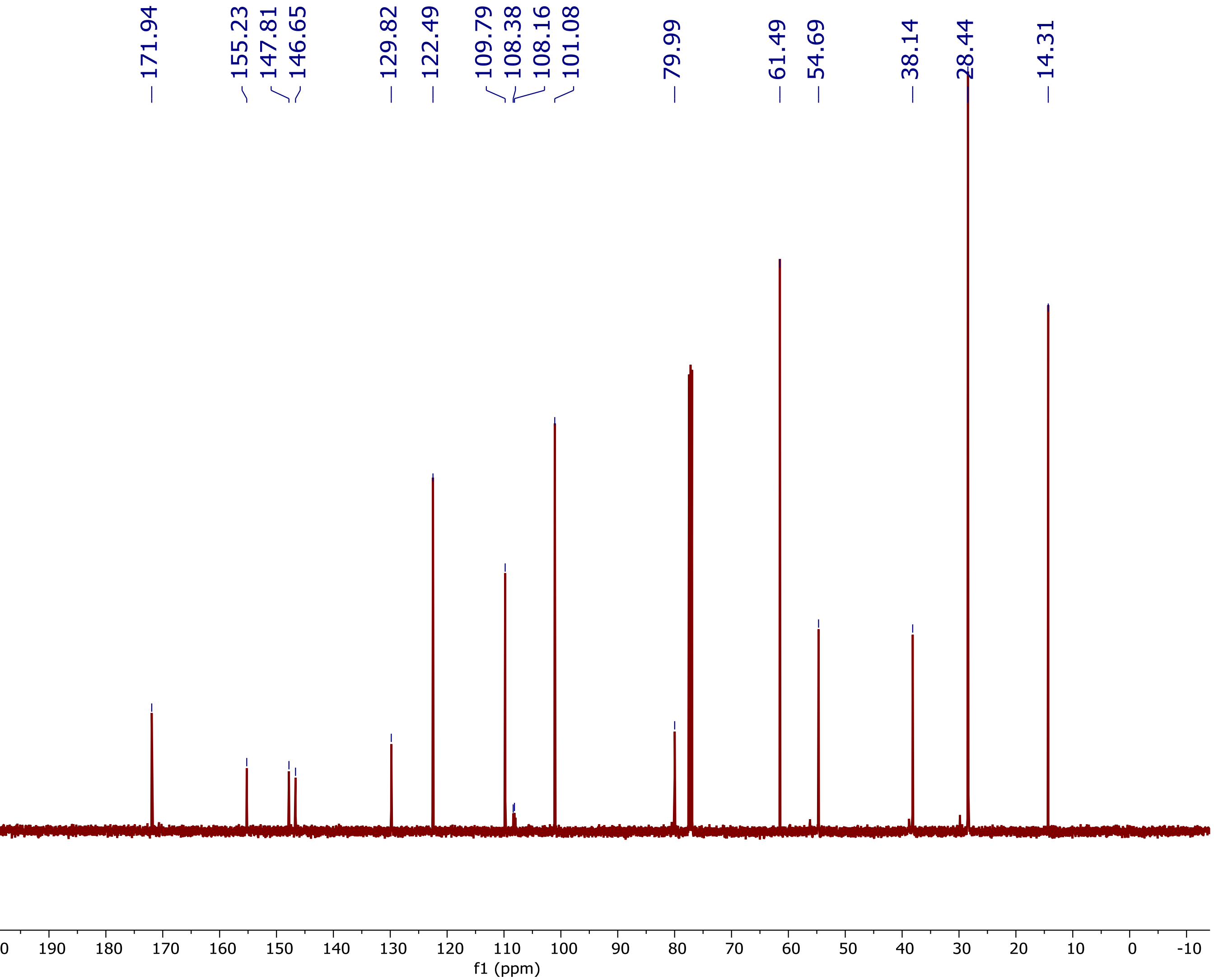

^{1}H-NMR of (*S*)-1-ethoxy-1-oxo-3-(7-(4,4,5,5-tetramethyl-1,3,2-dioxaborolan-2-yl) benzo[*d*][1,3]dioxol-5-yl)propan-2-aminium trifluoroacetate (14) – (500 MHz, $CDCl_3$)

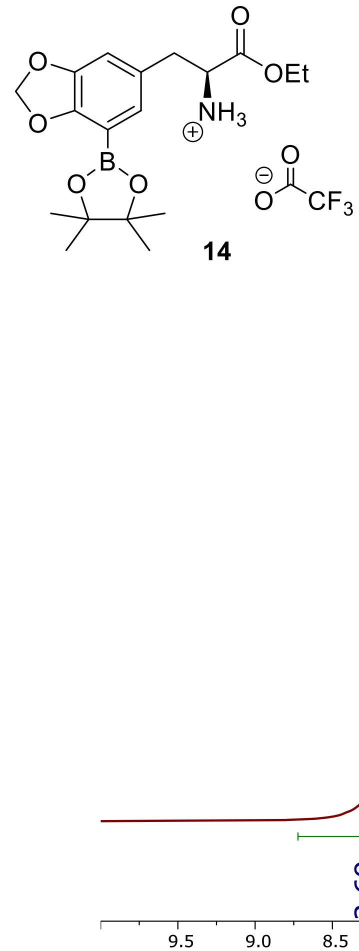

14

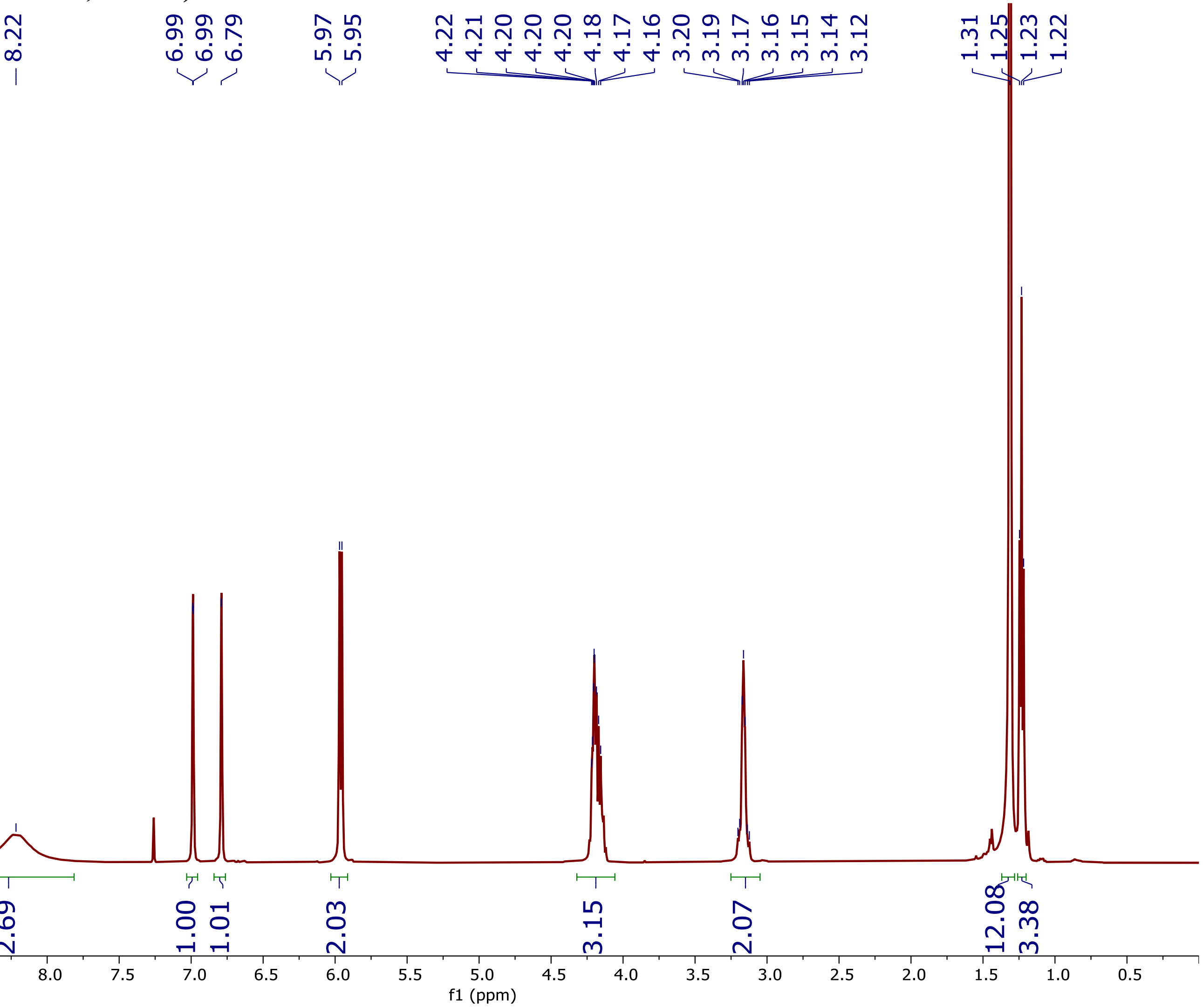

^{13}C{^{1}H}-NMR of (*S*)-1-ethoxy-1-oxo-3-(7-(4,4,5,5-tetramethyl-1,3,2-dioxaborolan-2-yl) benzo[*d*][1,3]dioxol-5-yl)propan-2-aminium trifluoroacetate (14) – (126 MHz, CDCl₃)

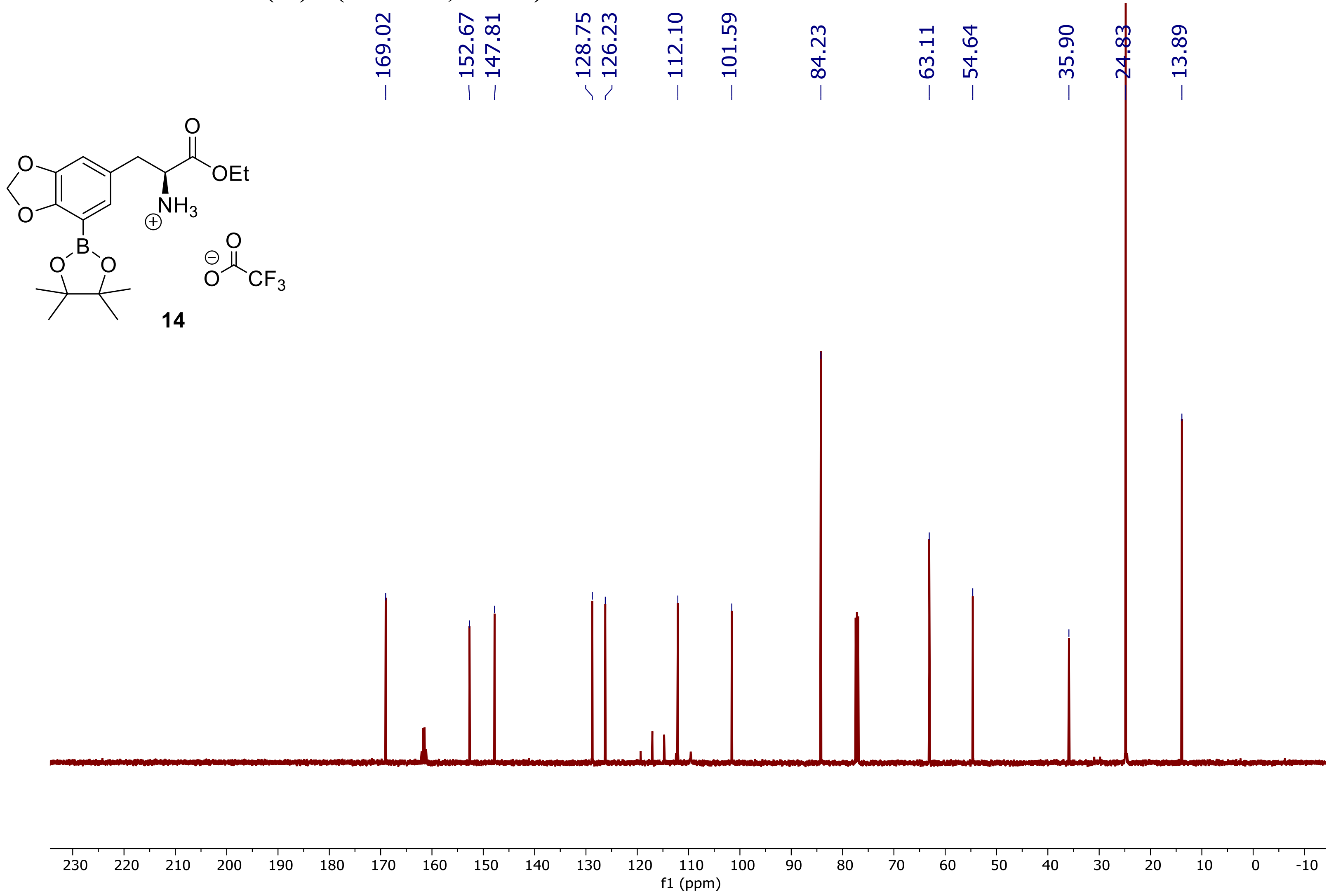

^{11}B-NMR of (*S*)-1-ethoxy-1-oxo-3-(7-(4,4,5,5-tetramethyl-1,3,2-dioxaborolan-2-yl) benzo[*d*][1,3]dioxol-5-yl)propan-2-aminium trifluoroacetate (14) – (160 MHz, CDCl3)

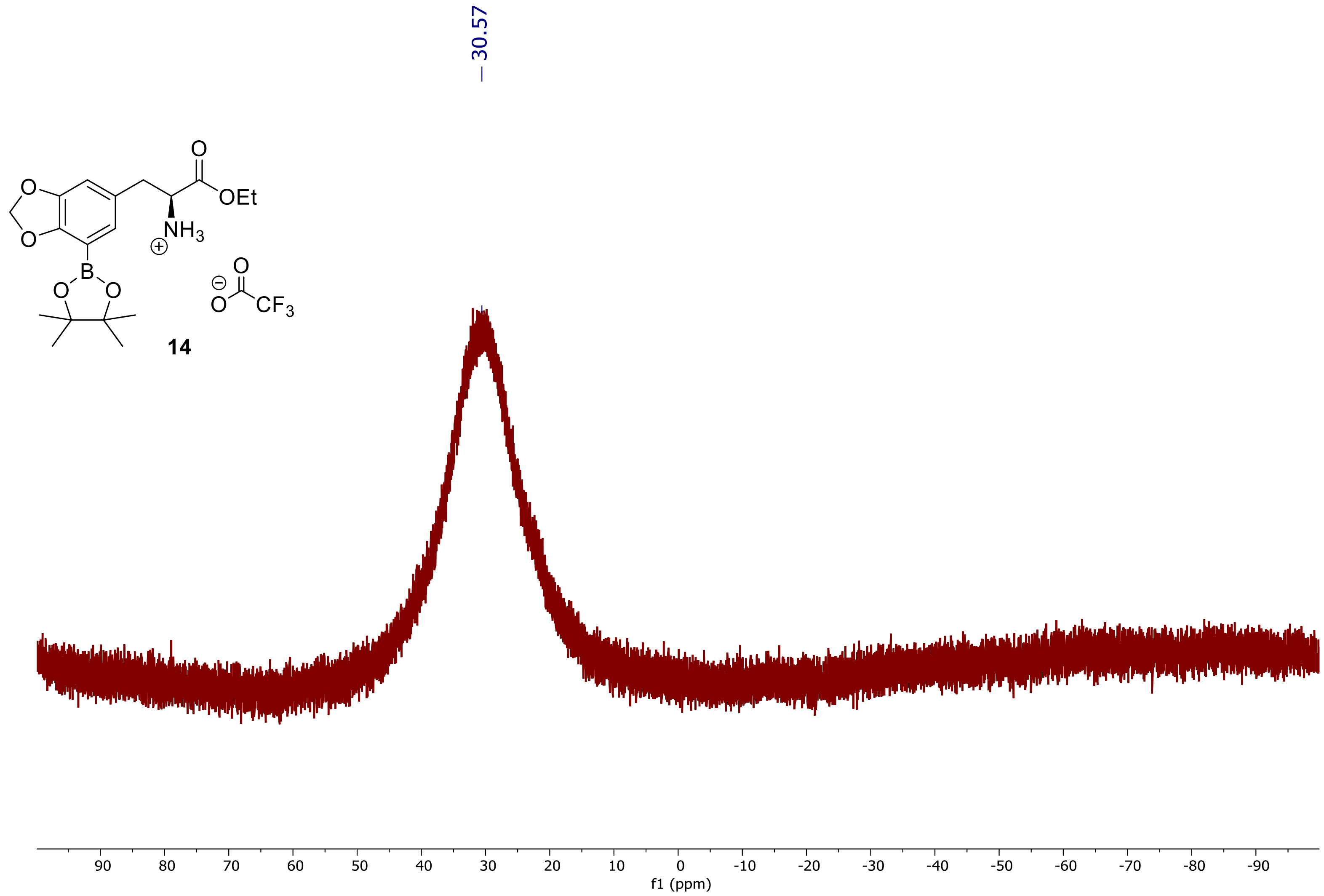

^{19}F-NMR of (*S*)-1-ethoxy-1-oxo-3-(7-(4,4,5,5-tetramethyl-1,3,2-dioxaborolan-2-yl) benzo[*d*][1,3]dioxol-5-yl)propan-2-aminium trifluoroacetate (14) – (470 MHz, CDCl₃)

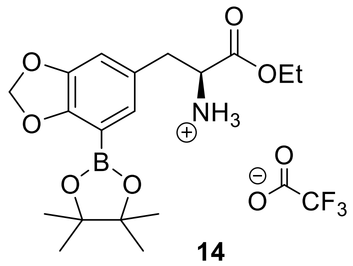

14

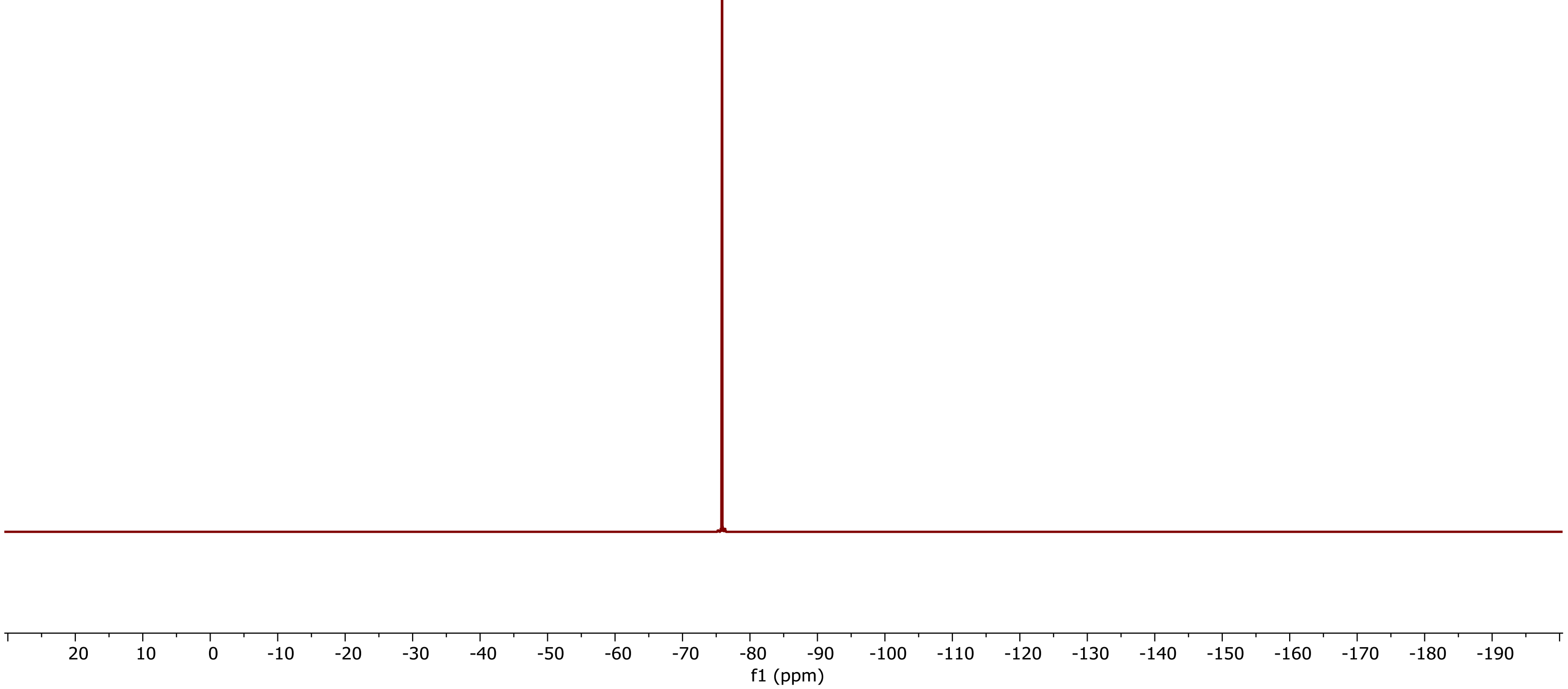

^{1}H-NMR of ethyl (*S*)-2-((*tert*-butoxycarbonyl)amino)-3-(7-hydroxybenzo[*d*][1,3]dioxol-5-yl)propanoate (15) – (500 MHz, $CDCl_3$)

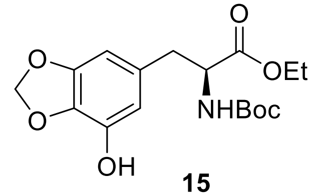

15

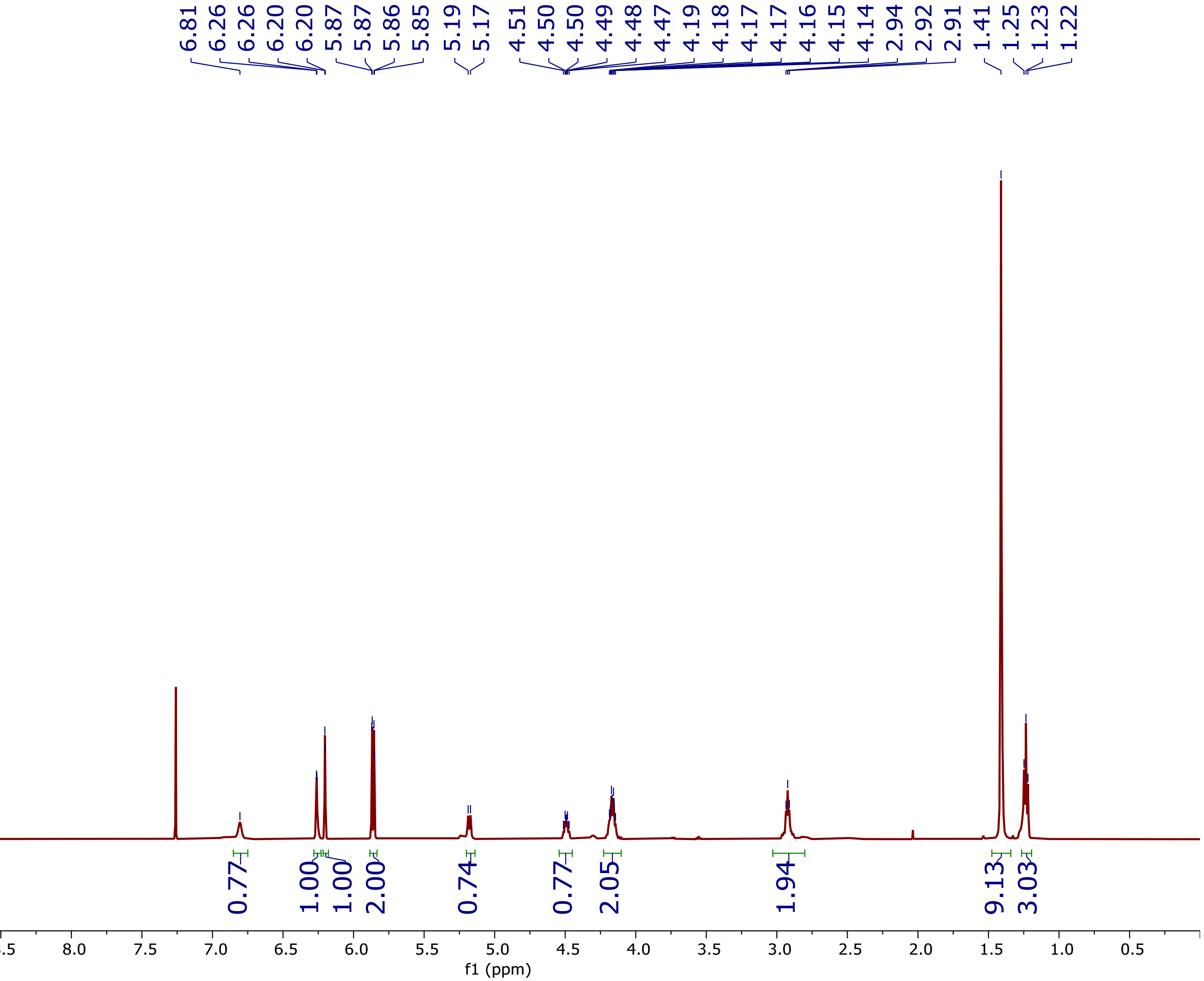

^{13}C-NMR of ethyl (*S*)-2-((*tert*-butoxycarbonyl)amino)-3-(7-hydroxybenzo[*d*][1,3]dioxol-5-yl)propanoate (15) – (126 MHz, $CDCl_3$)

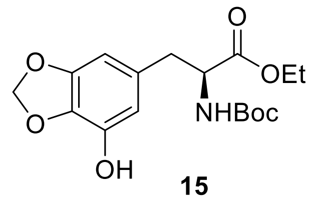

15

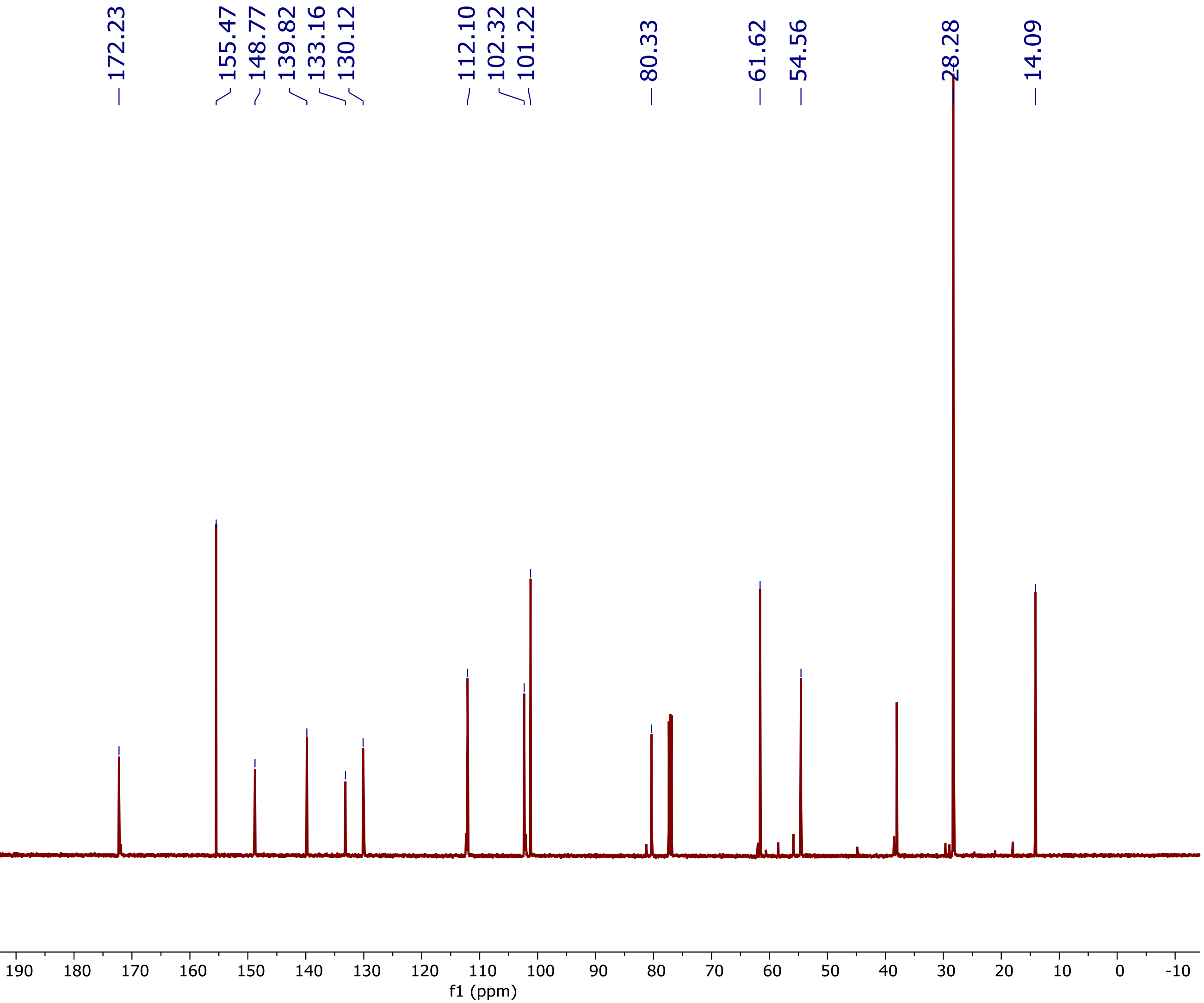

HSQC of ethyl (*S*)-2-((*tert*-butoxycarbonyl)amino)-3-(7-hydroxybenzo[*d*][1,3]dioxol-5-yl)propanoate (15) – ($CDCl_3$)

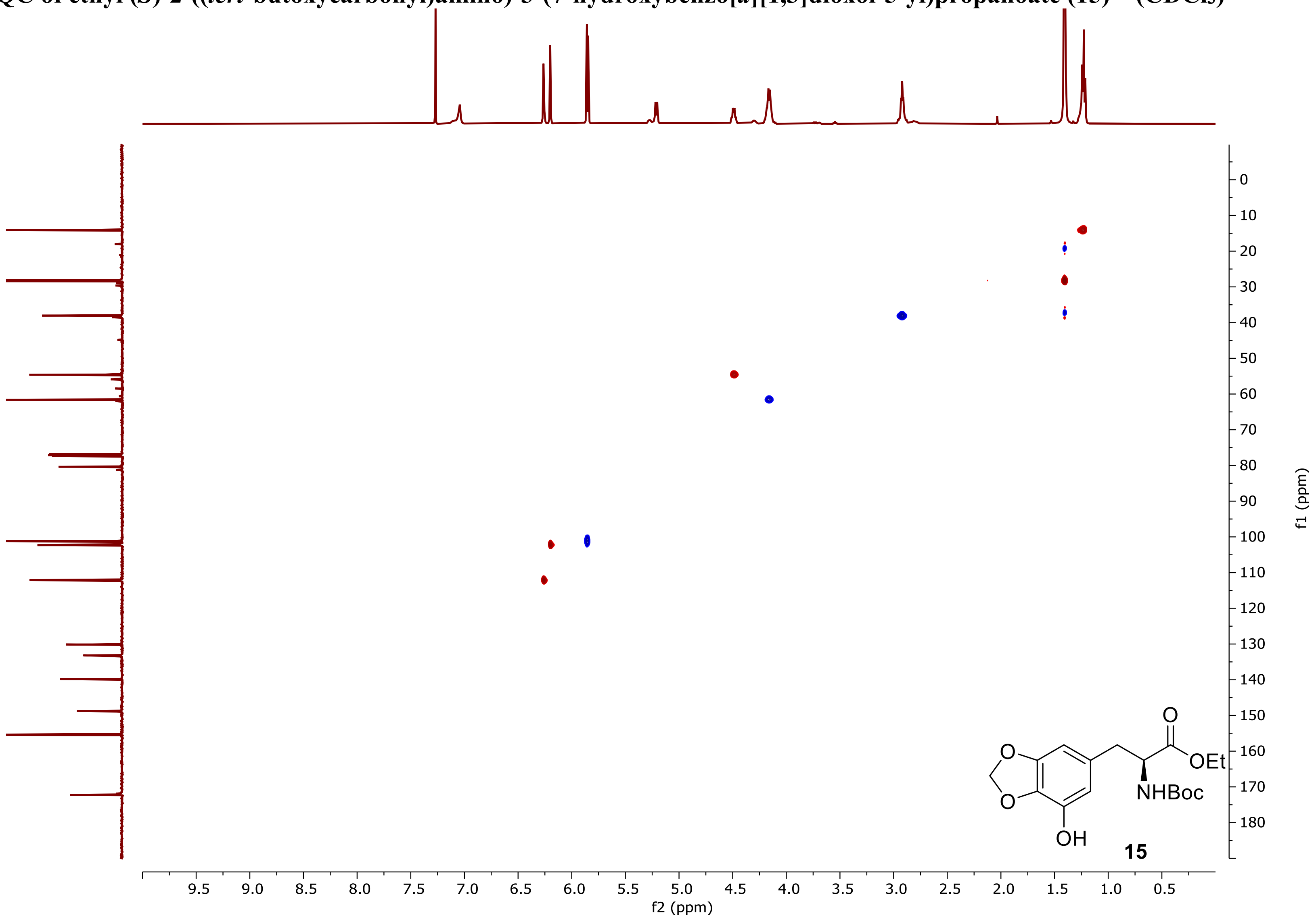

HSQC of ethyl (*S*)-2-((*tert*-butoxycarbonyl)amino)-3-(7-hydroxybenzo[*d*][1,3]dioxol-5-yl)propanoate (15) – ($CDCl_3$) – 5.5 to 7.5 ppm

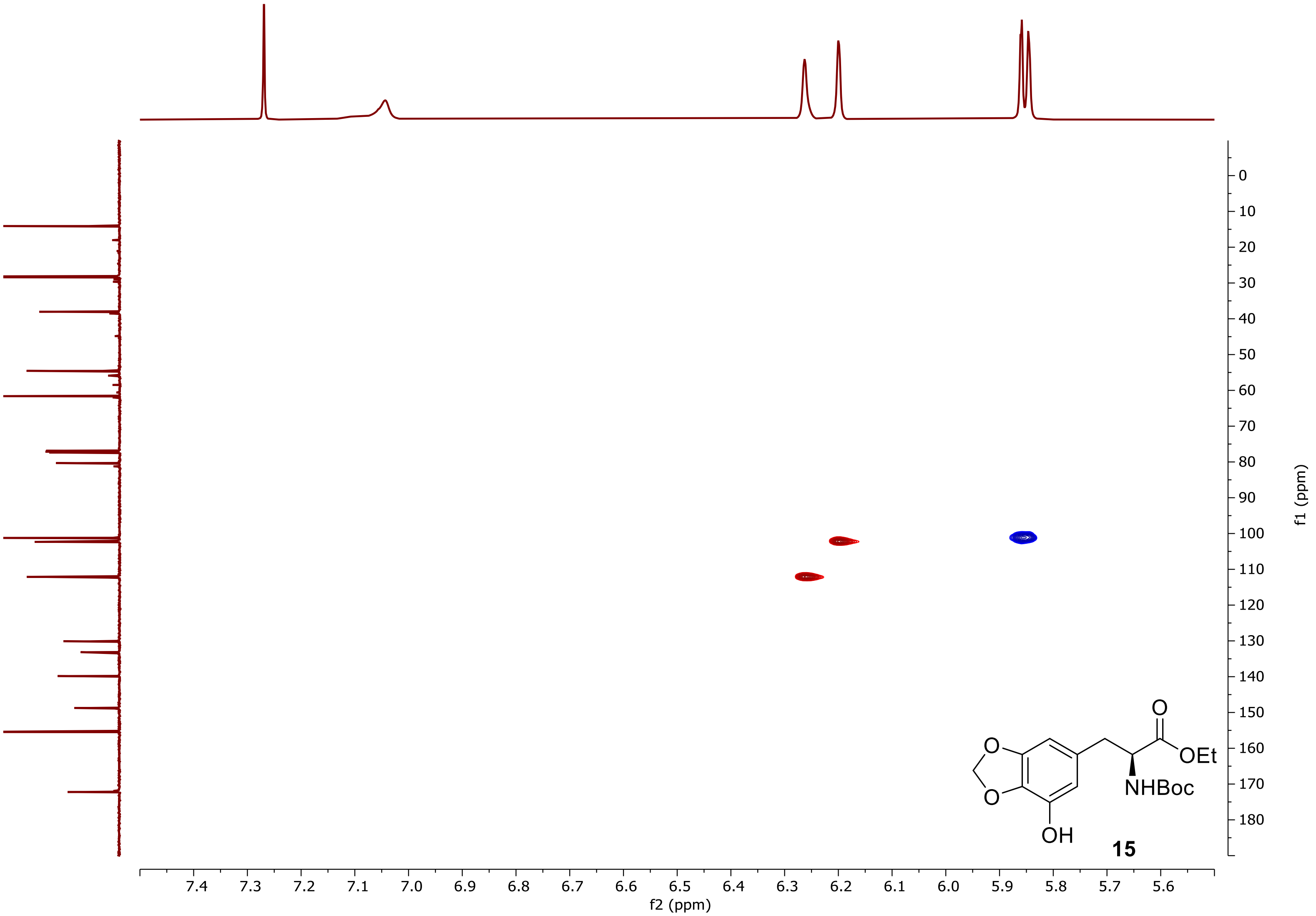

HMBC of ethyl (*S*)-2-((*tert*-butoxycarbonyl)amino)-3-(7-hydroxybenzo[*d*][1,3]dioxol-5-yl)propanoate (15) – ($CDCl_3$)

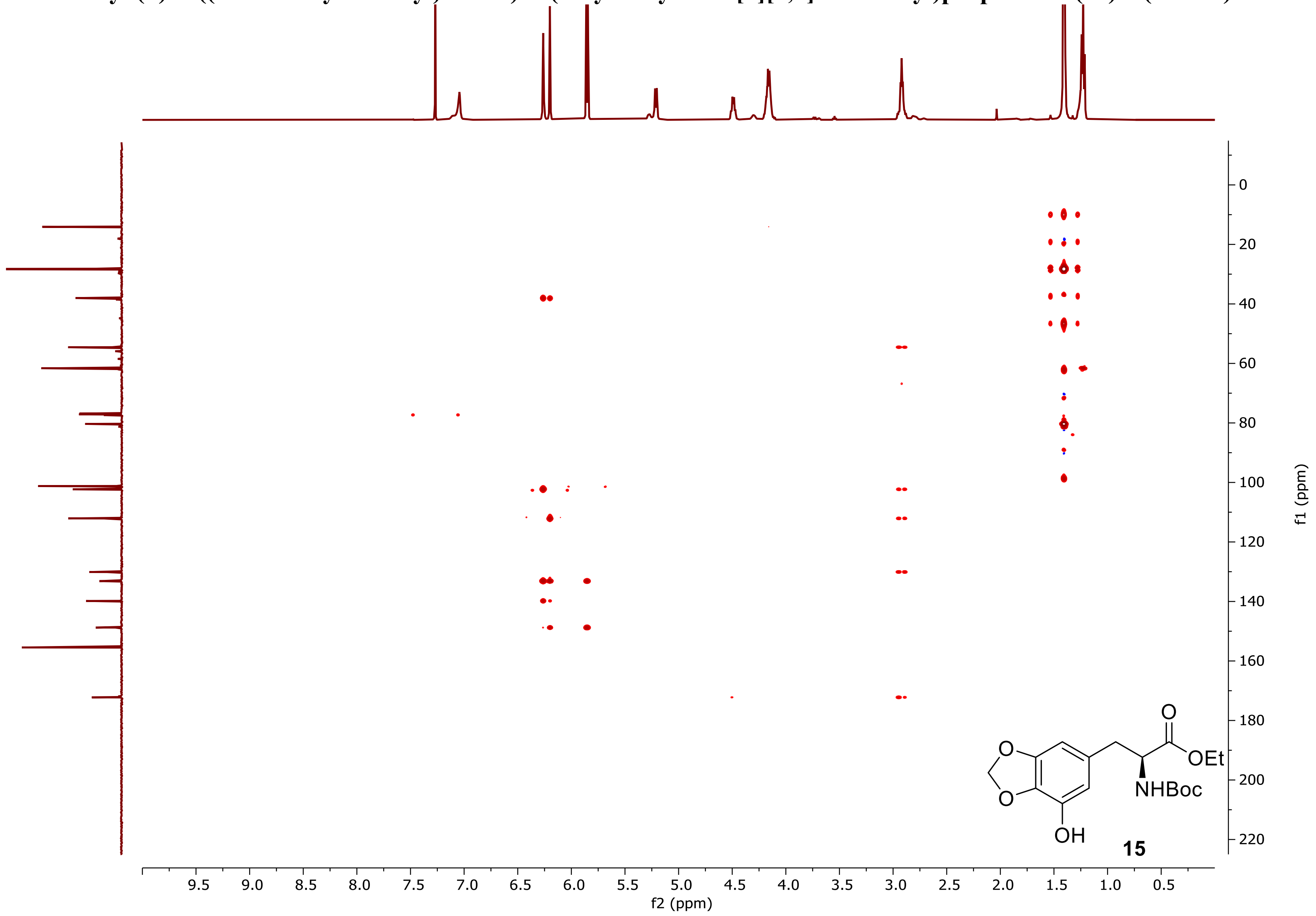

HMBC of ethyl (*S*)-2-((*tert*-butoxycarbonyl)amino)-3-(7-hydroxybenzo[*d*][1,3]dioxol-5-yl)propanoate (15) – ($CDCl_3$) – 5.5 to 7.5 ppm

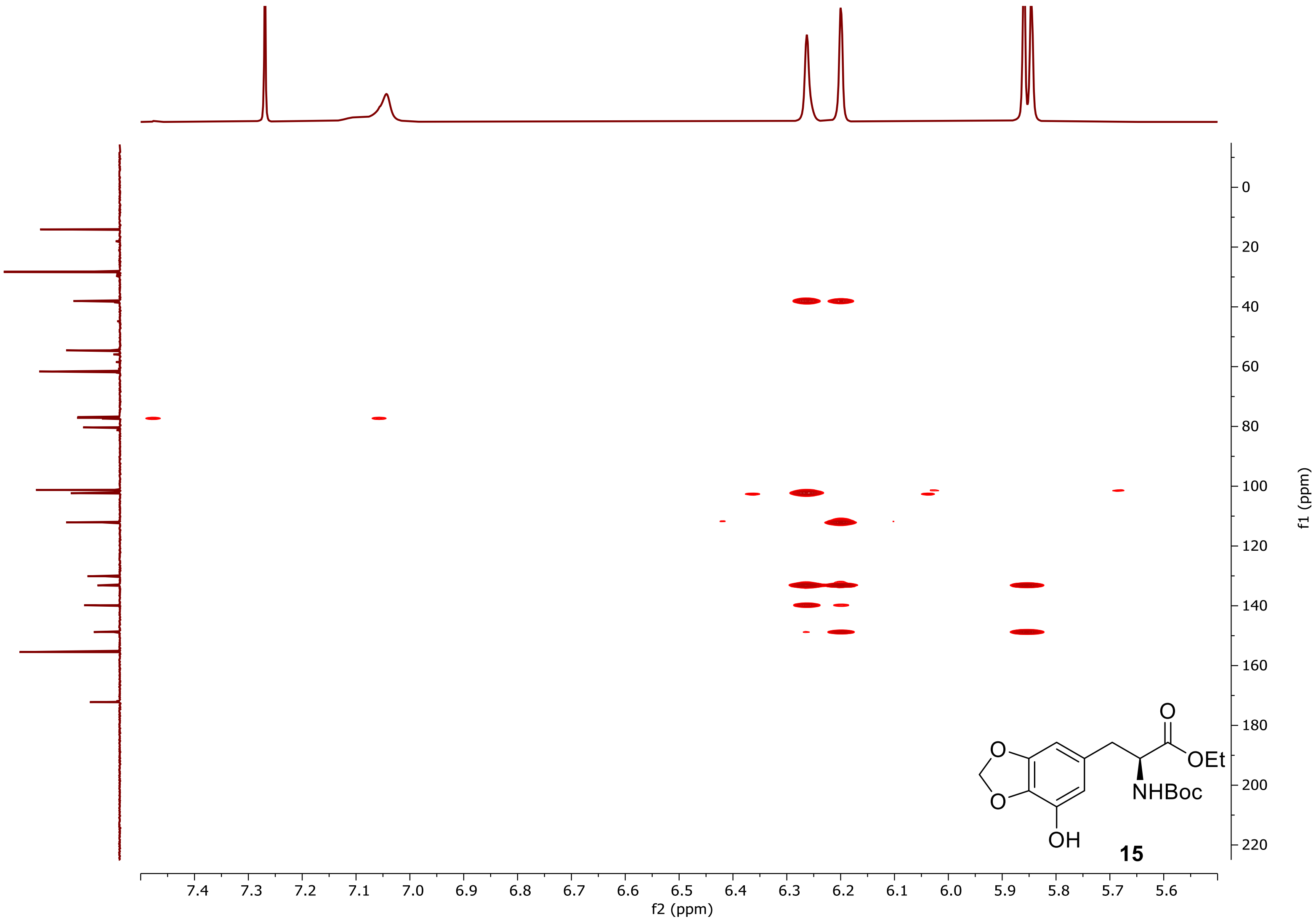

^{1}H-NMR of ethyl (*S*)-4-(6-(2-((*tert*-butoxycarbonyl)amino)-3-ethoxy-3-oxopropyl) benzo[*d*][1,3]dioxol-4-yl)benzoate (16) – (500 MHz, $CDCl_3$)

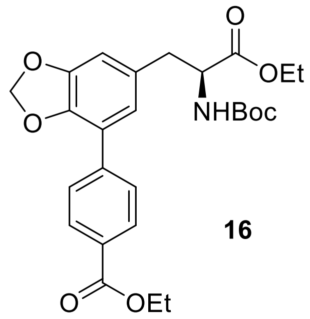

16

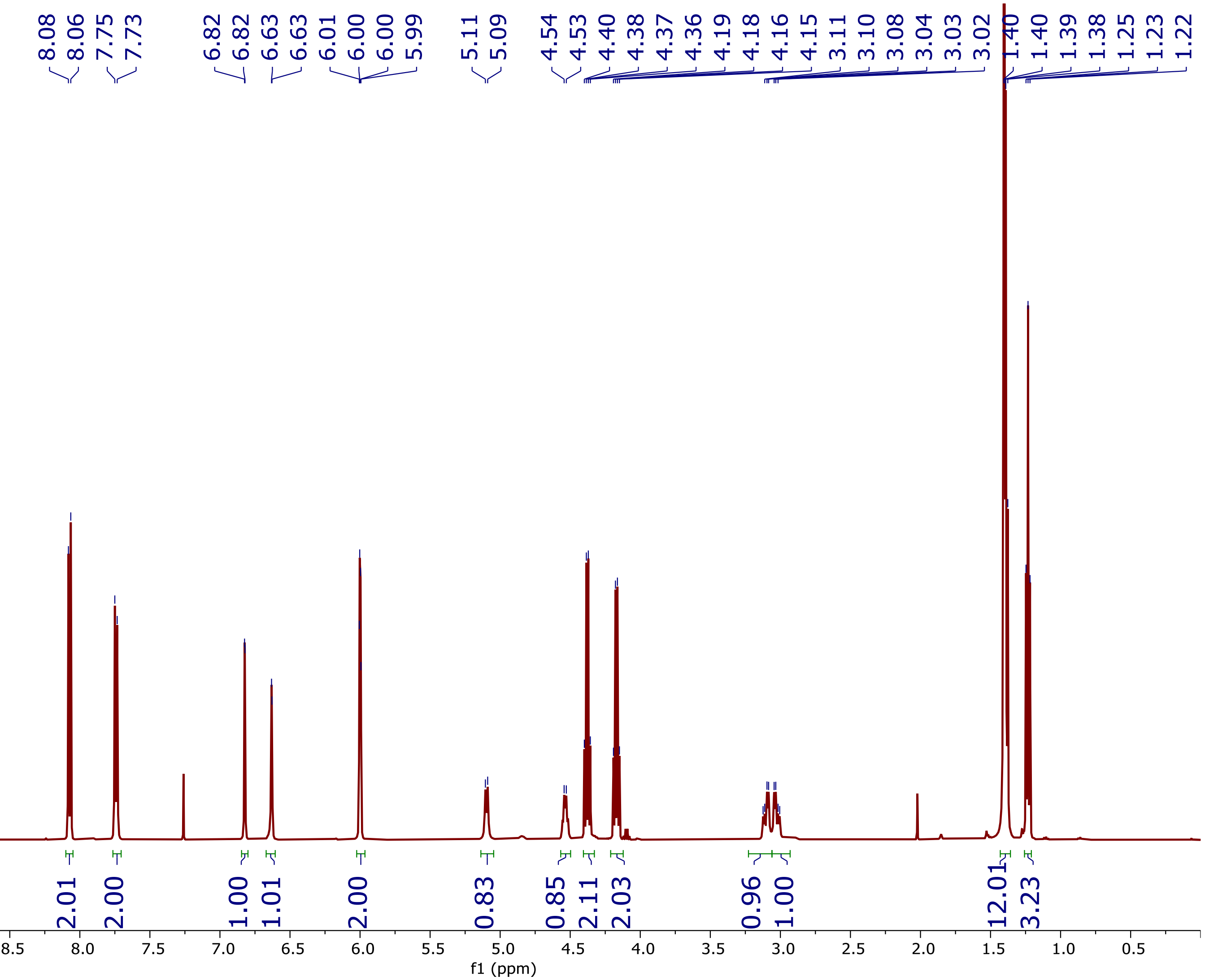

$^{13}C\{^{1}H\}$-NMR of ethyl (*S*)-4-(6-(2-((*tert*-butoxycarbonyl)amino)-3-ethoxy-3-oxopropyl) benzo[*d*][1,3]dioxol-4-yl)benzoate (16) – (126 MHz, $CDCl_3$)

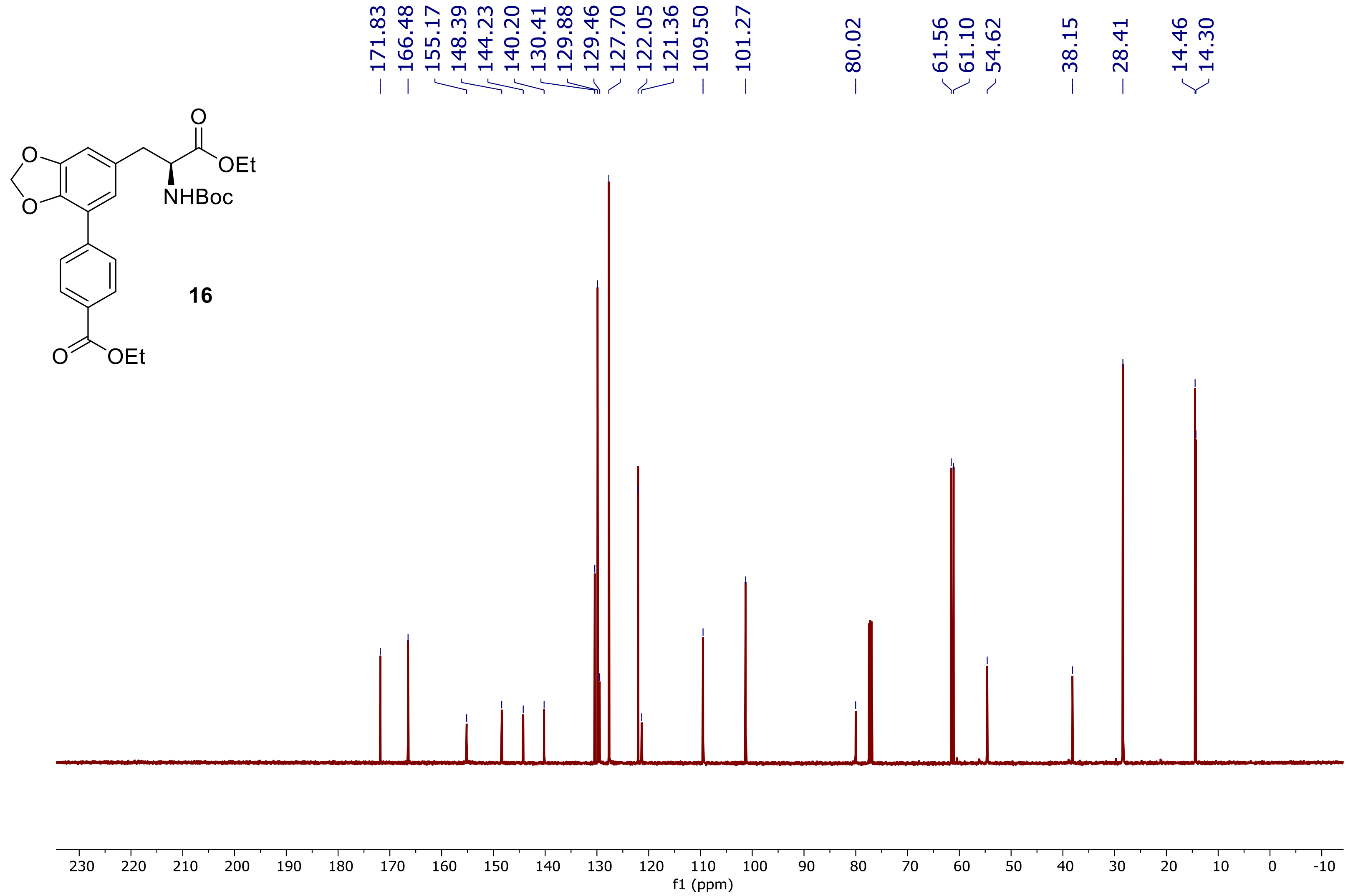

HSQC of ethyl (*S*)-4-(6-(2-((*tert*-butoxycarbonyl)amino)-3-ethoxy-3-oxopropyl) benzo[*d*][1,3]dioxol-4-yl)benzoate (16) – (CDCl$_3$)

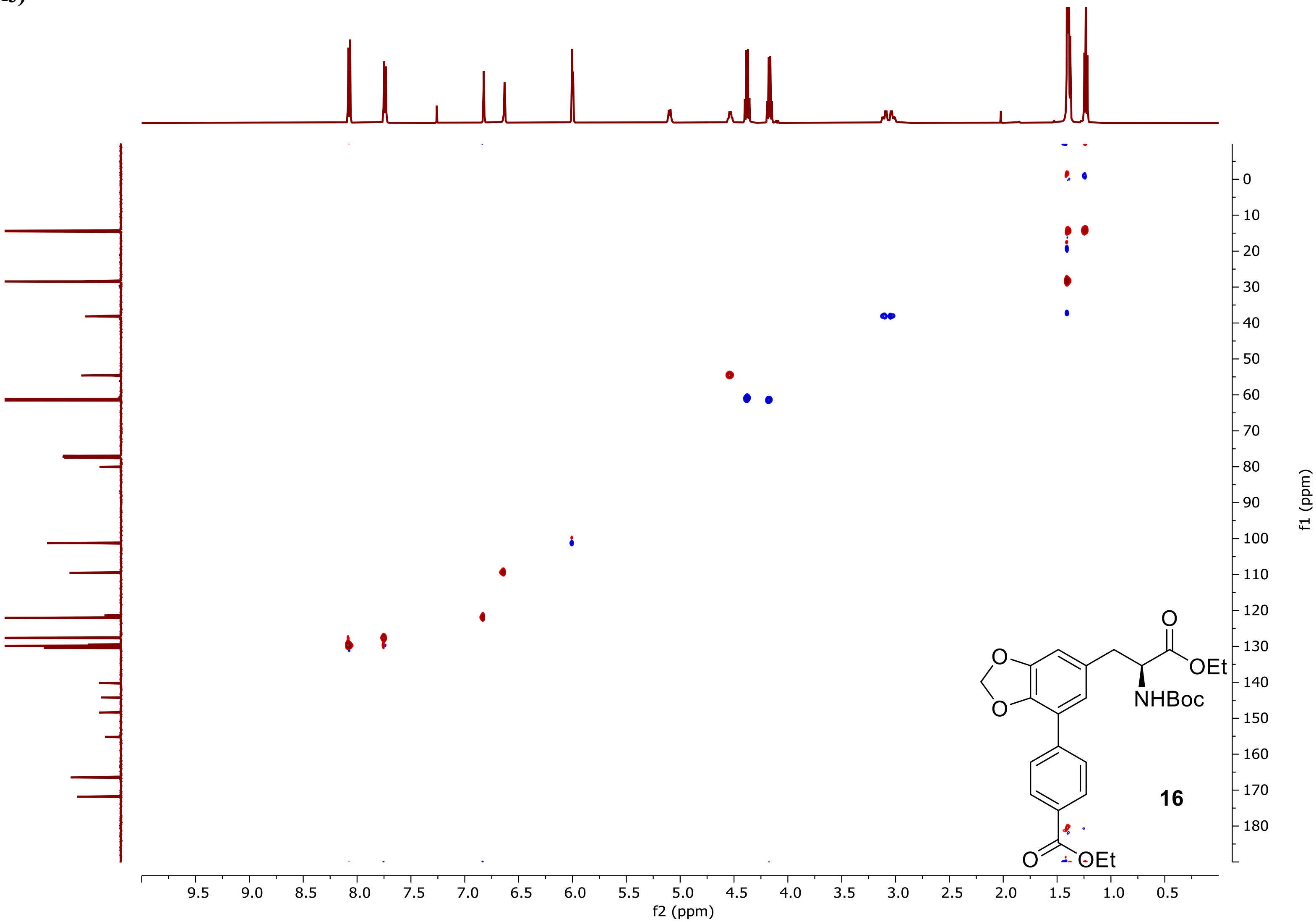

HSQC of ethyl (*S*)-4-(6-(2-((*tert*-butoxycarbonyl)amino)-3-ethoxy-3-oxopropyl) benzo[*d*][1,3]dioxol-4-yl)benzoate (16) – ($CDCl_3$) – 5.5 to 8.5 ppm

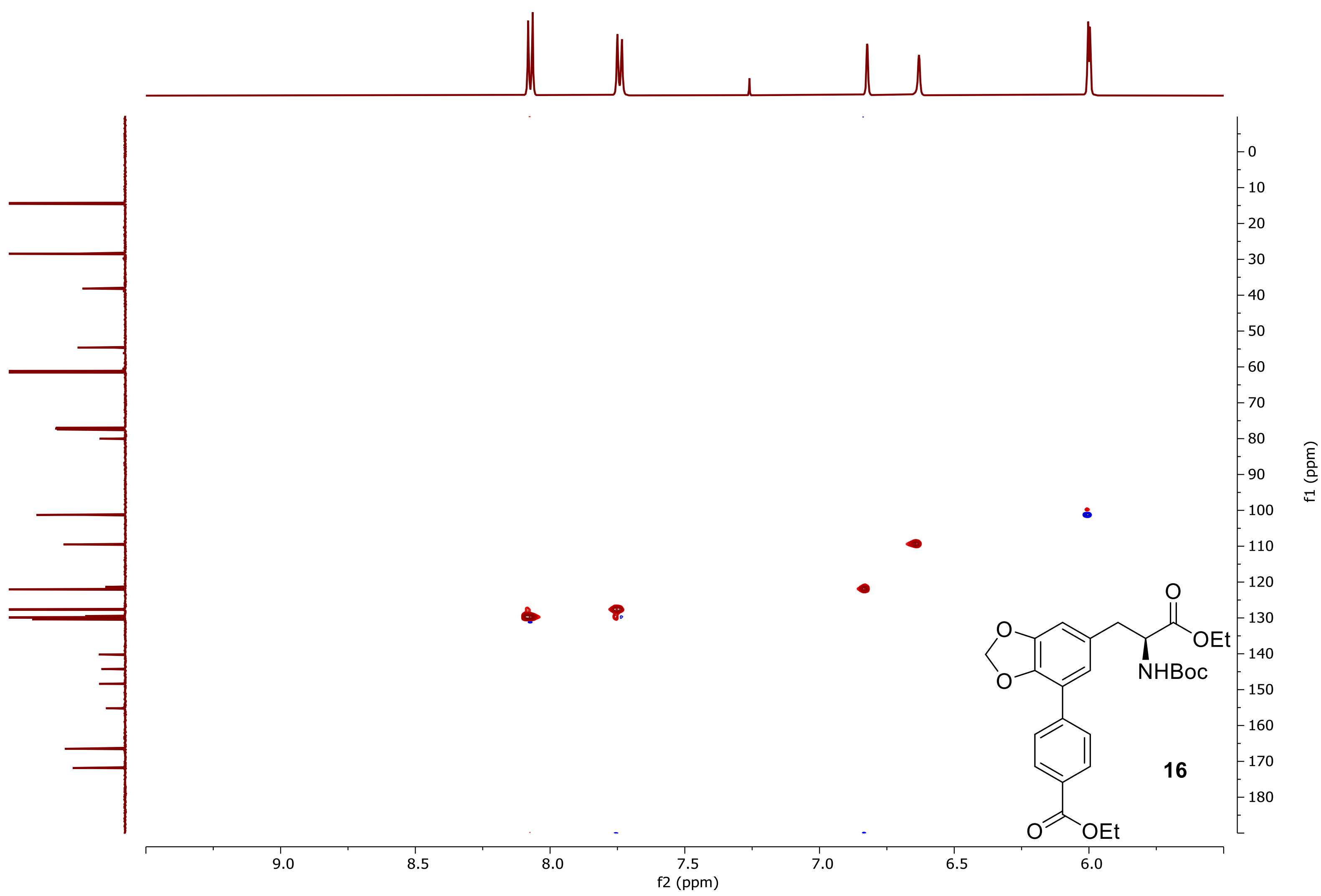

HMBC of ethyl (*S*)-4-(6-(2-((*tert*-butoxycarbonyl)amino)-3-ethoxy-3-oxopropyl) benzo[*d*][1,3]dioxol-4-yl)benzoate (16) – ($CDCl_3$)

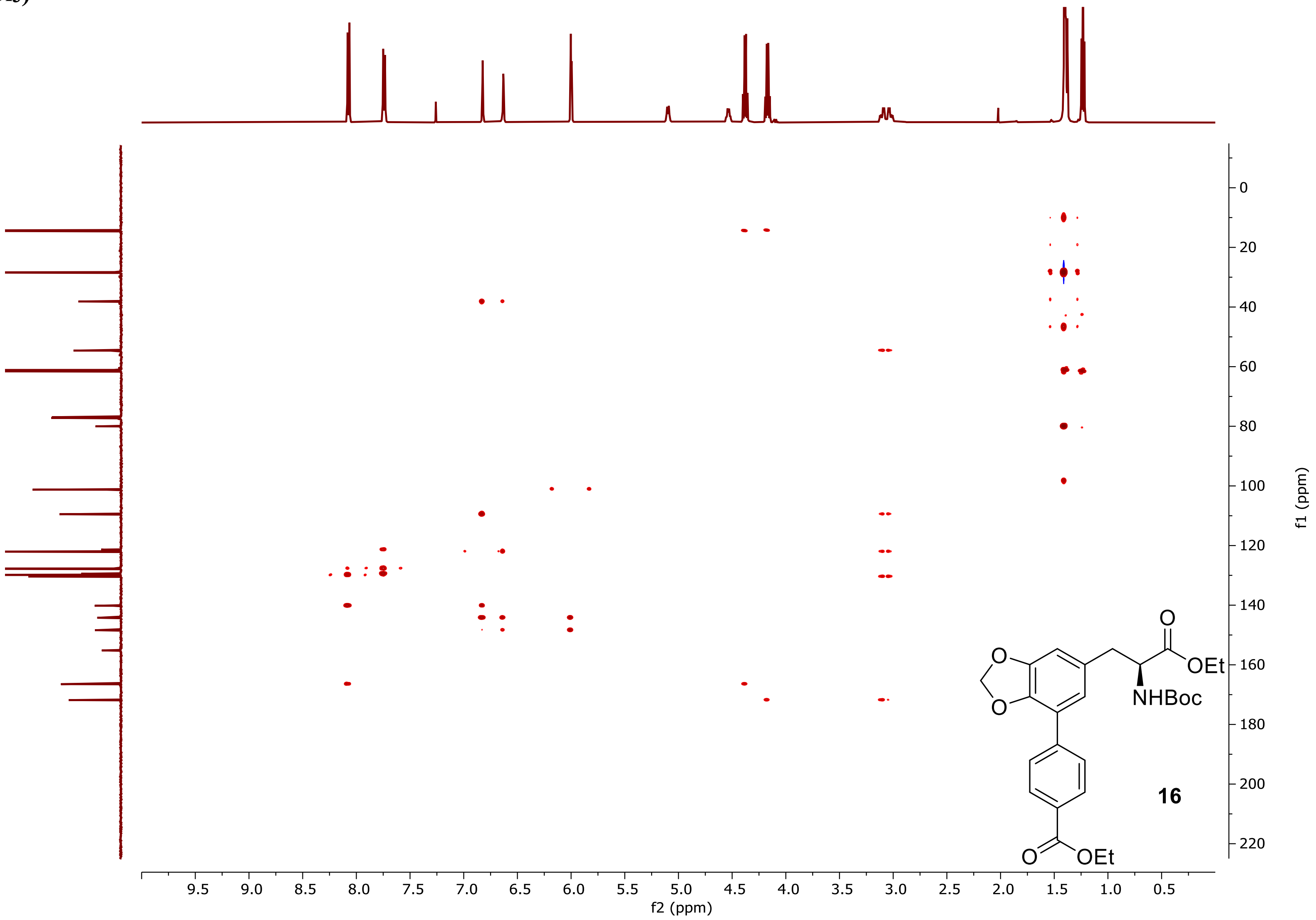

HMBC of ethyl (*S*)-4-(6-(2-((*tert*-butoxycarbonyl)amino)-3-ethoxy-3-oxopropyl) benzo[*d*][1,3]dioxol-4-yl)benzoate (16) – ($CDCl_3$) – 5.5 to 8.5 ppm

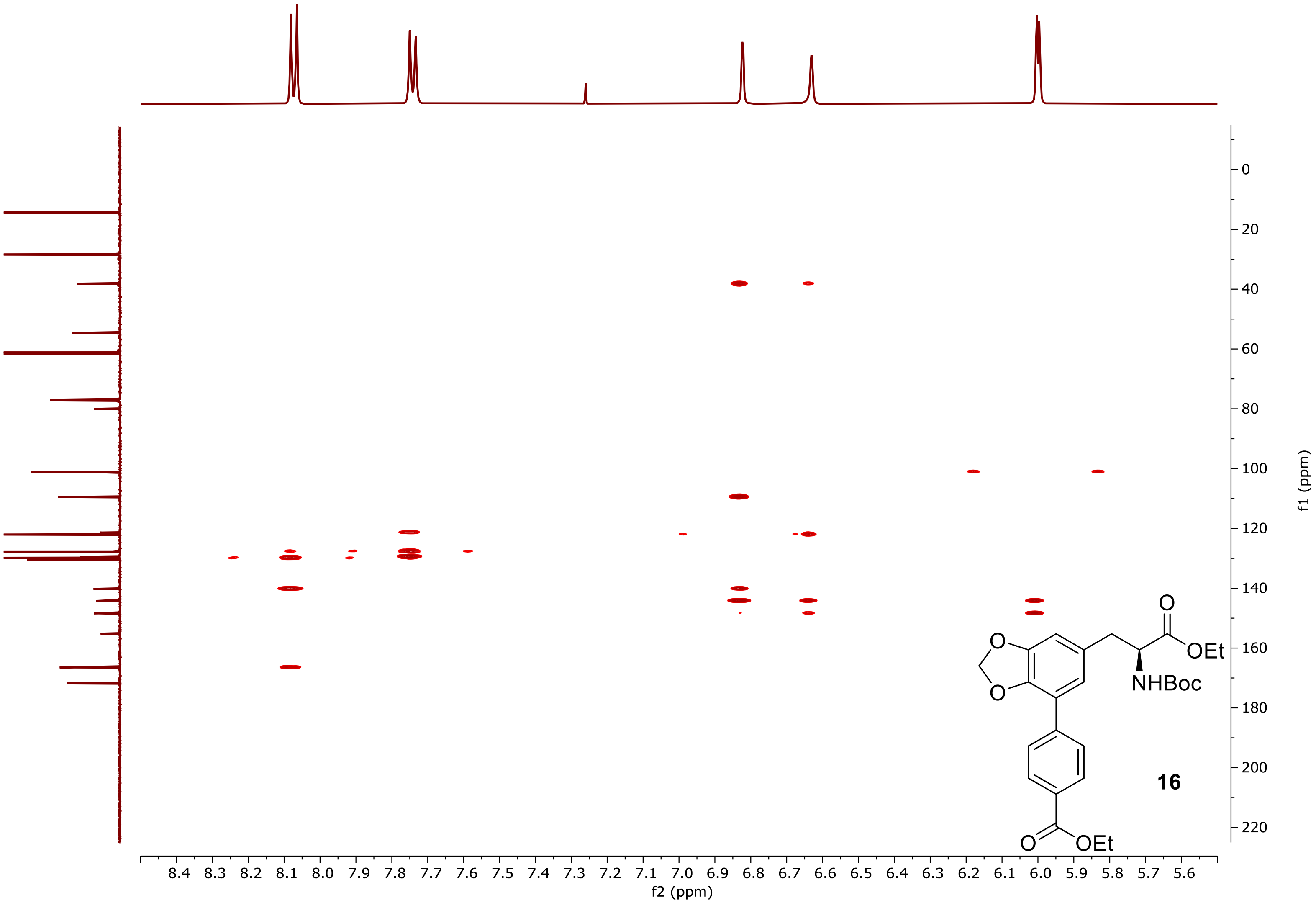

Supplement: Supplementary file 1 [file jo5c00476_si_001.pdf]
